# Platinum-based metal complexes as chloride transporters that trigger apoptosis†

**DOI:** 10.1039/d4sc02115k

**Published:** 2024-06-26

**Authors:** Patrick Wang, Mohamed Fares, Radwa A. Eladwy, Deep J. Bhuyan, Xin Wu, William Lewis, Stephen J. Loeb, Lauren K. Macreadie, Philip A. Gale

**Affiliations:** a School of Chemistry, The University of Sydney NSW 2006 Australia; b School of Pharmacy, The University of Sydney NSW 2006 Australia; c NICM, Research Health Institute, Western Sydney University NSW 2751 Australia; d School of Pharmaceutical Sciences, Xiamen University Xiamen 361102 Fujian China; e Department of Chemistry and Biochemistry, University of Windsor Ontario N9B 3P4 Canada; f School of Chemistry, The University of New South Wales NSW Australia; g School of Mathematical and Physical Sciences, Faculty of Science, University of Technology Sydney Ultimo NSW 2007 Australia philip.gale@uts.edu.au

## Abstract

In this paper we demonstrate that Pt(ii) complexes can function as efficient transmembrane chloride transporters. A series of Pt(ii) metal complexes with urea-appended isoquinoline ligands were synthesised and operate *via* classical hydrogen bonding interactions rather than ligand exchange. A number of the complexes exhibited potent transmembrane chloride activity in vesicle studies, while also showing strong antiproliferative activity in cisplatin-resistant cell lines *via* induction of apoptosis and inhibition of intracellular ROS.

## Abstract 2

In this paper we demonstrate that Pt(ii) complexes can function as efficient transmembrane chloride transporters and can trigger apoptosis.

## Graphical abstract

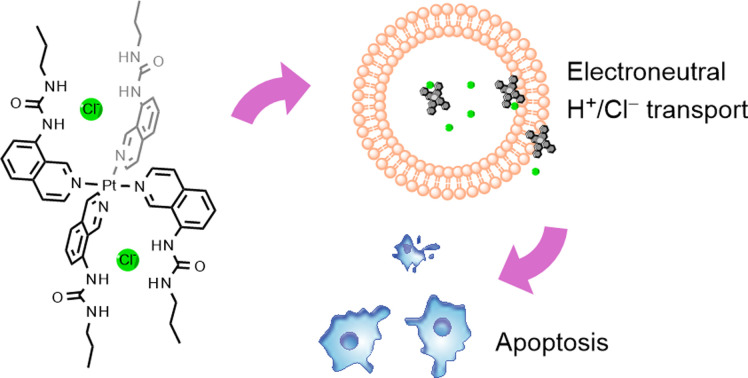

## Introduction

Transmembrane transport of chloride is a critical biological process and has been linked to several diseases.^1,2^ One prominent example is cystic fibrosis, caused by a mutation of the gene that encodes for the cystic fibrosis transmembrane conductance regulator (CFTR) channel, which results in the reduction of chloride flux across epithelial cell membranes.^3^ More recently, chloride transport (in conjunction with H^+^ transport) has been shown to dissipate lysosomal pH levels within cancer cells. Mechanistic studies have shown that HCl transport disrupts autophagy and chloride transport induces apoptosis within cancer cells, which presents another attractive potential application of synthetic anionophores.^4–7^

Research in this area has focused on using receptors containing hydrogen bond donor motifs to bind to anions and facilitate their movement across cell membranes. In particular, urea and amide groups have been used extensively due to their polarised nature, resulting in strong binding interactions.^8–11^ These donor motifs can also be exploited in combination by functionalising molecular scaffolds with multiple hydrogen bond donor groups. Notable examples of potent transporters include *o*-phenylenediamines,^12^ cholapods,^13^ and tetra-urea macrocycles.^14^

Recently, another approach for transporting chloride has involved the exploration of metal complexes as anionophores. The inherent properties of transition metal centres make them attractive scaffolds for building synthetic transporters.^15–17^ Current research into metal–organic anionophores is divided into two strategies; labile complexes that can self-assemble into ion channels or inert complexes that act as discrete transmembrane anion carriers. Complexes capable of functioning as discrete carriers operate *via* two distinct mechanisms. The first is a ligand exchange mechanism, where labile ligands exchange with chloride diffusion across a lipid bilayer. One example of this mechanism was reported for several Pd(ii) complexes with the transport properties of this system studied using the 8-hydroxypyrene-1,3,6-trisulfonic acid (HPTS) assay.^18,19^ Alternatively, the ligands can remain on the metal centre and transport *via* traditional hydrogen bonding interactions between the ligand and anion. This mechanism was reported for an Ir(iii) complex and a number of phosphazane-based complexes with various metal centres, such as Rh(i), Mo(0), and Au(i).^20,21^ The fixed-ligand complexes showed remarkable transport activity in both the Cl^−^/NO_3_^−^ exchange and lucigenin assays.

Pt(ii) has been used in some of the most effective anticancer therapeutics to date.^22–24^ One notable example is cisplatin and, by extension, the platin family of platinum-based drugs. Cisplatin is a platinum-based drug that has found extensive use in the treatment of different cancer types.^25^ The complex relies on the exchange of labile chlorides with water molecules. The aquated complex then enters the nucleus and binds to the purine bases of DNA, forming interstrand cross-links, which inhibit cellular function and result in apoptosis.^26^

Earlier research in our group investigated the use of Pt(ii) complexes in binding halides and larger oxoanions.^27–32^ Previously, we explored a Pt(ii) complex that consisted of urea-functionalised isoquinolines and was shown to bind to sulfate (in 1 : 1 stoichiometry) and various other halide anions (in 1 : 2 stoichiometry).^33^ Using ^1^H-NMR binding studies, the complex displayed significant binding affinity towards sulfate in DMSO-*d*_6_ (*K*_a_ > 10^5^ M^−1^) but also displayed a strong affinity for chloride (*K*_12_ = 2220 M^−1^). More recently, our group investigated a series of Pt(ii) complexes capable of ‘uphill’ OH^−^ transport into vesicles studied using the HPTS assay.^34^ The complex underwent solvolysis of two labile triflate ligands, with the subsequent formation of a membrane-permeable neutral species after deprotonation of the new aqua-ligated complex. The ability to facilitate ‘uphill’ OH^−^ transport demonstrated a precedence for pH disruption by Pt(ii) complexes. The results of these studies inspired the development of a Pt(ii) complex capable of transmembrane chloride transport.

The incorporation of Pt(ii) in coordination complexes has been a fruitful endeavour in the search for anti-cancer therapeutics;^35–37^ however, the transmembrane ionophoric activity of such complexes has been only briefly studied.^38^

**Fig. 1:**
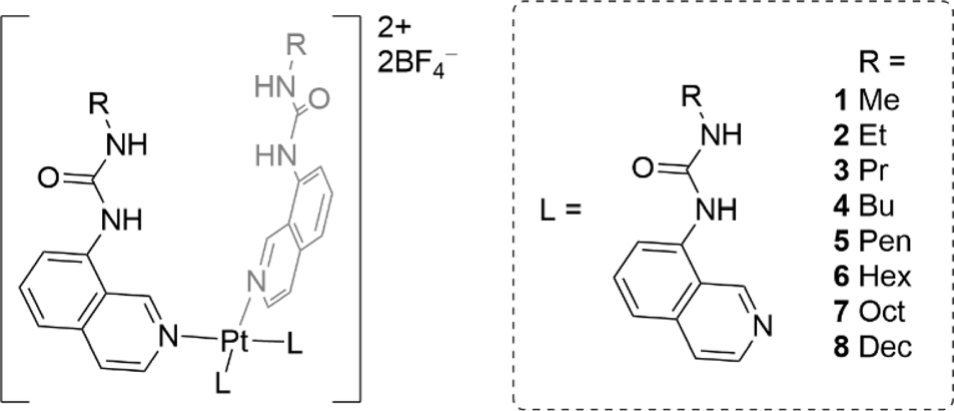
Structures of the Pt(ii) complexes (1–8) studied in this work.

In this work we report a series of eight metal–organic anionophores that contain four urea-functionalised isoquinoline ligands appended to a square planar Pt(ii) centre (Fig. 1). The ligands of the platinum complexes have varying *n*-alkyl chain lengths (from methyl to decyl), which were incorporated to examine to what extent the lipophilicity of the final complex would dictate chloride transport efficiency. The complexes were characterised extensively using spectroscopic and crystallographic methods before being subjected to a rigorous series of chloride transport assays. A number of complexes displayed strong transport activity in both the Cl^−^/NO_3_^−^ exchange assay and the HPTS assay.

## Results and discussion

### Synthetic procedures

**Scheme 1:**
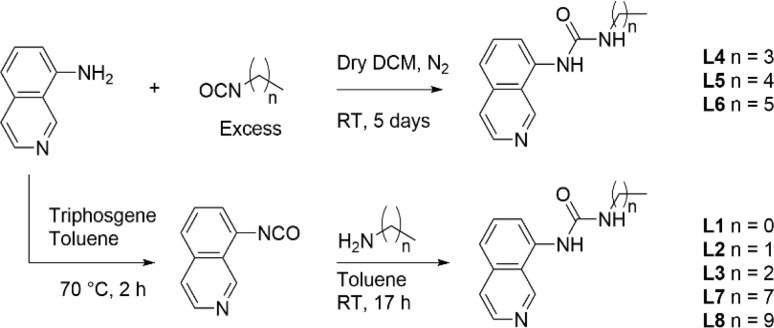
Synthesis of ligands L1–L8 from either an *in situ* isocyanate or from the respective *n*-alkyl isocyanate.

Ligands L1–L8 were synthesised following two distinct synthetic methods. For ligands L4, L5, and L6, 8-aminoisoquinoline was reacted with the respective *n*-alkyl isocyanate (Scheme 1). For the remaining complexes, 8-aminoisoquinoline was converted to an isocyanate *in situ* using triphosgene before condensation with the relevant alkyl amine.

Complexes 1–8 were synthesised by treating dichlorobis(propionitrile)platinum(ii) with the corresponding ligand L1–L8. The reaction mixture was refluxed for 17 h in acetonitrile in the presence of silver tetrafluoroborate. The cooled reaction mixture was then filtered, and the precipitate washed with hot acetonitrile. The final complexes were recovered from the hot filtrate *via* evaporation (see ESI† for details).

### Crystallography

**Fig. 2:**
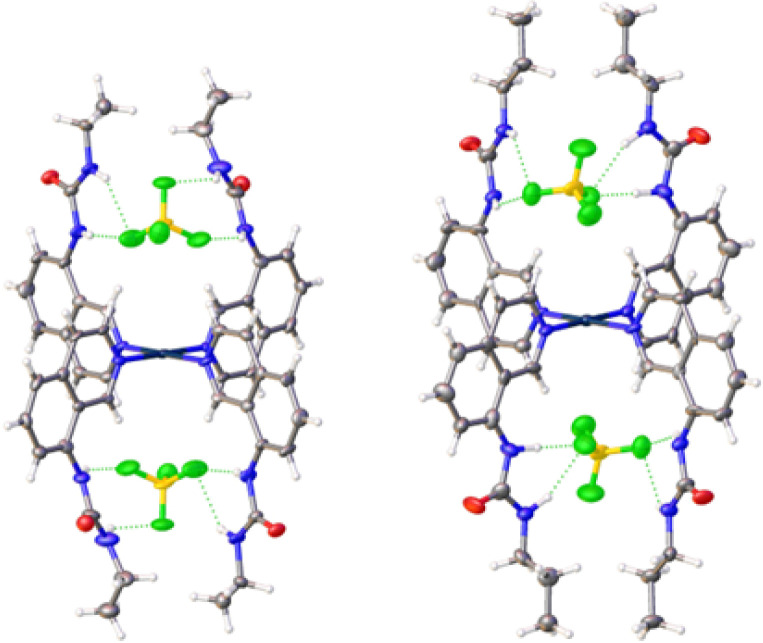
Crystal structures of complexes 2 (left) and 3 (right) showing the positions of the BF_4_^−^ counter-anions in the binding pockets. Thermal ellipsoids are shown at 50% probability.

Crystals of complexes 2, 3 and 5–7 suitable for single crystal X-ray diffraction were obtained by slow diffusion of diethyl ether into a saturated solution of the complex in DMF. All the complexes adopt a 2-up 2-down conformation (Fig. 2 and ESI†) with the BF_4_^−^ anions above and below the platinum centre. The Pt⋯B distance has a narrow range of 4.26–4.49 Å. This conformation was also observed previously for complex 4.^33^ The urea groups show distinct N–H⋯F^−^ hydrogen bonds in all complexes and confirm the proposed binding pocket for anionic guests.

There were no C–H_α_⋯F^−^ hydrogen bonds observed in any of the complexes. This was unexpected as it was documented initially for complex 4 and a large shift of the corresponding proton was also observed in ^1^H NMR titrations (see ESI†). However, an intramolecular hydrogen bond between the urea oxygen and a nearby aromatic C–H was observed in complex 7. This may simply be a result of the different crystal packing forces in the solid-state for these complexes.

### Binding studies

Proton NMR titrations of the complexes with tetrabutylammonium chloride (TBACl) were conducted in a competitive solvent mixture of DMSO-*d*_6_/0.5% H_2_O. A downfield shift was observed for the resonances attributed to the aromatic CH in the 1-position of the isoquinoline ring as well as the two urea NH peaks. The shift in the resonance of these protons agrees with previous titrations of complex 4 (ref. 33) and infers that the chloride anions are H-bonded to these residues and reside in the same pockets as the BF_4_^−^ anions as previously confirmed through X-ray structures of complex 4.^33^

The changes in the chemical shifts of the resonances attributed to the three interacting protons were plotted against the respective equivalents of chloride. The resulting data set was then fit to a 1 : 1 or 1 : 2 binding model using the BindFit v0.5 software.^39^ As a 1 : 2 model naturally results in a better fit to the data, an analysis of the covariance of fit for both models was performed and the ratio between the 1 : 1 and 1 : 2 values compared. A ratio greater than 5 is typically required to confirm that a 1 : 2 binding model is favoured.^40^ The covariance of fit ratio was ambiguous for complexes 1, 2, 4, 6, and 8 as they were less than 5, however, there is literature precedence for a 1 : 2 stoichiometry with chloride for complex 4 and similar Pt(ii) complexes.^27,33^ The results of the ^1^H NMR titrations are displayed in Table 1.

**Table 1:** Transport and anion binding properties of complexes 1–8

		1	2	3	4	5	6	7	8
Binding properties	*K*_11_ (M^−1^)	17 900	16 500	24 500	24 000	90 000	30 500	25 100	42 000
	*K*_12_ (M^−1^)	1400	1500	2300	2000	3700	2200	2800	2000
	*α* a	0.31	0.36	0.38	0.33	0.16	0.29	0.44	0.19
Transport properties	*c* log *P*^b^	2.70	3.14	3.47	3.85	4.32	4.99	6.36	7.37
	Cl^−^/NO_3_ (EC_50_, mol%)^c^	1.66 ± 0.17	0.150 ± 0.014	0.0712 ± 0.0019	—	—	—	—	—
	*n* d	1.66 ± 0.13	1.32 ± 0.12	1.26 ± 0.03	—	—	—	—	—
	HPTS (EC_50_, mol%)^e^	0.092 ± 0.004	0.0085 ± 0.0008	0.005 ± 0.003	0.0111 ± 0.0016	—	—	—	—
	*n*	1.21 ± 0.03	2.2 ± 0.3	2.1 ± 0.2	1.2 ± 0.2	—	—	—	—

a The interaction parameter (*α*) was calculated by multiplying *K*_12_ by four and dividing by *K*_11_. An *α* value <1 indicates negative cooperativity, a value >1 indicates positive cooperativity, while a value of 1 describes non-cooperative binding.

b Log *P* values were calculated using VCC Lab's Pt(ii) drug library tool.

c EC_50_ from the Cl^−^/NO_3_^−^ exchange assay.

d Hill co-efficient is an indicator of the transport stoichiometry between the complex and chloride.

e EC_50_ from the HPTS assay. “—” indicates the complexes were not active enough in the vesicle studies to calculate an EC_50_ value.

All complexes exhibited negative cooperativity throughout the course of the binding studies (Table 1). The negative cooperativity results from the reduced electrostatic attraction and increased electrostatic repulsion of subsequent anionic guests following the first binding event.

The Cl^−^ binding affinities noticeably increased for complexes with longer alkyl chains with an approximate plateau of 2000–3000 M^−1^ for *K*_12_. This was presumably due to the longer alkyl chains forming a more encapsulating coordination pocket for the chloride anion. A similar effect was observed by Engberts and co-workers, where a general increase in binding affinity to trypsin was observed with successively longer *n*-alkyl chains, which was attributed to a combination of hydrophobic interactions and encapsulation of the binding pocket.^41^ Binding affinities began to decrease after the alkyl chain length exceeded five carbons, suggesting the benefit of encapsulation at this length is only marginal. At longer chain lengths, there is likely to be an unfavourable entropic penalty during the process of encapsulation of the binding site.

## Stability in DMSO

The anionophores most likely transport Cl^−^*via* hydrogen bonding rather than a ligand exchange mechanism. Due to the inert nature of the Pt–N bond, it is unlikely that the isoquinoline ligands will be displaced even in the presence of a competing solvent such as DMSO. Previous work has indicated the viability of DMSO as a ligand for Pt(ii) complexes in cytotoxic applications.^42^ However, DMSO is easily displaced by other ligands containing a pyridine group.^43^

**Fig. 3:**
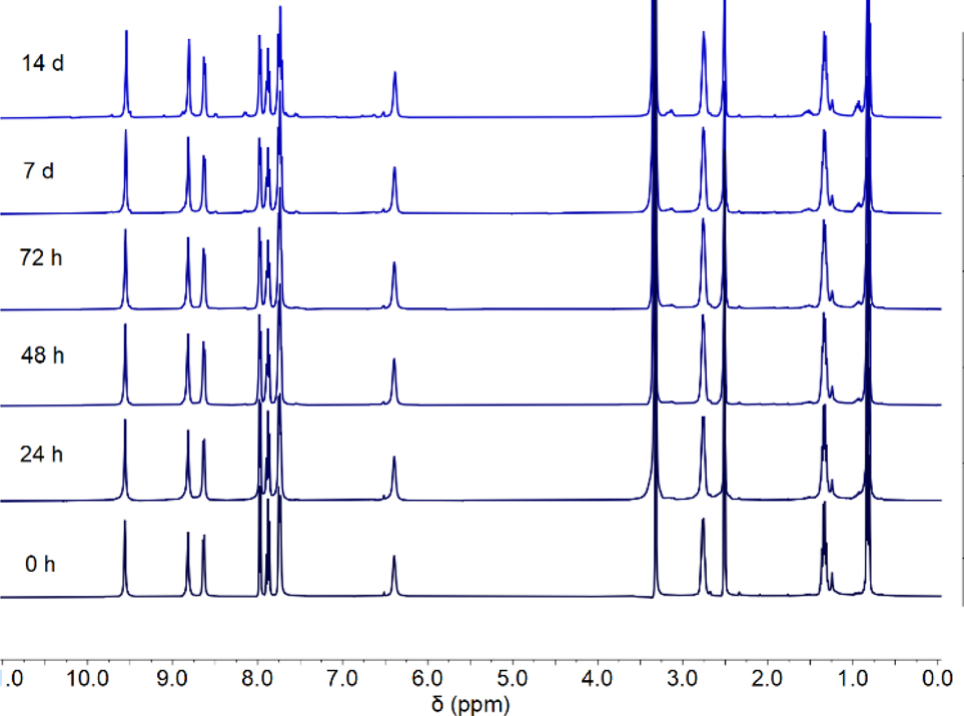
^1^H NMR stability study of complex 3 in DMSO over 14 days.

The complexes were prepared as a DMSO solution for use in the binding studies, vesicle assays, and *in vitro* testing. Since cisplatin is known to be deactivated when prepared in a DMSO solution,^44–46^ we investigated the stability of complexes 1–8 in DMSO over the course of 14 days using ^1^H NMR spectroscopy. A representative stability profile is shown below for complex 3 (Fig. 3). All complexes showed strong stability to ligand exchange in a DMSO environment for up to 7 days with minimal decomposition and up to 14 days with moderate decomposition (Fig. S113–S120†).

### Transport studies

**Fig. 4:**
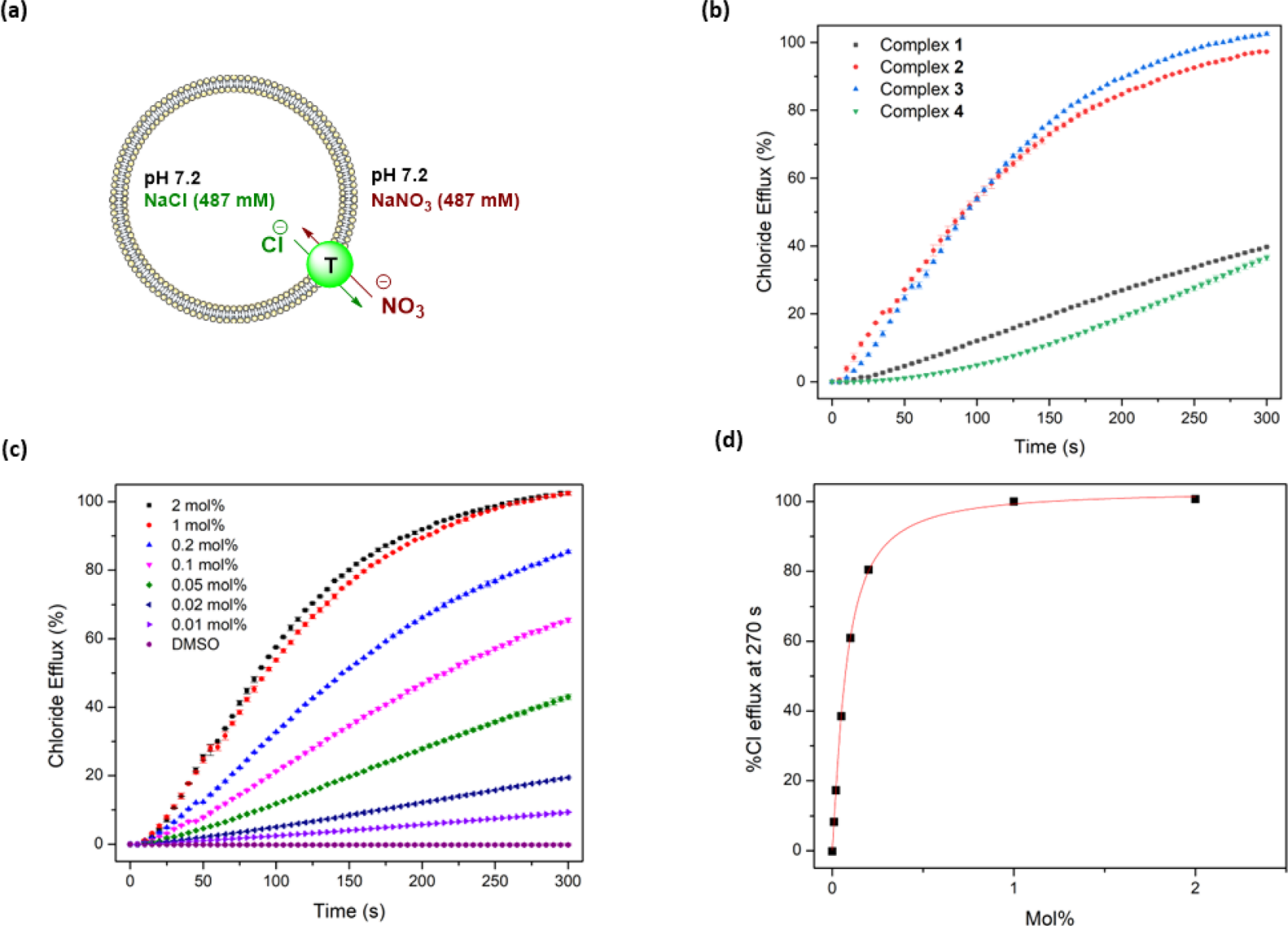
Overview of ISE Cl^−^/NO_3_^−^ exchange assay in 200 nm POPC vesicles loaded with NaCl (487 mM) and suspended in NaNO_3_ (487 mM) at pH 7.20: (a) schematic diagram of the assay showing Cl^−^/NO_3_^−^ exchange mediated by the complex; (b) transport activity of compounds 1–4 at 1 mol%; (c) dose–response curve of complex 3 as a molar percentage relative to lipid concentration; (d) transport activity at 270 s of complex 3, used to calculate an EC_50_ value using the Hill equation.

Following confirmation of the binding affinity of the complexes towards chloride, their transport properties were initially tested using an ion-selective electrode (ISE) Cl^−^/NO_3_^−^ exchange assay using 1-palmitoyl-2-oleoyl-*sn*-glycero-3-phosphocholine (POPC) vesicles (Fig. 4a).^47^ Vesicles were prepared using POPC lipids and loaded with a NaCl (487 mM) solution, buffered to pH 7.2 in sodium phosphate salts (5 mM). The vesicles were then suspended in a NaNO_3_ (487 mM) solution similarly buffered to pH 7.2. Chloride efflux was induced with the addition of the anionophores as a DMSO solution, and the efflux of chloride into the extracellular solution was recorded using the ISE over 300 s. A dose–response curve was collected by evaluating the chloride efflux facilitated by the anionophore at different loading concentrations. This curve was fit to the Hill equation to calculate an EC_50_ value – the concentration of transporter (in mol% relative to the lipid concentration) needed for 50% efflux.^47^

Complexes 1–3 displayed sufficient transport activity to calculate an EC_50_ value, but the longer chain complexes (4–8) precipitated upon addition to the aqueous solution at concentrations required for suitable analysis (Fig. 4b). An increase in transport activity was observed for complexes 1–3 as an extra –CH_2_ was added to the alkyl chain of the urea units (Table 1). Indeed, complex 3 showed the highest transport activity in the series (Fig. 4c and d). One rationale for these findings is the higher binding constants observed in Table 1 which indicate a better ability to bind chloride within the vesicles and thus facilitate anion transport. Furthermore, the extra –CH_2_ groups increase the overall lipophilicity of the complexes, which enhances their transport activity. Lipophilicity has been previously shown to be a significant determinant of the activity of anion transporters, as it directly impacts both the aqueous solubility and the ability to cross the phospholipid bilayer.^19,48–51^ This strategy of coordinating an anion inside a lipophilic sphere has been previously explored in our group, resulting in highly potent chloride transporters.^52^ On the other hand, highly lipophilic complexes typically display diminished aqueous solubility, which is likely responsible for the observed precipitation of complexes 4–8 during the ISE experiments.

The *c* log *P* values of the anionophores were calculated using the VCC Labs Pt(ii) drug library reference tool and are shown in Table 2.^53^ These values support the hypothesis that introducing additional –CH_2_ groups increases lipophilicity. The Hill coefficients (a measure of the transport stoichiometry with chloride) are close to 1.0 for 2 and 3, suggesting one transporter binding to one anion, and the slightly higher Hill coefficient for 1 might be due to transport *via* a dimeric aggregate. These EC_50_ values represent modest transport compared to some of the best organic chloride transporters in the literature.^12,54–56^ However, the complexes show comparable EC_50_ values to those reported by Mao and Wright,^21,57^ which are among the lowest values for metal organic transporters reported to date.

**Table 2:** *In vitro* antiproliferative activity [IC_50_ (μM); the concentration that inhibits cell growth by 50%] of complexes 1–8 and cisplatin against AGS gastric, MCF-7 breast, and MDA-MB-231 breast adenocarcinoma cells using the Alamar Blue assay after 72 h of treatment^a^

Complex	AGS	MCF-7	MDA-MB-231	*c* log *P*
1	—	—	—	2.70
2	1.42 ± 0.25	3.06 ± 0.15	2.2 ± 0.4	3.14
3	1.02 ± 0.10	0.77 ± 0.23	1.4 ± 0.4	3.47
4	1.19 ± 0.18	1.30 ± 0.26	1.39 ± 0.06	3.85
5	1.65 ± 0.07	5.43 ± 0.17	6.48 ± 0.04	4.32
6	—	—	—	4.99
7	—	—	—	6.36
8	—	—	—	7.37
Cisplatin	99.89 ± 7.26	166.4 ± 18.8	163.8 ± 15.7	−2.59

a The values for IC_50_ presented are means ± standard deviations. “—” indicates <50% cell growth inhibition at the highest tested concentration (30 μg mL^−1^). Therefore, the IC_50_ wasn't calculated.

We further investigated the mechanism of chloride transport using a modified version of the Cl^−^/NO_3_^−^ exchange assay. This assay uses potassium salts instead of sodium salts, and the external solution contains gluconate. Gluconate is a large hydrophilic anion that cannot be transported or freely diffuse across the phospholipid bilayer. Under these conditions, chloride transport occurs in the presence of a cationophore, either valinomycin or monensin. Valinomycin strictly uniports K^+^ thus, coupling to the cationophore requires the ability to uniport chloride, known as electrogenic transport. In contrast, monensin facilitates H^+^/K^+^ exchange, meaning that coupling an anionophore to this species requires the co-transport of H^+^/Cl^−^, known as electroneutral transport.

The results indicate that anionophores 1–3 facilitate electroneutral transport *via* coupling to monensin (see ESI†). This was not unexpected due to the difficulty in achieving electrogenic transport. The mechanism of electrogenic transport would require the complex to diffuse across the phospholipid bilayer as an anion decomplexed species which is positively charged. However, this process can easily be disrupted by phosphate headgroup interactions.^58^ Our previous work on complex 4 indicated strong phosphate binding affinities, likely explaining the inability of 4 to facilitate electrogenic transport.

On the other hand, electroneutral transport does not require the complex to diffuse across the bilayer as an anion-free species. Instead, the mechanism can occur in two distinct pathways. The complex can deprotonate and diffuse back over as an anionic species, or the free complex can bind to a deprotonated fatty acid head group. The fatty acid-complex transports over the membrane, which can then be followed by diffusion of the protonated form of the fatty acid back across the membrane thus resulting in proton transport in addition to chloride transport.

As the anionophores exhibited electroneutral HCl co-transport (or the equivalent OH^−^/Cl^−^ exchange), their activity was further investigated using the HPTS assay.^59^ POPC vesicles were loaded with a solution of KCl (100 mM) and HPTS (1 mM) dye, adjusted to pH 7.0 in HEPES buffer (10 mM). HPTS is a water-soluble fluorescent dye that is sensitive to pH changes and changes its excitation wavelength after being deprotonated.

After adding a base pulse of NaOH (5 mM) and raising the external pH to approx. 8, the change in fluorescence emission of HPTS was recorded on a spectrophotometer and analysed to give insights into the direction and magnitude of HCl transport. Due to the lower vesicle concentrations and ionic strength in this assay, transport activity could be detected for complexes 1–5, unlike in the ISE assay (Table 1).

Overall, the EC_50_ values were noticeably lower in the HPTS assay (meaning more effective transport) compared to the ISE assay. This could be attributed to improved solubility conditions in the HPTS assay as EC_50_s could be calculated for up to complex 4, which precipitated out of solution in the ISE assay. The trend of increasing transport activity between complexes 1–3 in the ISE assay was also observed in the HPTS assay, with complex 3 having an EC_50_ of 0.005 mol%. The Hill coefficients in the HPTS assay for complexes 1–3 are different compared to the ISE assay coefficients, which is likely a result of different transporter concentration ranges or different rate-limiting steps in the two assays.

The transport activity decreased for complexes 4 and 5, resulting in a maximum of activity relative to alkyl chain length, peaking at three carbons. This trend is similar to that observed in the ISE assays, suggesting that the poorly water-soluble longer chain complexes are not delivered effectively into the membrane.^48,51^

**Fig. 5:**
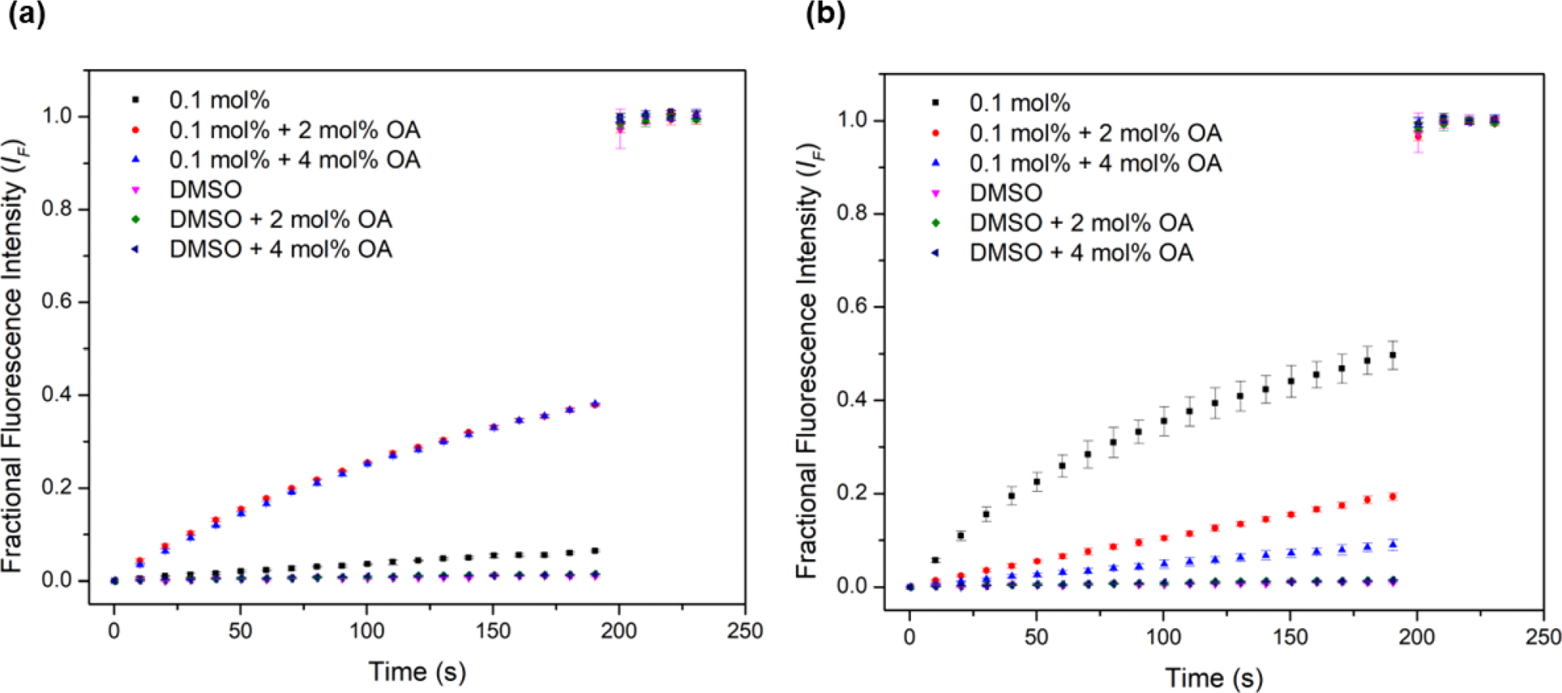
Investigation of fatty acid flip-flop by the addition of oleic acid (2–4 mol%) in the HPTS assay using Avanti Polar Lipids to (a) complex 1 and (b) complex 5.

Urea-based anion transporters are known to facilitate proton transport *via* a transporter deprotonation mechanism or a fatty acid flip-flop mechanism that involves the transporter carrying the deprotonated form of fatty acids across the membrane.^58^ Previous HPTS assays were conducted with POPC lipids that contain a high fatty acid content (up to 2 mol%). In order to investigate whether the fatty acid flip-flop mechanism contributes to the proton transport activity, the HPTS assay was performed using lipids containing a lower fatty acid content before and after the addition of oleic acid (OA) at 2 mol% and 4 mol%. Complex 1 showed the most dramatic increase of the transport rate with OA addition (Fig. 5a), with the activity comparable to the EC_50_ level obtained when higher fatty acid content lipids were used. This confirms that fatty acid flip-flop was the dominant proton transport pathway for complex 1. Complexes 2 and 3 showed similar increases in activity after adding 2 mol% OA, indicating the reliance on fatty acid flip-flop. However, a further addition to 4 mol% OA resulted in a decrease, suggesting competitive binding of oleate to the complex. In contrast, for complexes 4 and 5 the addition of OA did not further increase activity for either complex. Complex 5 showed a sizeable drop after the addition of OA (Fig. 5b). These results indicate that the short chain Pt complexes require fatty acids to transport protons, but the longer chain complexes can facilitate proton transport by themselves, presumably *via* a transporter deprotonation mechanism. It is likely that the deprotonated forms of the short chain Pt complexes cannot effectively cross the membrane due to poor lipophilicity and thus binding of a fatty acid anion to the short chain Pt complexes is required to form a sufficient lipophilic species to cross the membrane. For the long chain complexes, fatty acids are not involved in proton transport but instead compete with chloride binding leading to attenuated activity.

The anion selectivity of the complexes was also investigated in a modified HPTS assay using a method reported recently.^60^ POPC vesicles were loaded with the same buffered solution as typical HPTS vesicles, with the exception of using NaCl (100 mM) instead of KCl. The vesicles were suspended in an isotonic external buffer with Cl^−^, Br^−^, NO_3_^−^, I^−^, or ClO_4_^−^ anions (100 mM, as the respective NaX salts) None of complexes 1–5 showed preferential Cl^−^ selectivity (Fig. S108–S112†). Although large binding constants for Cl^−^ compared to other halides were reported in previous work, the strong hydration of Cl^−^ and its high charge–density renders this anion more challenging to transport than other halides.^33^ Interestingly, the complexes generally showed non-Hofmeister selectivity for anions, with a higher selectivity for halides over ClO_4_^−^, indicating that ClO_4_^−^ is not a good guest for the cationic hydrogen bonding cavity, likely due to the low charge density of ClO_4_^−^. Previously, we have observed that in cationic hydrogen bonding systems, ClO_4_^−^ transport is disfavoured compared with the transport of halides.^61^

## Cell studies

Previous work from our group has shown that synthetic HCl transporters are capable of inducing apoptosis in cancer cells.^4^ As the complexes displayed strong HCl transport activity for a metal organic complex, combined with the platinum(ii) centre, we investigated the cytotoxicity of complexes 1–8. The activity was assessed in three cancer cell lines: AGS gastric adenocarcinoma, MCF-7 breast adenocarcinoma, and MDA-MB-231 triple-negative (ER, PR, and E-cadherin negative) breast adenocarcinoma cells. Their activities were quantified *via* the Alamar Blue cell viability assay.^62^ The complexes were tested against cisplatin as a standard due to the shared motif of a platinum(ii) centre.

Four of the complexes are significantly more active compared to cisplatin (Table 2), with IC_50_ values ranging from 1.02–1.65 μM in AGS cells, 0.77–5.43 μM in MCF-7 cells, and 1.39–6.48 μM in MDA-MB-231 cells. These three cell lines are also known to be more resistant to cisplatin,^63–65^ therefore, the active complexes with significantly lower IC_50_ values than cisplatin are quite promising. The activity of the complexes showed similarities to the transport activities in the vesicle assays, with a similar correlation between potency and *c* log *P*. Complexes 2–5 showed strong antiproliferative activity, whereas complexes 1 and 6–8 were not active enough to calculate an IC_50_ as at their highest tested concentration of (30 μg mL^−1^) they exhibited <50% cell growth inhibition activity. Complex 1 did not show strong antiproliferative activity across all three cell lines which may be a direct result of its hydrophilicity. Similarly, the lack of strong antiproliferative activity in complexes 6–8 may also be a result of their excessive hydrophobicity. This is supported by the lipophilicity values in Table 2, which indicate optimal activity when the *c* log *P* values were between 3.14 and 4.32. When compared with the trends in the EC_50_ values in the ISE and HPTS assay, this further supports the importance of optimal lipophilicity for the activity of the platinum complexes.

To determine whether cell death was the result of a chloride-based transport effect, we investigated the effect of extracellular Cl^−^ on cell death caused by complexes 2–4. AGS cells were incubated with various concentrations of each complex in either a HEPES-buffered solution containing Cl^−^, or an equivalent buffer containing gluconate instead of Cl^−^.^4^ All three complexes showed a significant reduction in antiproliferative activity in Cl^−^ free buffer at concentrations of 5 and 2.5 μg mL^−1^ (Fig. S63†). Under more dilute concentrations, it is likely that the amount of transporter available is not enough to perturb intracellular Cl^−^ concentrations. In contrast, at 10 μg mL^−1^, the complexes may not be solely involved in Cl^−^ transport and could be affecting other cellular processes. Based on their lower IC_50_ values across all three cell lines, complexes 2–4 were selected for further mechanistic studies into the mode of cell death.

### Flow cytometry analysis in AGS cells

Apoptosis is an important pathway of programmed cell death in the treatment of cancer as it minimises the release of inflammatory cellular contents. In contrast, the necrotic pathway of cell death is less desirable because of the uncontrolled release of intracellular contents.^66^ To determine whether the platinum complexes were inducing apoptosis, complexes 2–4 were tested in AGS cells using the Annexin V-CF blue and 7-aminoactinomycin D (7-AAD) flow cytometric assay with 24 h incubation.^62^ Membrane flipping during the early stages of apoptosis can be detected through the preferential binding of Annexin V to phosphotidylserine, which is normally localised in the intracellular side of the phospholipid bilayer. In contrast, 7-AAD preferentially binds to GC-rich sections of double-stranded DNA and is indicative of late-stage apoptosis or necrosis. In combination, these two stains are able to differentiate between necrotic and early- or late-stage apoptotic cells.

**Fig. 6:**
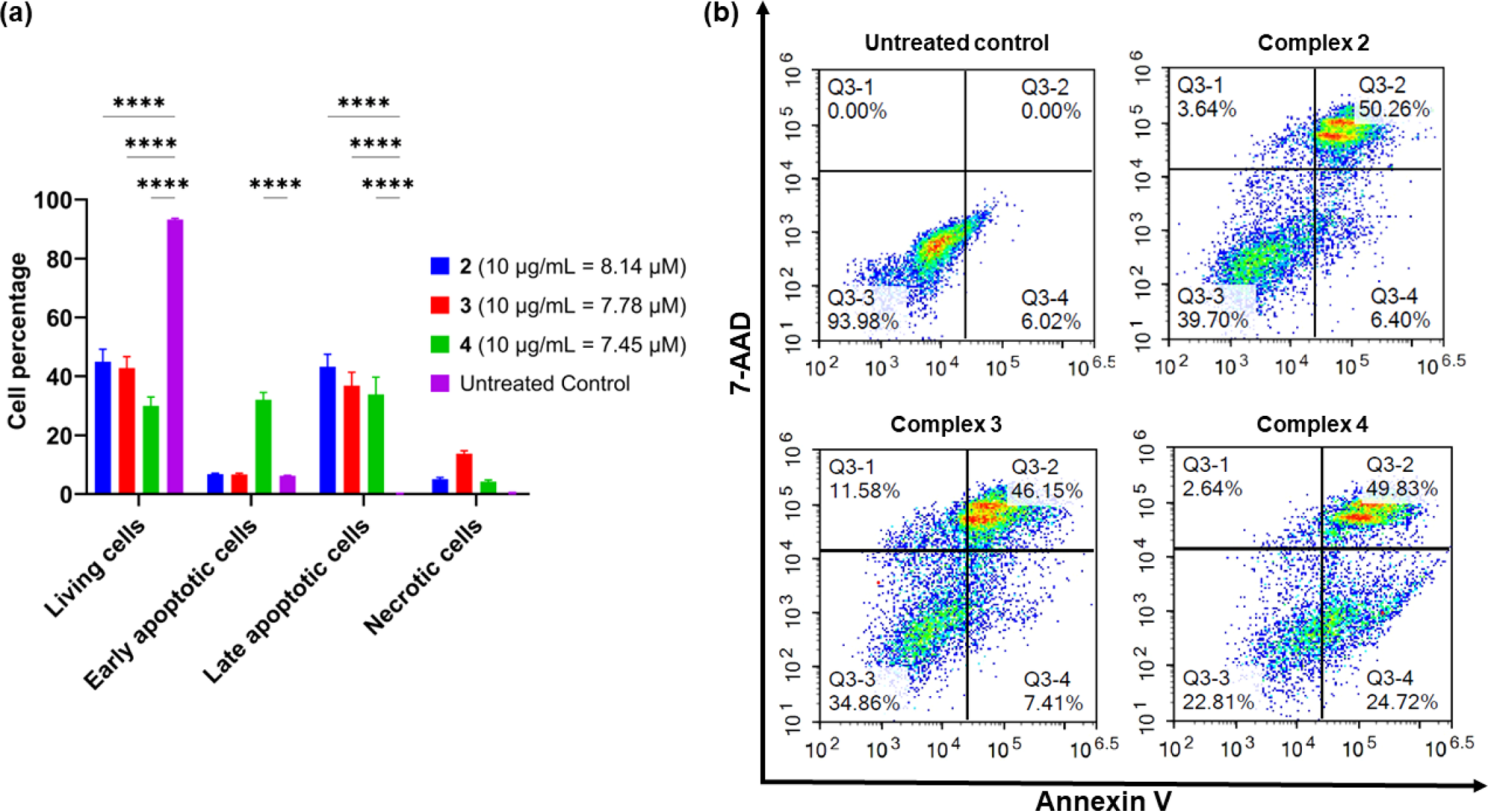
Flow cytometric assessment of apoptotic profiles of the AGS gastric cancer cells after 24 h of treatment with complexes 2–4 (10 μg mL^−1^). (a) The live, early apoptotic, late apoptotic, and necrotic cell percentages after 24 h treatment with complexes 2–4. (b) Representative dot plots from separate flow cytometry experiments (*n* = 3) to analyse the apoptotic profiles of the AGS cells after treatment with complexes 2–4. The vehicle control was implemented using Annexin-V labelled with CF-Blue and the non-vital dye 7-AAD after 24 h of treatment. Asterisks are used to denote statistically significant differences compared to the treated control determined through a two-way ANOVA followed by Tukey's multiple comparisons test within the same group of cells (**** indicates *p* < 0.0001 compared to the untreated control).

All three complexes induced apoptosis after 24 h, with a combined early- and late-stage apoptosis percentage of 57% (complex 2), 54% (complex 3), and 75% (complex 4). A significant decrease (*p* < 0.0001) in the percentage of live AGS cells after treatment compared to the untreated control was observed, indicating the induction of apoptosis in most cells by the complexes (Fig. 6). Complexes 2 and 4 showed desirable activity as they exhibited very low necrotic activity (4% and 3% respectively) compared to complex 3 (12%). This is an important consideration due to the release of tumour-promoting cellular contents in necrotic cell death.^67,68^ As a result of these differences, recent platinum-based anticancer therapeutics aim to induce apoptosis in tumour cells as their preferred method of cell death.^69,70^

### Reactive oxygen species studies

Reactive oxygen species (ROS) levels are elevated in cancer cells as a result of increased cellular activity, which simultaneously promote further tumour growth.^71^ However, elevated ROS levels are also capable of inducing cell death and are sometimes a target for anticancer drugs. Previous studies have shown that platinum-based complexes, especially cisplatin, induce higher levels of ROS within cancer cells and are capable of inducing cell death as a direct result.^72^

**Fig. 7:**
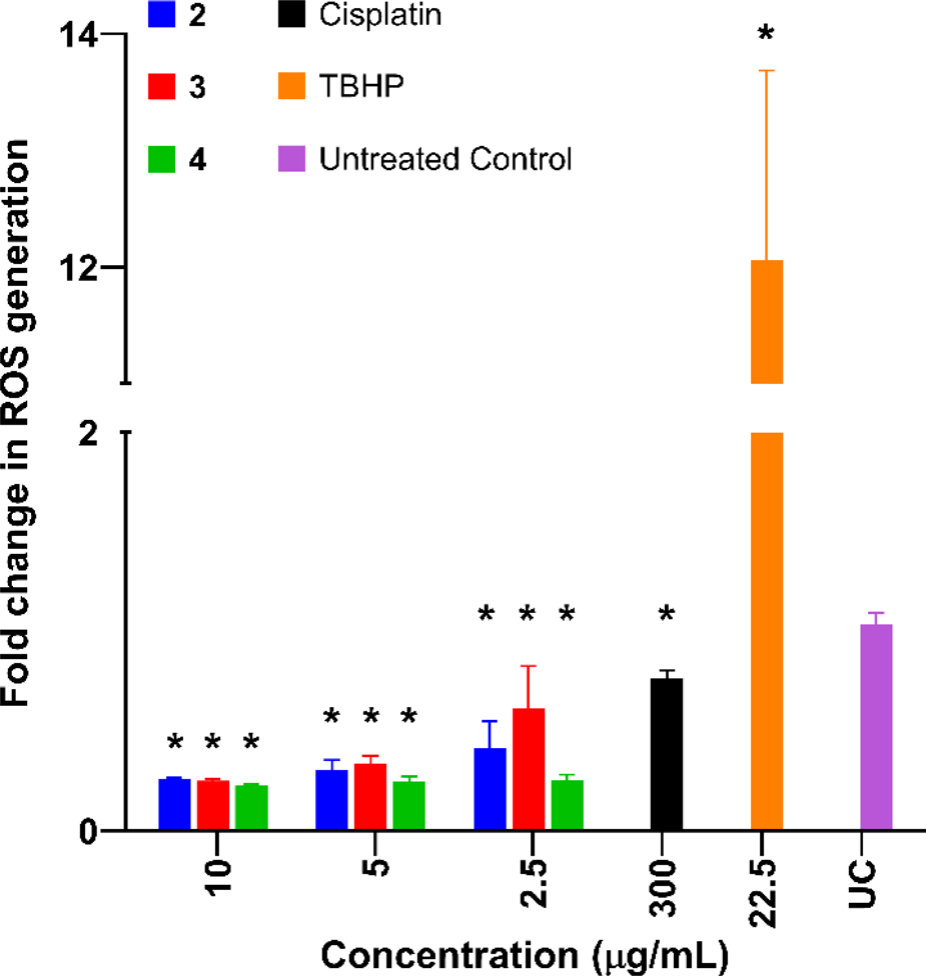
Relative ROS production in AGS cells compared to the negative control, upon treatment with complexes 2–4, cisplatin (Cis), and the positive control *tert*-butyl hydroperoxide (TBHP). Values expressed as mean standard deviation (SD) (*n* = 3), One-way ANOVA was used for multiple comparisons. Values are expressed as mean ± SD. Asterisks indicate significant differences (*P* ≤ 0.0001) in the fold increase in ROS generation compared to the untreated control and doxorubicin, respectively according to Dunnett's multiple comparison test.

Complexes 2–4 were tested in AGS cells and compared to cisplatin (Cis) and *tert*-butyl hydroperoxide (TBHP) in order to investigate the effects of the complexes on ROS production levels. All complexes exhibited a significant decrease in ROS production compared to the untreated control (Fig. 7). This was unexpected as cisplatin and similar-platin drugs are known to promote the generation of ROS as a precursor to apoptosis and is a major contributor to the cytotoxicity of the drug family.^69,72,73^ The lack of ROS generation by complexes 2–4 is also atypical of other cytotoxic Pt(ii) complexes which induce apoptosis *via* localised generation of ROS within cancer cells.^74–76^ These results suggest an alternative method of apoptosis caused by these new Pt(ii) complexes.

## Conclusions

We present a series of Pt(ii) complexes bearing isoquinoline ligands and have demonstrated, for the first time, their ability to transport the biologically relevant chloride anion across a phospholipid bilayer in synthetic vesicles. We have shown that varying the lipophilicity of the complexes plays a significant role in their transport efficiency, with a propyl chain length being the most effective for chloride transport. These anionophores display potent antiproliferative activity against cisplatin-resistant cancer cell lines, indicating their potential use as a therapeutic. We hope the work presented here can provoke inspiration in the design of new Pt(ii) complexes for antiproliferative activity. Further experimentation with the choice of ligands may strike a balance between inert and labile ligands, potentially providing new pathways for treating cancer.

## Data availability

The data supporting this article have been included as part of the ESI.†

## Author contributions

PW conducted the synthesis, binding, anion transport studies, and wrote the manuscript. XW provided guidance on interpreting the anion transport studies. MF, WL, and LKM conducted the crystallography experiments, resolved the structures, and wrote the crystallography experimental section. RAE and DJB conducted the cellular studies and provided figures from the data. SJL provided guidance on the synthesis pathway. PAG conceived the project and directed the research.

## Conflicts of interest

There are no conflicts to declare.

## Supplementary Material

SC-015-D4SC02115K-s001

SC-015-D4SC02115K-s002
