# Supplementary material for: Platinum-based metal complexes as chloride transporters that trigger apoptosis
Source: Chem Sci. 2024 Jun 26;15(29):11584–93. doi: 10.1039/d4sc02115k (PMC11268493; doi:10.1039/d4sc02115k)
Supplement: SC-015-D4SC02115K-s001 [file SC-015-D4SC02115K-s001.pdf]

Electronic Supplementary Material (ESI) for Chemical Science.
This journal is © The Royal Society of Chemistry 2024

Supporting Information

Platinum-based metal complexes as chloride transporters that trigger apoptosis

Patrick Wang,[a] Mohamed Fares,[b] Radwa A. Eladwy,[c] Deep J. Bhuyan,[c] Xin Wu,[d] William Lewis,[a] Stephen J. Loeb,[e] Lauren K. Macreadie[f] and Philip A. Gale*[g]

[a] School of Chemistry, The University of Sydney, NSW 2006, Australia.

[b] School of Pharmacy, The University of Sydney, NSW 2006, Australia.

[c] NICM, Research Health Institute, Western Sydney University, NSW 2145, Australia

[d] School of Pharmaceutical Sciences, Xiamen University, Xiamen 361102, Fujian, China.

[e] Department of Chemistry and Biochemistry, University of Windsor, Ontario, Canada.

[f] School of Chemistry, The University of New South Wales, NSW, Australia.

[g] School of Mathematical and Physical Sciences, Faculty of Science, University of Technology Sydney, Ultimo NSW 2007, Australia. philip.gale@uts.edu.au

Contents

Synthesis ... 3
Synthetic Procedures ... 3
Characterisation spectra ... 15
Experimental procedures ... 51
Binding studies ... 51
^{1}H NMR Titrations ... 51
DMSO Stability Studies ... 52
Transport studies ... 56
General Vesicle Preparation ... 56
ISE Cl^{-}/NO_3^{-} Exchange Assay ... 56
ISE Cl^{-}/SO_4^{2-} Exchange Assay ... 57
Cationophore Coupled Transport ... 57
HPTS Assay ... 57
HPTS Oleic Acid Assay ... 59
Anion Selectivity Assay ... 59
Cell Studies ... 59
Cell Culture ... 59
Alamar Blue Assay ... 60
Flow cytometric analysis of apoptosis in the AGS gastric cancer cells using the most active complexes **2**–**4**. ... 60
Reactive Oxygen Species (ROS) Assay ... 61
Crystallography ... 62
Binding Data and Fittings ... 64
Covariance of Fit Calculations ... 72
Transport data ... 73
ISE Cl^{-}/NO_3^{-} Exchange ... 73
ISE Cl^{-}/SO_4^{2-} Exchange ... 76
ISE Cationophore Coupled Transport ... 76
HPTS Assay ... 78
HPTS Oleic Acid Assay ... 83
Anion Selectivity Assay ... 85
DMSO Stability Studies ... 88
References ... 92

Synthesis

Synthetic Procedures

Chemicals were purchased from commercial sources and used without purification unless otherwise stated. Anhydrous solvents were collected from an Inert Corp PureSolv MD7 solvent purification system.

NMR spectra were recorded on Bruker Avance NEO 300 or Bruker AVIII 400 NMR spectrometers in the indicated solvent at 298 K. Chemical shifts are reported relative to the deuterated solvent used. Spectra resolution was either 300 or 400 MHz for ^{1}H NMR, 101 MHz for ^{13}C NMR, and 86 MHz for ^{195}Pt NMR.

Low-resolution mass spectrometry (LR-MS) was collected using positive and negative electrospray ionisation on a Bruker amaZon SL mass spectrometer. High-resolution mass spectrometry (HR-MS) was collected on a Bruker Solarix 2XR mass spectrometer by Sydney Analytical. All mass spectrometry data are reported as m/z.

1-(isoquinolin-8-yl)-3-methylurea (L1)

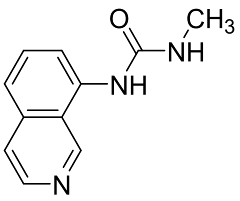

8-Aminoisoquinoline (1.0068 g, 6.983 mmol) was dissolved in dry THF (50 mL) with mild heating at 40 °C before adding triphosgene (0.9483 g, 3.196 mmol). TEA (3.65 mL) was immediately added afterwards before stirring at 70 °C for 3 h. The reaction was cooled to RT before adding pentane (2 mL) and stirring for 10 min. The mixture was filtered, and the pentane removed from the filtrate. A 2 M solution of methylamine in THF (4 mL, 8 mmol) was added to the filtrate, and the solution was stirred at RT for 18 h. The mixture was bubbled with N_2 for 10 min before it was filtered and concentrated to a brown oil, redissolved in minimal THF, then precipitated out with hexane. The solid was filtered and washed with excess water until the filtrate became colourless. The final product was obtained via flash chromatography, eluting with ethyl acetate, as a pale-yellow powder. Yield 0.3677 g (26 %). Melting point: 195.2 – 198.7 °C.

^{1}H NMR (400 MHz, DMSO-d_6) δ: 9.49 (s, 1H), 8.93 (s, 1H), 8.48 (d, J = 5.6 Hz, 1H), 8.11 (d, J = 7.8 Hz, 1H), 7.76 (d, J = 5.7 Hz, 1H), 7.66 (t, J = 8.0 Hz, 1H), 7.54 (d, J = 8.1 Hz, 1H), 6.47 (q, J = 4.7 Hz, 1H), 2.72 (d, J = 4.6 Hz, 3H). **^{13}C NMR** (101 MHz, DMSO-d_6) δ: 155.89, 146.80, 142.63, 136.39, 135.95, 130.85, 120.50, 120.41, 120.03, 117.08, 26.26. **LR-MS** (ESI+) [M+H]$^+$: 201.95 m/z. **HR-MS** (ESI+) calculated for $C_{11}H_{11}N_3O$ [M+H]$^+$: 202.09804 m/z, found [M+H]$^+$: 202.09716 m/z.

1-ethyl-3-(isoquinolin-8-yl)urea (L2)

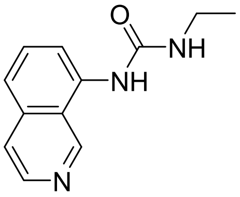

8-aminoisoquinoline (0.9834 g, 6.821 mmol) was suspended in dry toluene (150 mL) with mild heating at 40 °C. Triphosgene (0.9604 g, 3.236 mmol) was added to the mixture, followed immediately by TEA (3.8 mL). The mixture was stirred at 70 °C for 2 h then cooled to RT. Pentane (2 mL) was added and the mixture was stirred for 10 min before filtering. The pentane was removed from the filtrate, and the solution cooled to 0 °C before ethylamine (6 mL), chilled to −20 °C, was added. The temperature was maintained at 0 °C for 3 h before it was slowly warmed to RT and stirred for 20 h. The mixture was filtered and the filtrate concentrated to a brown oil, which was dissolved in minimal toluene. A crude product was precipitated out with hexane (100 mL). The two precipitates were combined and purified via flash chromatography, eluting with ethyl acetate. The fractions were concentrated and the final product recrystallised from hot ethanol as a pale-yellow powder. Yield 0.4721 g (32 %). Melting point: 190.8 – 195.3 °C.

^{1}H NMR (400 MHz, DMSO-d_6) δ: 9.49 (s, 1H), 8.87 (s, 1H), 8.48 (d, J = 5.7 Hz, 1H), 8.13 (d, J = 7.7 Hz, 1H), 7.76 (d, J = 5.7 Hz, 1H), 7.66 (t, J = 7.9 Hz, 1H), 7.54 (d, J = 8.2 Hz, 1H), 6.58 (t, J = 5.6 Hz, 1H), 3.19 (p, J = 6.8 Hz, 2H), 1.11 (t, J = 7.2 Hz, 3H). **^{13}C NMR** (101 MHz, DMSO-d_6) δ: 155.15, 146.70, 142.63, 136.39, 135.96, 130.86, 120.43, 120.38, 119.93, 116.84, 34.09, 15.31. **LR-MS** (ESI+) [M+H]$^+$: 216.17 m/z. **HR-MS** (ESI+) calculated for $C_{12}H_{14}N_3O$ [M+H]$^+$: 216.11314 m/z, found [M+H]$^+$: 216.11279 m/z.

1-(isoquinolin-8-yl)-3-propylurea (L3)

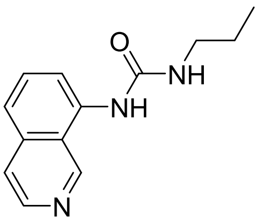

8-aminoisoquinoline (1.0148 g, 7.038 mmol) was suspended in dry toluene (150 mL) with mild heating at 40 °C. Triphosgene (0.9206 g, 3.102 mmol) was added to the mixture, followed immediately by TEA (3.7 mL). The mixture was stirred at 70 °C for 2.5 h then cooled to RT. Pentane (2 mL) was added and the mixture stirred for 10 min before it was filtered. The pentane was removed from the filtrate and propylamine (0.7 mL) was added dropwise to the solution and stirred at RT for 22 h. A precipitate formed, which was filtered and washed with excess hexane. The final product was obtained via flash chromatography, eluting with ethyl acetate, as a white powder. Yield 0.4420 g (27 %). Melting point: 165.0 – 165.9 °C.

^{1}H NMR (400 MHz, DMSO-d_6) δ: 9.50 (s, 1H), 8.88 (s, 1H), 8.48 (d, *J* = 5.6 Hz, 1H), 8.15 (d, *J* = 7.7 Hz, 1H), 7.76 (d, *J* = 5.7 Hz, 1H), 7.66 (t, *J* = 8.0 Hz, 1H), 7.53 (d, *J* = 8.1 Hz, 1H), 6.62 (t, *J* = 5.6 Hz, 1H), 3.13 (q, *J* = 6.6 Hz, 2H), 1.50 (h, *J* = 7.3 Hz, 2H), 0.92 (t, *J* = 7.4 Hz, 3H). **^{13}C NMR** (101 MHz, DMSO-d_6) δ: 155.24, 146.66, 142.64, 136.41, 135.96, 130.88, 120.45, 120.32, 119.89, 116.73, 40.99, 22.87, 11.33. **LR-MS** (ESI+) $[M+Na]^+$: 252.17 m/z. **HR-MS** (ESI+) calculated for $C_{13}H_{16}N_3O$ $[M+H]^+$: 230.12879 m/z, found $[M+H]^+$: 230.18278 m/z.

1-butyl-3-(isoquinolin-8-yl)urea (L4)

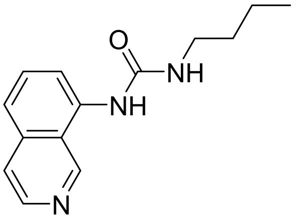

8-aminoisoquinoline (0.2498 g, 1.733 mmol) was dissolved in dry DCM (20 mL). The flask was purged with N_2 for 10 min before adding butyl isocyanate (0.95 mL) and stirring at RT for 5 days. Hexane (100 mL) was added to the solution and the resulting precipitate was collected, washed with hexane (30 mL) and dried to give the product as a yellow powder. Yield 0.3329 g (79 %).

^{1}H NMR (300 MHz, DMSO-d_6) δ: 9.51 (s, 1H), 8.90 (s, 1H), 8.50 (s, 1H), 8.15 (d, J = 7.2 Hz, 1H), 7.80 (d, J = 5.7 Hz, 1H), 7.68 (t, J = 8.0 Hz, 1H), 6.62 (t, J = 5.6 Hz, 1H), 3.16 (q, J = 6.6 Hz, 2H), 1.55 – 1.27 (m, 4H), 0.92 (t, J = 7.2 Hz, 3H). **LR-MS** (ESI+) $[M+H]^+$: 244.02 m/z.

1-(isoquinolin-8-yl)-3-pentylurea (L5)

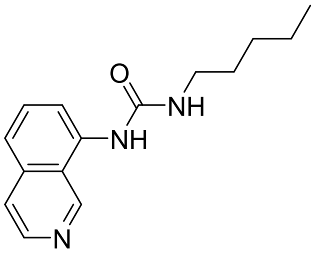

8-aminoisoquinoline (0.2006 g, 1.391 mmol) was placed into a small flask and the atmosphere evacuated before pentyl isocyanate (0.9 mL) was added. The solution was stirred at 30 °C for 4 h before dry DCM (7 mL) was added and the solution stirred at RT for 6 d. Hexane (150 mL) was added and the resulting precipitate filtered and washed with excess hexane. The product was purified via flash chromatography in pure EtOAc and dried to give a white powder. Yield 0.2080 g (58 %). Melting point: 141.9 – 142.7 °C.

^{1}H NMR (400 MHz, DMSO-d_6) δ: 9.50 (s, 1H), 8.87 (s, 1H), 8.48 (d, J = 5.7 Hz, 1H), 8.14 (d, J = 7.4 Hz, 1H), 7.76 (d, J = 5.7 Hz, 1H), 7.66 (t, J = 8.0 Hz, 1H), 7.53 (d, J = 8.1 Hz, 1H), 6.60 (t, J = 5.6 Hz, 1H), 3.15 (q, J = 6.7 Hz, 2H), 1.49 (p, J = 6.9 Hz, 2H), 1.38 – 1.27 (m, 4H), 0.90 (t, J = 6.8 Hz, 3H). **^{13}C NMR** (101 MHz, DMSO-d_6) δ: 155.71, 147.15, 143.14, 136.91, 136.46, 131.38, 120.95, 120.81, 120.38, 117.20, 29.79, 29.10, 22.35, 14.41. **LR-MS** (ESI+) $[M+H]^+$: 258.12 m/z. **HR-MS** (ESI+) calculated for $C_{15}H_{19}N_3O$ $[M+H]^+$: 258.16009 m/z, found $[M+H]^+$: 258.16017 m/z.

1-hexyl-3-(isoquinolin-8-yl)urea (L6)

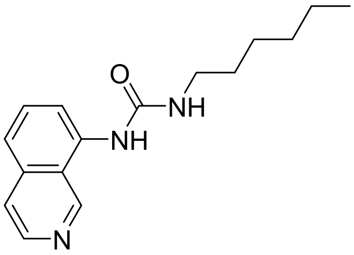

8-aminoisoquinoline (0.2005 g, 1.391 mmol) was placed into a small flask and the atmosphere evacuated before hexyl isocyanate (1 mL) was added. The solution was stirred

at 40 °C for 3 h before another aliquot of isocyanate (0.5 mL) was added and the solution stirred at 50 °C for 1 h. Dry DCM (7 mL) was added and the solution stirred at RT for 2.5 days. Hexane (100 mL) was added to the solution and the precipitate filtered, washed with hexane (30 mL) and dried to give the product as a light brown powder. Yield 0.2854 g (76 %). Melting point: 123.0 – 126.1 °C.

^{1}H NMR (400 MHz, DMSO-d_6) δ: 9.51 (s, 1H), 8.89 (s, 1H), 8.48 (d, *J* = 5.7 Hz, 1H), 8.15 (d, *J* = 7.7 Hz, 1H), 7.76 (d, *J* = 5.7 Hz, 1H), 7.66 (t, *J* = 8.0 Hz, 1H), 7.53 (d, *J* = 8.1 Hz, 1H), 6.63 (t, *J* = 5.5 Hz, 1H), 3.15 (q, *J* = 6.5 Hz, 2H), 1.53 – 1.42 (m, 2H), 1.37 – 1.25 (m, 6H), 0.88 (t, 3H). **^{13}C NMR** (101 MHz, DMSO-d_6) δ: 155.72, 147.16, 143.08, 136.95, 136.47, 131.40, 120.96, 120.80, 120.35, 117.18, 39.67, 31.50, 30.08, 26.57, 22.56, 14.39. **LR-MS** (ESI+) $[M+H]^+$: 272.23 m/z. **HR-MS** (ESI+) calculated for $C_{16}H_{21}N_3O$ $[M+H]^+$: 272.17574 m/z, found $[M+H]^+$: 272.17572 m/z.

1-(isoquinolin-8-yl)-3-octylurea (L7)

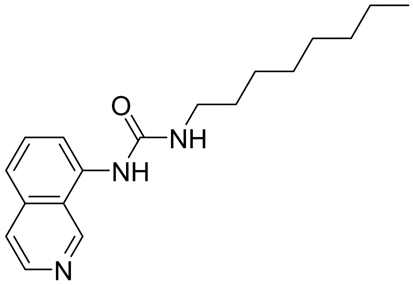

8-aminoisoquinoline (0.5066 g, 3.514 mmol) was suspended in dry toluene (60 mL) before triphosgene (0.5455 g, 1.838 mmol) was added, followed immediately by TEA (1.8 mL). The solution was stirred at 70 °C for 2 h before pentane (1 mL) was added. The mixture was cooled for 10 min before the precipitate filtered, and the pentane removed from the filtrate. Octylamine (0.7 mL) was added and the solution refluxed at 125 °C for 24 h. The solution was cooled to RT then concentrated to a dark oil, which solidified into a dark solid over 48 h. The solid was collected, washed with hexane (3 × 30 mL), then purified via flash chromatography, eluting with 4 % MeOH in DCM. The final product was obtained as a pale powder. Yield (0.4352 g, 41 %). Melting point: 128.6 – 132.0 °C.

^{1}H NMR (400 MHz, DMSO-d_6) δ: 9.50 (s, 1H), 8.86 (s, 1H), 8.48 (d, *J* = 5.6 Hz, 1H), 8.15 (d, *J* = 7.7 Hz, 1H), 7.76 (d, *J* = 5.6 Hz, 1H), 7.66 (t, *J* = 8.0 Hz, 1H), 7.53 (d, *J* = 8.1 Hz, 1H), 6.60 (t, *J* = 5.6 Hz, 1H), 3.15 (q, *J* = 6.9 Hz, 2H), 1.48 (p, *J* = 6.8 Hz, 2H), 1.35 – 1.21 (m, 10H), 0.89 – 0.82

(m, 3H). **^{13}C NMR** (101 MHz, DMSO-d_6) δ: 155.70, 147.14, 143.12, 136.92, 136.46, 131.38, 120.95, 120.79, 120.36, 117.17, 39.66, 31.72, 30.11, 29.22, 29.17, 26.90, 22.55, 14.40. **LR-MS** (ESI+) $[M+H]^+$: 300.29 m/z. **HR-MS** (ESI+) calculated for $C_{18}H_{25}N_3O$ $[M+H]^+$: 300.20704 m/z, found $[M+H]^+$: 300.20700 m/z.

1-decyl-3-(isoquinolin-8-yl)urea (L8)

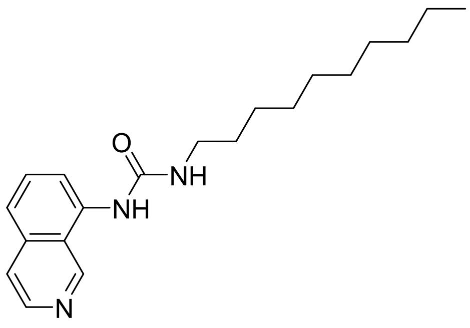

8-aminoisoquinoline (0.5729 g, 3.974 mmol) was suspended in dry toluene (100 mL) before triphosgene (0.6475 g, 2.182 mmol) was added, followed immediately by TEA (2.1 mL). The mixture was stirred at 70 °C for 2 h before pentane (1 mL) was added. The mixture was cooled for 10 min before the precipitate filtered and the pentane removed from the filtrate. Decylamine (0.88 mL) was added and the solution refluxed at 130 °C for 21 h. The solution was cooled to RT then concentrated to a dark oil, which was purified via flash chromatography, eluting with 4 % MeOH in DCM. The final product was obtained as light-orange flakes. Yield (0.2338 g, 18 %). Melting point: 122.7 – 126.2 °C.

^{1}H NMR (400 MHz, DMSO-d_6) δ: 9.50 (s, 1H), 8.87 (s, 1H), 8.48 (d, J = 5.6 Hz, 1H), 8.14 (d, J = 7.7 Hz, 1H), 7.76 (d, J = 5.7 Hz, 1H), 7.65 (t, J = 7.9 Hz, 1H), 7.53 (d, J = 8.2 Hz, 1H), 6.60 (t, J = 5.6 Hz, 1H), 3.15 (q, J = 6.5 Hz, 2H), 1.54 – 1.43 (m, 2H), 1.38 – 1.17 (m, 14H), 0.84 (t, J = 6.4 Hz, 3H). **^{13}C NMR** (101 MHz, DMSO-d_6) δ: 155.70, 147.15, 143.13, 136.91, 136.46, 131.37, 120.95, 120.79, 120.36, 117.17, 31.76, 30.10, 29.51, 29.44, 29.24, 29.16, 26.88, 22.55, 14.40. **LR-MS** (ESI+) $[M+H]^+$: 328.29 m/z. **HR-MS** (ESI+) calculated for $C_{20}H_{29}N_3O$ $[M+H]^+$: 326.22269 m/z, found $[M+H]^+$: 326.22400 m/z.

Dichlorobis(propanenitrile)platinum(II) ([$PtCl_2(EtCN)_2$])

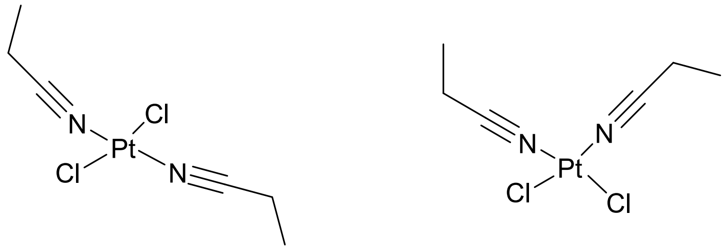

Potassium tetrachloroplatinate(II) (0.5147 g, 1.240 mmol) was dissolved in water (4 mL). Propanenitrile (0.5 mL) was added to the solution, before covering the vessel with foil and standing for 6 days. The yellow crystals were filtered, washed with water (2 × 60 mL) and dried under high vacuum. Yield 0.3096 g (66 %).

^{1}H NMR (300 MHz, CD_2Cl_2) δ: 2.83 (dq, *J* = 12.0, 7.5 Hz, 2H), 1.38 (t, *J* = 7.5 Hz, 3H). **LR-MS** (ESI+) $[M+Na]^+$: 398.88 m/z.

[Pt(8-methylureaisoquinoline)$_4$][BF_4]$_2$ (1)

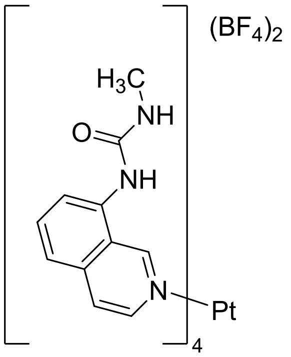

[$PtCl_2(EtCN)_2$] (0.0694 g, 0.1845 mmol) was mixed with **L1** (0.1485 g, 0.7380 mmol) and silver tetrafluoroborate (0.1043 g, 0.5356 mmol). Dry MeCN (15 mL) was degassed with N_2 for 20 min, then added to the mixture and refluxed at 95 °C for 23 h. The mixture was cooled to RT and filtered. The yellow residue was washed with hot MeCN (200 mL) into a separate flask until the filtrate turned colourless. The filtrate was concentrated to give a yellow powder, which was suspended in EtOAc (60 mL) and stirred overnight at 70 °C. The mixture was filtered, and the product obtained as a yellow powder. Yield 0.1332 g (62 %). Melting point: 283.4 – 292.6 °C (decomposed).

^{1}H NMR (400 MHz, DMSO-*d$_6$*) δ: 9.66 (s, 1H), 8.93 (s, 1H), 8.76 (d, *J* = 6.6 Hz, 1H), 8.00 (d, *J* = 6.6 Hz, 1H), 7.87 (t, *J* = 7.9 Hz, 1H), 7.75 (d, *J* = 8.2 Hz, 1H), 7.69 (d, *J* = 7.7 Hz, 1H), 6.33 (s, 1H). **^{13}C NMR** (101 MHz, DMSO-*d$_6$*) δ: 156.54, 154.12, 142.53, 138.09, 136.94, 135.00,

124.99, 124.05, 122.45, 26.74. **^{195}Pt NMR** (86 MHz, DMSO-d_6) δ: −2328. **LR-MS** (ESI+) [M−$(BF_4)_2$]$^{2+}$: 499.65 m/z. **HR-MS** (ESI+) calculated for $C_{44}H_{44}N_{12}O_4Pt$ [M−$(BF_4)_2$]$^{2+}$: 499.66248 m/z, found [M−$(BF_4)_2$]$^{2+}$: 499.66187 m/z.

[Pt(8-ethylureaisoquinoline)$_4$][BF$_4$]$_2$ (2)

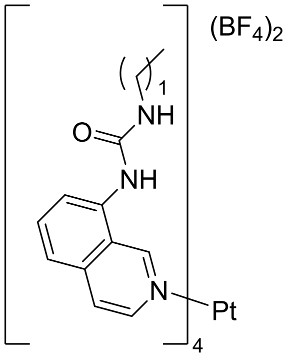

[PtCl$_2$(EtCN)$_2$] (0.0700 g, 0.1861 mmol) was mixed with **L2** (0.1687 g, 0.7837 mmol) and silver tetrafluoroborate (0.1161 g, 0.5964 mmol). Dry MeCN (15 mL) was degassed with N_2 for 20 min, then added to the mixture and refluxed at 95 °C for 22 h. The mixture was cooled to RT and filtered. The residue was washed with hot MeCN (200 mL) into a separate flask until the filtrate turned colourless. The filtrate was concentrated to give a powder, which was suspended in EtOAc (60 mL) and stirred overnight at 70 °C. The mixture was filtered, and the product obtained as a pale powder. Yield 0.1224 g (53 %). Melting point: 282.2 – 294.5 °C (decomposed).

^{1}H NMR (400 MHz, DMSO-d_6) δ: 9.59 (s, 1H), 8.82 (s, 1H), 8.68 (d, *J* = 6.6 Hz, 1H), 7.97 (d, *J* = 6.6 Hz, 1H), 7.87 (t, *J* = 7.9 Hz, 1H), 7.73 (t, *J* = 8.1 Hz, 2H), 6.38 (t, *J* = 5.7 Hz, 1H), 2.87 (p, *J* = 6.9 Hz, 2H), 0.97 (t, *J* = 7.2 Hz, 3H). **^{13}C NMR** (101 MHz, DMSO-d_6) δ: 155.69, 154.18, 142.49, 138.14, 136.97, 135.00, 125.01, 123.81, 122.23, 122.01, 34.59, 15.74. **^{195}Pt NMR** (86 MHz, DMSO-d_6) δ: −2315. **LR-MS** (ESI+) [M−$(BF_4)_2$]$^{2+}$: 528.16. **HR-MS** (ESI+) calculated for $C_{48}H_{52}N_{12}O_4Pt$ [M−$(BF_4)_2$]$^{2+}$: 527.69379 m/z, found [M−$(BF_4)_2$]$^{2+}$: 527.69324 m/z.

[Pt(8-propylureaisoquinoline)$_4$][BF$_4$]$_2$ (3)

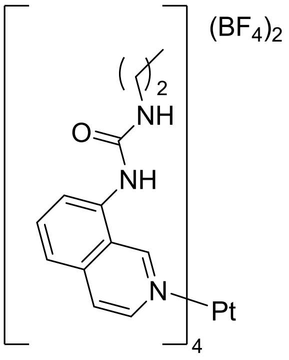

[PtCl$_2$(EtCN)$_2$] (0.0701 g, 0.1864 mmol) was mixed with **L3** (0.1707 g, 0.7444 mmol) and silver tetrafluoroborate (0.1087 g, 0.5583 mmol). Dry MeCN (15 mL) was degassed with N_2 for 20 min, then added to the mixture and refluxed at 95 °C for 23 h. The mixture was cooled to RT and filtered. The residue was washed with hot MeCN (200 mL) into a separate flask until the filtrate turned colourless. The filtrate was concentrated to give the product as a pale-yellow powder, which turned yellow-green after drying. Yield 0.1333 g (56 %). Melting point: 251.5 – 263.6 °C (decomposed).

^{1}H NMR (400 MHz, DMSO-*d$_6$*) δ: 9.54 (s, 1H), 8.80 (s, 1H), 8.62 (d, *J* = 6.6 Hz, 1H), 7.97 (d, *J* = 6.6 Hz, 1H), 7.87 (t, *J* = 7.9 Hz, 1H), 7.80 – 7.67 (m, 2H), 6.38 (t, *J* = 5.8 Hz, 1H), 2.75 (q, *J* = 6.7 Hz, 2H), 1.33 (h, *J* = 7.3 Hz, 2H), 0.81 (t, *J* = 7.4 Hz, 3H). **^{13}C NMR** (101 MHz, DMSO-*d$_6$*) δ: 155.78, 154.23, 142.49, 138.16, 137.00, 135.04, 125.04, 123.80, 122.25, 121.94, 41.39, 23.29, 11.77. **^{195}Pt NMR** (86 MHz, DMSO-d$_6$) δ: −2307. **LR-MS** (ESI+) $[M-(BF_4)_2]^{2+}$: 555.70 m/z. **HR-MS** (ESI+) calculated for $C_{52}H_{60}N_{12}O_4Pt$ $[M-(BF_4)_2]^{2+}$: 555.72503 m/z, found $[M-(BF_4)_2]^{2+}$: 555.72485 m/z.

[Pt(8-butylureaisoquinoline)$_4$][BF$_4$]$_2$ (4)

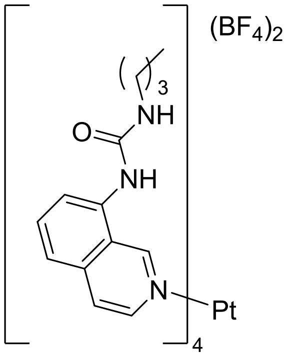

$[PtCl_2(EtCN)_2]$ (0.0655 g, 0.1741 mmol) was mixed with **L4** (0.1694 g, 0.6962 mmol) and silver tetrafluoroborate (0.0748 g, 0.3842 mmol). Dry MeCN (15 mL) was degassed with N_2 for 20 min, then added to the mixture and refluxed at 95 °C for 24 h. The mixture was transferred to a centrifuge tube and centrifuged at 1700 RCF for 5 min. The supernatant was transferred to a flask and a yellow solid precipitated overnight. The solid was filtered and dried to give the product. Yield 0.0542 g (23 %).

^{1}H NMR (300 MHz, DMSO-*d_6*) δ: 9.54 (s, 1H), 8.80 (s, 1H), 8.62 (d, *J* = 6.6 Hz, 1H), 7.97 (d, *J* = 6.6 Hz, 1H), 7.92 – 7.82 (m, 1H), 7.78 – 7.68 (m, 2H), 6.35 (s, 1H), 2.79 (q, *J* = 6.3 Hz, 2H), 1.37 – 1.14 (m, 4H), 0.86 (t, *J* = 7.0 Hz, 3H). **LR-MS** (ESI+) $[M-(BF_4)]^+$: 1254.67 m/z.

$[Pt(8\text{-pentylureaisoquinoline})_4][BF_4]_2$ (5)

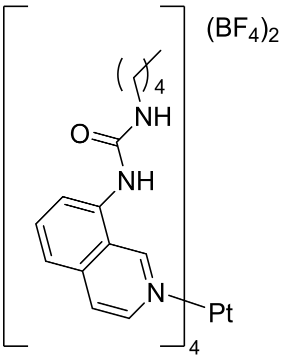

$[PtCl_2(EtCN)_2]$ (0.0700 g, 0.1861 mmol) was mixed with **L5** (0.1916 g, 0.7444 mmol) and silver tetrafluoroborate (0.1068 g, 0.5486 mmol). Dry MeCN (15 mL) was degassed with N_2 for 20 min, then added to the mixture and refluxed at 95 °C for 25 h. The mixture was cooled to RT and filtered. The residue was washed with hot MeCN (200 mL) into a separate flask until the filtrate turned colourless. The filtrate was concentrated to give the product as a yellow powder, which turned yellow-green after drying. Yield 0.1784 g (69 %). Melting point: 247.8 – 256.5 °C (decomposed).

^{1}H NMR (400 MHz, DMSO-*d_6*) δ: 9.56 (s, 1H), 8.80 (s, 1H), 8.64 (d, *J* = 6.6 Hz, 1H), 7.97 (d, *J* = 6.6 Hz, 1H), 7.87 (t, *J* = 7.9 Hz, 1H), 7.73 (d, *J* = 8.0 Hz, 2H), 6.36 (s, 1H), 2.79 (q, *J* = 6.6 Hz, 2H), 1.39 – 1.13 (m, 6H), 0.88 (t, *J* = 7.1 Hz, 3H). **^{13}C NMR** (101 MHz, DMSO-*d_6*) δ: 155.75, 154.19, 142.48, 138.18, 136.98, 135.05, 125.02, 123.74, 122.18, 121.84, 29.73, 28.99, 22.31, 14.39. **^{195}Pt NMR** (86 MHz, DMSO-*d_6*) δ: −2313. **LR-MS** (ESI+) $[M-(BF_4)_2]^{2+}$: 612.29 m/z. **HR-MS** (ESI+) calculated for $C_{60}H_{76}N_{12}O_4Pt$ $[M-(BF_4)_2]^{2+}$: 612.28841 m/z, found $[M-(BF_4)_2]^{2+}$: 611.78752 m/z.

[Pt(8-hexylureaisoquinoline)$_4$][BF$_4$]$_2$ (6)

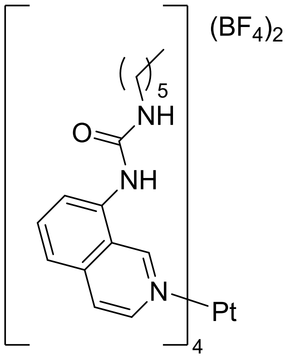

[PtCl$_2$(EtCN)$_2$] (0.0700 g, 0.1861 mmol) was mixed with **L6** (0.2019 g, 0.7444 mmol) and silver tetrafluoroborate (0.0878 g, 0.451 mmol). Dry MeCN (15 mL) was degassed with N_2 for 20 min, then added to the mixture and refluxed at 95 °C for 22 h. The mixture was cooled to RT and filtered. The residue was washed with hot MeCN (200 mL) into a separate flask until the filtrate turned colourless. The filtrate was concentrated to give the product as a yellow powder. Yield 0.1120 g (44 %). Melting point: 250.3 – 258.7 °C (decomposed).

^{1}H NMR (400 MHz, DMSO-*d$_6$*) δ: 9.58 (s, 1H), 8.81 (s, 1H), 8.65 (d, *J* = 6.6 Hz, 1H), 7.96 (d, *J* = 6.7 Hz, 1H), 7.87 (t, *J* = 7.9 Hz, 1H), 7.73 (t, *J* = 7.8 Hz, 2H), 6.38 (s, 1H), 2.80 (q, *J* = 6.6 Hz, 2H), 1.38 – 1.16 (m, 8H), 0.88 (t, *J* = 6.9 Hz, 3H). **^{13}C NMR** (101 MHz, DMSO-*d$_6$*) δ: 155.73, 154.11, 142.47, 138.17, 136.95, 135.05, 125.01, 123.68, 122.11, 121.76, 31.48, 30.04, 26.50, 22.55, 14.39. **^{195}Pt NMR** (86 MHz, DMSO-*d$_6$*) δ: −2311. **LR-MS** (ESI+) [M−(BF$_4$)$_2$]$^{2+}$: 640.36 m/z. **HR-MS** (ESI+) calculated for $C_{64}H_{84}N_{12}O_4Pt$ [M−(BF$_4$)$_2$]$^{2+}$: 639.81897 m/z, found [M−(BF$_4$)$_2$]$^{2+}$: 639.81878 m/z.

[Pt(8-octylureaisoquinoline)$_4$][BF$_4$]$_2$ (7)

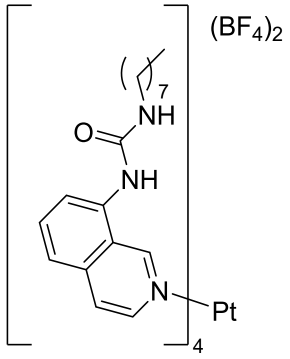

[PtCl$_2$(EtCN)$_2$] (0.0699 g, 0.1858 mmol) was mixed with **L7** (0.2225 g, 0.7431 mmol) and silver tetrafluoroborate (0.1302 g, 0.6688 mmol). Dry MeCN (15 mL) was degassed with N_2

for 20 min, then added to the mixture and refluxed at 95 °C for 24 h. The mixture was cooled to RT and filtered. The residue was washed with hot MeCN (200 mL) into a separate flask until the filtrate turned colourless. The filtrate was concentrated to give the product as a pale-yellow powder. Yield 0.1400 g (48 %). Melting point: 210.5 – 233.4 °C (decomposed).

^{1}H NMR (400 MHz, DMSO-*d_6*) δ: 9.58 (s, 1H), 8.81 (s, 1H), 8.66 (d, *J* = 6.6 Hz, 1H), 7.97 (d, *J* = 6.6 Hz, 1H), 7.87 (t, *J* = 7.9 Hz, 1H), 7.74 (d, *J* = 5.1 Hz, 2H), 6.36 (s, 1H), 2.81 (q, *J* = 6.7 Hz, 2H), 1.39 – 1.15 (m, 12H), 0.86 (t, *J* = 6.6 Hz, 3H). **^{13}C NMR** (101 MHz, DMSO-*d_6*) δ: 155.75, 154.13, 142.49, 138.17, 136.97, 135.04, 125.02, 123.73, 122.16, 121.83, 31.75, 30.10, 29.24, 29.19, 26.85, 22.58, 14.41. **^{195}Pt NMR** (86 MHz, DMSO-d_6) δ: −2315. **LR-MS** (ESI+) $[M-(BF_4)_2]^{2+}$: 695.93 m/z. **HR-MS** (ESI+) calculated for $C_{72}H_{100}N_{12}O_4Pt$ $[M-(BF_4)_2]^{2+}$: 696.38242 m/z, found $[M-(BF_4)_2]^{2+}$: 695.88170 m/z.

[Pt(8-decylureaisoquinoline)$_4$][BF$_4$]$_2$ (8)

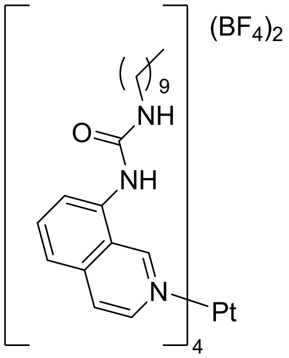

$[PtCl_2(EtCN)_2]$ (0.0703 g, 0.1869 mmol) was mixed with **L8** (0.2637 g, 0.8053 mmol) and silver tetrafluoroborate (0.2901 g, 1.490 mmol). Dry MeCN (15 mL) was degassed with N_2 for 20 min, then added to the mixture and refluxed at 85 °C for 24 h. The mixture was cooled to RT, transferred, then centrifuged at 1700 RCF for 5 min. The supernatant was decanted and the pellet resuspended in acetonitrile (5 mL) and filtered. The solid was washed with cold diethyl ether (30 mL) followed by hot MeCN (200 mL) until the filtrate turned colourless. The filtrate was concentrated to give the product as yellow-brown needles. Yield 0.1187 g (38 %). Melting point: 206.5 – 214.2 °C (decomposed). **^{1}H NMR** (400 MHz, DMSO-*d_6*) δ: 9.59 (s, 1H), 8.81 (s, 1H), 8.67 (d, *J* = 6.6 Hz, 1H), 7.97 (d, *J* = 6.6 Hz, 1H), 7.86 (t, *J* = 7.9 Hz, 1H), 7.73 (t, *J* = 9.0 Hz, 2H), 6.37 (s, 1H), 2.82 (q, *J* = 6.6 Hz, 2H), 1.38 – 1.16 (m, 16H), 0.85 (t, *J* = 6.6 Hz, 3H). **^{13}C NMR** (101 MHz, DMSO-*d_6*) δ: 155.25, 153.54, 141.98, 137.65, 136.44, 134.54, 124.51, 123.16, 121.60, 121.26, 31.29, 29.59, 29.05, 29.00, 28.79, 28.71, 26.35, 22.07, 13.89. **^{195}Pt**

NMR (86 MHz, DMSO-d_6) δ: −2318. **LR-MS** (ESI+) $[M-(BF_4)_2]^{2+}$: 752.02 m/z. **HR-MS** (ESI+) calculated for $C_{80}H_{116}N_{12}O_4Pt$ $[M-(BF_4)_2]^{2+}$: 752.44509 m/z, found $[M-(BF_4)_2]^{2+}$: 751.94413 m/z.

Characterisation spectra

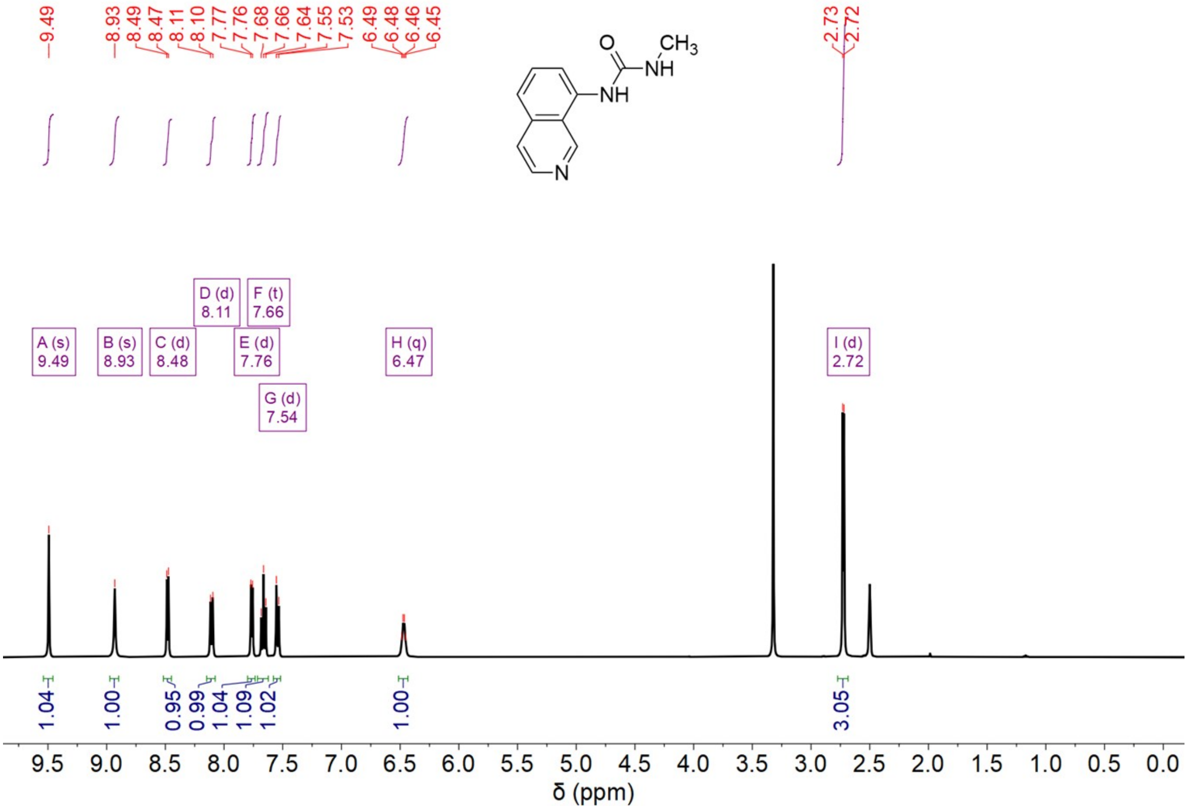

Figure S1. ^1H NMR (400 MHz, DMSO-d_6) spectrum of ligand **L1**.

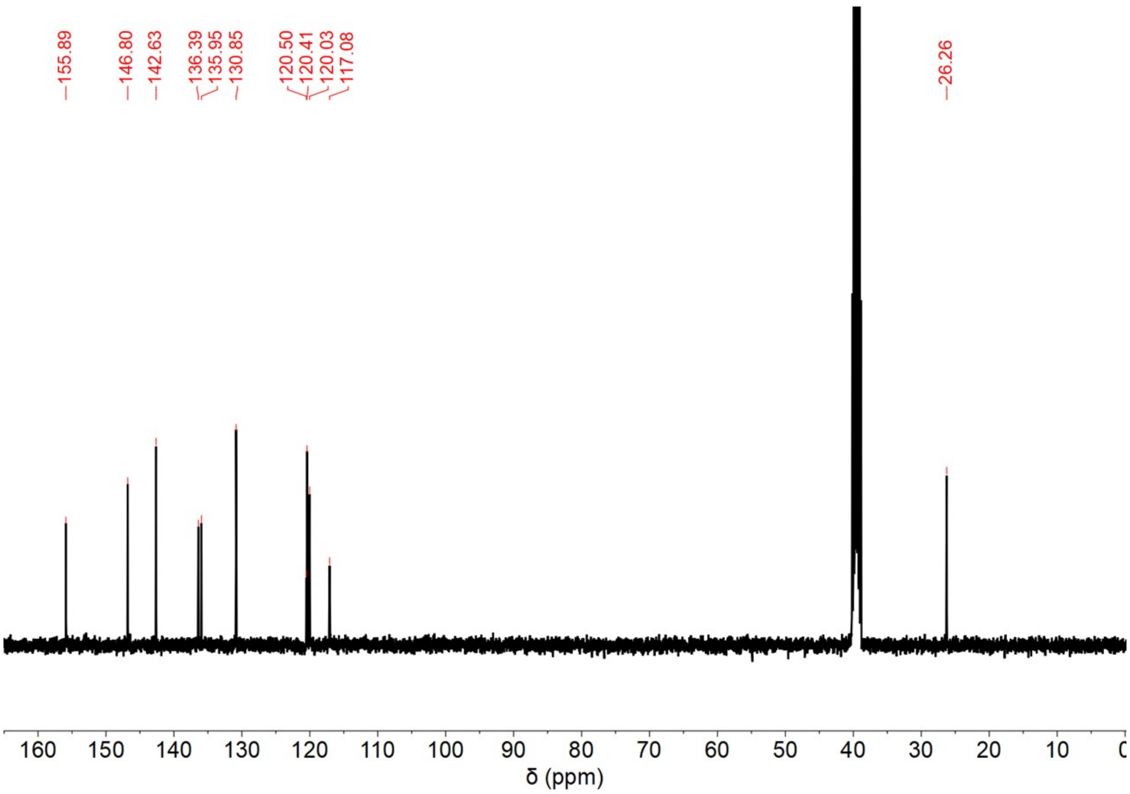

Figure S2. ^{13}C NMR (101 MHz, DMSO-d_6) spectrum of ligand **L1**.

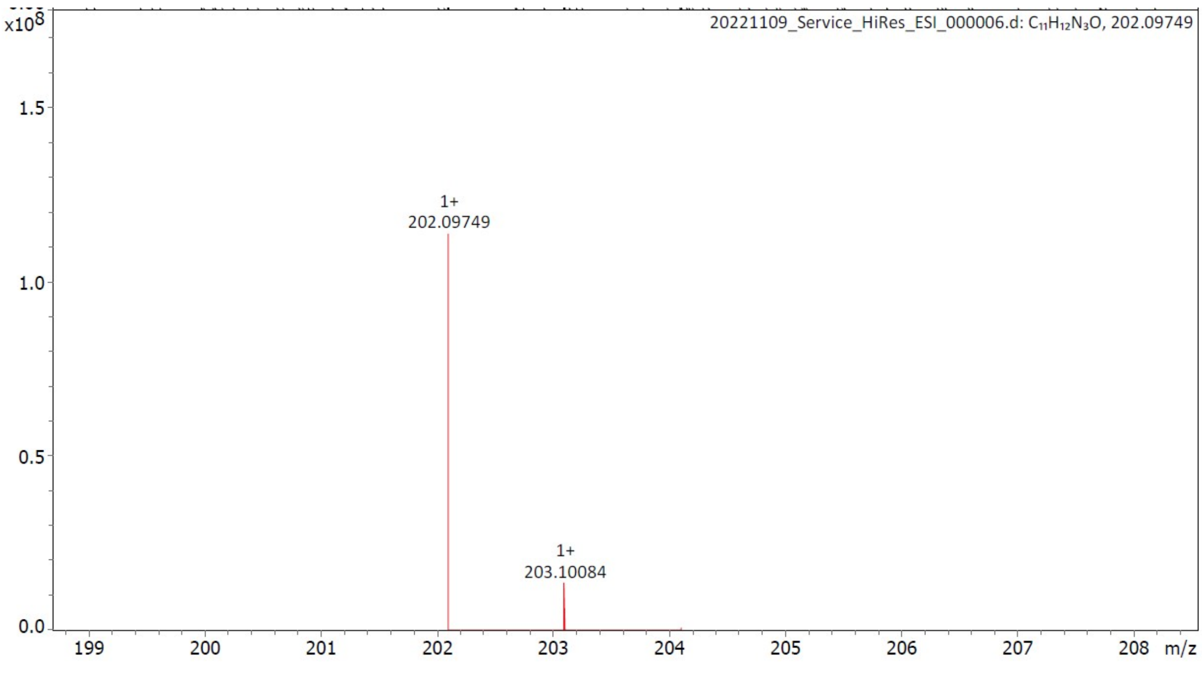

Figure S3. HR-MS (ESI+) of ligand **L1**.

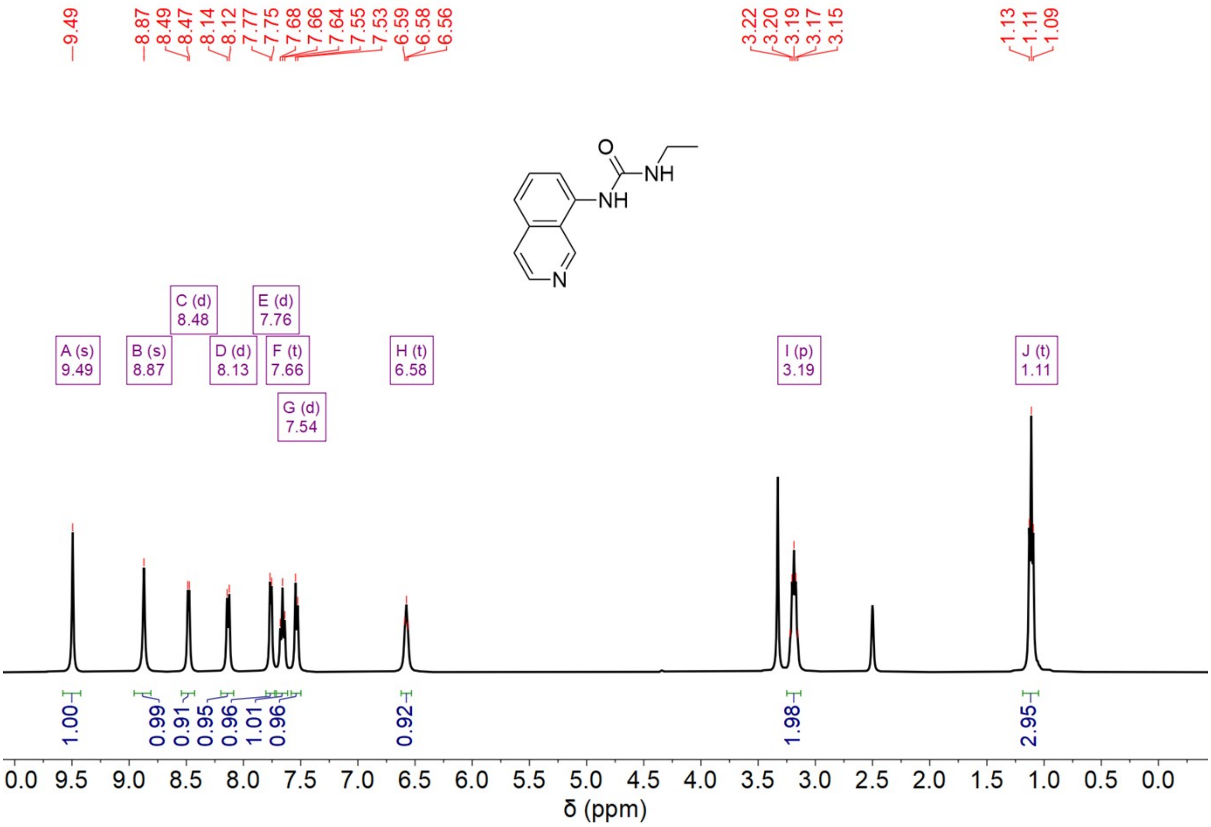

Figure S4. ^{1}H NMR (400 MHz, DMSO-d_6) spectrum of ligand **L2**.

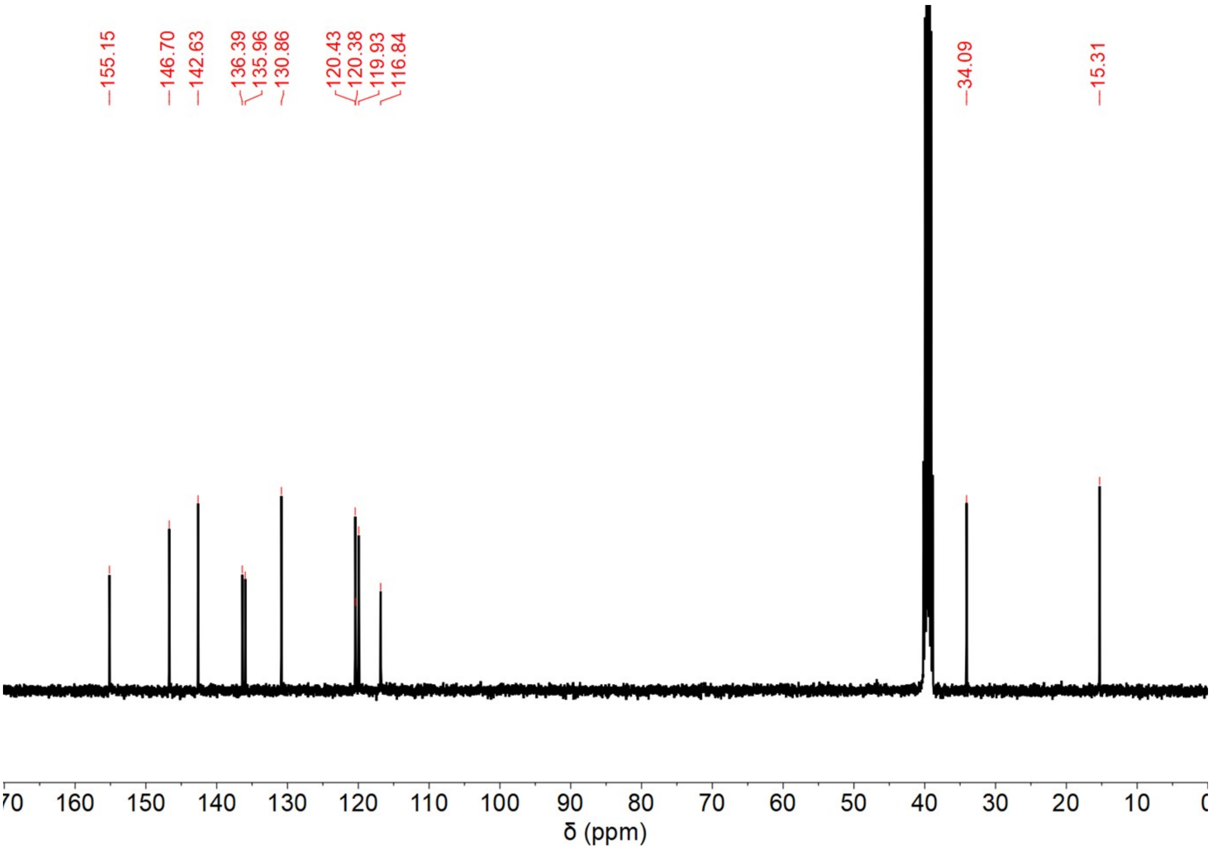

Figure S5. ^{13}C NMR (101 MHz, DMSO-d_6) spectrum of ligand **L2**.

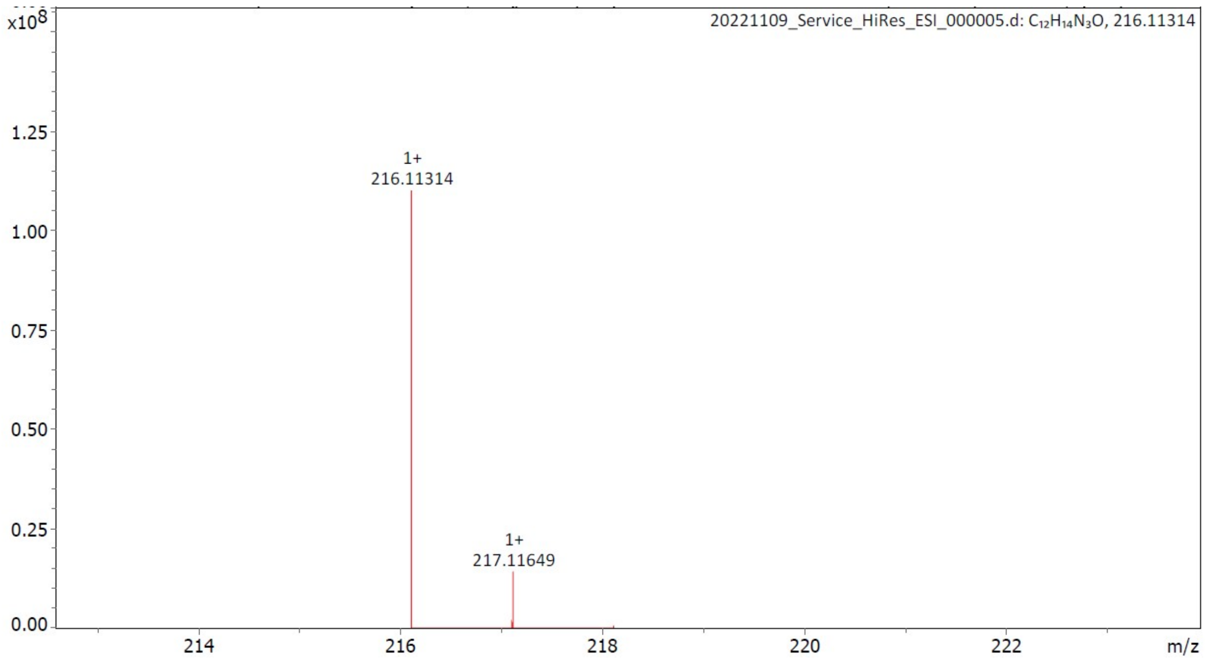

Figure S6. HR-MS (ESI+) of ligand **L2**.

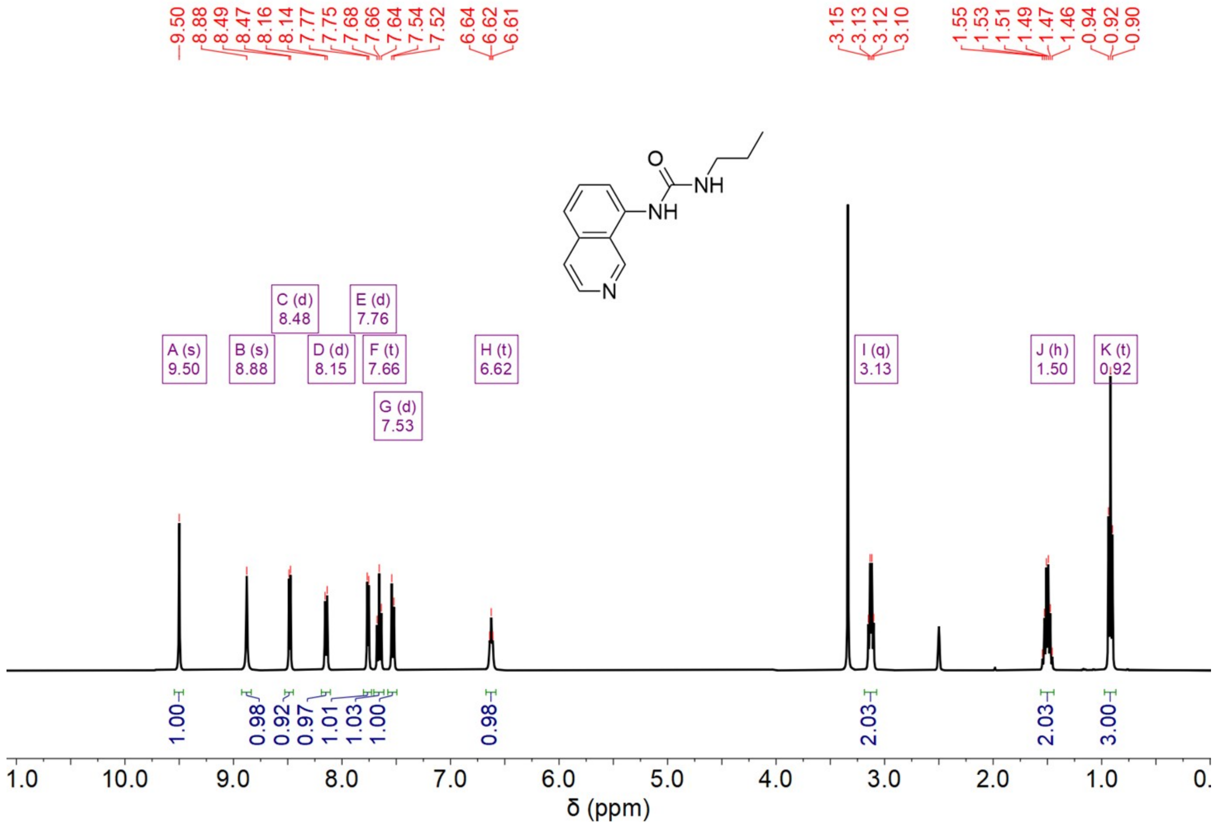

Figure S7. ^{1}H NMR (400 MHz, DMSO-d_6) spectrum of ligand **L3**.

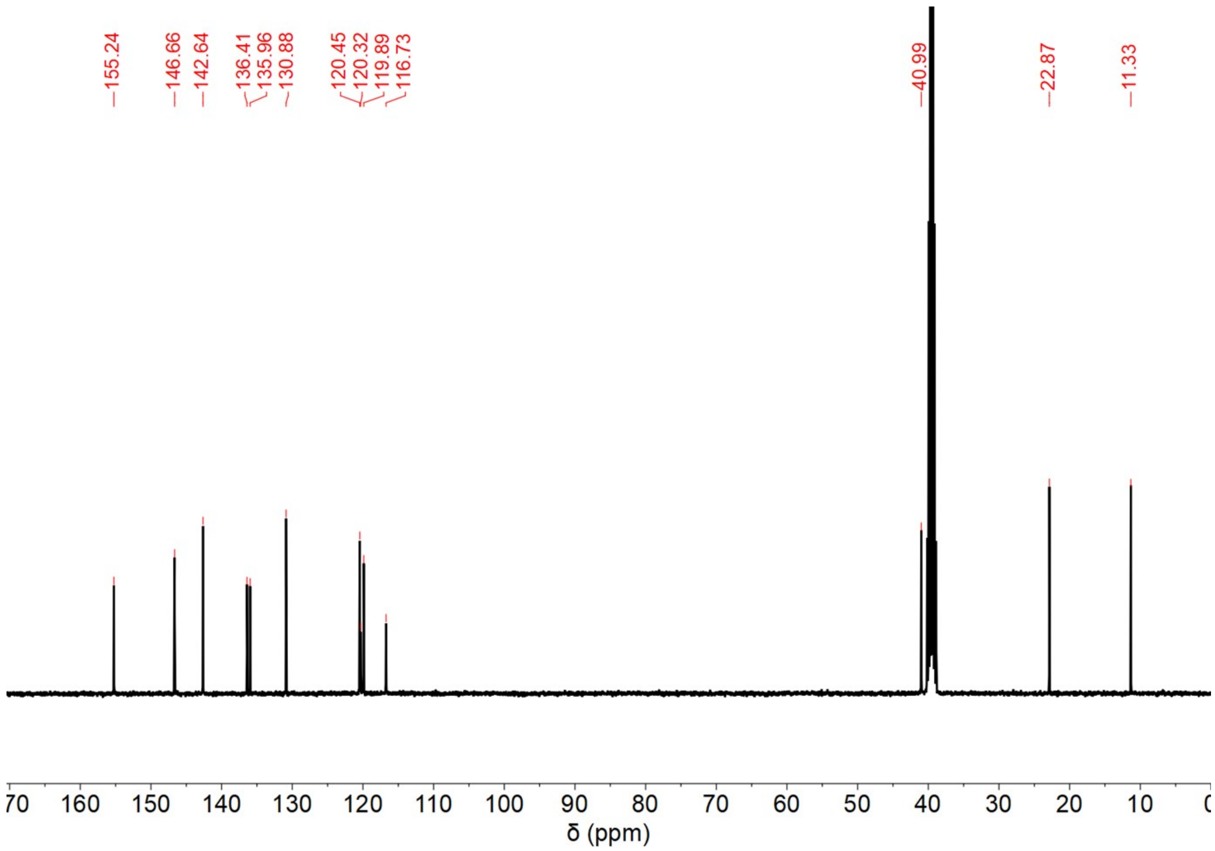

Figure S8. ^{13}C NMR (101 MHz, DMSO-d_6) spectrum of ligand **L3**.

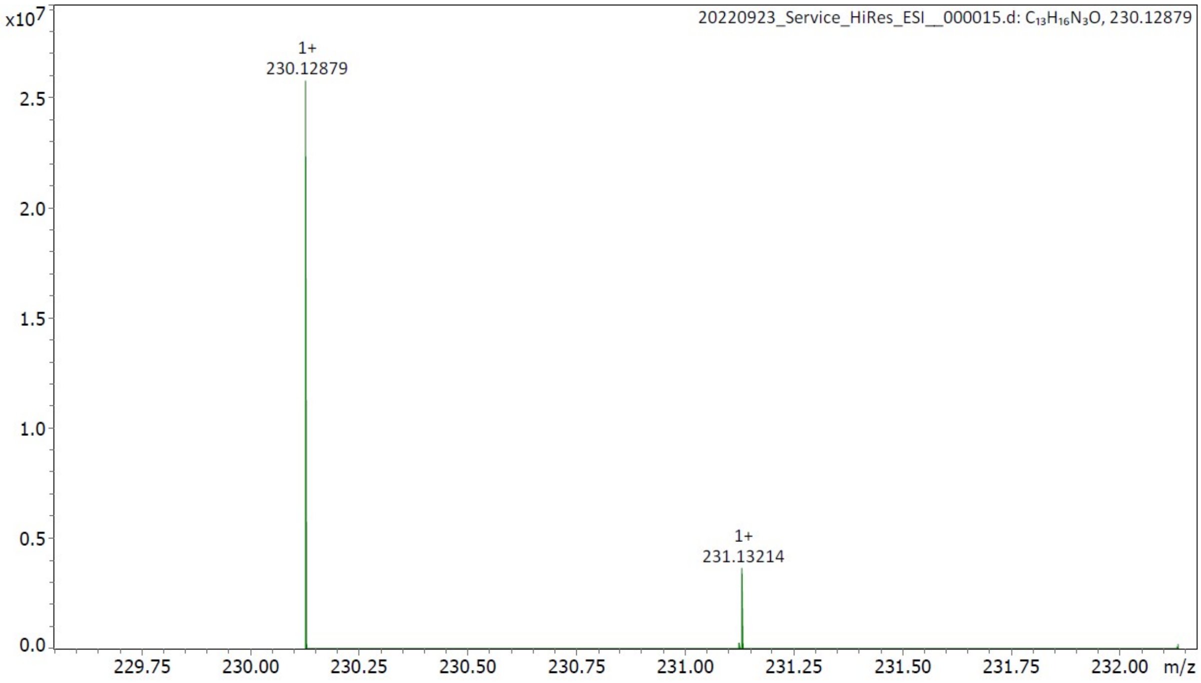

Figure S9. HR-MS (ESI+) of ligand **L3**.

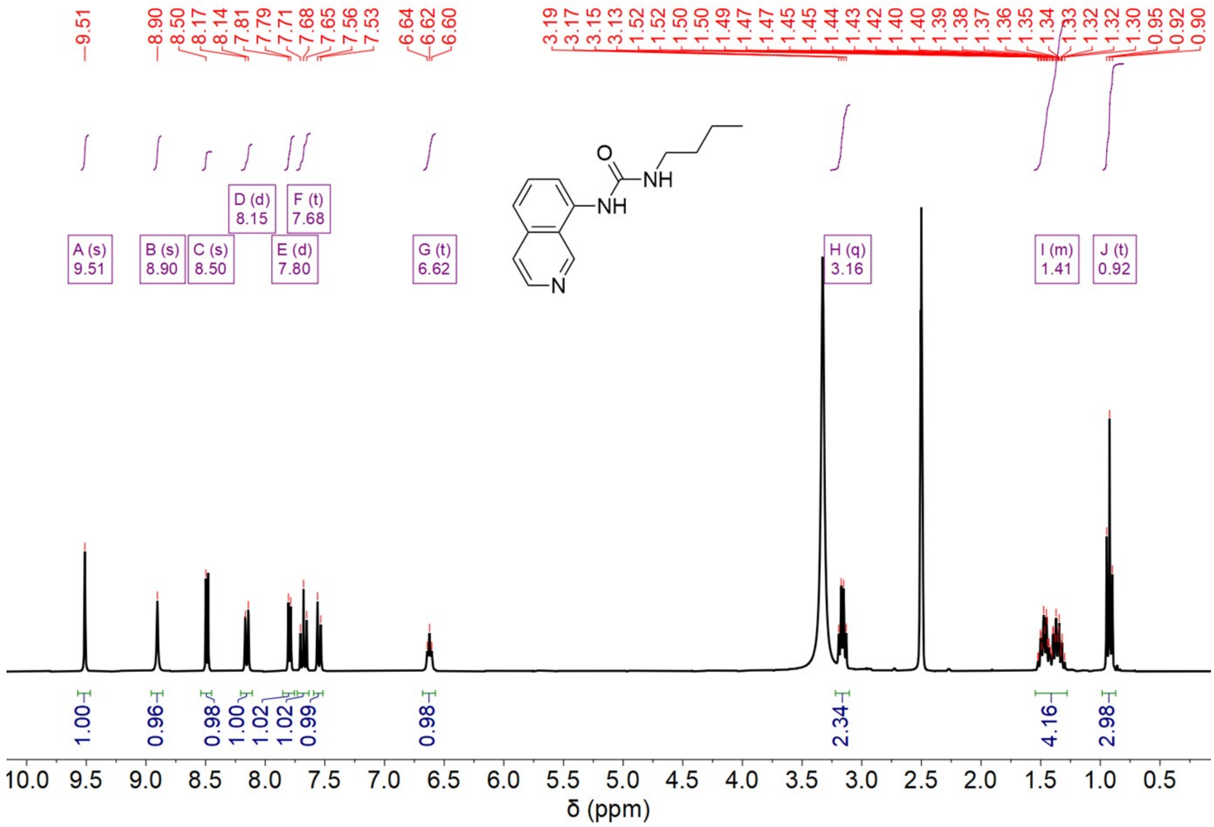

Figure S10. ^{1}H NMR (300 MHz, DMSO-d_6) spectrum of ligand **L4**.

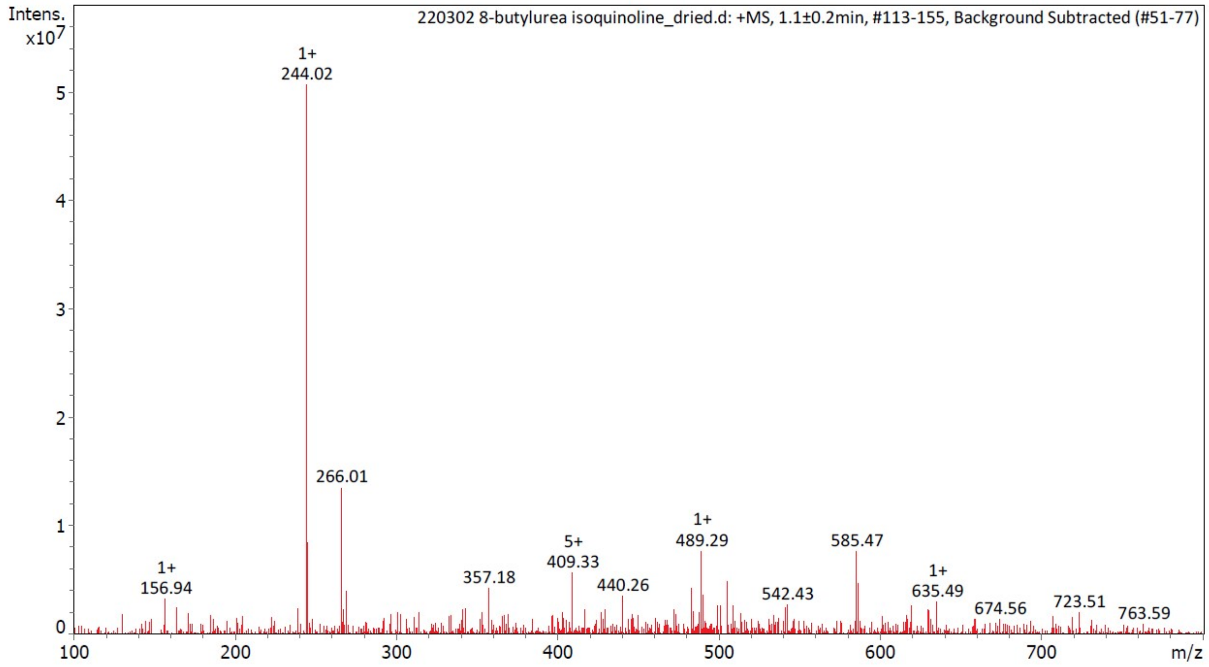

Figure S11. LR-MS (ESI+) of ligand **L4**.

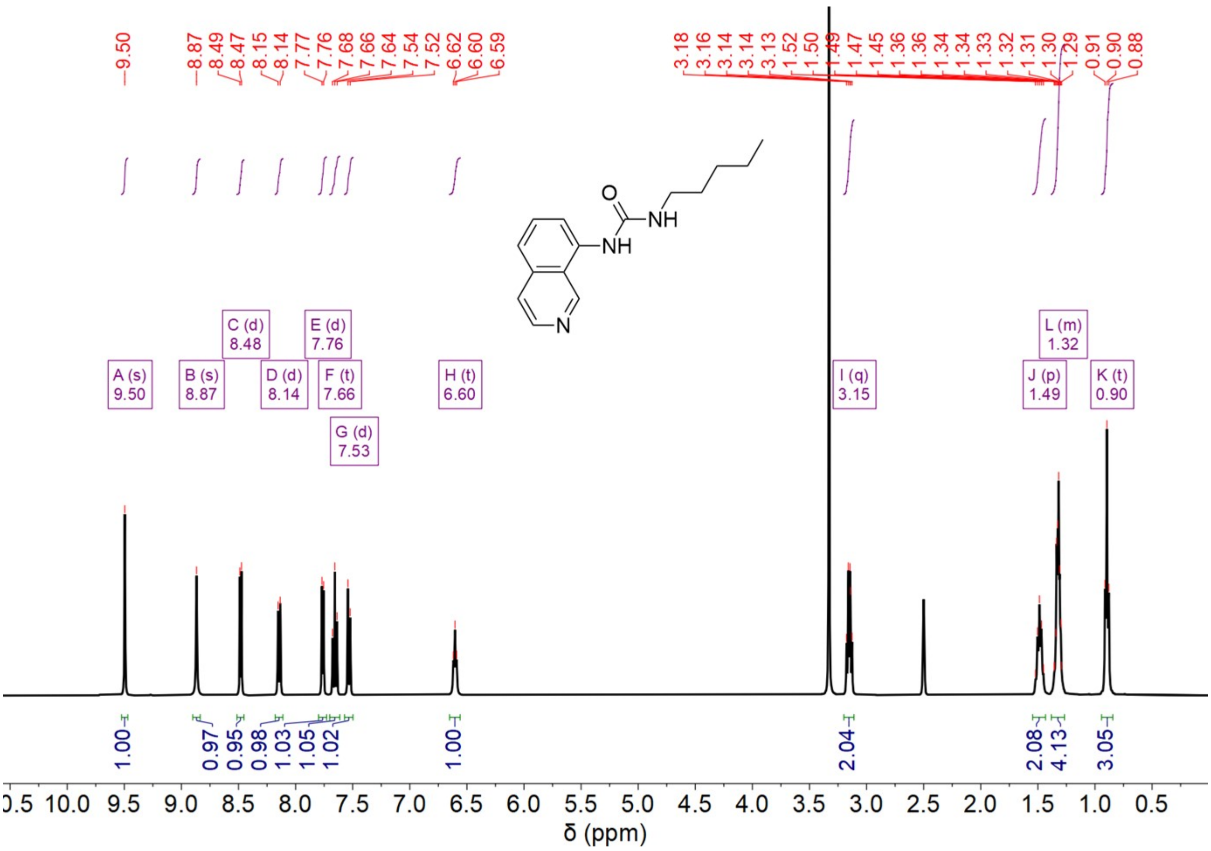

Figure S12. ^{1}H NMR (400 MHz, DMSO-d_6) spectrum of ligand **L5**.

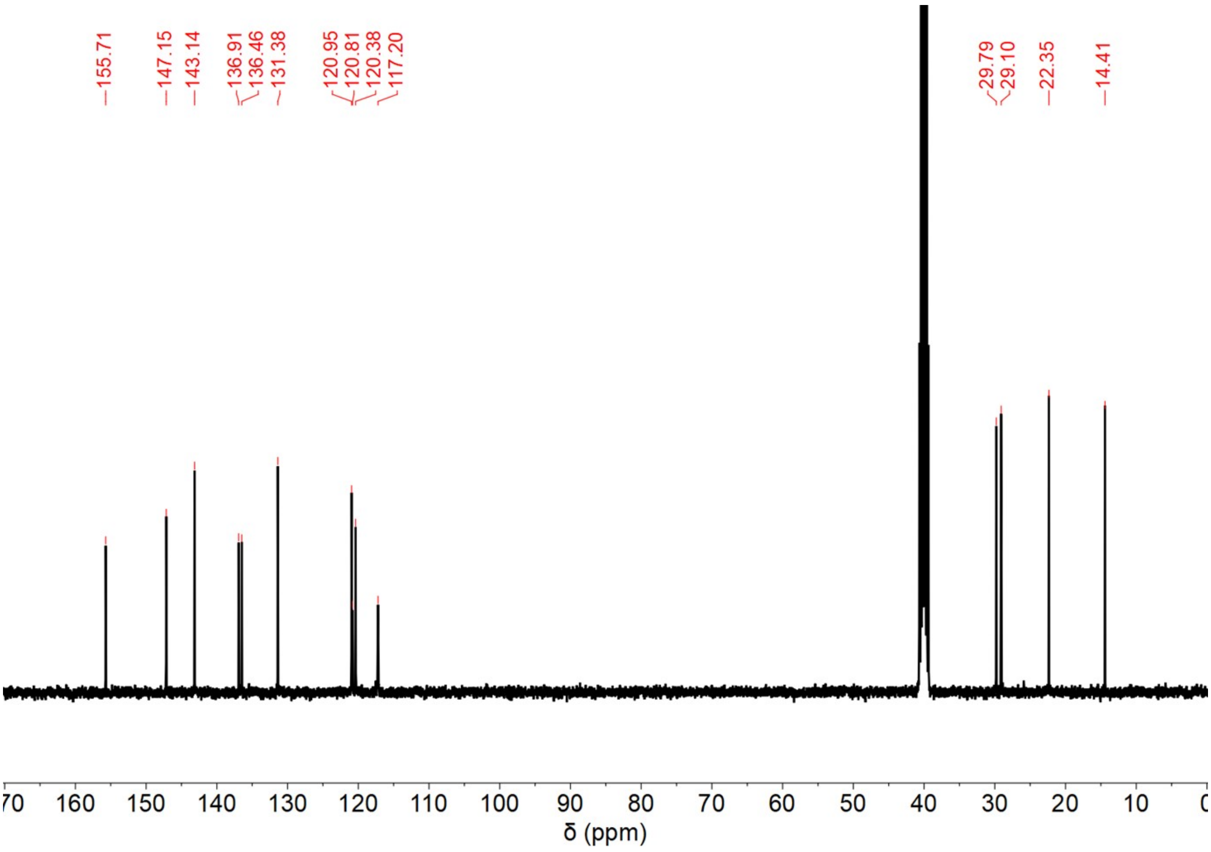

Figure S13. ^{13}C NMR (101 MHz, DMSO-d_6) spectrum of ligand **L5**.

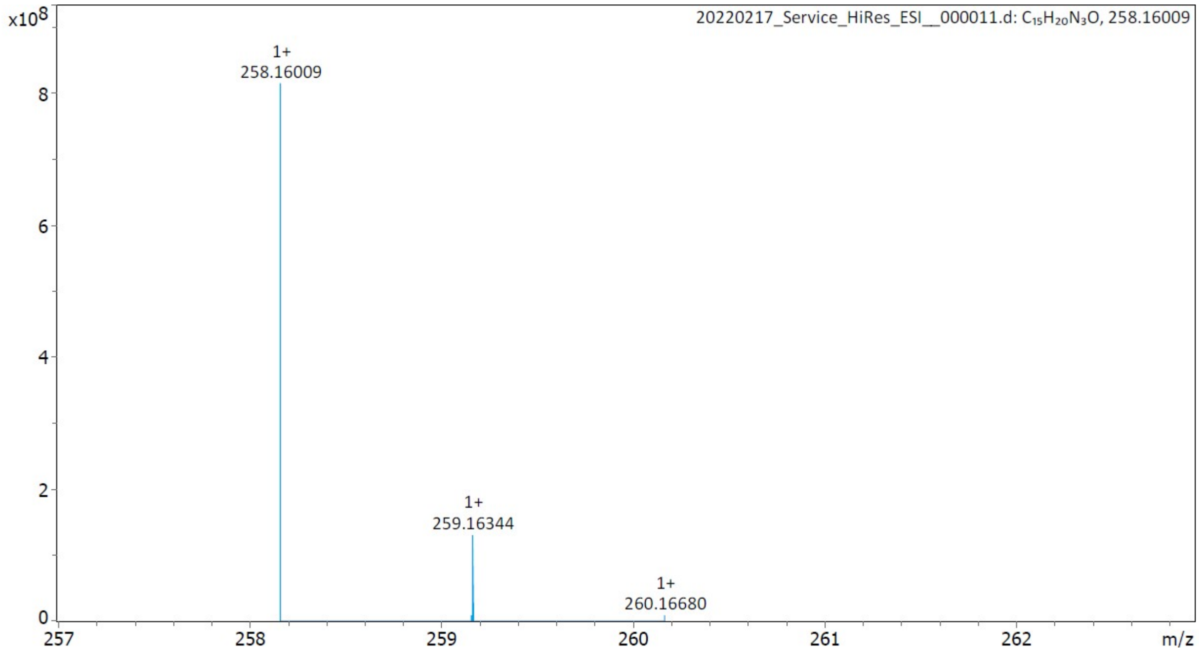

Figure S14. HR-MS (ESI+) of ligand **L5**.

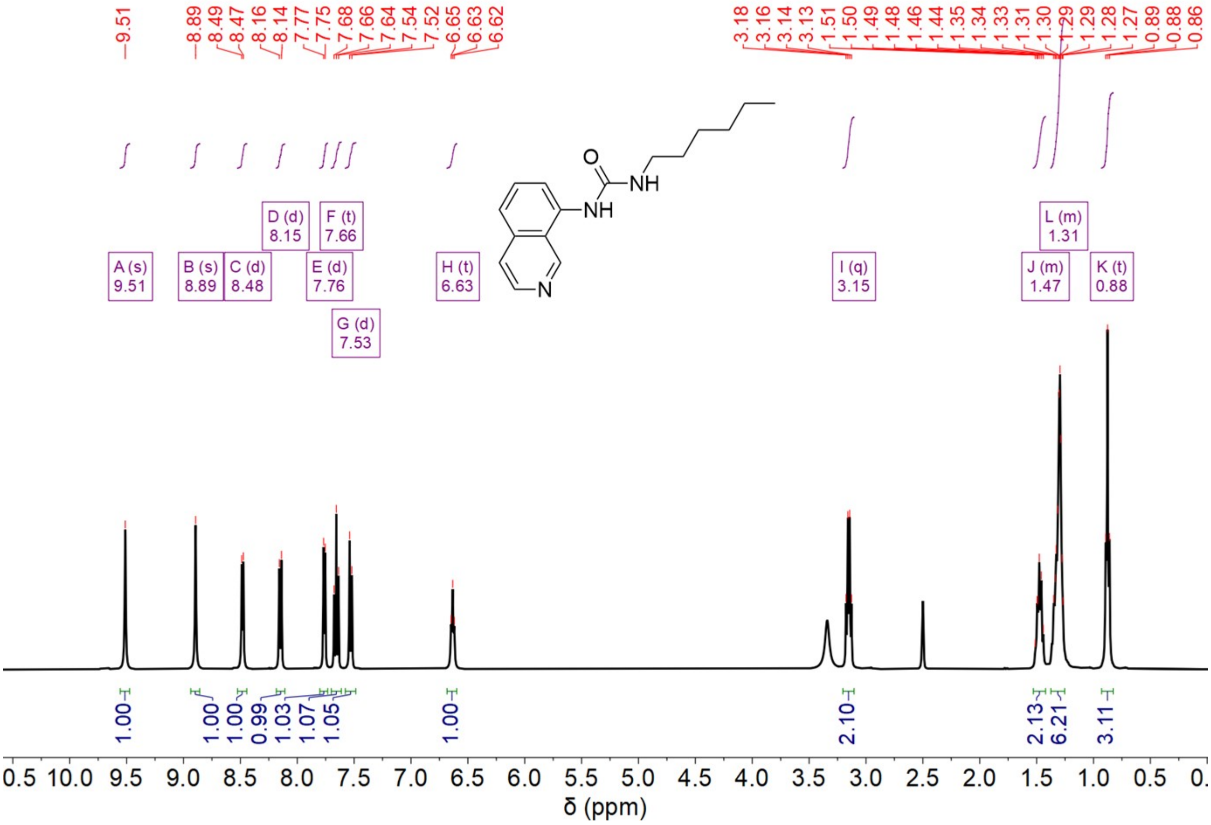

Figure S15. ^{1}H NMR (400 MHz, DMSO-d_6) spectrum of ligand **L6**.

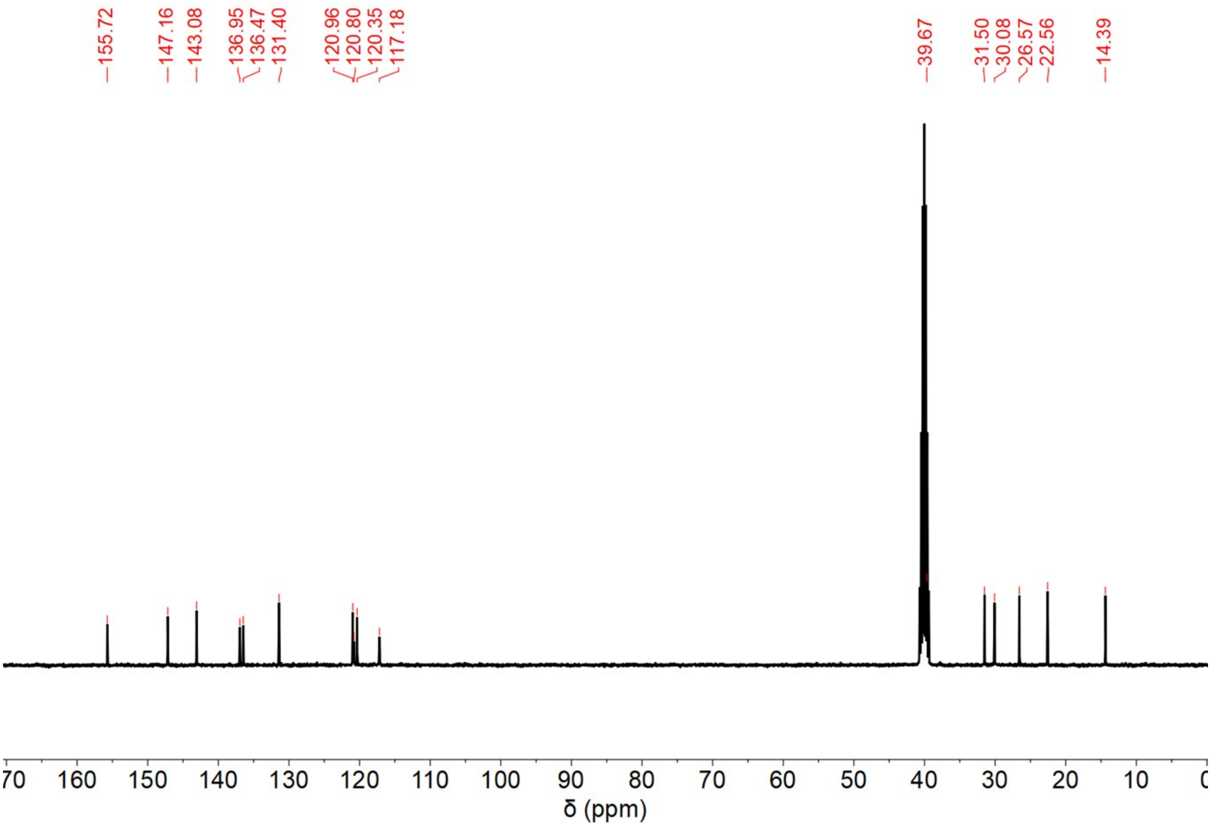

Figure S16. ^{13}C NMR (101 MHz, DMSO-d_6) spectrum of ligand **L6**.

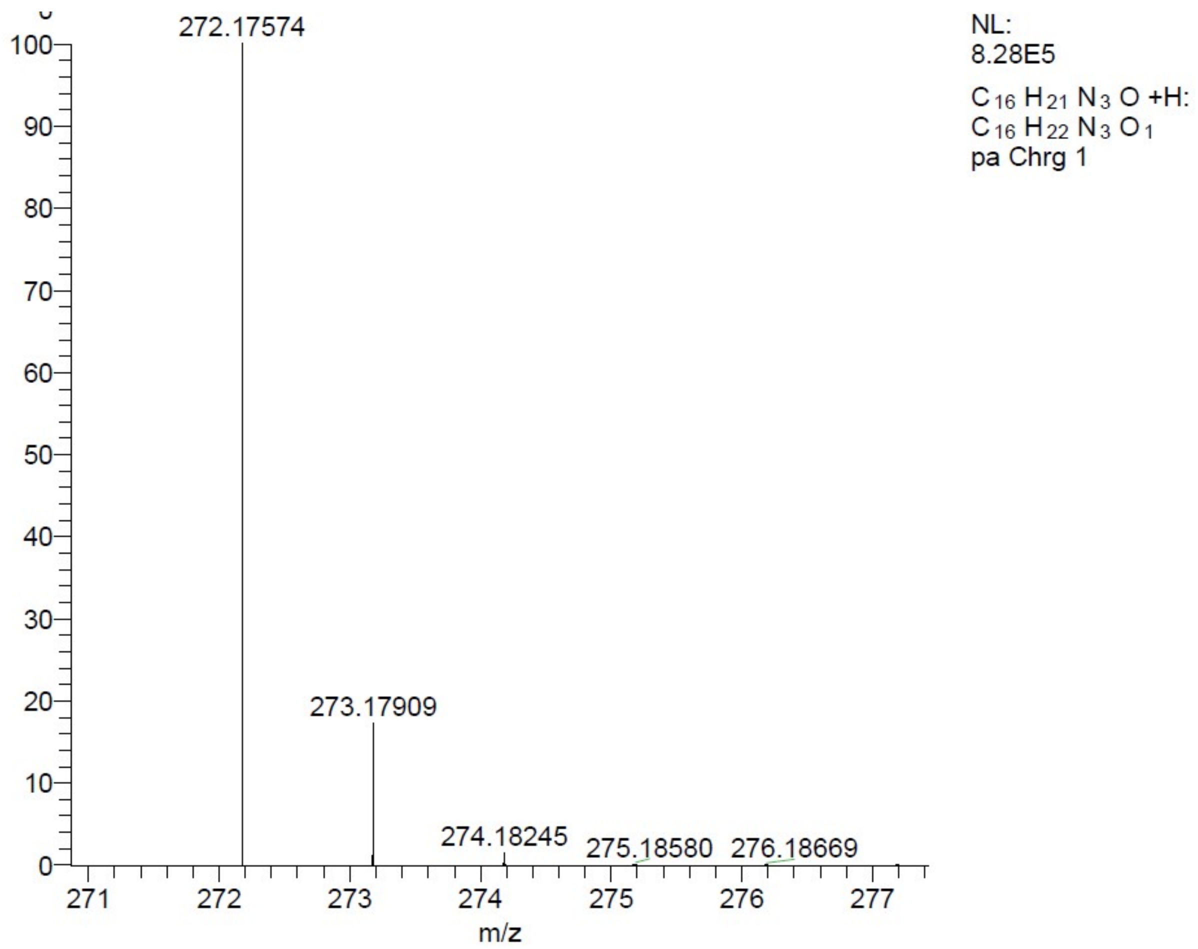

Figure S17. HR-MS (ESI+) of ligand **L6**.

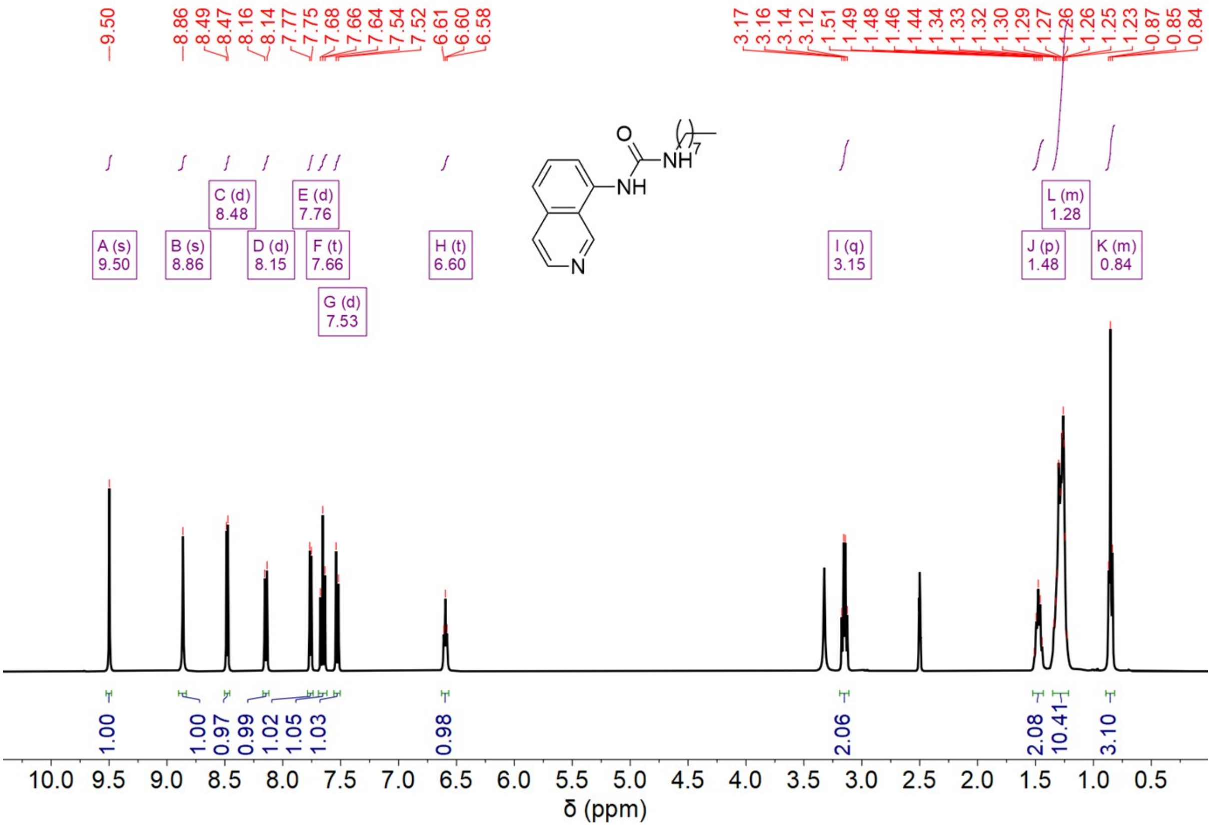

Figure S18. ^{1}H NMR (400 MHz, DMSO-d_6) spectrum of ligand **L7**.

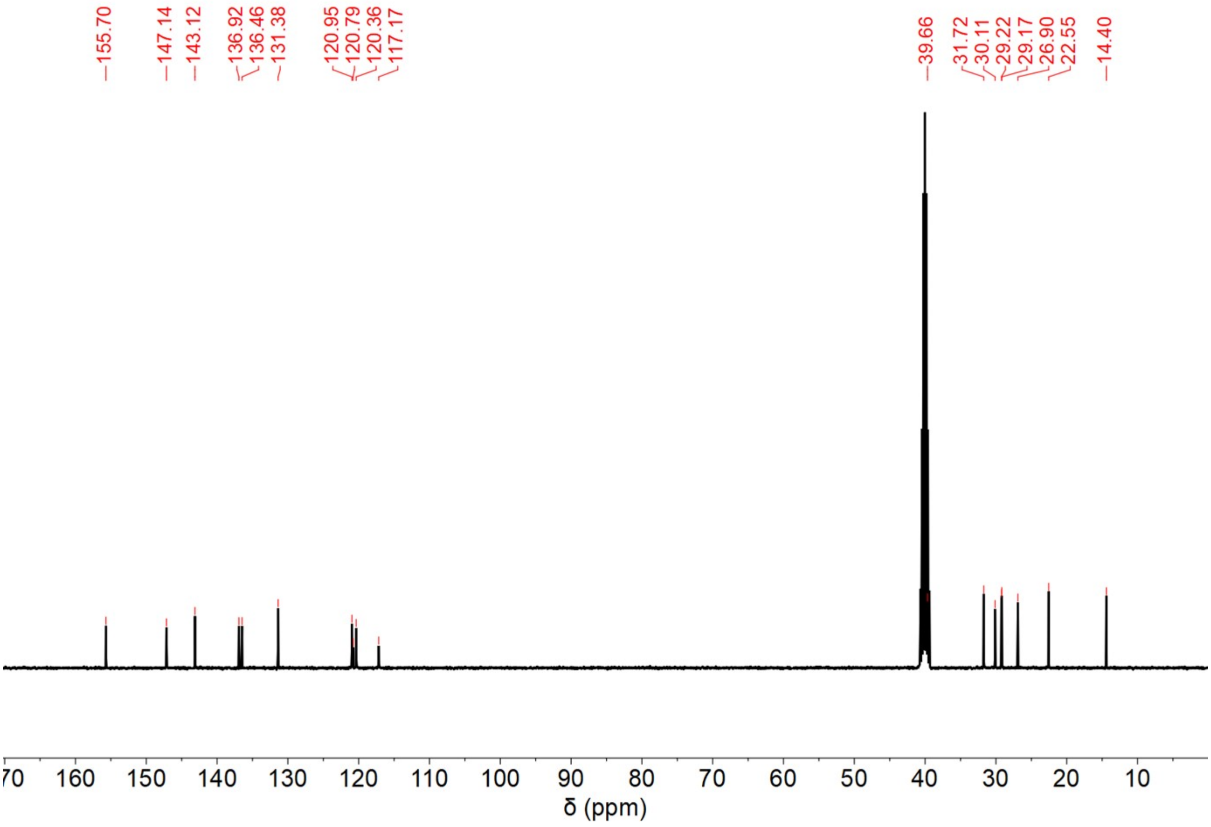

Figure S19. ^{13}C NMR (101 MHz, DMSO-d_6) spectrum of ligand **L7**.

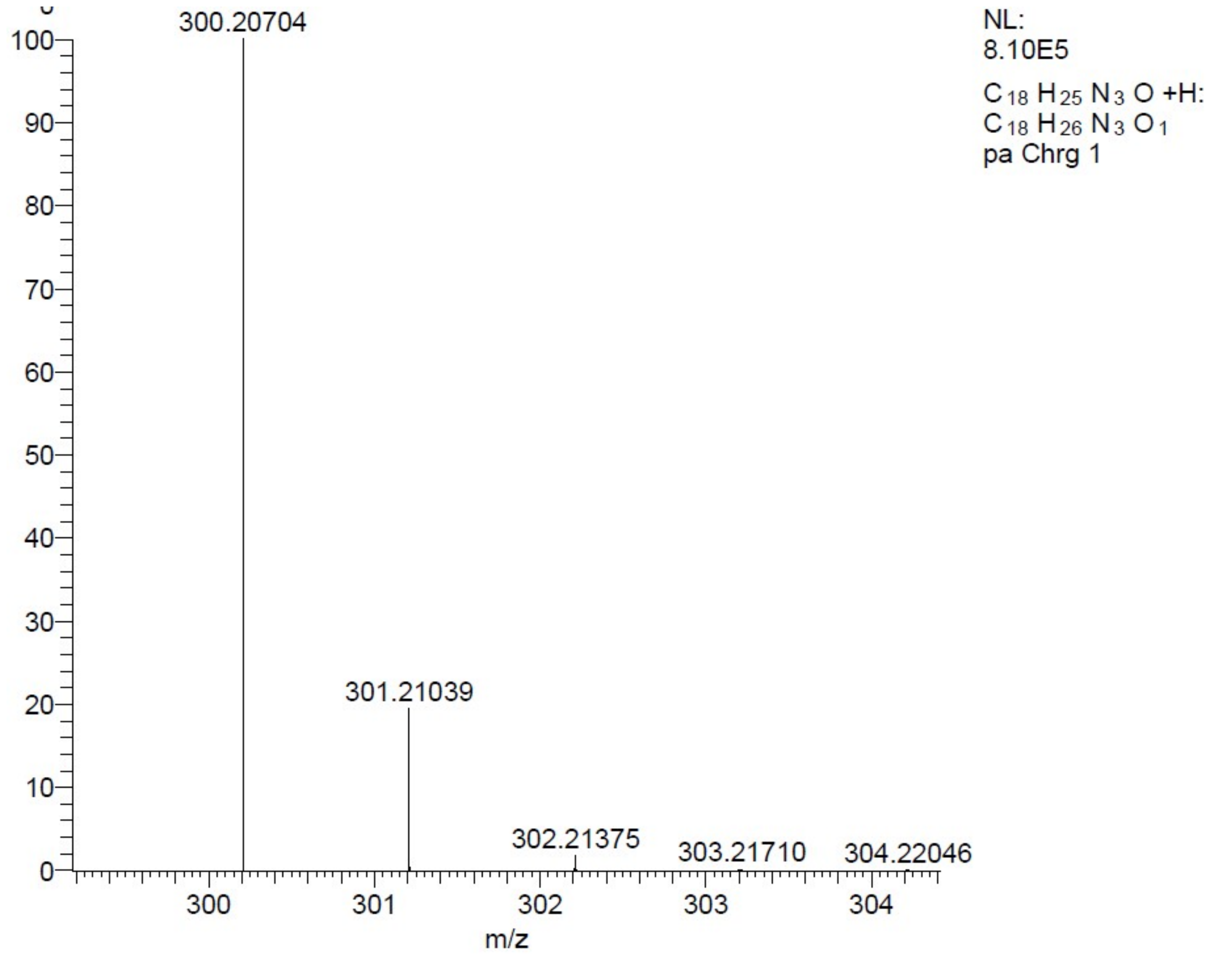

Figure S20. HR-MS (ESI+) of ligand **L7**.

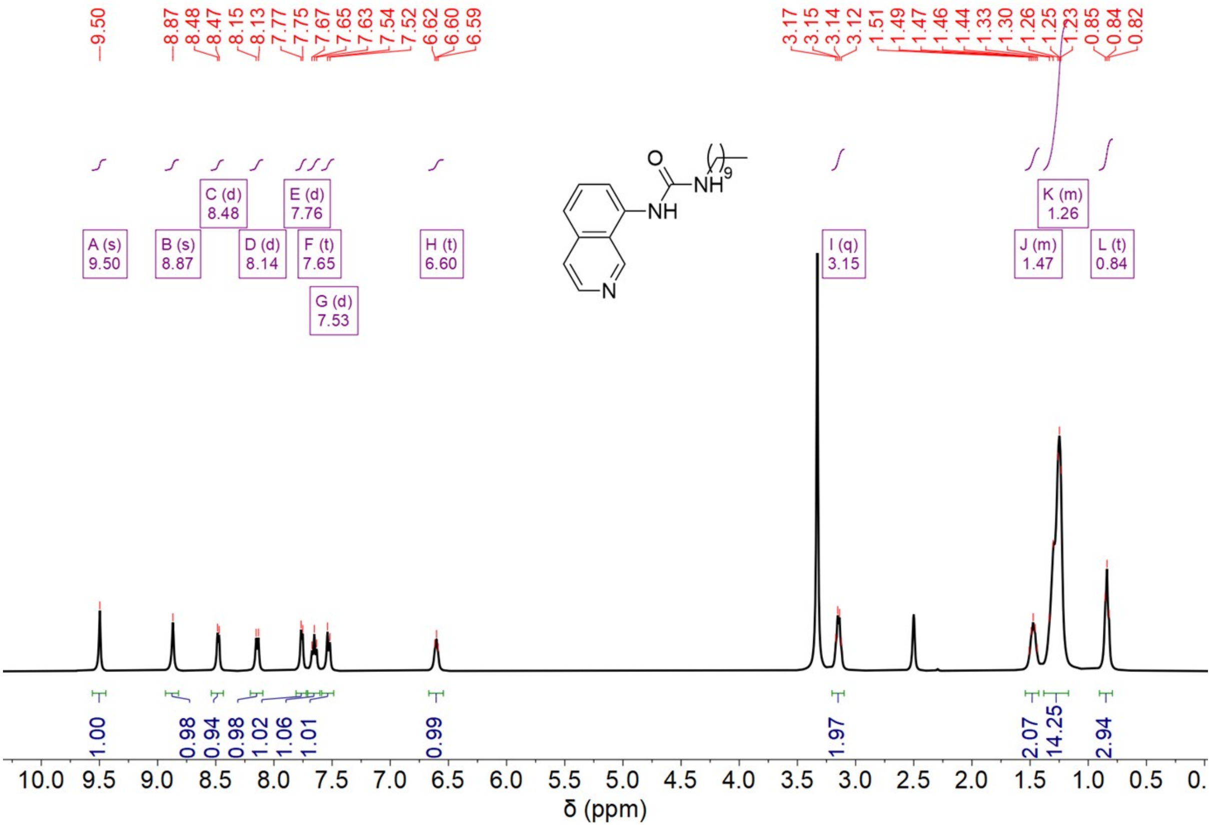

Figure S21. ^{1}H NMR (400 MHz, DMSO-d_6) spectrum of ligand **L8**.

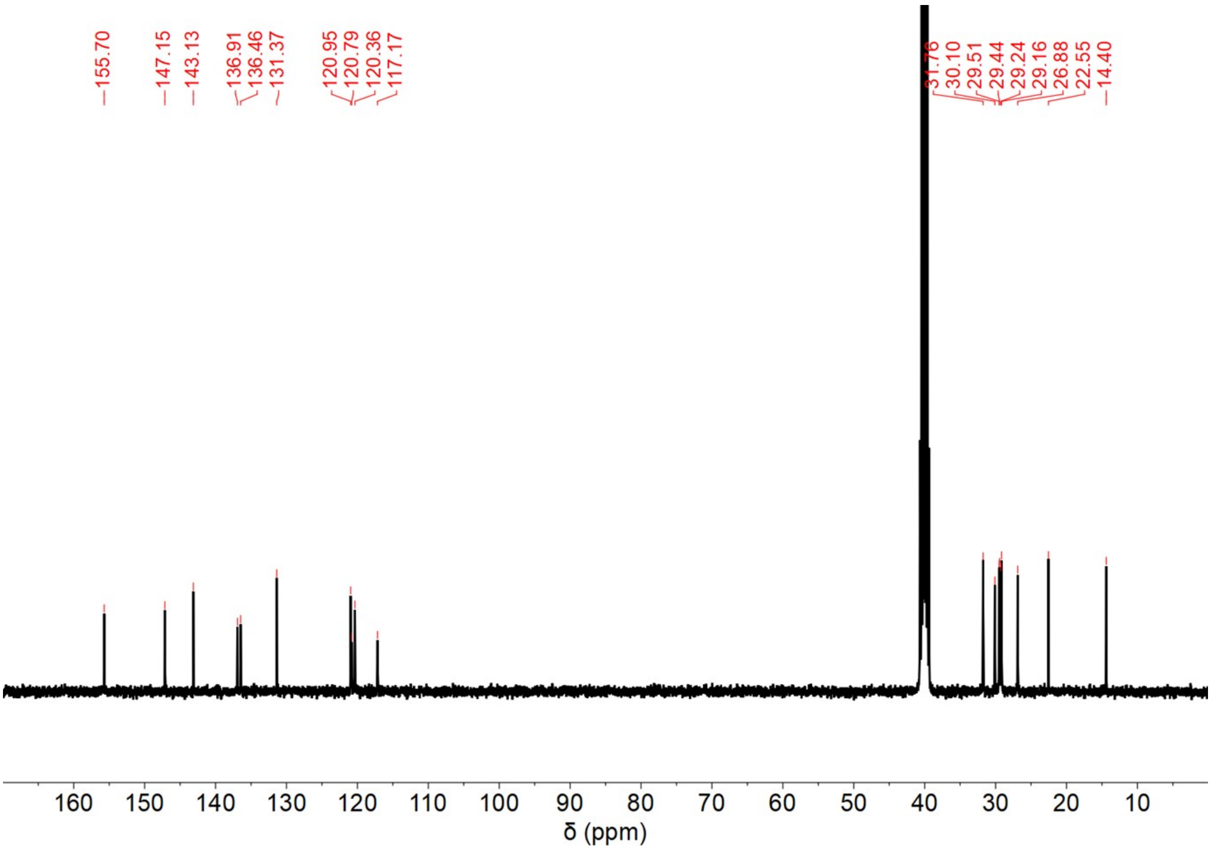

Figure S22. ^{13}C NMR (101 MHz, DMSO-d_6) spectrum of ligand **L8**.

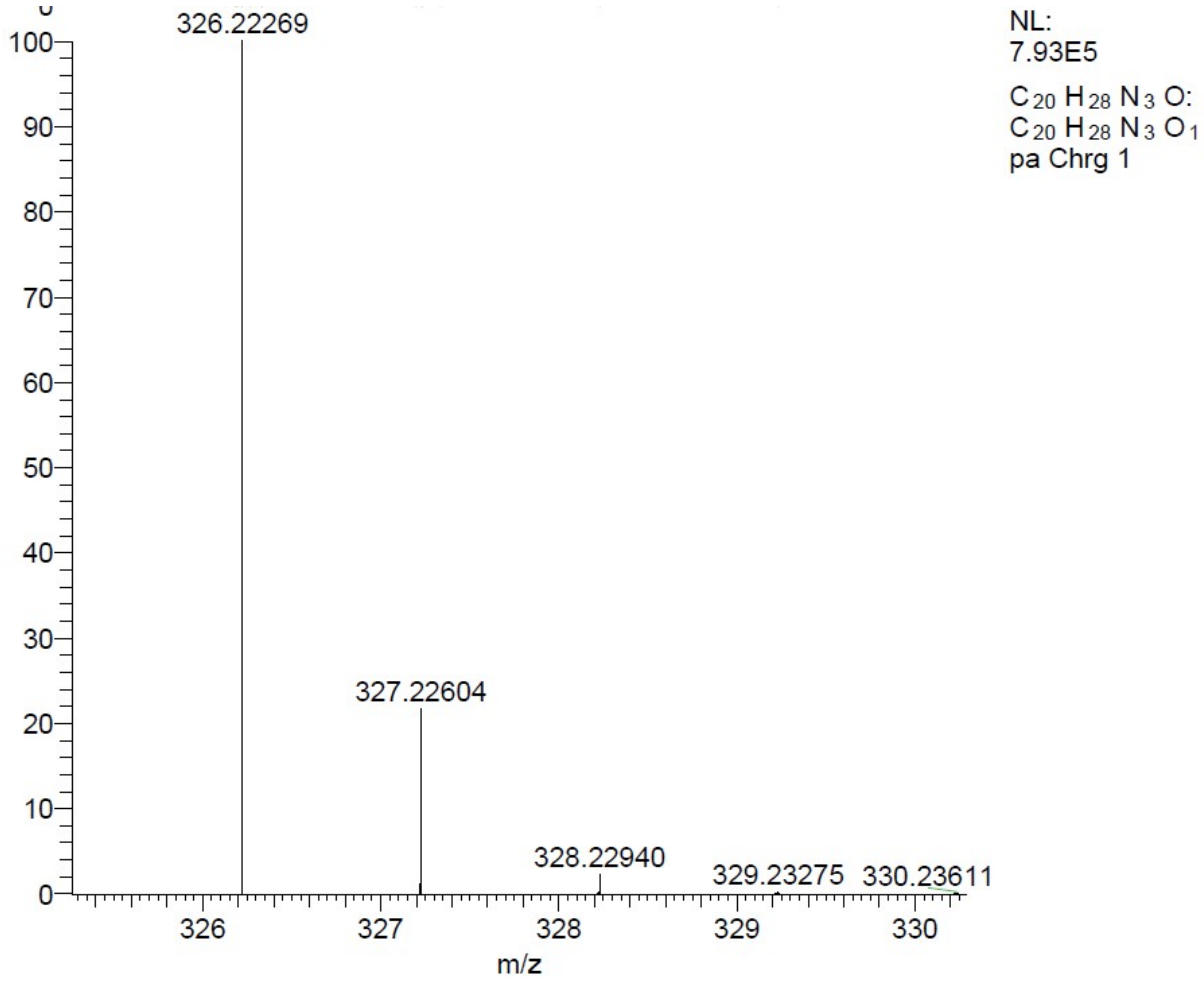

Figure S23. HR-MS (ESI+) of ligand **L8**.

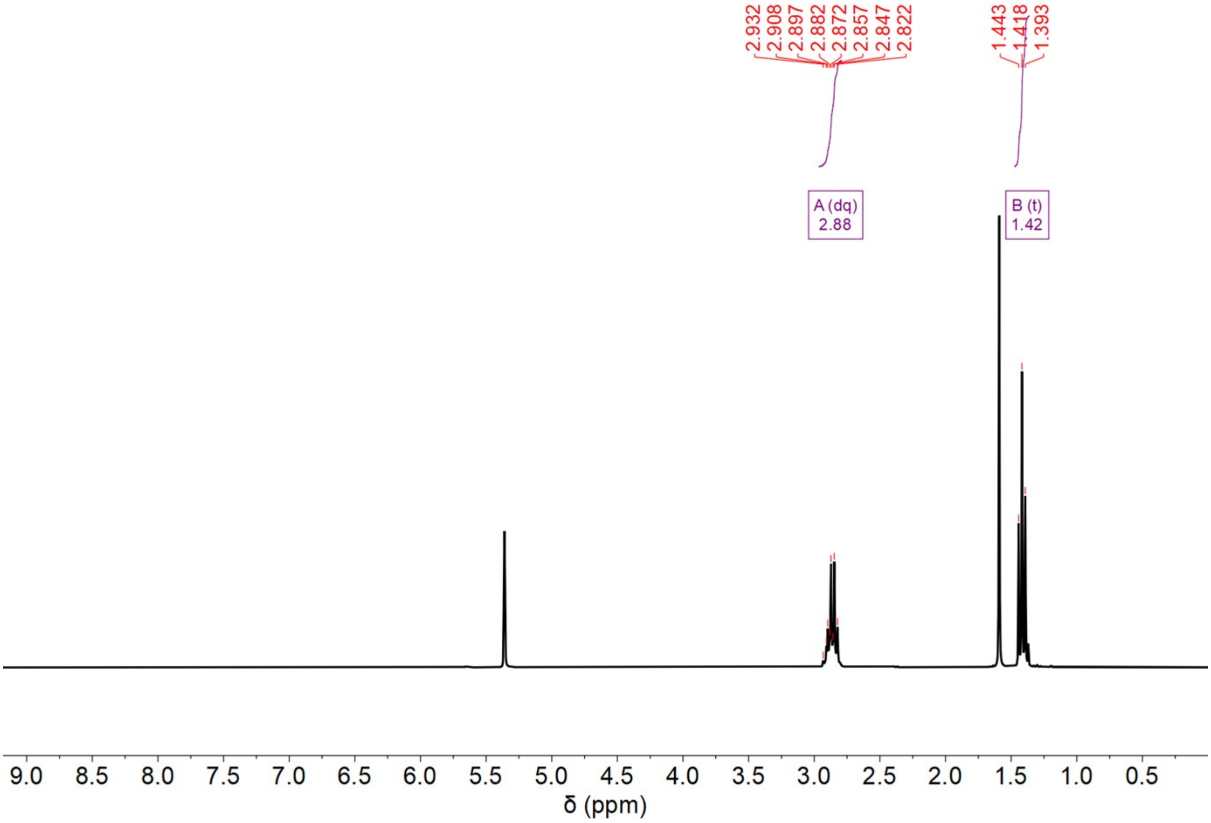

Figure S24. ^{1}H NMR (300 MHz, CD_2Cl_2) spectrum of $[PtCl_2(EtCN)_2]$.

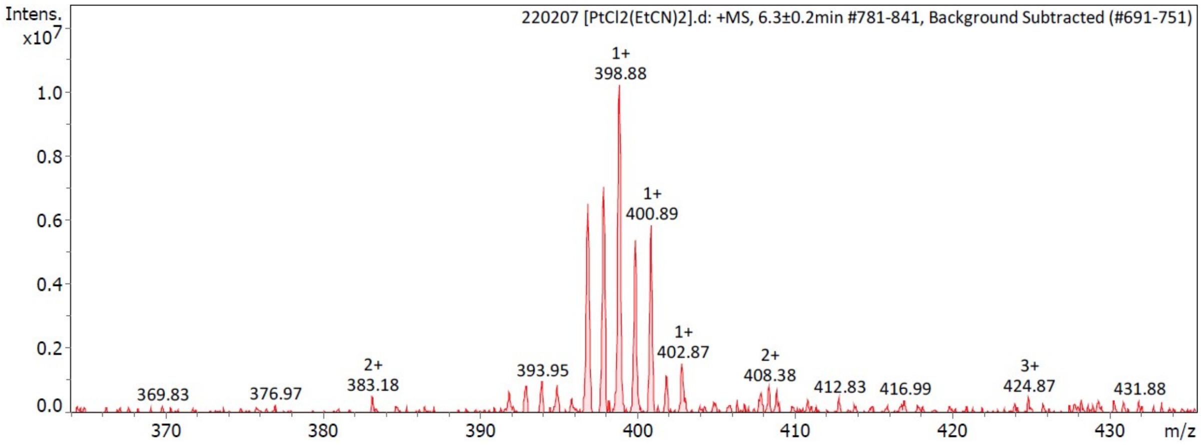

Figure S25. LR-MS (ESI+) of $[PtCl_2(EtCN)_2]$.

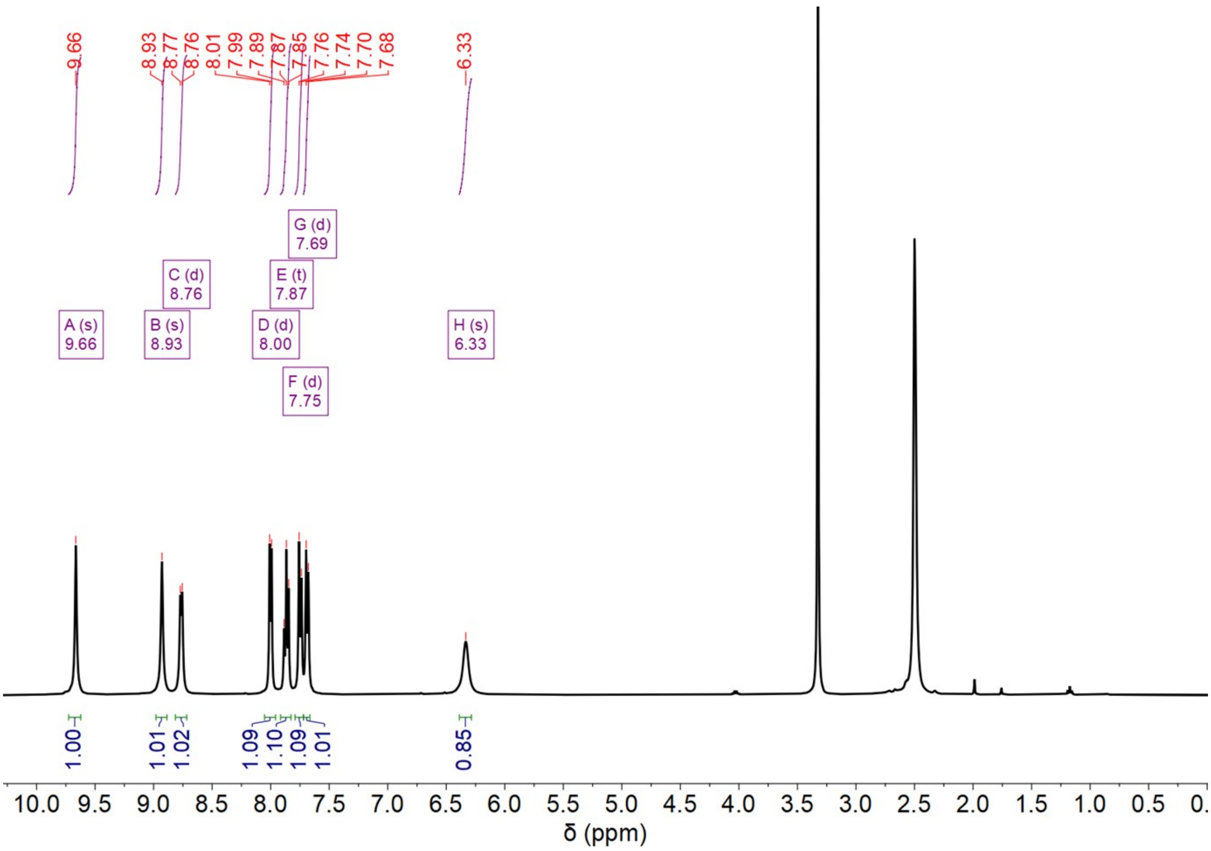

Figure S26. ^{1}H NMR (400 MHz, DMSO-d_6) spectrum of complex **1**. The CH_3 peak is hidden under the residual DMSO solvent peak.

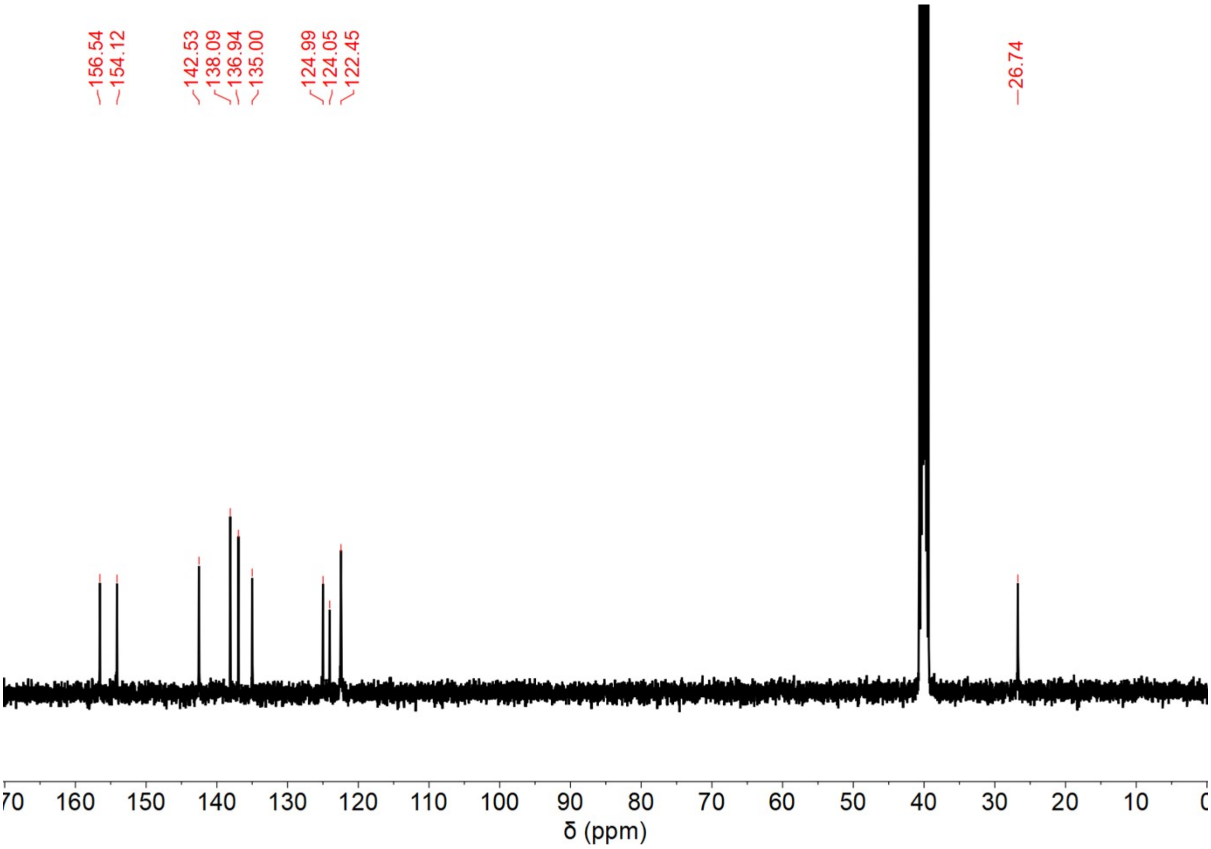

Figure S27. ^{13}C NMR (101 MHz, DMSO-d_6) spectrum of complex **1**.

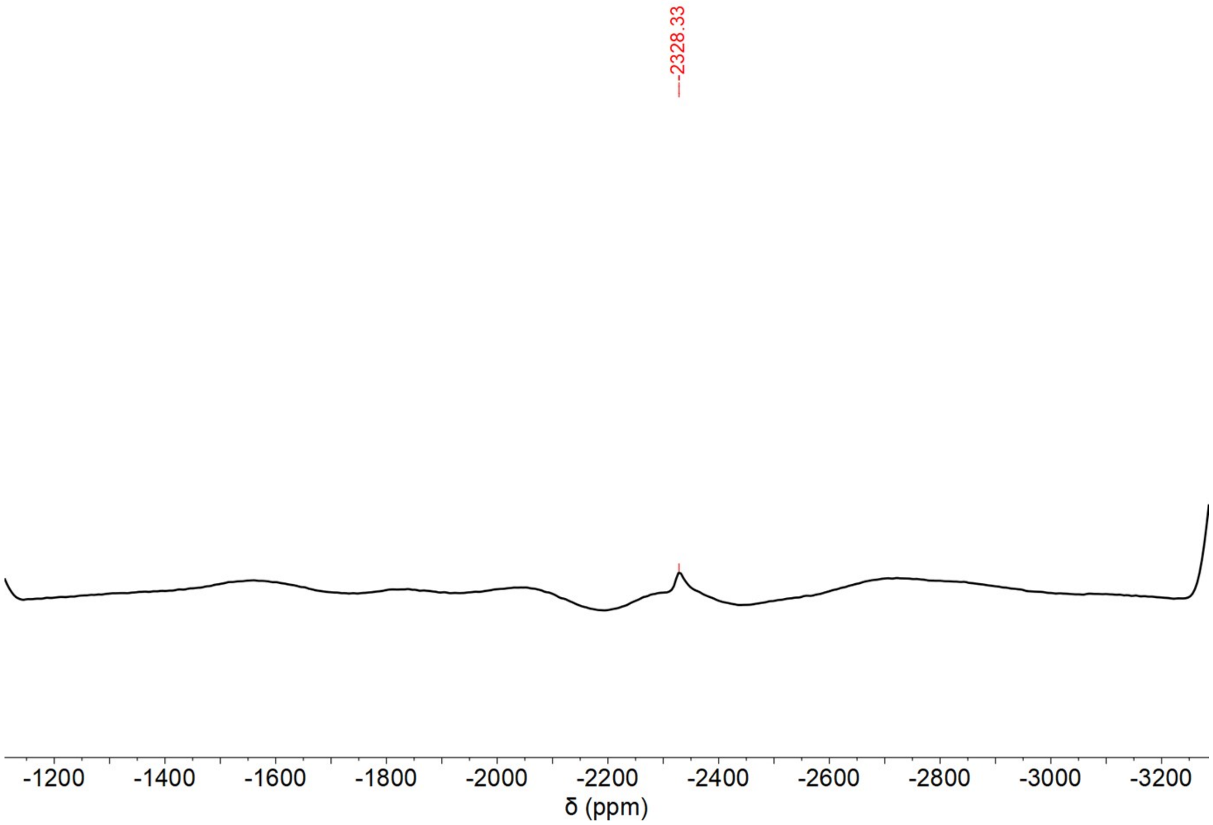

Figure S28. ^{195}Pt NMR (86 MHz, DMSO-d_6) spectrum of complex **1**.

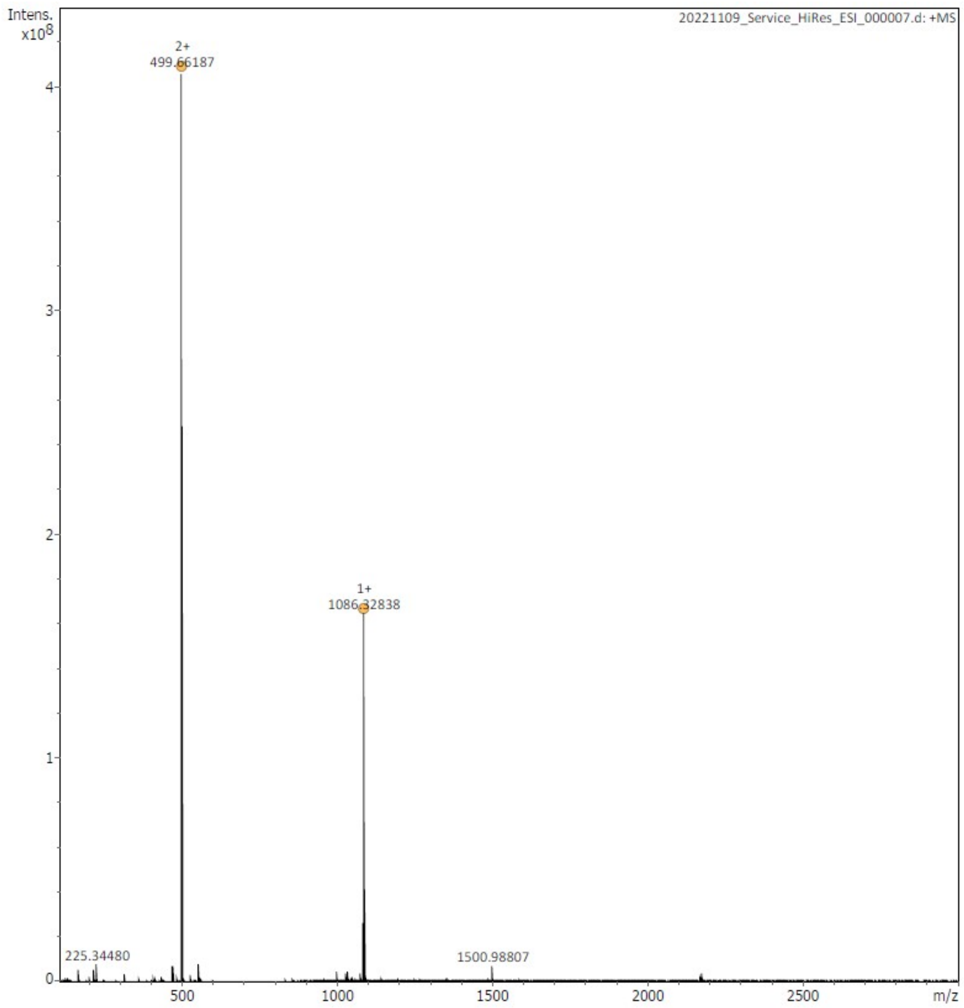

Figure S29. HR-MS (ESI+) of complex **1**.

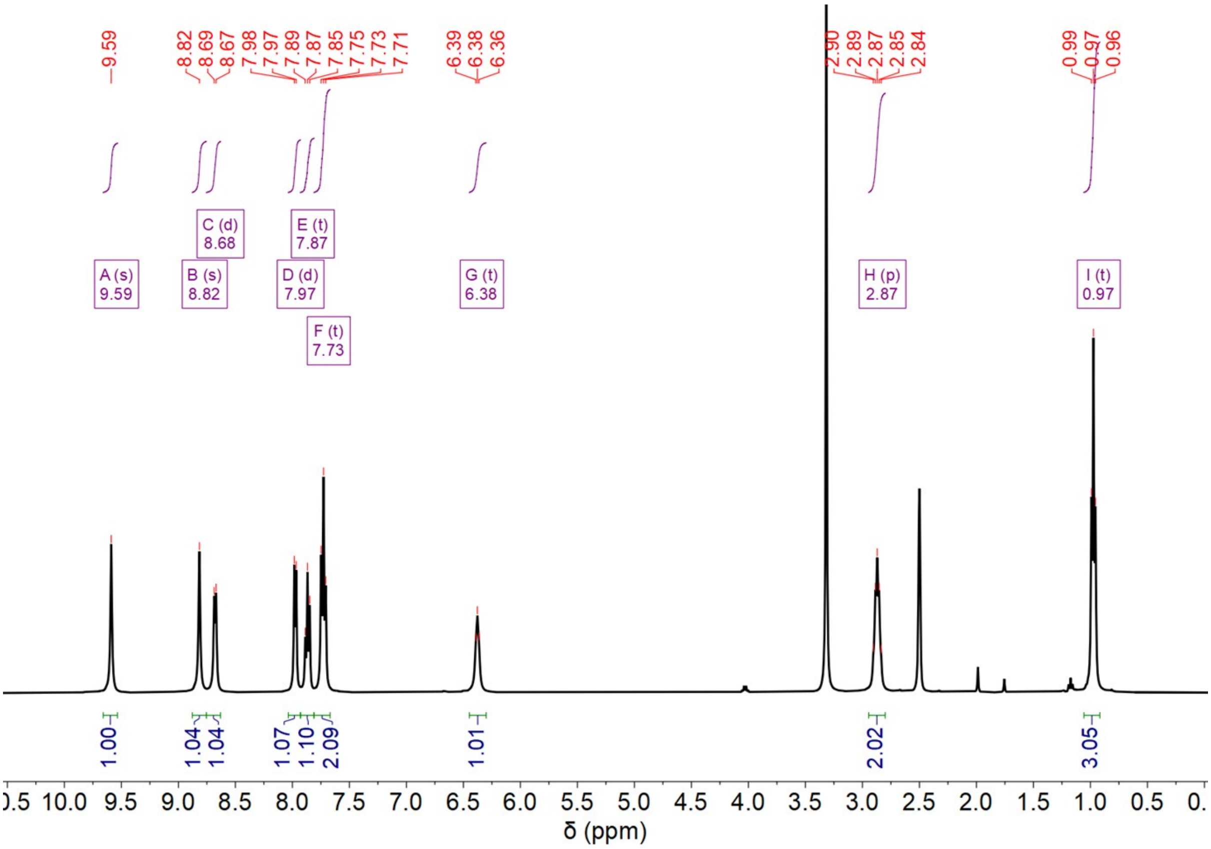

Figure S30. ^{1}H NMR (400 MHz, DMSO-d_6) spectrum of complex **2**.

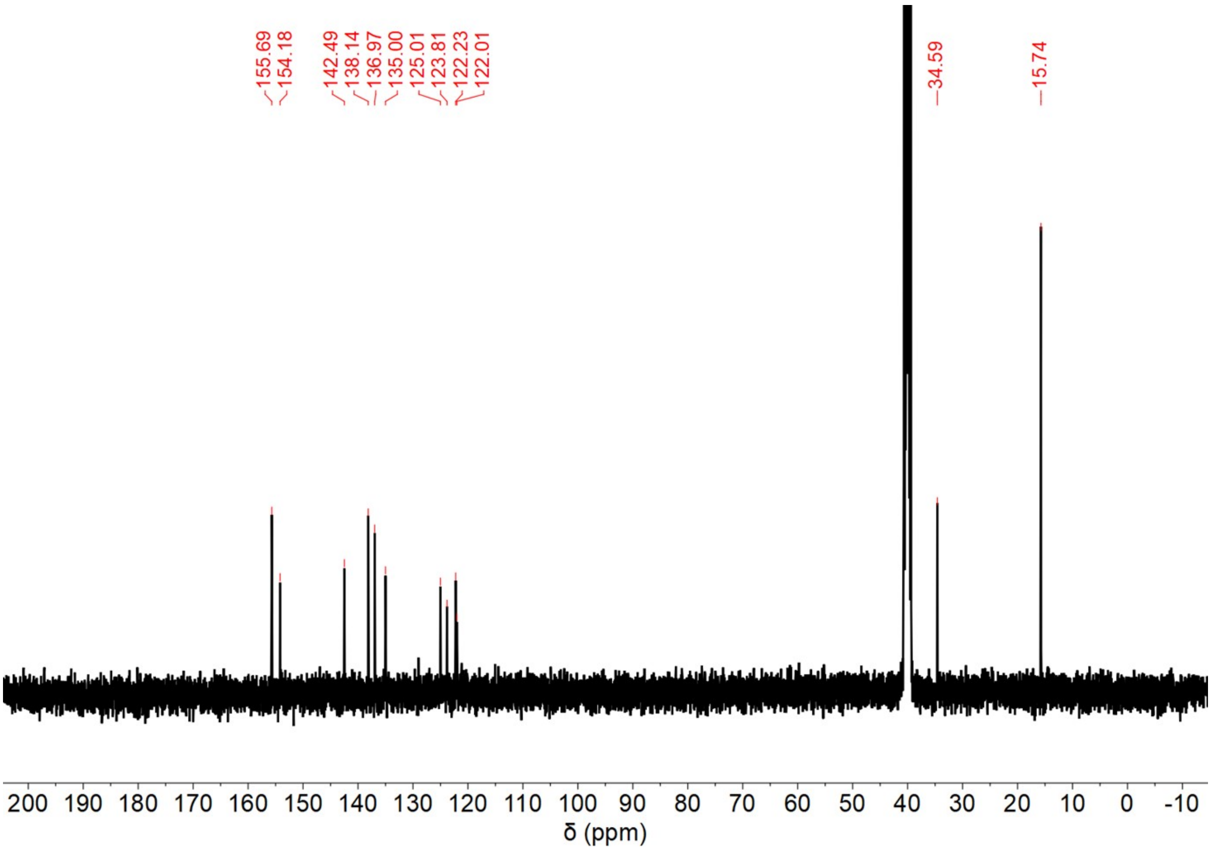

Figure S31. ^{13}C NMR (101 MHz, DMSO-d_6) spectrum of complex **2**.

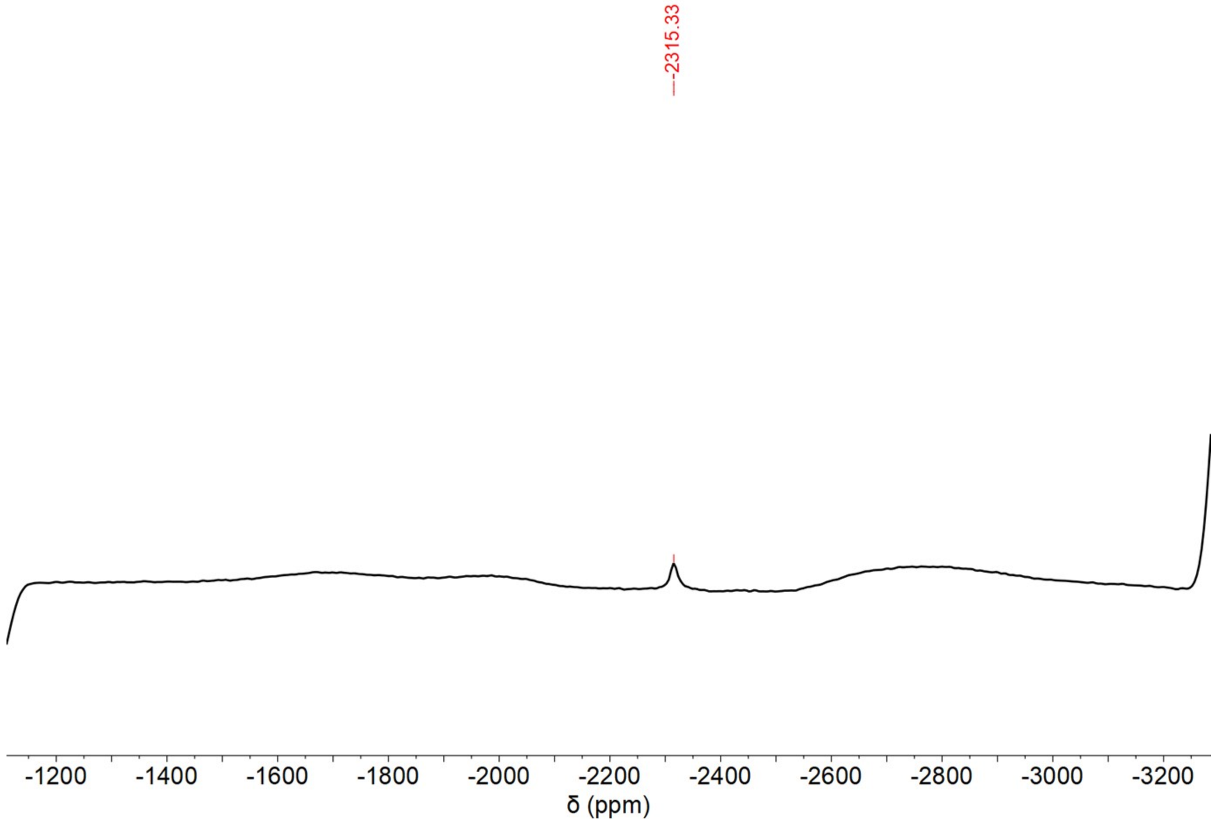

Figure S32. ^{195}Pt NMR (86 MHz, DMSO-d_6) spectrum of complex **2**.

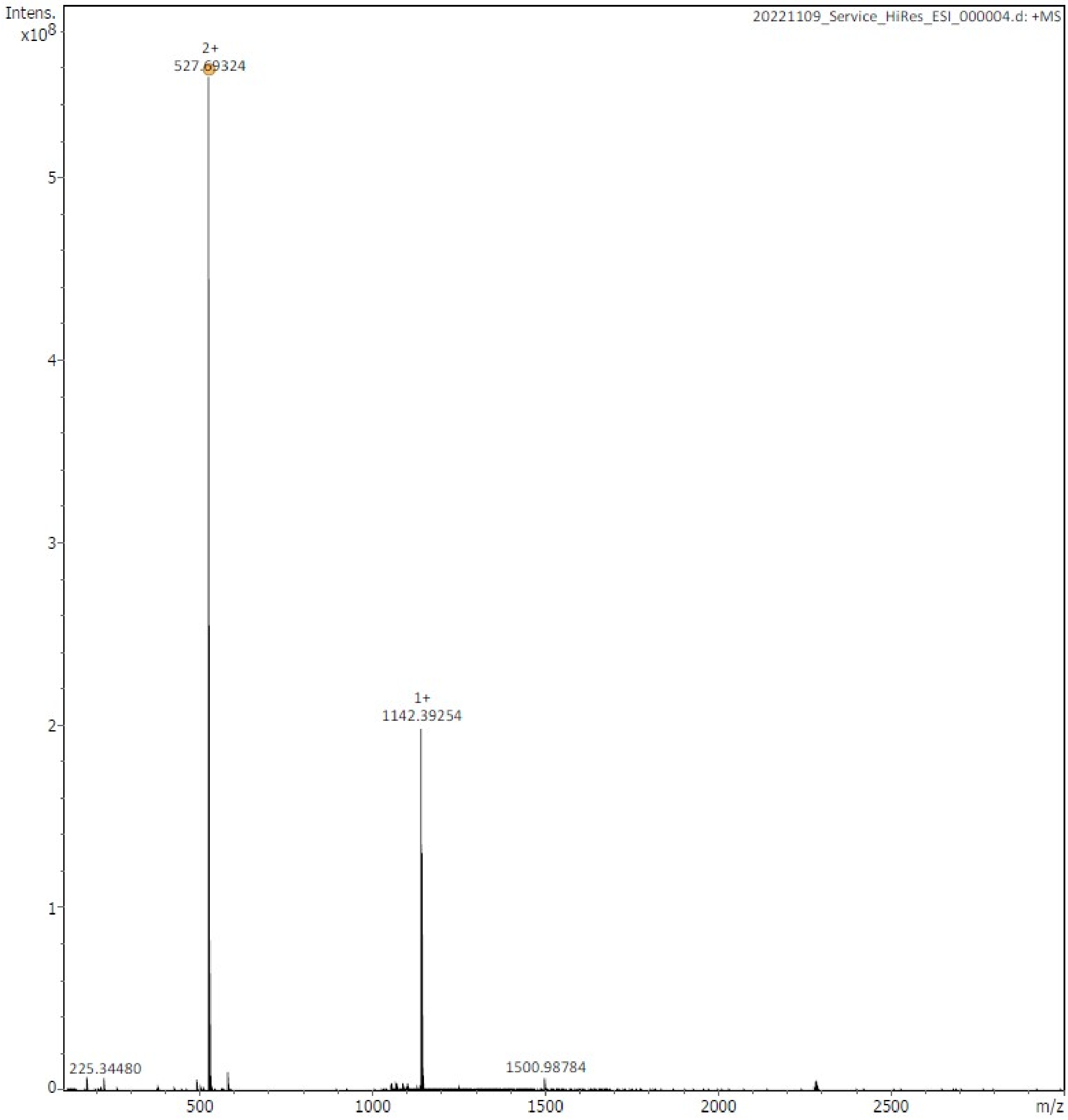

Figure S33. HR-MS (ESI+) of complex **2**.

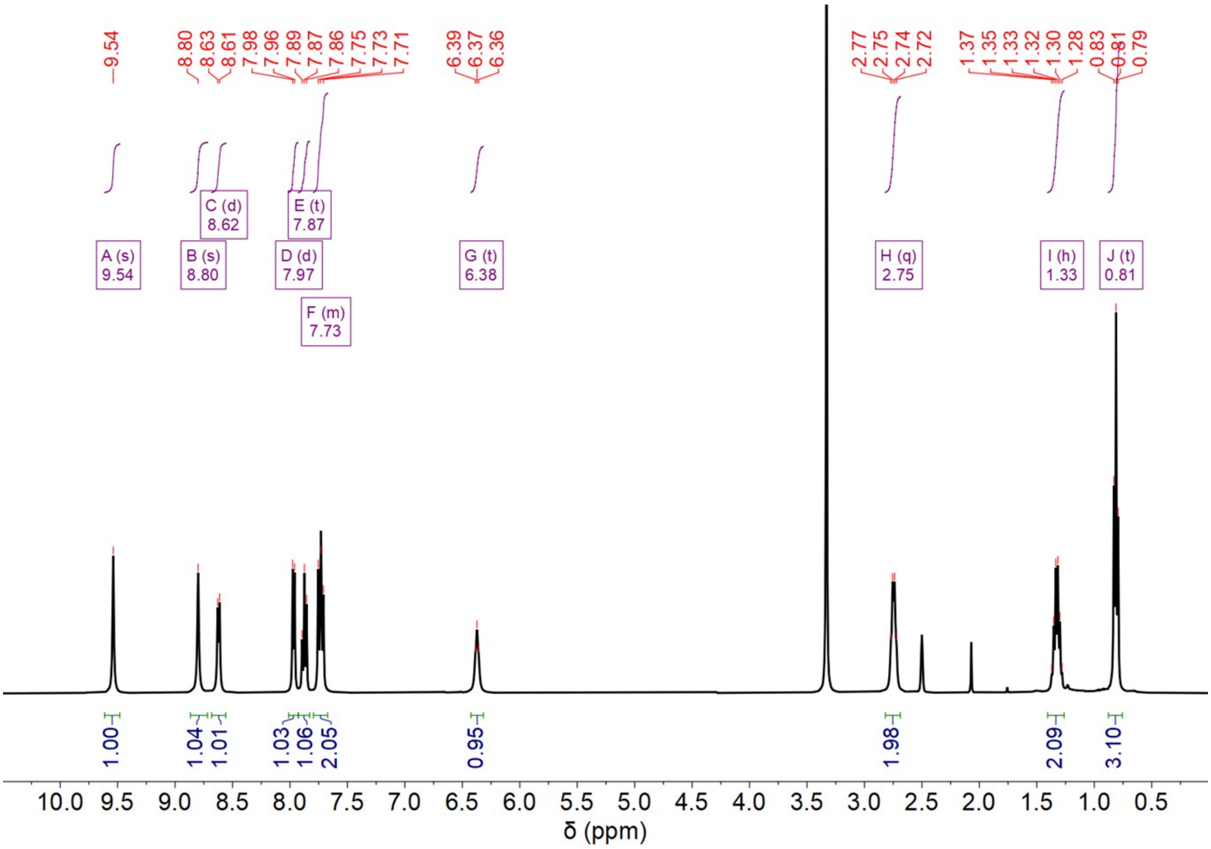

Figure S34. ^{1}H NMR (400 MHz, DMSO-d_6) spectrum of complex **3**.

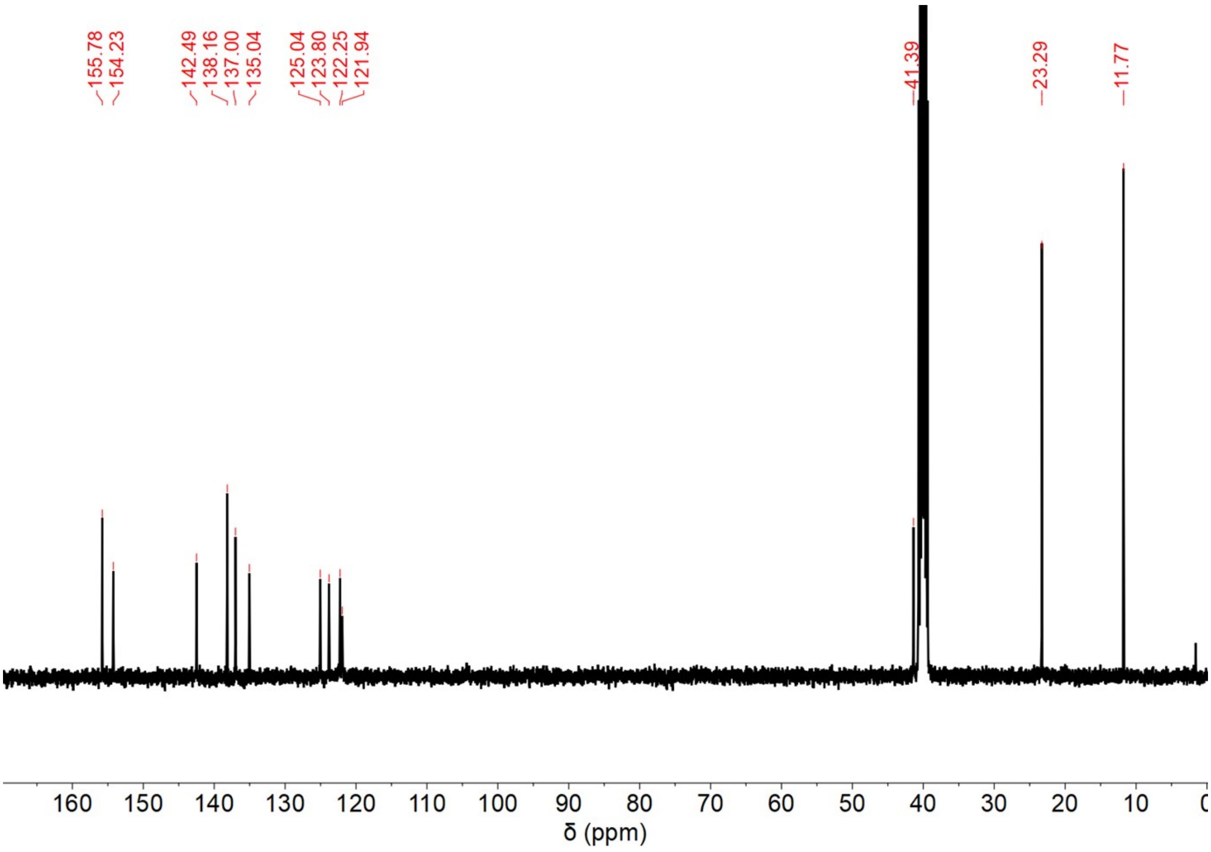

Figure S35. ^{13}C NMR (101 MHz, DMSO-d_6) spectrum of complex **3**.

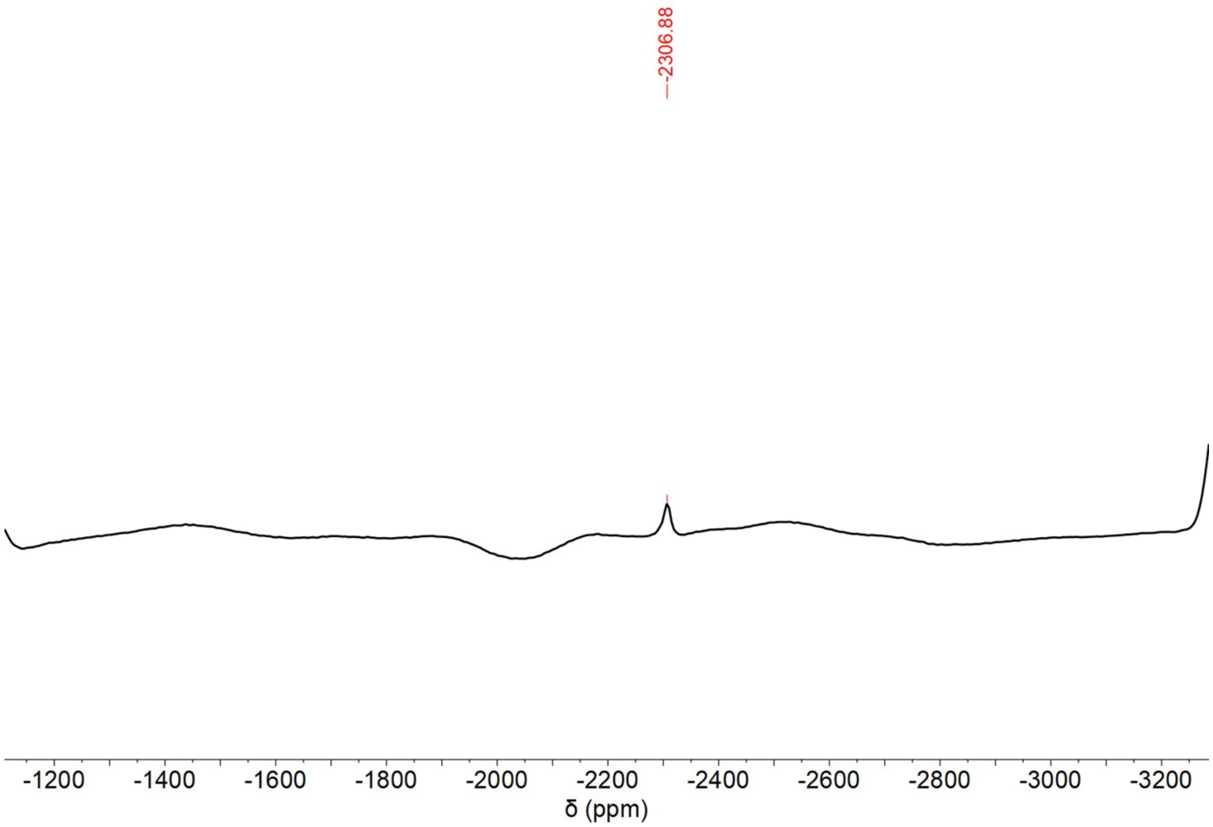

Figure S36. ^{195}Pt NMR (86 MHz, DMSO-d_6) spectrum of complex **3**.

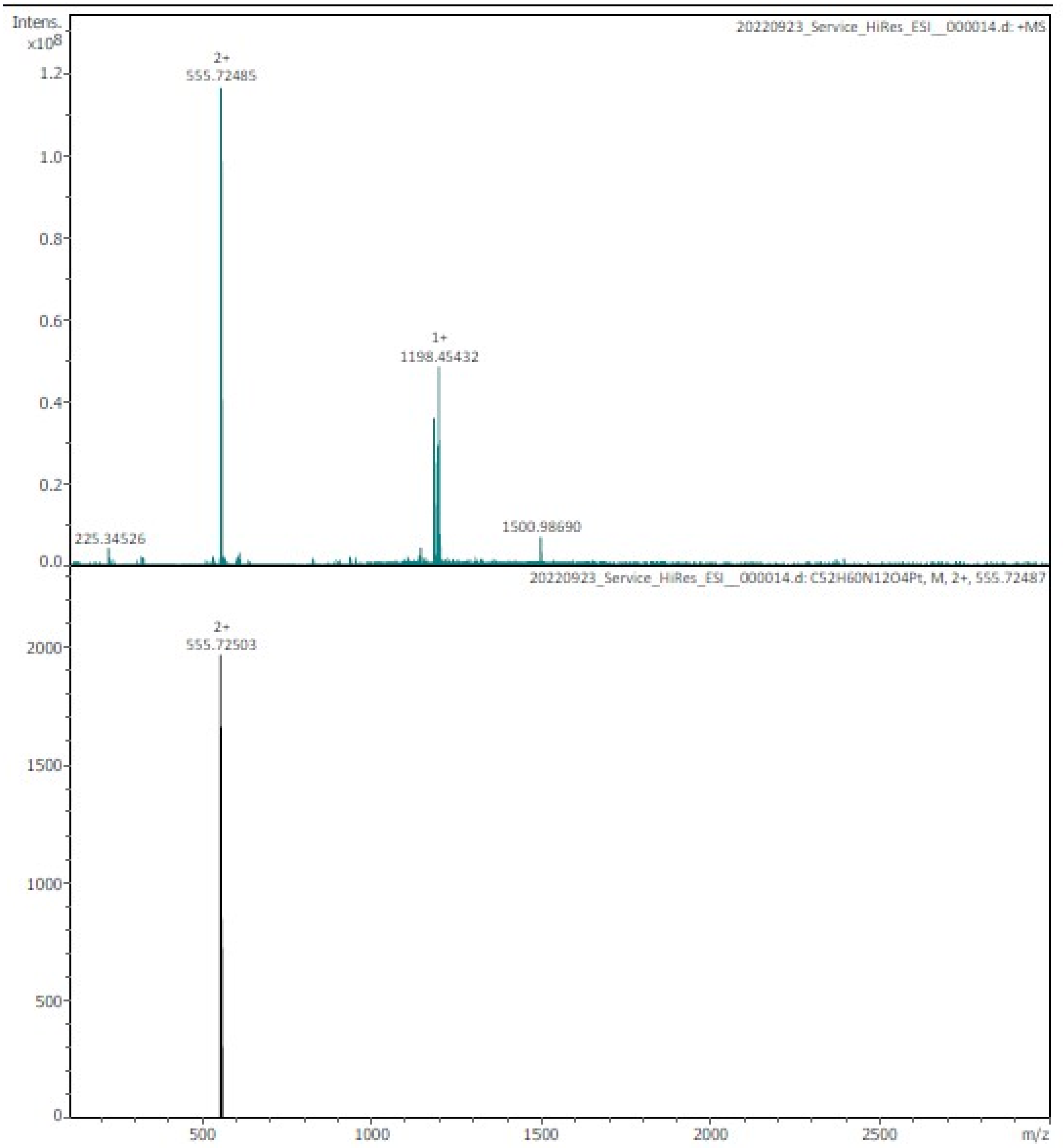

Figure S37. HR-MS (ESI+) of complex **3**.

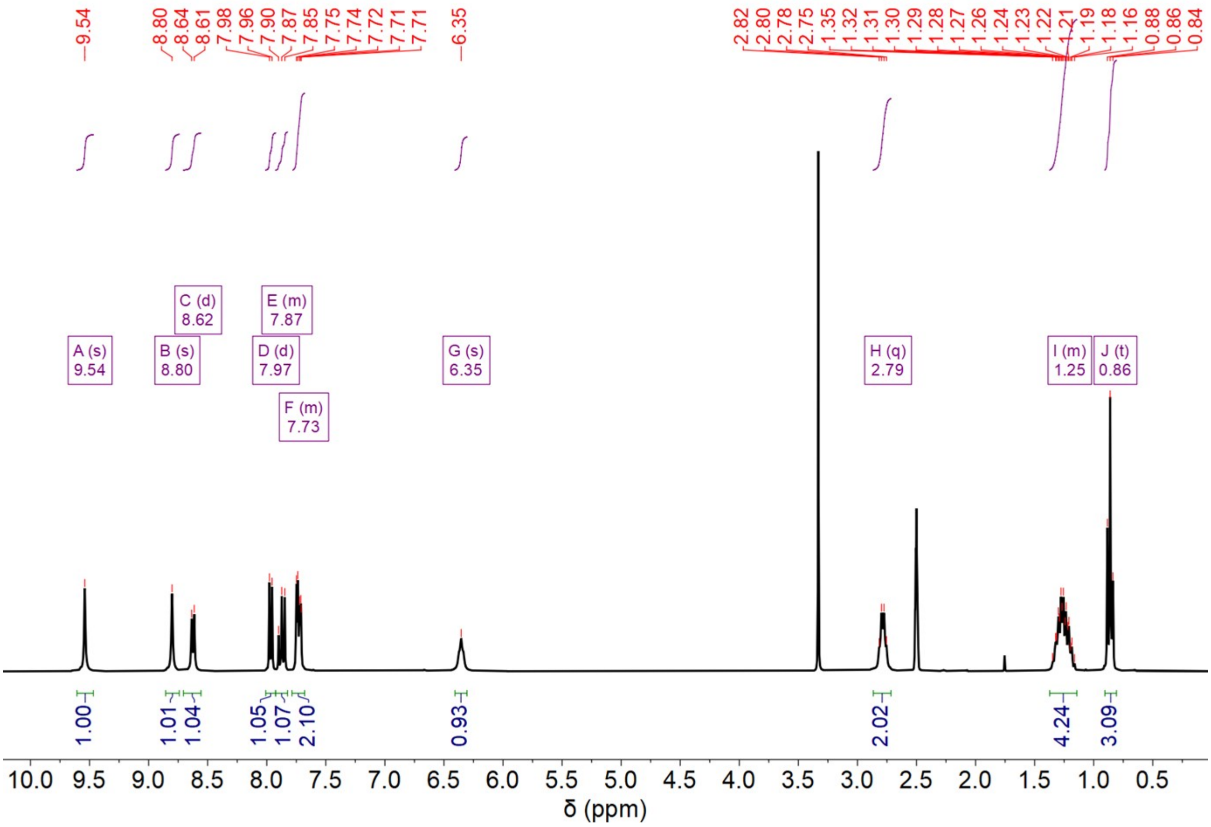

Figure S38. ^{1}H NMR (300 MHz, DMSO-d_6) spectrum of complex **4.**

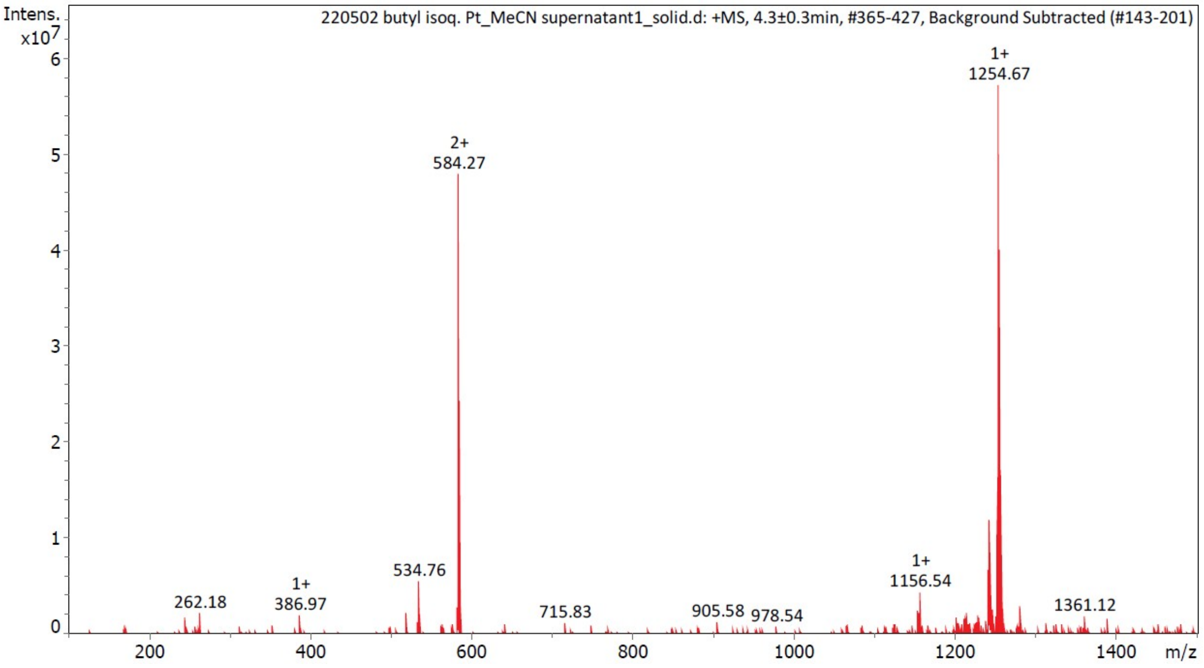

Figure S39. LR-MS (ESI+) of complex **4.**

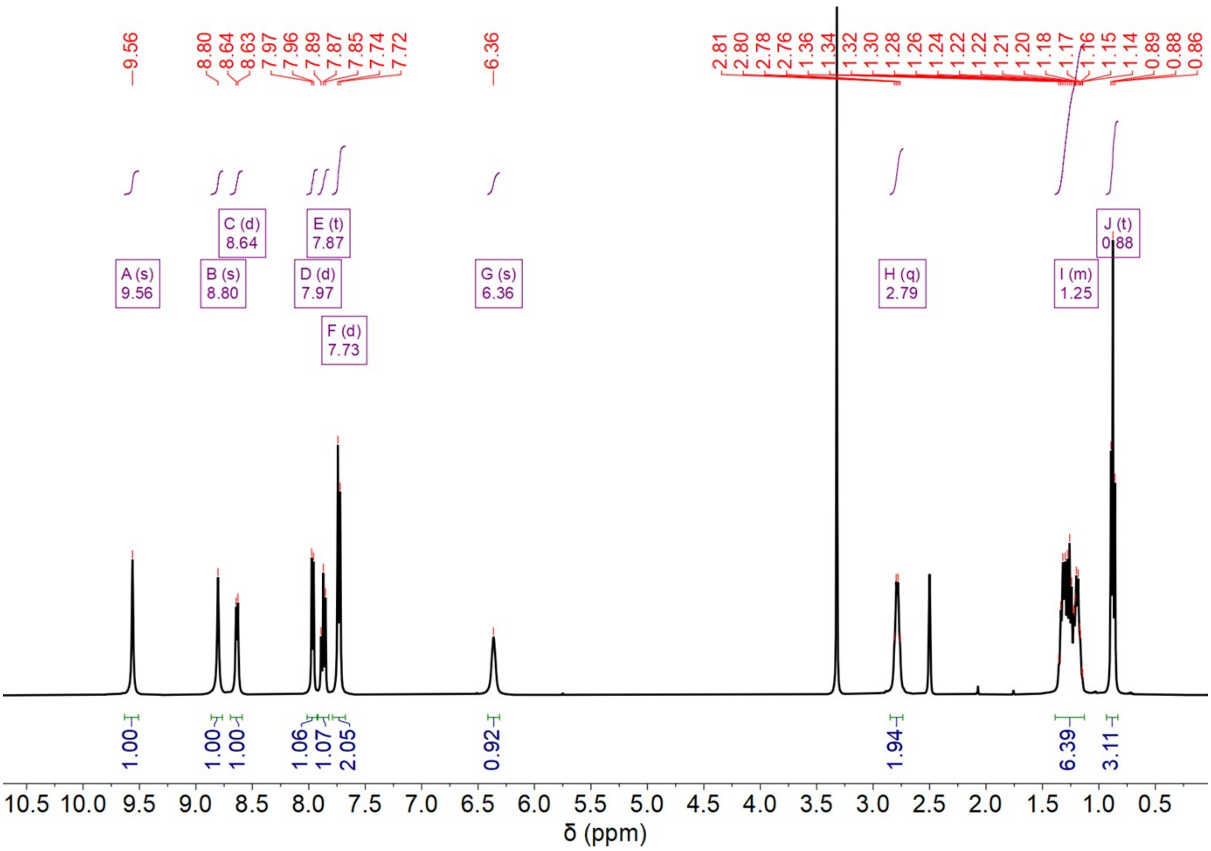

Figure S40. ^{1}H NMR (400 MHz, DMSO-d_6) spectrum of complex **5**.

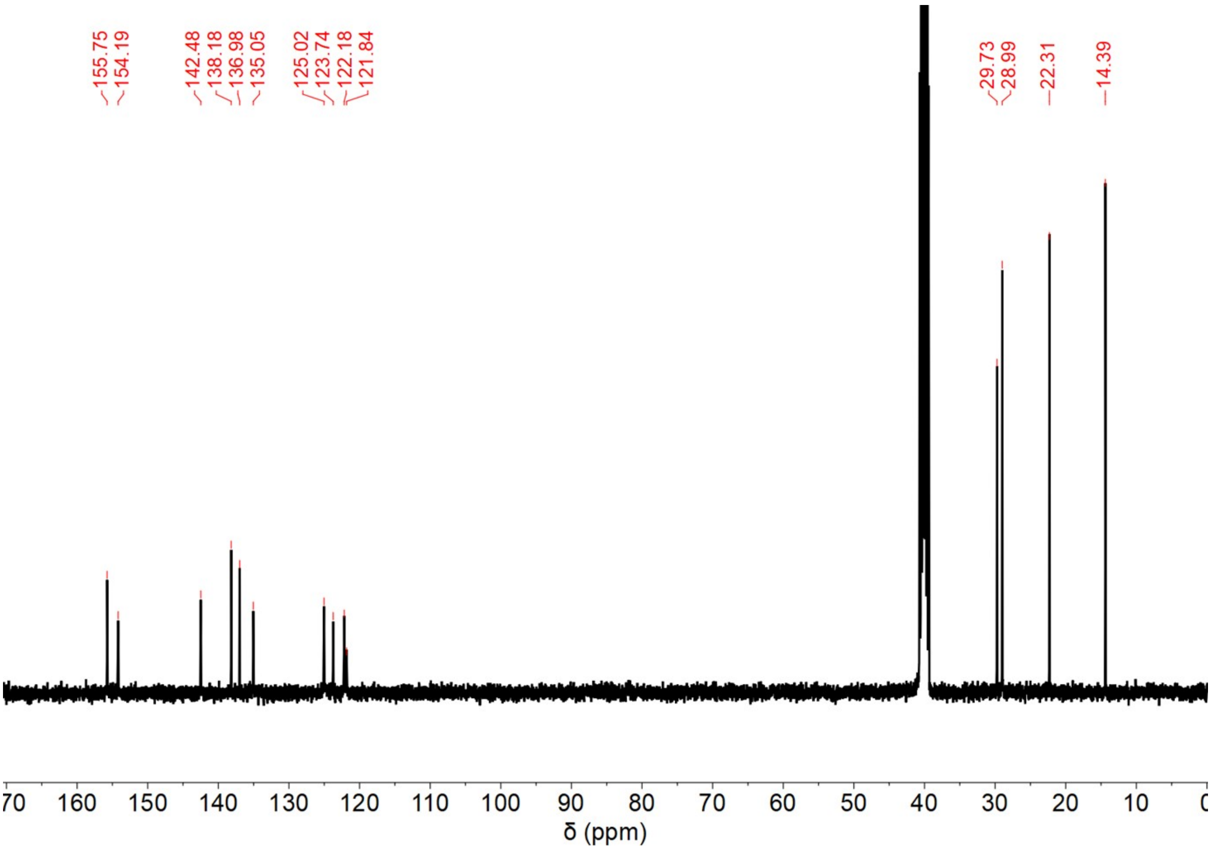

Figure S41. ^{13}C NMR (101 MHz, DMSO-d_6) spectrum of complex **5**.

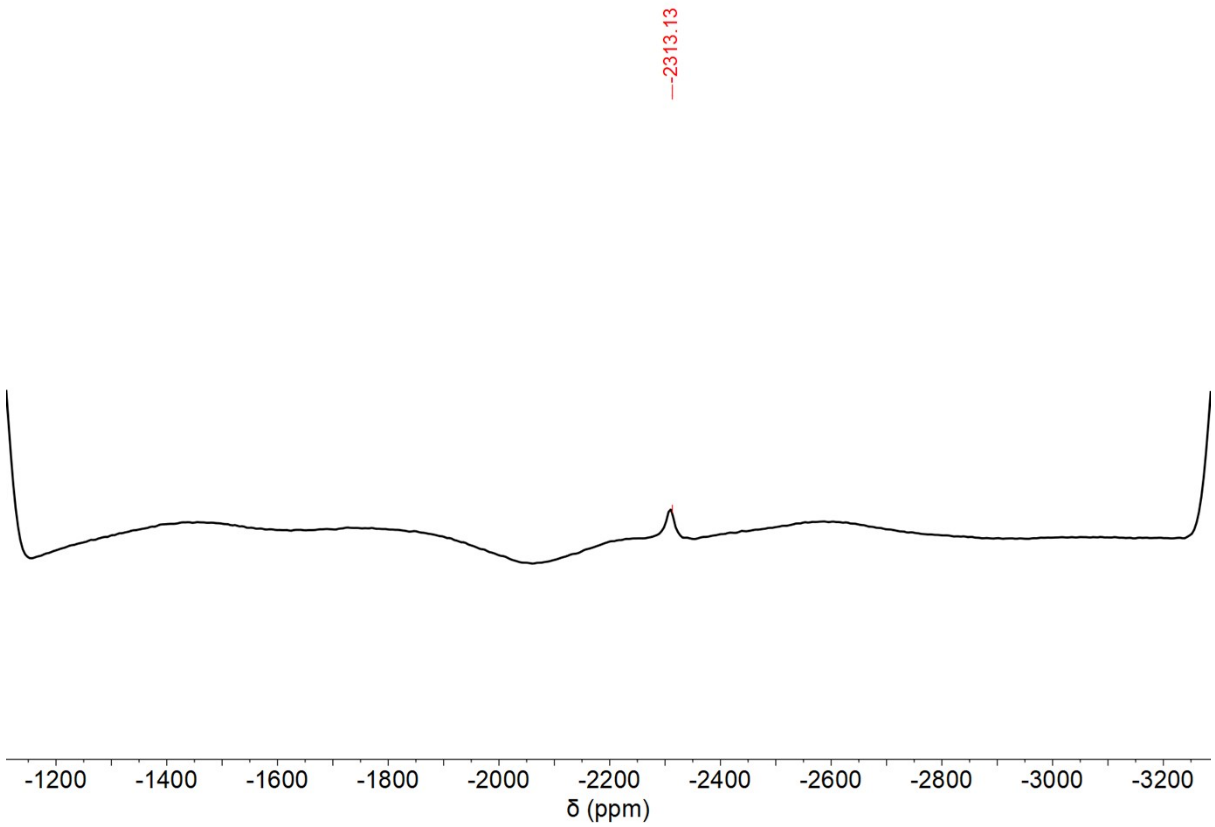

Figure S42. ^{195}Pt NMR (86 MHz, DMSO-d_6) spectrum of complex **5**.

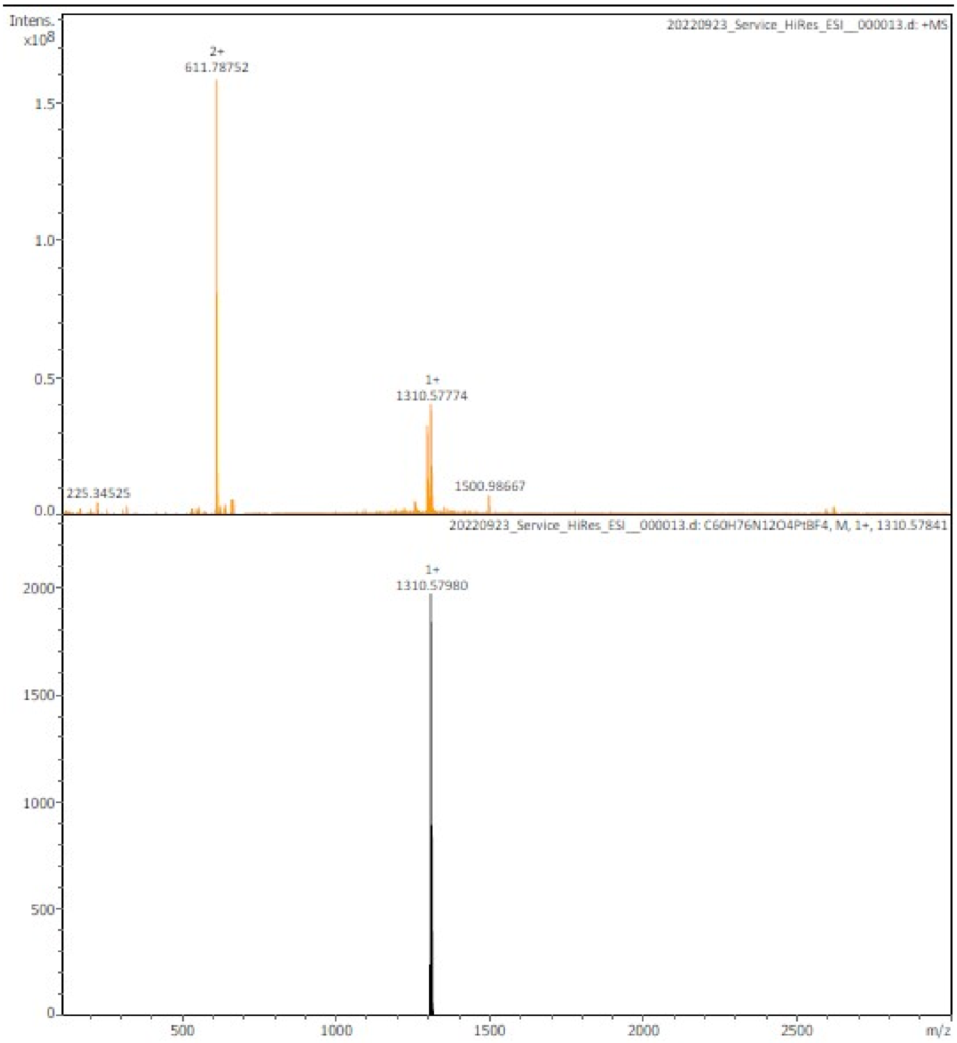

Figure S43. HR-MS (ESI+) of complex **5**.

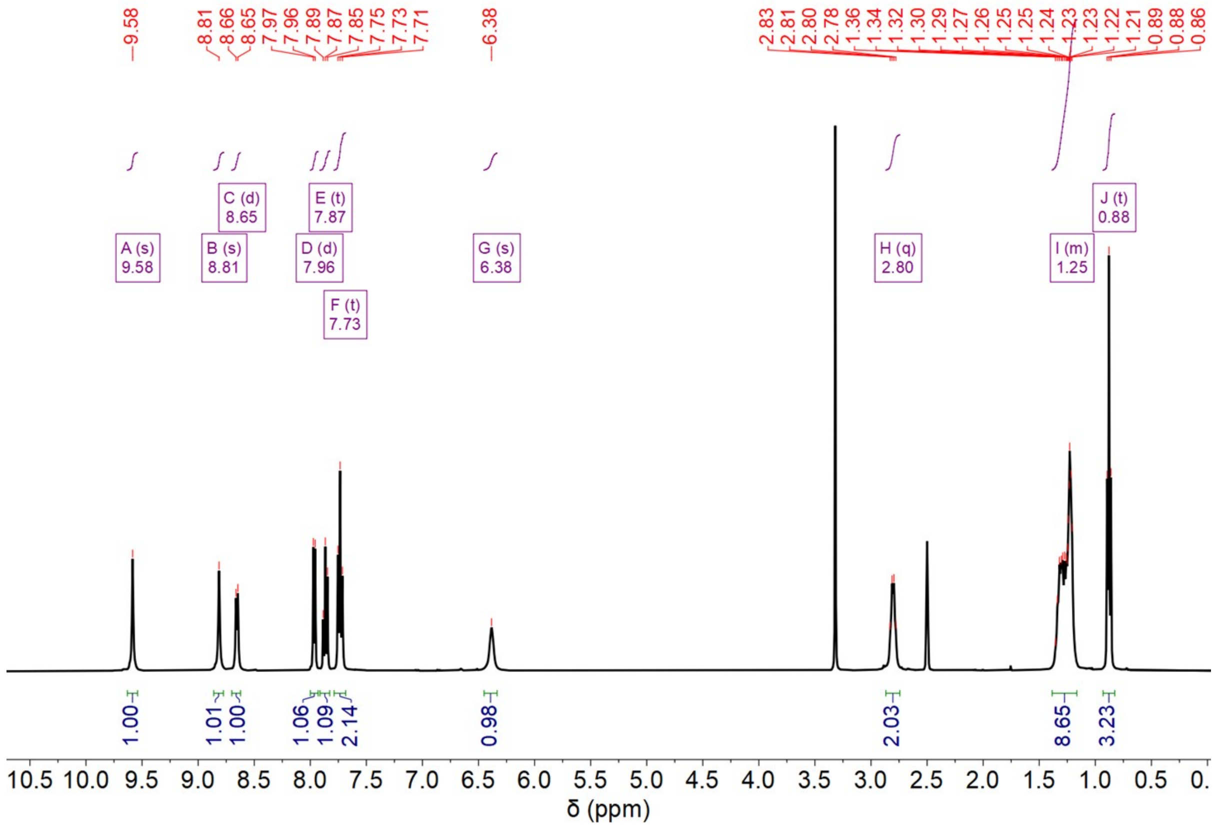

Figure S44. ^{1}H NMR (400 MHz, DMSO-d_6) spectrum of complex **6**.

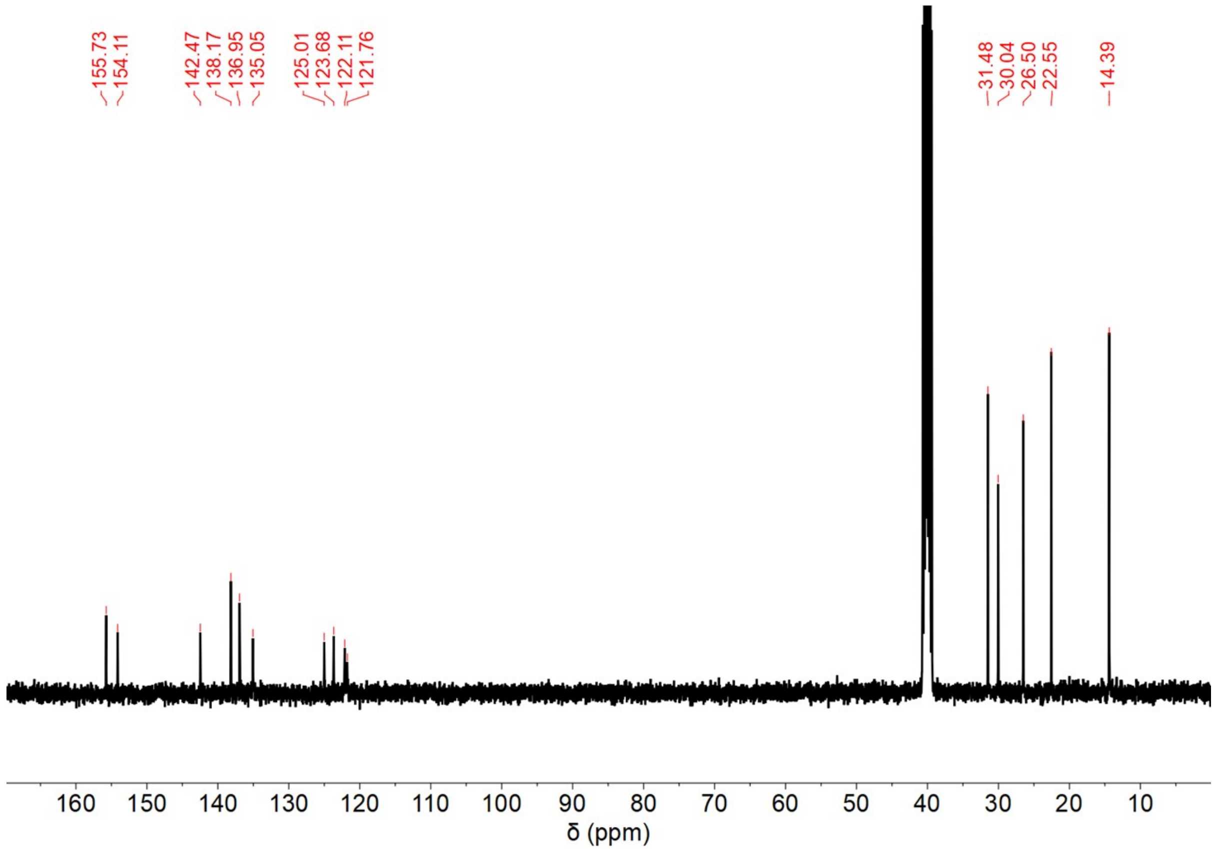

Figure S45. ^{13}C NMR (101 MHz, DMSO-d_6) spectrum of complex **6**.

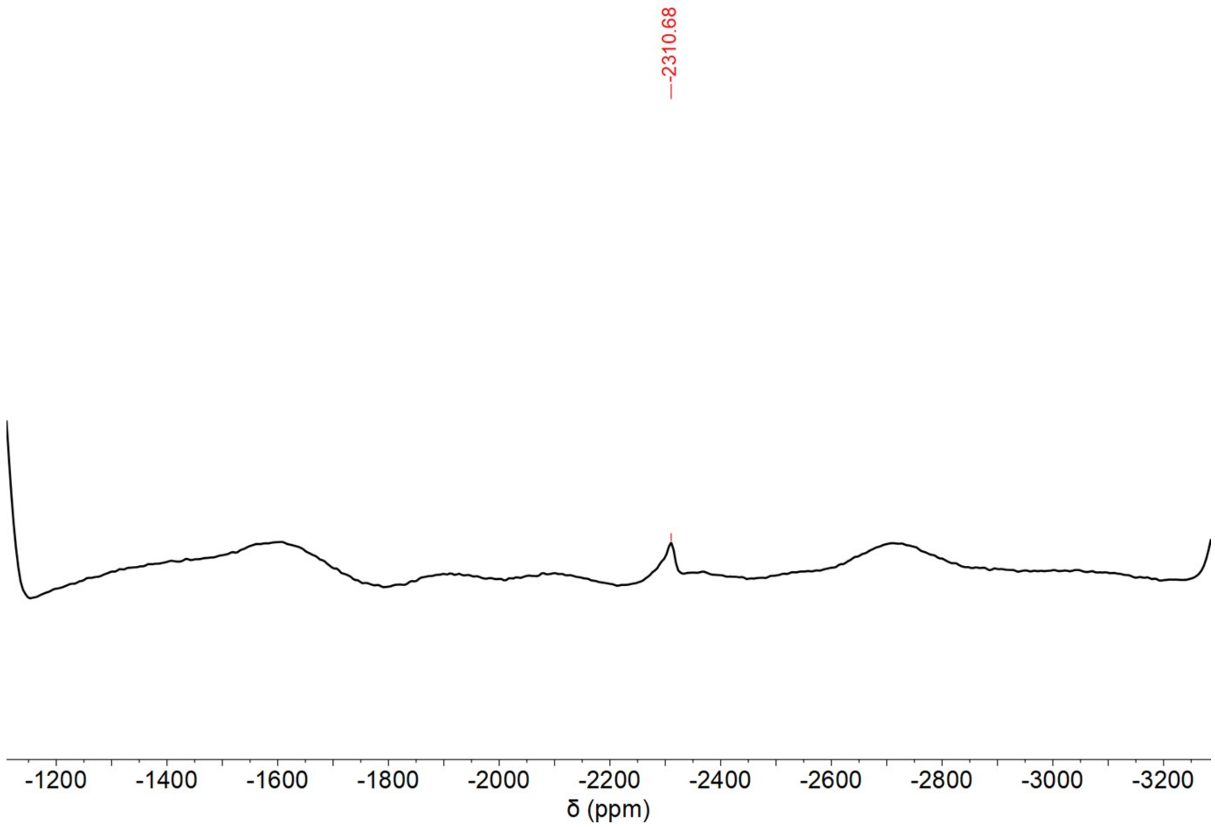

Figure S46. ^{195}Pt NMR (86 MHz, DMSO-d_6) spectrum of complex **6.**

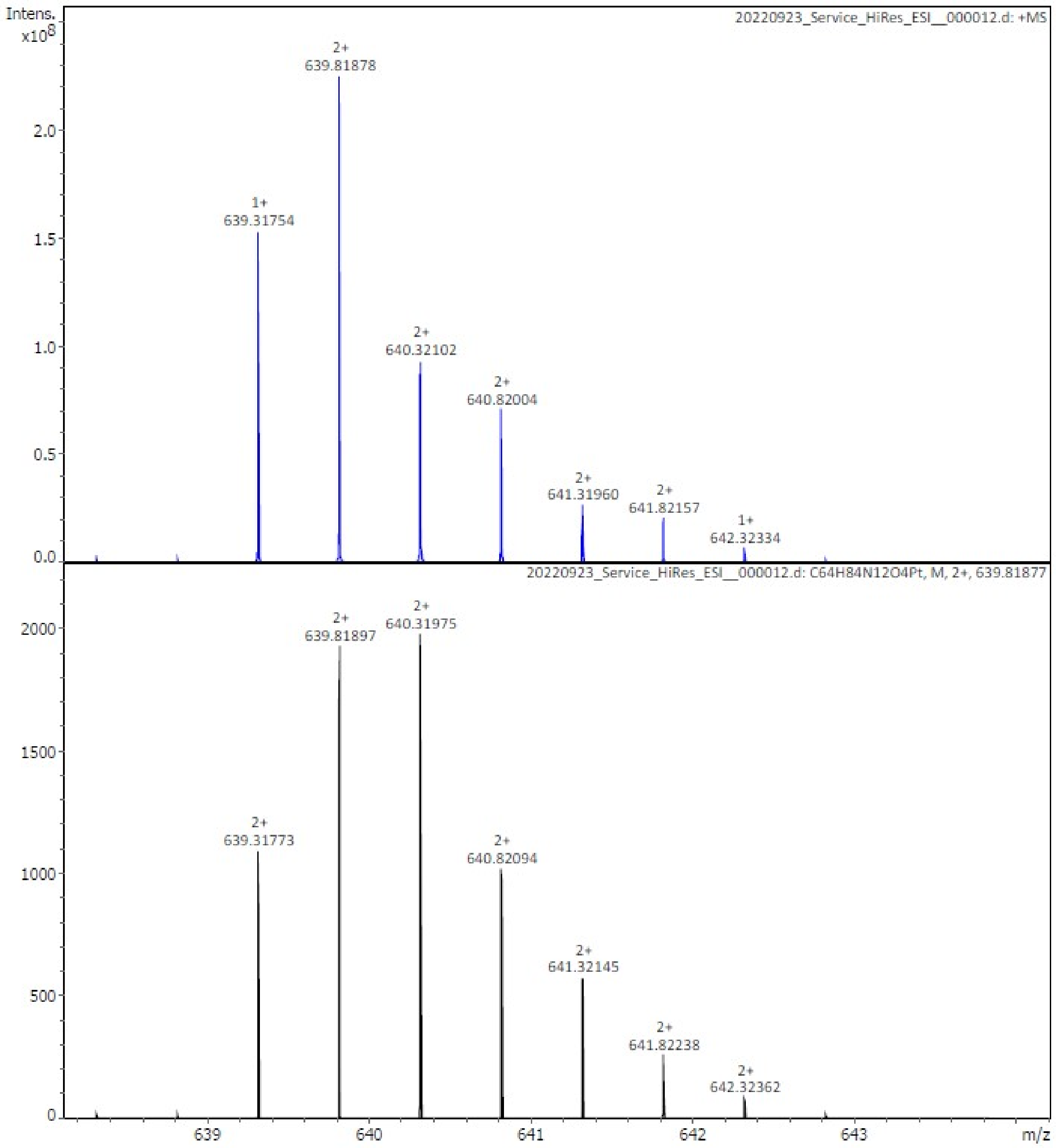

Figure S47. HR-MS (ESI+) of complex **6**.

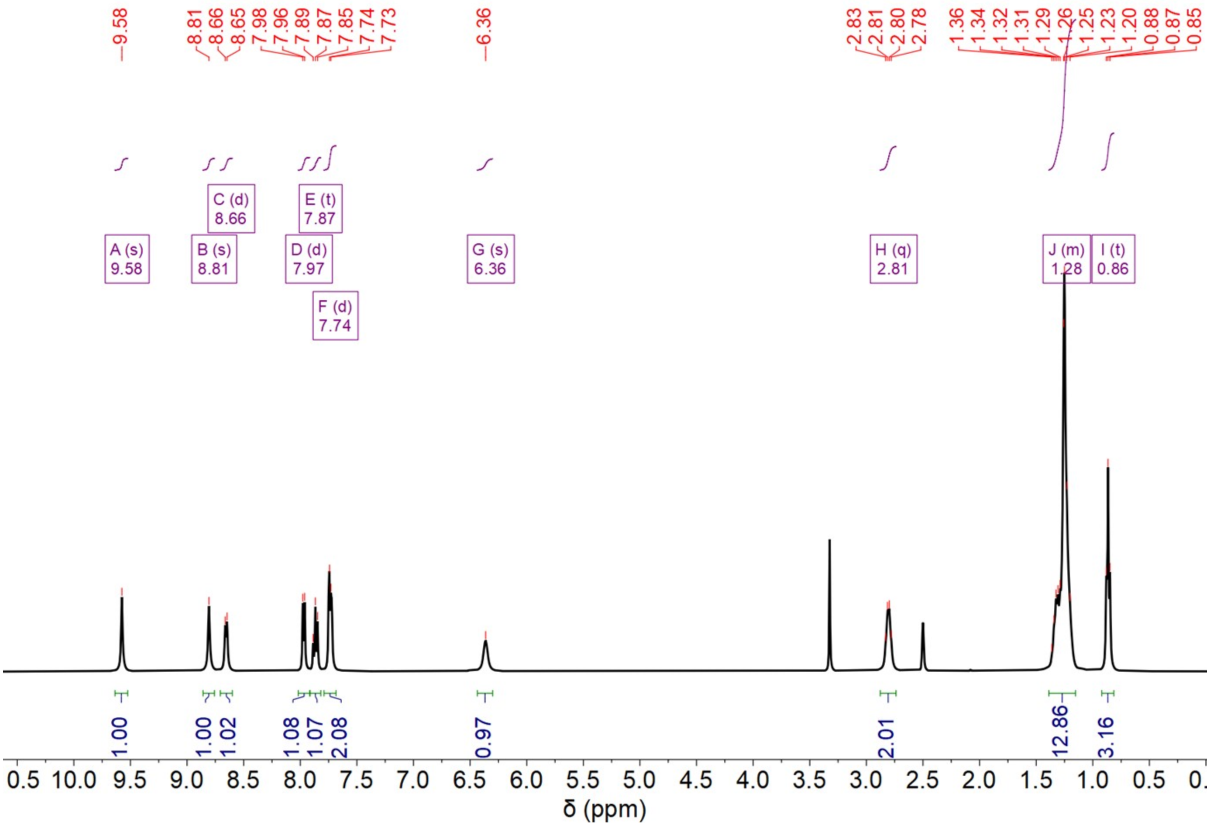

Figure S48. ^{1}H NMR (400 MHz, DMSO-d_6) spectrum of complex **7**.

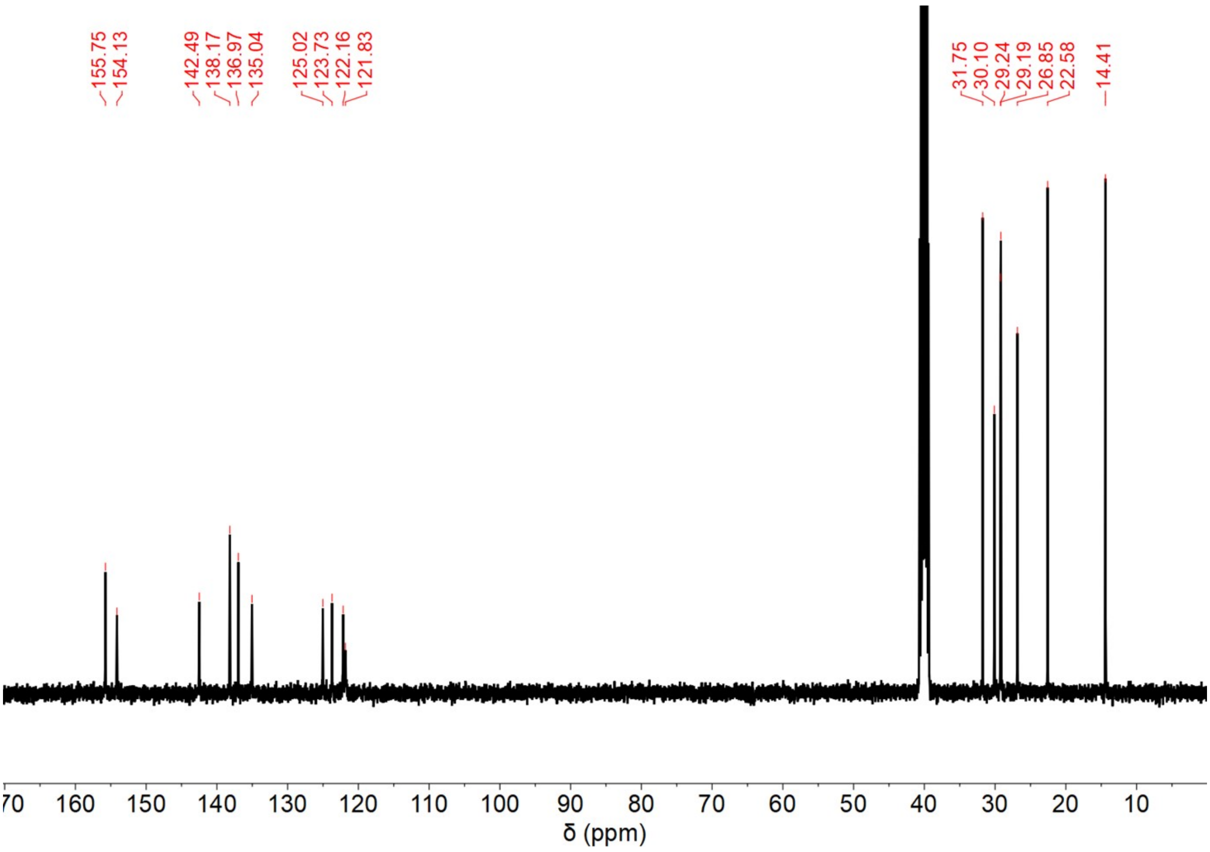

Figure S49. ^{13}C NMR (101 MHz, DMSO-d_6) spectrum of complex **7**.

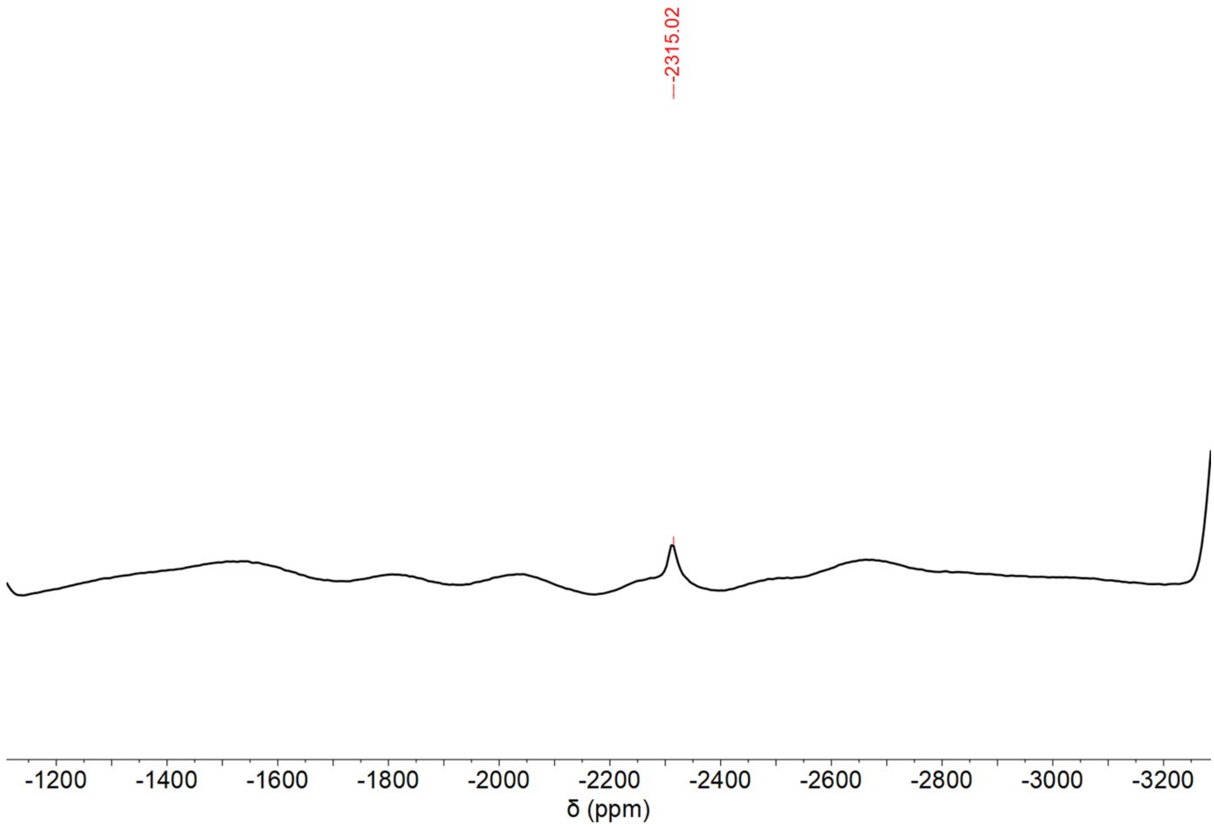

Figure S50. ^{195}Pt NMR (86 MHz, DMSO-d_6) spectrum of complex **7**.

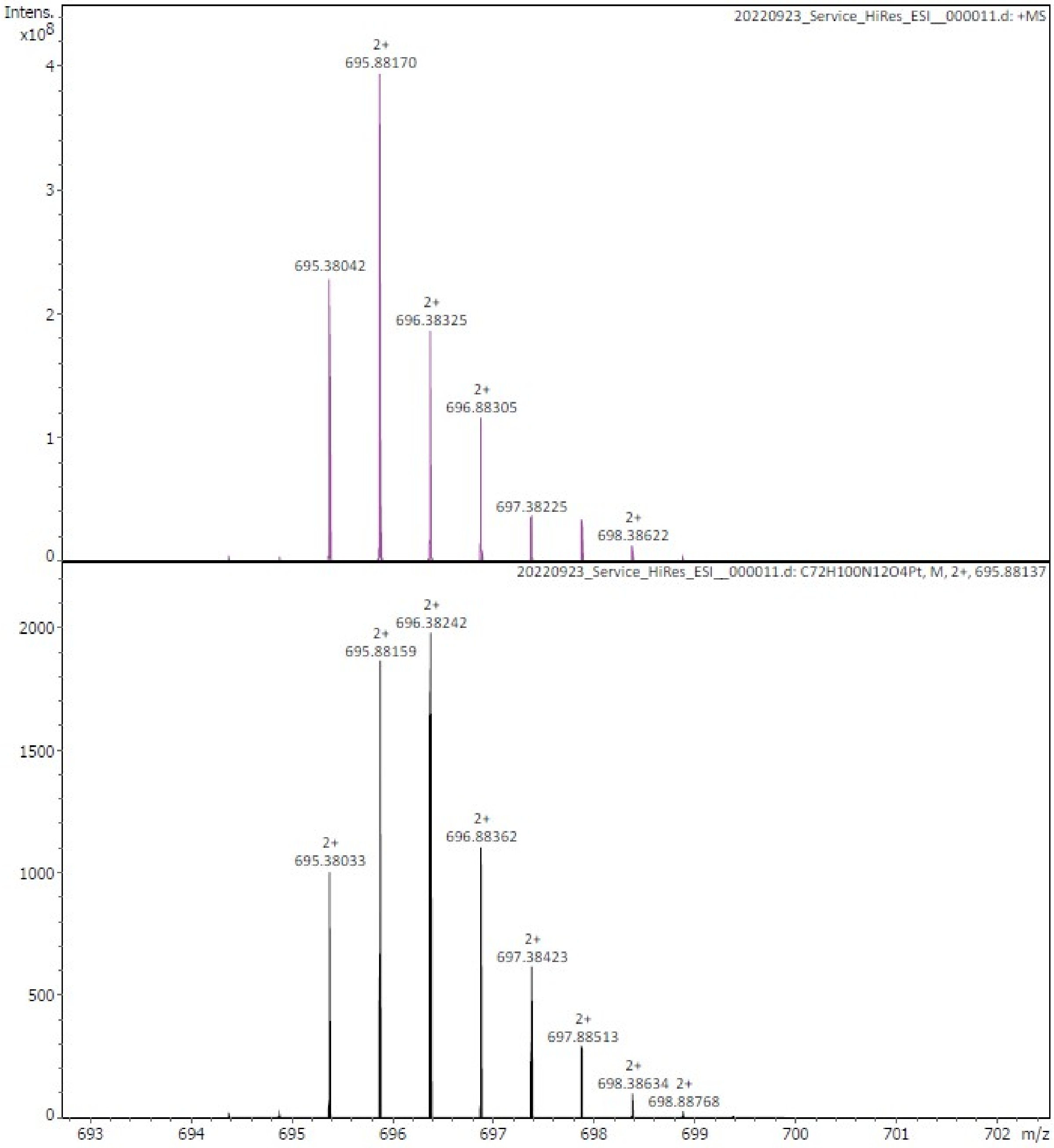

Figure S51. HR-MS (ESI+) of complex **7**.

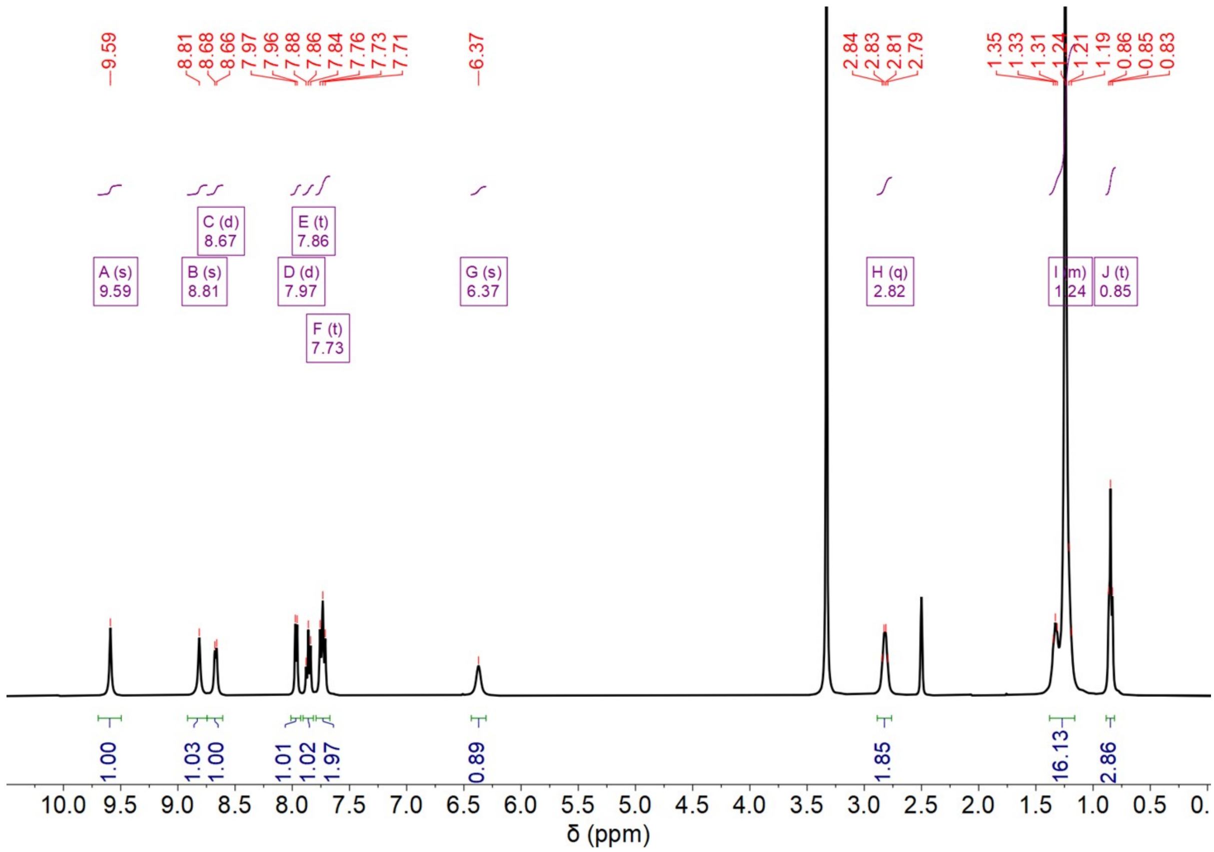

Figure S52. ^{1}H NMR (400 MHz, DMSO-d_6) spectrum of complex **8**.

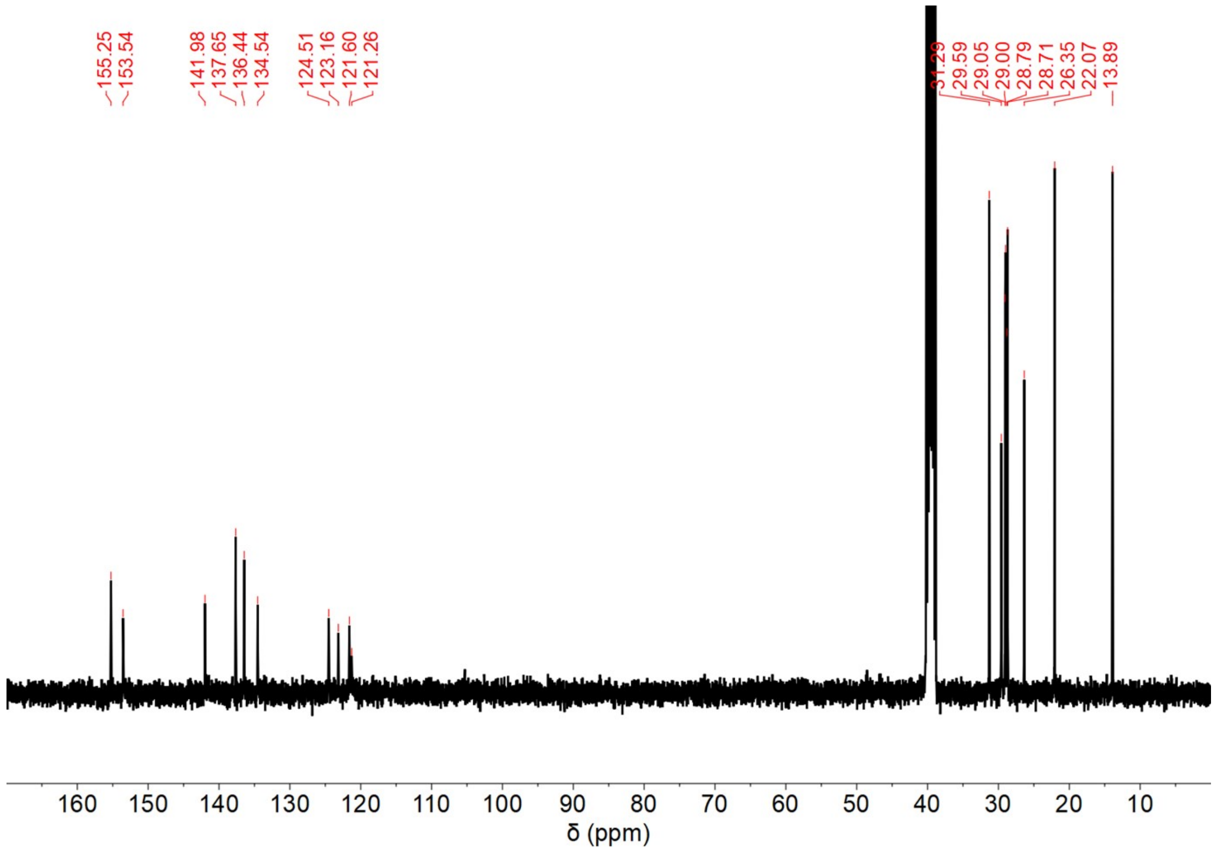

Figure S53. ^{13}C NMR (101 MHz, DMSO-d_6) spectrum of complex **8**.

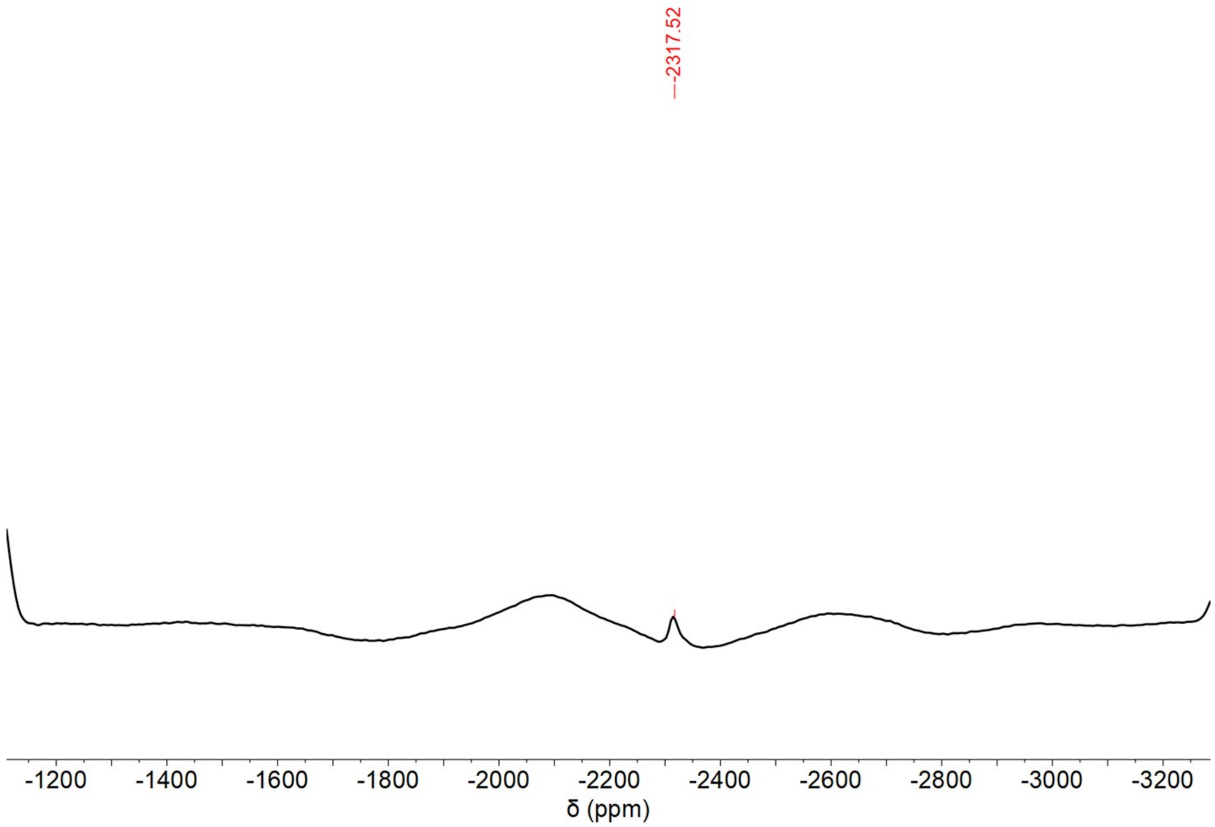

Figure S54. ^{195}Pt NMR (86 MHz, DMSO-d_6) spectrum of complex **8**.

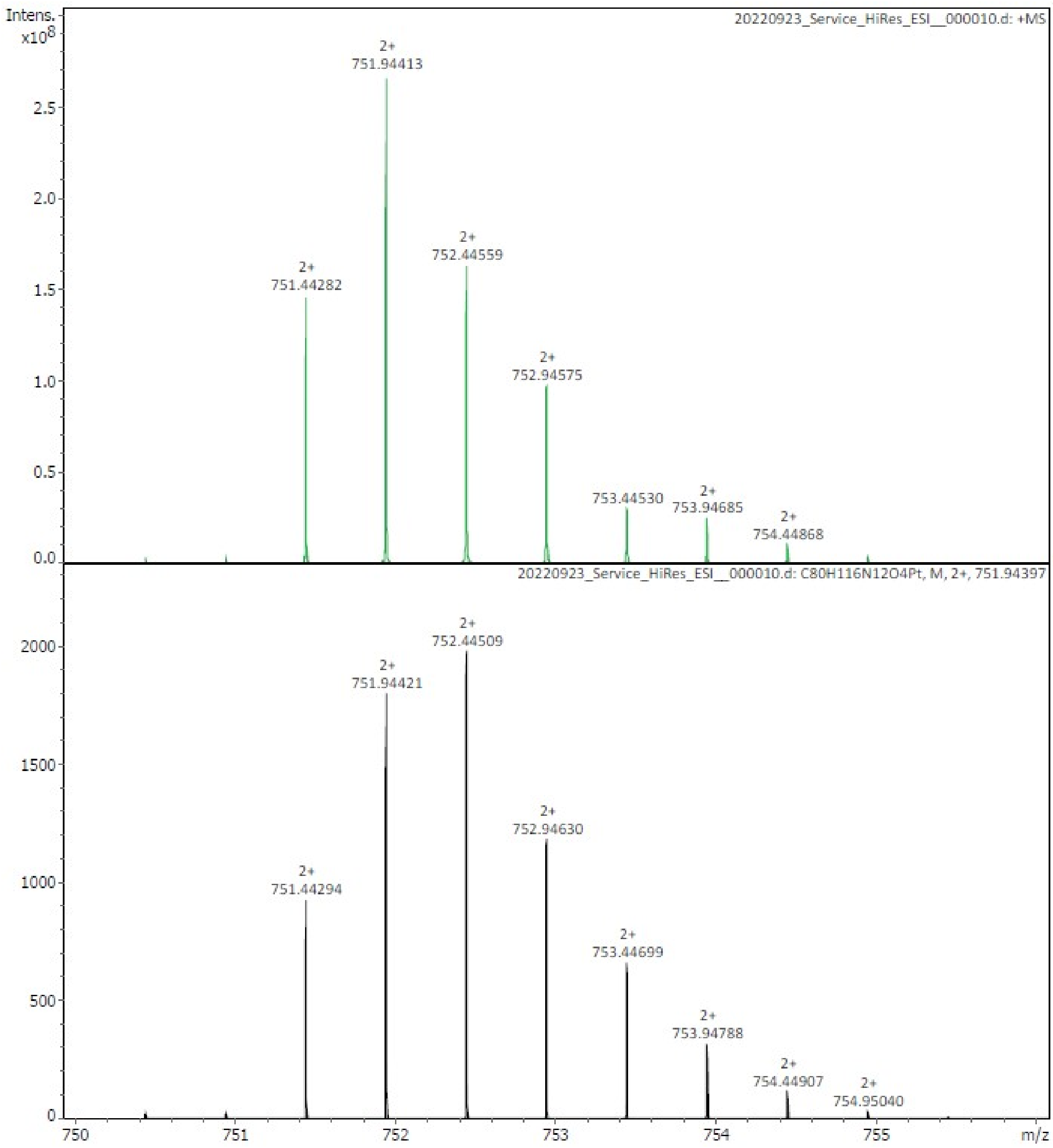

Figure S55. HR-MS (ESI+) of complex **8**.

Experimental procedures

Binding studies

^{1}H NMR Titrations

A 0.2 mM stock solution of the complex ('host') was prepared in DMSO-d_6/0.5 % H_2O, which was used to prepare the salt solutions. Tetrabutylammonium chloride (TBACl) was purchased commercially from Sigma Aldrich and dried for a minimum of 48 h in a desiccator under high vacuum before use.

A 0.03 M stock solution of TBACl was made in the host solution. The host solution (600 µL) was added into an NMR tube before adding 2 µL aliquots of the TBACl solution and collecting a spectrum for each titre. Titres were added from 0 – 13 equivalents of chloride.

The peak shifts of both urea N–H protons and the aromatic C–H proton in the 1 position were plotted against the chloride concentration and fit using BindFit v0.5 to a 1:2 binding model.[1]

Preferred binding models were calculated by performing a covariance of fit on the output data from Bindfit. The 1:2 binding was determined as the preferred binding for complexes **3**, **5**, and **7** based on the covariance of fit being at least 5 times smaller than the covariance for 1:1 binding. The covariance of fit ratio was ambiguous for complexes **1**, **2**, **4**, **6**, and **10** as they were less than 5. However, there is literature precedence for 1:2 binding in complex **4** and in similar Pt(II) complexes.[2]

DMSO Stability Studies

Solutions of complexes **1**–**8** were prepared in DMSO-d_6 at approx. 1 mM concentration before collecting ^{1}H NMR spectra of the samples at 0-, 1-, 2-, 3-, 7-, and 14-day timepoints.

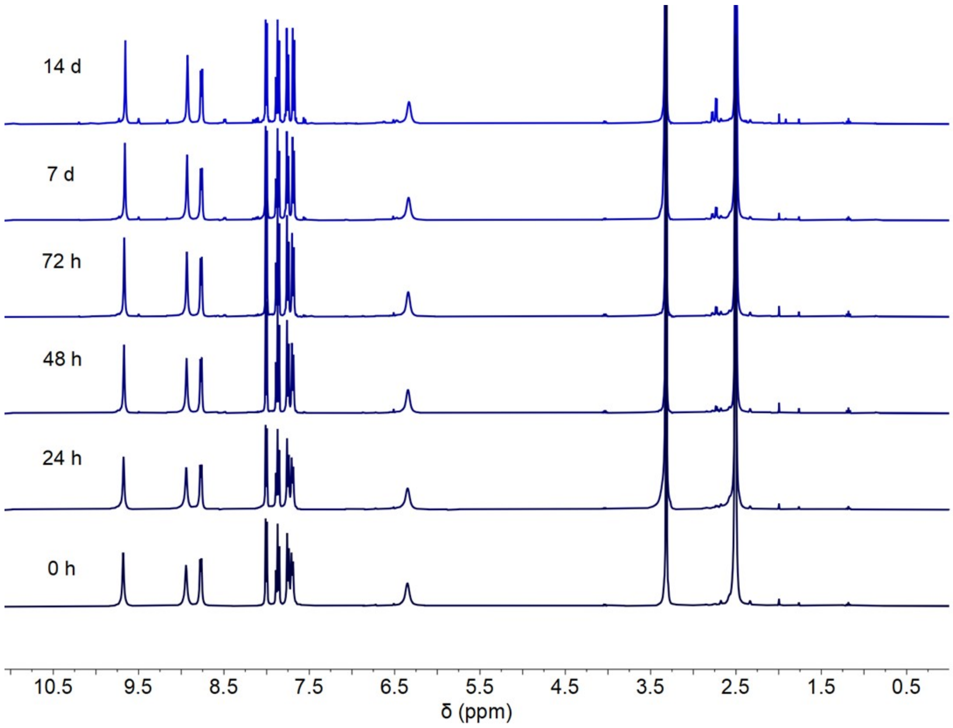

Figure S56. Stability of complex **1** in DMSO-d_6 over 14 days at room temperature.

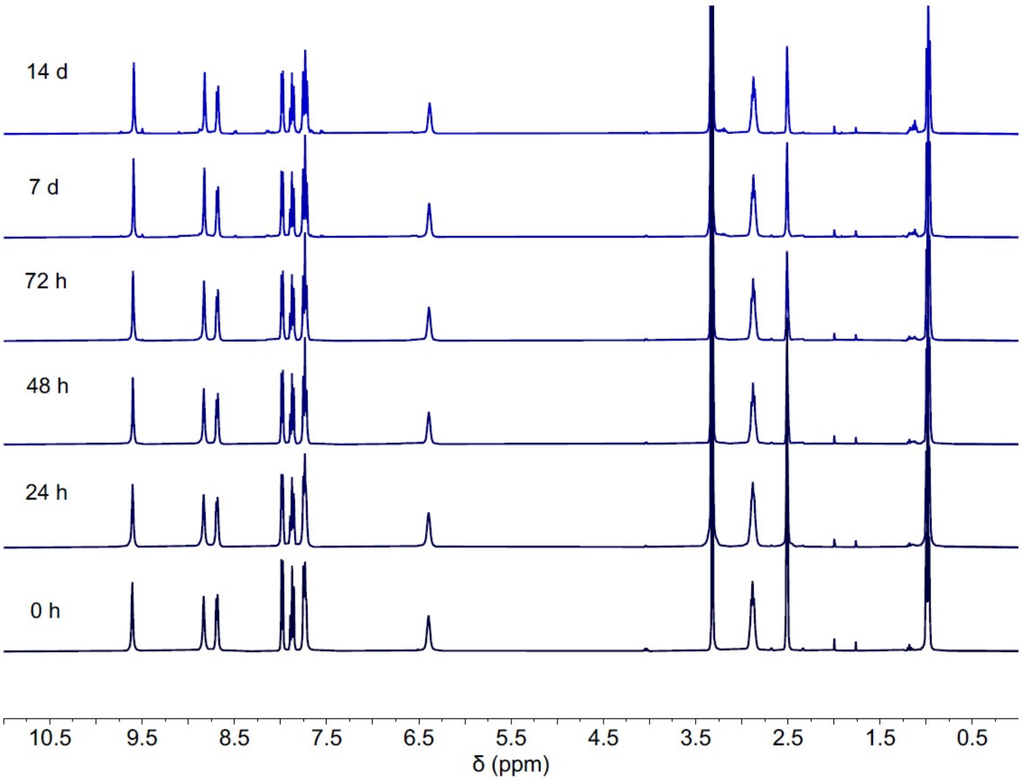

Figure S57. Stability of complex **2** in DMSO-d_6 over 14 days at room temperature.

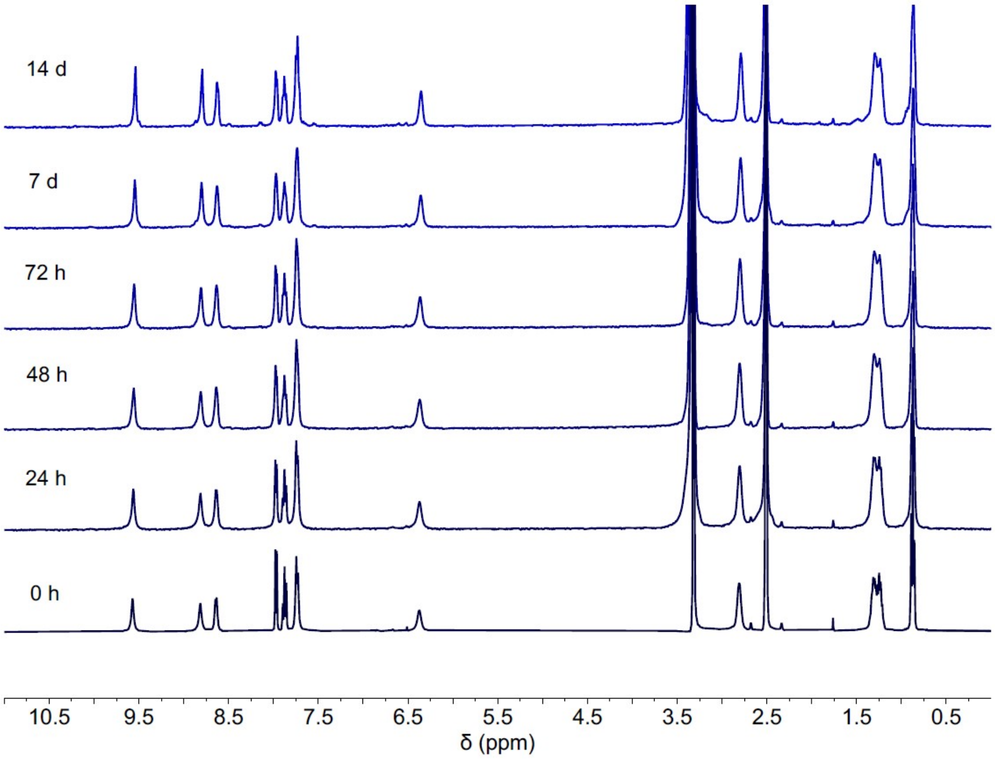

Figure S58. Stability of complex **4** in DMSO-d_6 over 14 days at room temperature.

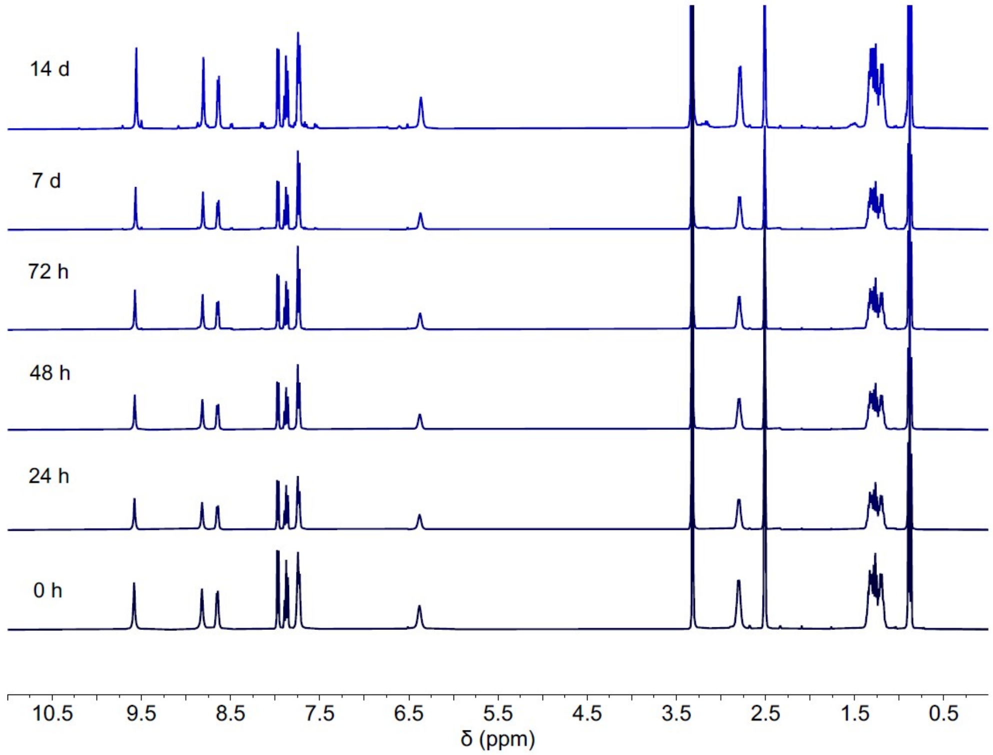

Figure S59. Stability of complex **5** in DMSO-d_6 over 14 days at room temperature.

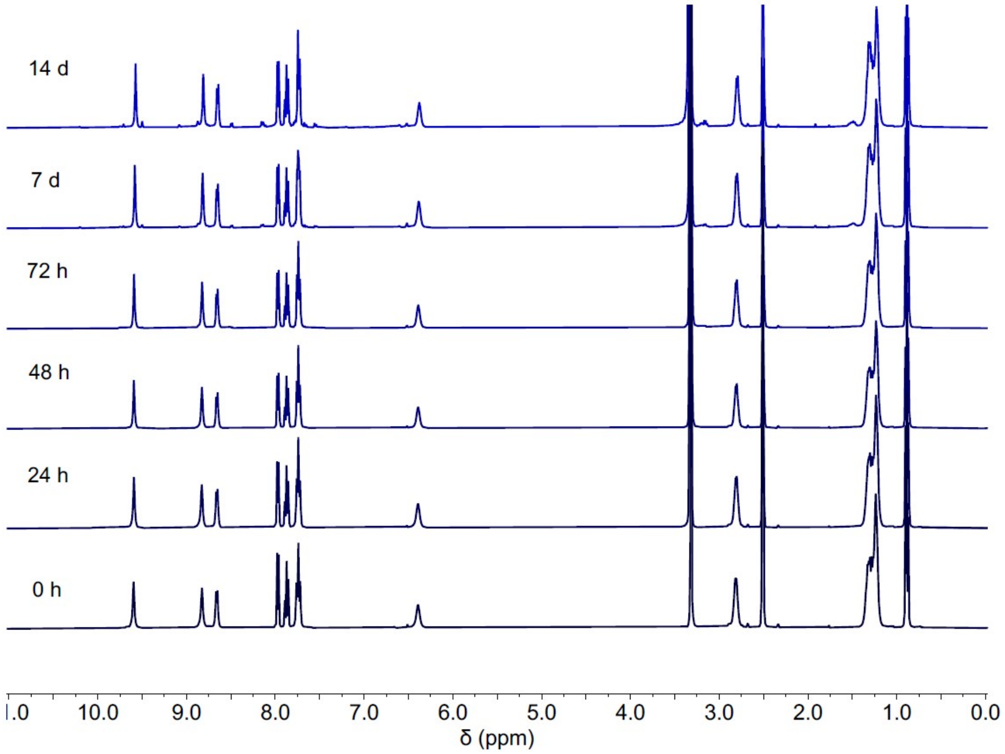

Figure S60. Stability of complex **6** in DMSO-d_6 over 14 days at room temperature.

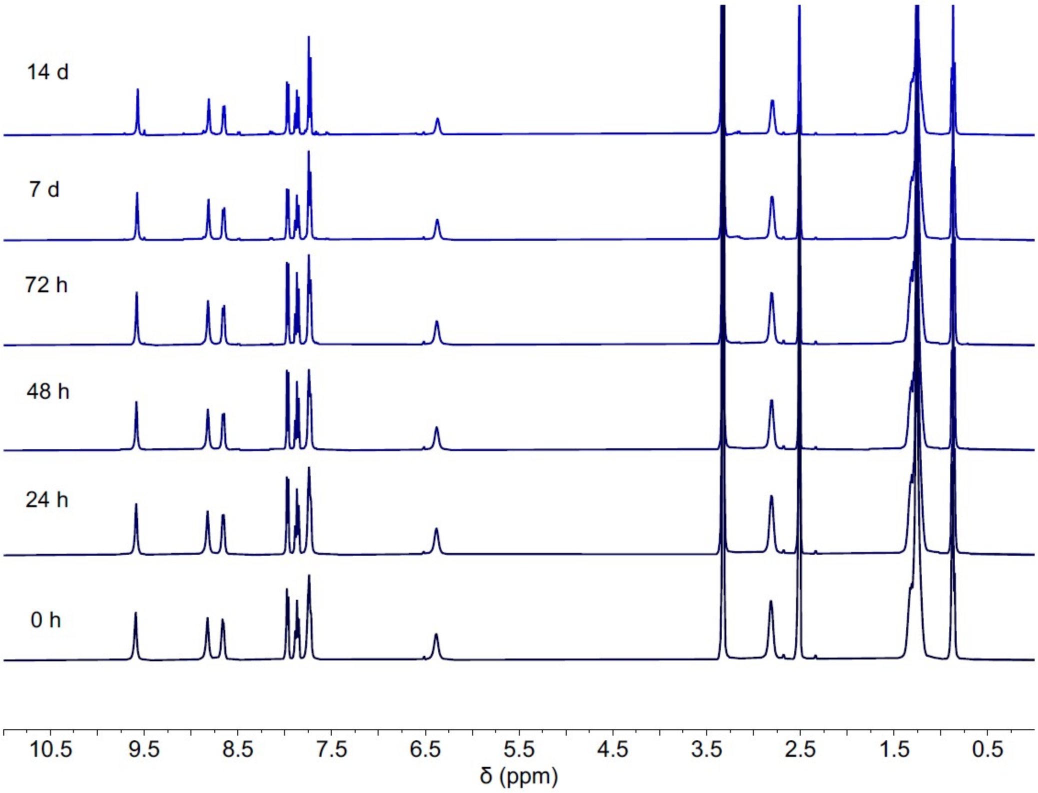

Figure S61. Stability of complex **7** in DMSO-d_6 over 14 days at room temperature.

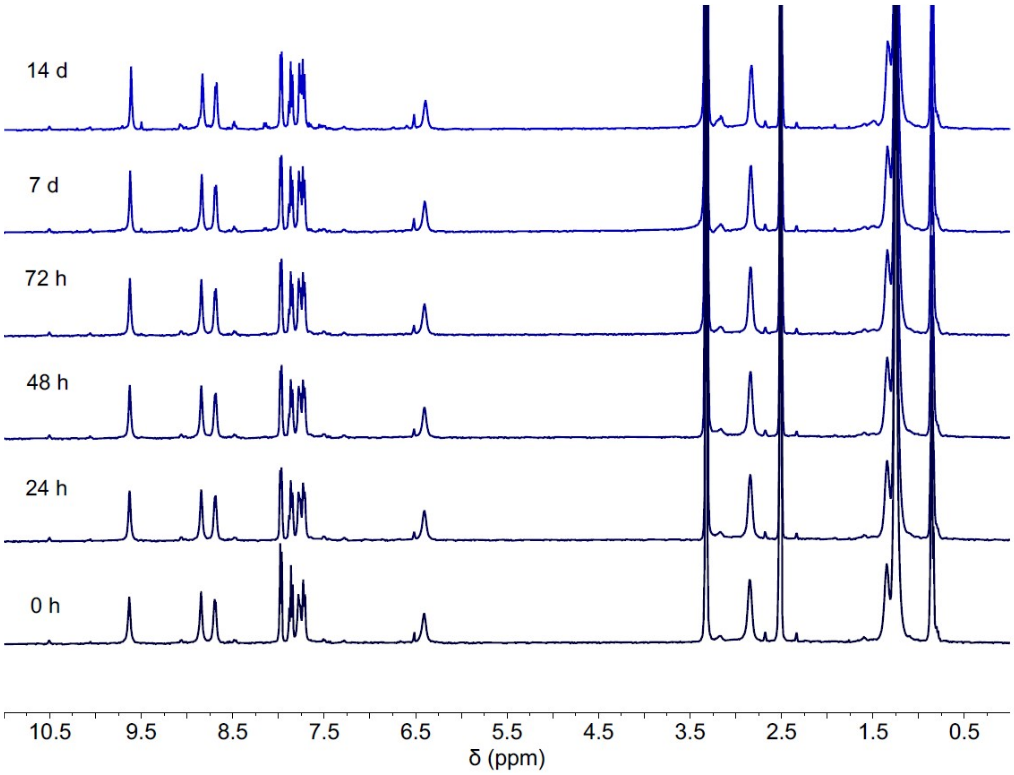

Figure S62. Stability of complex **8** in DMSO-d_6 over 14 days at room temperature.

Transport studies

Transport studies were performed using an Orion Chloride Selective electrode from ThermoFisher Scientific, which was calibrated using five NaCl stock solutions ranging from 1×10^{-5} M to 1×10^{-2} M.

General Vesicle Preparation

Vesicles were prepared according to the method outlined in 'Supramolecular methods: the chloride/nitrate transmembrane exchange assay'. [3] POPC lipids from Corden Pharma were dissolved in chloroform to make a 1 g in 35 mL solution. In a pre-weighed round bottom flask, 4 mL of the POPC solution was added and gently removed under reduced pressure, ensuring a smooth lipid cake. The lipids were dried in a desiccator under vacuum overnight before rehydration with 4 mL of internal solution. The lipid suspension was frozen in dry ice/acetone and thawed in water, repeating nine times. The suspension of lipids was extruded by passing through a 200 nm polycarbonate membrane 25 times. The external solution of the extruded vesicles was exchanged with the final external solution to be used for transport studies, either through dialysis or size-exclusion chromatography. The vesicles were then diluted to either a known concentration or to a known volume and the correct volume to add for each experiment was calculated.

ISE Cl^-/NO_3^- Exchange Assay

Vesicles were prepared with an internal solution of sodium chloride (487 mM) in a sodium phosphate buffer (5 mM) at pH 7.20. An external solution of sodium nitrate (487 mM) was made in the same phosphate salt buffer solution. Vesicles were dialysed in sodium nitrate solution overnight, before diluting to 10 mL with fresh sodium nitrate external solution.

For each run, vesicles were diluted to a final concentration of 1 mM in 5 mL external solution. The receptor was added at t = 0 s and data was collected for 300 s. At t = 300 s, 50 µL of Triton X-100 (10 % v/v in H_2O) detergent was added to lyse the vesicles and release any remaining internal chloride. At t = 420 s, data collection was stopped, and the final reading taken as 100 % efflux. Reported concentrations and error for each complex were repeated in duplicates.

Hill plot analysis was conducted on the results by plotting the percentage chloride efflux at t = 270 s against the receptor concentration in mol %. A derived equation (Equation S1) from the 'Hill 1' function in OriginPro 8.6 was used to calculate the EC_{50}.

$$EC_{50} = k * \left(\frac{50}{END - START - 50}\right)^{\frac{1}{n}}$$

Equation S1: Derived Hill function used to calculate EC_{50} values when chloride efflux did not reach 100 % over the duration of the ISE assay.

ISE Cl^-/SO_4^{2-} Exchange Assay

Vesicles were prepared using the same method as for the ISE Cl^-/NO_3^- exchange assay, except sodium sulfate was used for the external solution instead of sodium nitrate.

Cationophore Coupled Transport

Vesicles were prepared with an internal solution of potassium chloride (300 mM) in a buffer solution of potassium phosphate salts (5 mM), adjusted to pH 7.20. Two external solutions were made in the potassium phosphate buffer; potassium nitrate (333 mM) and potassium gluconate (300 mM), both adjusted to pH 7.20. After extrusion, vesicles were filtered through a column containing Sephadex G-25 Coarse, eluting with the potassium gluconate solution. The vesicles were collected and diluted to 10 mM with the same potassium gluconate solution. Cationophore-coupled transport for the complexes was tested at the calculated EC_{50} concentrations from the Cl^-/NO_3^- exchange assay.

For each experiment, 0.5 mL of vesicles were added to 4.5 mL of either potassium nitrate or potassium gluconate external solution. 10 µL of a cationophore (valinomycin or monensin) in DMSO was added to a final concentration of 0.1 mol % at t = −30 s. 100 µL of the receptor in DMSO was added at t = 0 s, before collecting data for 300 s. At t = 300 s, 50 µL of Triton X-100 (10 % v/v in H_2O) detergent was added to lyse the vesicles and release any remaining internal chloride. At t = 420 s, data collection was stopped, and the final reading taken as 100 % efflux. Experiments were performed in duplicate.

HPTS Assay

An external solution of potassium chloride (100 mM) and HEPES (5 mM) was made, adjusted to pH 7.00. Vesicles were prepared with an internal solution containing 1 mM HPTS dissolved in the external solution. After extrusion, vesicles were filtered through a column containing Sephadex G-25, eluting with the potassium chloride external solution. The vesicles were

collected and diluted to 10 mL with fresh external solution. For each experiment, HPTS vesicles were diluted to 0.1 mM in a final volume of 2.5 mL external solution in a cuvette. Experiments were performed in triplicates simultaneously.

Every addition of solution into the lipids were staggered by 10 s between each cuvette. At the start of each run, a pH gradient was induced in each cuvette by adding 25 µL of 0.5 M sodium hydroxide. After 30 s, the transporter was added as a 5 µL DMSO solution before collecting data for 200 s. At t = 200 s, 25 µL Triton X-100 (10 % v/v in H_2O) was added before stopping at t = 240 s, with the final reading taken as 100 % proton efflux.

Ionophore-coupled studies required the further addition of either valinomycin or carbonyl cyanide *m*-chlorophenyl hydrazone (CCCP) to a final concentration of 0.1 or 1 mol % respectively. The cationophores were added prior to the base pulse and allowed to evenly distribute throughout the lipids first. For fatty acid-free conditions, 1 mol % bovine serum albumin (BSA) was incorporated into the HPTS vesicles prior to dilution in cuvettes. Transporters were added to the lipids at a concentration corresponding to approximately 50 % efflux.

Proton efflux was studied by using an HPTS ratiometric fluorescent probe, detecting the emission of HPTS at 510 nm. As HPTS is fluorescent in its acidic and basic form, a ratio (*R*) can be interpreted by exciting the vesicles at 403 nm and 460 nm respectively. Fractional fluorescence intensity (I_F) can then be calculated using the following equation:

$$I_F = \frac{R_t - R_0}{R_d - R_0}$$

Equation S2: HPTS fractional fluorescence intensity (I_F) based on fluorescence ratios at the start (R_0), end (R_d), and any chosen time point (R_t).

Data analysis was conducted via Hill analysis on OriginPro 8.6, plotting I_F against the receptor concentration in mol %. A modified Hill equation was used for this analysis:

$$y = y_0 + (y_{max} - y_0) * \frac{x^n}{k^n + x^n}$$

Equation S3: Modified Hill equation used to analyse the data from the HPTS assay.

Where y_{max} is the maximum value of I_F, y_0 is the value at t = 200 s for a DMSO blank experiment, k is the EC_{50} concentration, and n is the Hill coefficient.

HPTS Oleic Acid Assay

This assay was set up identically to the regular HPTS assay, with a key difference of using POPC lipids purchased from Avanti Polar Lipids instead of Corden Pharma. Oleic acid was added to the lipids to a final concentration of either 2 mol% or 4 mol% prior to the start of each run. Following a base pulse, transporters were then added as a DMSO solution at a concentration corresponding to approximately 50 % efflux.

Anion Selectivity Assay

Vesicles were prepared with an internal solution of NaCl (300 mM) and HPTS (1 mM) in a buffer solution of HEPES (10 mM), adjusted to pH 7.00. Five external solutions were made in an isotonic buffer, containing NaCl, NaBr, $NaNO_3$, NaI, or $NaClO_4$. After extrusion, vesicles were filtered through a Sephadex G-25 Coarse column, eluting with NaCl. Anion exchange was induced by adding 5 µL of the transporter in DMSO to the solution. Anion exchange was continued for as long as required for all anions to reach a plateau in activity. Note that NaI contains iodine impurities which may act as transporter for I^-. Conversion of I_F values were done according to the published method.[4]

Cell Studies

Cell Culture

The MCF-7 breast adenocarcinoma cell line was purchased from Sigma Aldrich (86012803-1VL; Passage number 13; NSW, Australia), while the MDAMB231 triple-negative breast adenocarcinoma and AGS gastric adenocarcinoma cell lines were obtained from American Type Culture Collection (In vitro Technologies, NSW, Australia). The MCF7 and MDAMB231 cells were grown in high-glucose Dulbecco's modified Eagle's medium (DMEM with 4.5 g/L glucose; Lonza, NSW, Australia) with 10% fetal bovine serum (FBS; Bio-Strategy PTY, Campbellfield, VIC, Australia), and supplemented with 1% penicillin and streptomycin (Sigma-Aldrich, NSW, Australia). The MCF-7 and MDA-MB-231 cells were maintained at 37 °C in a 5% controlled CO_2 atmosphere and were sub-cultured every 48–72 h until they formed a confluent monolayer. The AGS cells were grown in ATCC-formulated F-12K medium with 10% fetal bovine serum (Bio-Strategy PTY, Campbellfield, VIC, Australia), and supplemented with 1% penicillin and streptomycin (Sigma-Aldrich, NSW, Australia) under

the same growth conditions as the MCF-7 and MDA-MB-231 cells. Cell maintenance was performed every 48–72 h, the time necessary for cells to achieve confluent monolayers.[5]

Alamar Blue Assay

The cells (100 µL) were cultured in 96 well-plates at a seeding density of 1 × 10^5 cells/mL. After 24 h, the cells were treated with the samples (dissolved in DMSO) and incubated for 72 h. The standard chemotherapeutic drug doxorubicin (1 µM) was used as the positive control, while a negative control with 0.1% DMSO was added to each plate. At the end of the incubation period, the culture media were aspirated followed by the addition of 100 µL of 0.1 mg/mL Alamar blue solution (stock solution of 1 mg/mL freshly prepared in PBS followed by 1:10 dilution with FBS-free media) to each well. After 4 h of incubation, the fluorescence intensity was measured with excitation wavelength at 555 nm and emission wavelength at 595 nm using a BMG LABTech FLUOstar OPTIMA plate reader (BMG Labtech, VIC, Australia). The drugs were tested in triplicate, with the negative control taken as 100% cell viability. Non-linear regression and IC_{50} calculations were performed using GraphPad Prism 9.0 (GraphPad Inc., San Diego, CA).[6]

Flow cytometric analysis of apoptosis in the AGS gastric cancer cells using the most active complexes 2–4.

Flow cytometry was used to evaluate the apoptosis in the AGS cells after treatment with the most active complexes **2**, **3**, and **4** as previously reported using an annexin V and 7-AAD-based kit (#ab214663, Abcam, Melbourne, VIC, Australia). AGS cells were grown in T75 cell culture flasks, starting with an initial density of 1 × 10^6 cells in 10 mL of culture medium. The cells were maintained at 37 °C in an environment with 5% CO_2 for 24 h. On the subsequent day, the culture medium was aspirated from each flask, and the cells were exposed to 10 µg/mL of complexes **2**, **3**, and **4**, while a serum-containing medium was used as the untreated control. The flasks were then returned to the incubator at 37 °C with 5% CO_2 for an additional 24 h. Following a 24 h incubation, the cell culture media were harvested from each flask. Subsequently, trypsin (0.25% w/v) was added to the flasks for a 3 min incubation at 37 °C. The trypsin reaction was then neutralized with an equal volume of 10% FBS-containing medium, and the cells were combined with the previously collected media. The cells were pelleted by centrifugation at 500×g for 5 min at room temperature. This process was repeated twice, with the cell pellets being resuspended in 1 mL of PBS on both occasions. The resulting cell pellets

from each treatment were suspended in 500 µL of 1X binding buffer and gently mixed by pipetting.

Exactly 5 µL of Annexin V-CF blue and 7-aminoactinomycin D (7-AAD) staining solutions were added to 100 µL of cell suspension. The stained cells were incubated in the dark at room temperature for 15 min, then 400 µL of a 1X assay buffer was introduced to each cell suspension. Subsequently, the cells were analysed using a flow cytometer (Novocyte 3000, ACEA Biosciences Inc, CA, USA), and data were analyzed and processed using NovoExpress software (version 1.5.0, ACEA Biosciences Inc, CA, USA). Initially, the cells were gated on forward and side scatter modes to remove cell aggregates and debris located close to the origin. The cells were analyzed using dot plots, where Annexin V-CF in Pacific Blue was plotted against 7-AAD fluorescence in PerCP. Quadrants were established in relation to the untreated control, with live cells (+Annexin V and −7-AAD) found in the lower-left quadrant, early apoptotic cells (+Annexin V and −7-AAD) in the lower-right quadrant, late apoptotic cells (+Annexin V and +7-AAD) in the upper-right quadrant, and necrotic cells (−Annexin V and +7-AAD) in the upper-left quadrant. For statistical analysis and visualization, the percentage of cells in each quadrant after different treatments (n = 3) was exported to GraphPad Prism software (version 9.0, CA, USA).

Reactive Oxygen Species (ROS) Assay

The effect of the most active complexes **2**, **3**, and **4** on the oxidative stress of the AGS cancer cells was studied using the H2DCFDA (2′,7′-dichlorofluorescein diacetate) cellular reactive oxygen species (ROS) Detection Assay Kit (#ab113851; Abcam, VIC, Australia) [7]. H2DCFDA is a fluorescent dye employed for the evaluation of cellular concentrations of hydroxyl, peroxyl, and diverse other reactive oxygen species (ROS) activity.[7]

The AGS cells were plated at a density of 2.5×10^5 cells per mL in a 96-well plate and allowed to adhere overnight at 37 °C with 5% CO_2. The cell culture medium was aspirated the following day, and each well was gently agitated while adding 100 µL of 1x buffer. Afterwards, the buffer was removed, and 100 µL of a 20 µM H2DCFDA solution was added to each well, followed by a 45 min incubation at 37 °C in the absence of light. The H2DCFDA solution was then removed, and the cells were washed once more with 100 µL of 1x buffer, with gentle manual agitation of the plate. Subsequently, the cells were exposed to 1 µM of doxorubicin and 250 µM of tert-butyl hydroperoxide (TBHP) then incubated at 37 °C for 4 h. Finally, the plate was read at

Ex/Em = 485/535 nm using a microplate spectrophotometer (BMG CLARIOstar, VIC, Australia). The fold increase in ROS production was calculated relative to the untreated control (cells treated with the supplement buffer following the specified procedure).

Chloride Depletion Studies

HEPES-buffered solutions were prepared with the following compositions: 120 mM NaCl, 5 mM KCl, 1 mM $MgCl_2$, 1 mM $CaCl_2$, 10 mM D-glucose, 10 mM HEPES (pH 7.4), and 25 mM $NaHCO_3$. To prepare Cl^- free HEPES-buffered solutions, Cl^- ions in buffer solutions were replaced with equimolar concentrations of gluconate salts. AGS cells were incubated for 18 hours with different concentrations of **2**, **3**, **4**, or cisplatin in HEPES-buffered solutions or Cl^- free HEPES-buffered solutions at 37 °C under a humidified atmosphere of 5% CO_2. Alamar Blue assay was conducted using standard procedures. The fluorescence levels were measured (excitation wavelength of 555 nm and emission at 595 nm) with a microplate spectrophotometer (BMG CLARIOstar, Mornington, VIC, Australia).

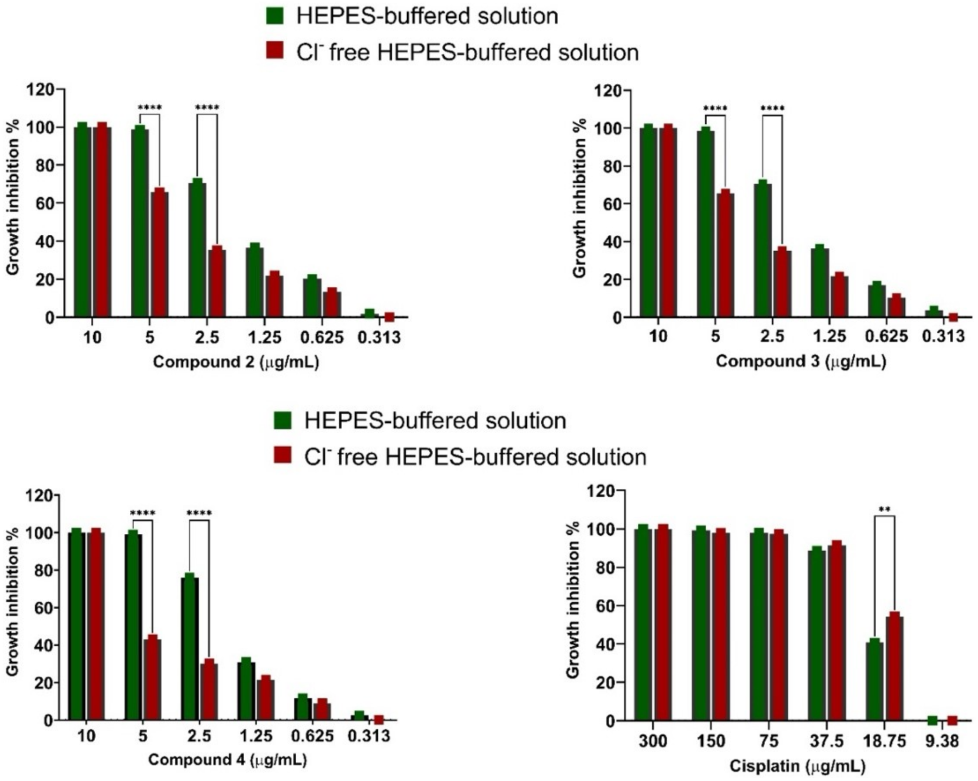

Figure S63. AGS gastric cancer cells were separately incubated with various concentrations of compounds **2**, **3**, **4** and cisplatin in HEPES-buffered solution and Cl^- free HEPES-buffered solution for 18 h. Cell death was measured by using an Alamar Blue assay (mean, n = 3). **indicates $P \leq 0.01$ and **** indicates $P \leq 0.0001$).

Crystallography

Single crystals of complexes **2**, **3**, and **5** were obtained by slow diffusion of diethyl ether into a solution of the complex in DMF. Single crystals of **6** and **7** were obtained from initial hot filtrations with MeCN during the synthesis. A suitable crystal was selected and in Paratone on a micromount on a SuperNova, Dual, Cu at home/near, Atlas diffractometer. The crystal was kept at 100 K during data collection. Using Olex2,[8] the structure was solved with the ShelXS[9] structure solution program using Direct Methods and refined with the ShelXL[10] refinement package using Least Squares minimization.

For complex **7**, X-ray diffraction data were collected at 100 K on the MX2 Macromolecular Crystallography beamline at the Australian Synchrotron.[11] The data collection and integration were performed within the Blu-Ice[11] and XDS[12] software programs. Structure solutions were obtained by intrinsic phasing methods from SHELXT[13] and were refined by a full-matrix least-squares on all unique F^2 values using SHELXL[14] as implemented within OLEX2-1.5.[9] All non-hydrogen atoms were refined with anisotropic thermal parameters, with hydrogen atoms being added geometrically and refined using riding thermal parameters.

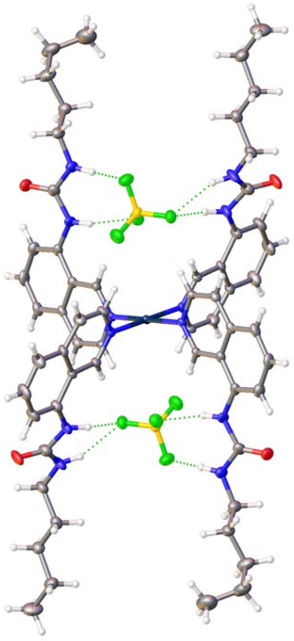

Figure S64. Olex2 depiction of complex **5** with thermal ellipsoids shown at the 50% level.

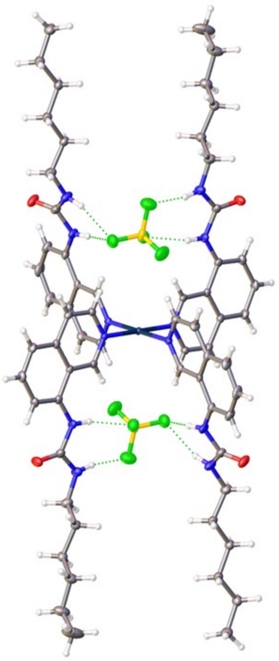

Figure S65. Olex2 depiction of complex **6** with thermal ellipsoids shown at the 50% level.

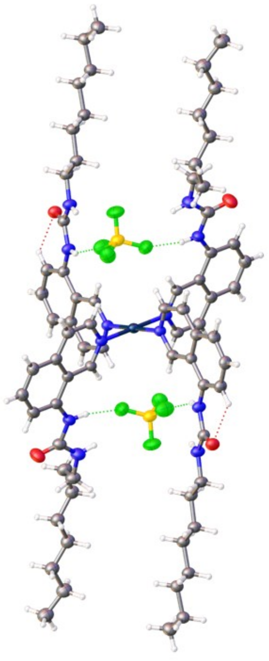

Figure S66. Olex2 depiction of complex **7** with thermal ellipsoids shown at the 50% level.

Table S1. Crystal and data refinement parameters for the X-ray crystallography studies.

Crystallographic details	Complex 2	Complex 3	Complex 5	Complex 6	Complex 7
Empirical formula	$C_{48}H_{52}B_2F_8N_{12}O_4$ Pt	$C_{52}H_{60}B_2F_8N_{12}O_4$ Pt	$C_{60}H_{76}B_2F_8N_{12}O_4$ Pt	$C_{64}H_{84}B_2F_8N_{12}O_4$Pt	$C_{72}H_{100}B_2F_8N_{12}O_4$ Pt
Formula weight	1229.72	1285.83	1398.03	1454.14	1566.34
Temperature/K	100(2)	100(2)	100(2)	100.00	100(2)
Crystal system	monoclinic	monoclinic	monoclinic	monoclinic	triclinic
Space group	$P2_1/c$	$P2_1/c$	$P2_1/c$	$P2_1/c$	P-1
a/Å	11.4301(13)	12.1096(12)	13.3731(8)	13.203(3)	11.013(2)
b/Å	19.8030(14)	19.8229(13)	14.0167(7)	14.387(3)	13.708(3)
c/Å	11.2570(9)	11.4298(12)	17.0490(7)	17.238(4)	14.075(3)
α/°	90	90	90	90	114.46(3)
β/°	103.529(10)	106.062(11)	103.604(5)	93.431(6)	99.20(3)
γ/°	90	90	90	90	104.70(3)
Volume/Å^3	2477.3(4)	2636.6(4)	3106.1(3)	3268.6(13)	1784.1(8)
Z	2	2	2	2	1
ρ_{calc}g/cm^3	1.649	1.620	1.495	1.477	1.458
μ/mm^{-1}	2.920	2.748	2.339	2.226	2.045
F(000)	1232.0	1296.0	1424.0	1488.0	808.0
Crystal size/mm^3	0.05 × 0.03 × 0.01	0.121 × 0.072 × 0.031	0.16 × 0.07 × 0.05	0.9 × 0.27 × 0.195	0.05 × 0.01 × 0.01
Radiation	Mo Kα (λ = 0.71073)	Mo Kα (λ = 0.71073)	Mo Kα (λ = 0.71073)	Mo Kα (λ = 0.71073)	synchrotron (λ = 0.71073)
2Θ range for data collection/°	3.664 to 50.048	4.058 to 50.05	3.806 to 52.74	3.69 to 54.97	3.334 to 57.396
Index ranges	-13 ≤ h ≤ 13, -23 ≤ k ≤ 23, -13 ≤ l ≤ 13	-14 ≤ h ≤ 14, -23 ≤ k ≤ 23, -13 ≤ l ≤ 13	-16 ≤ h ≤ 16, -17 ≤ k ≤ 17, -21 ≤ l ≤ 21	-17 ≤ h ≤ 17, -18 ≤ k ≤ 18, -22 ≤ l ≤ 22	-14 ≤ h ≤ 14, -16 ≤ k ≤ 16, -19 ≤ l ≤ 19
Reflections collected	37769	39122	36390	71595	29373
Independent reflections	4374 [R_{int} = 0.2077, R_{sigma} = 0.1094]	4655 [R_{int} = 0.2593, R_{sigma} = 0.1535]	6356 [R_{int} = 0.0758, R_{sigma} = 0.0591]	7499 [R_{int} = 0.0584, R_{sigma} = 0.0315]	8368 [R_{int} = 0.0279, R_{sigma} = 0.0253]
Data/restraints/parameters	4374/144/354	4655/6/372	6356/0/396	7499/0/426	8368/0/450
Goodness-of-fit on F^2	1.003	0.989	1.004	1.074	1.100
Final R indexes [I>=2σ (I)]	R_1 = 0.0540, wR_2 = 0.1139	R_1 = 0.0660, wR_2 = 0.1315	R_1 = 0.0292, wR_2 = 0.0504	R_1 = 0.0242, wR_2 = 0.0560	R_1 = 0.0283, wR_2 = 0.0727
Final R indexes [all data]	R_1 = 0.0970, wR_2 = 0.1342	R_1 = 0.1254, wR_2 = 0.1601	R_1 = 0.0558, wR_2 = 0.0567	R_1 = 0.0318, wR_2 = 0.0594	R_1 = 0.0283, wR_2 = 0.0728
Largest diff. peak/hole / e Å^{-3}	1.66/-2.61	1.42/-1.94	0.76/-0.60	1.04/-0.65	0.70/-1.67

Binding Data and Fittings

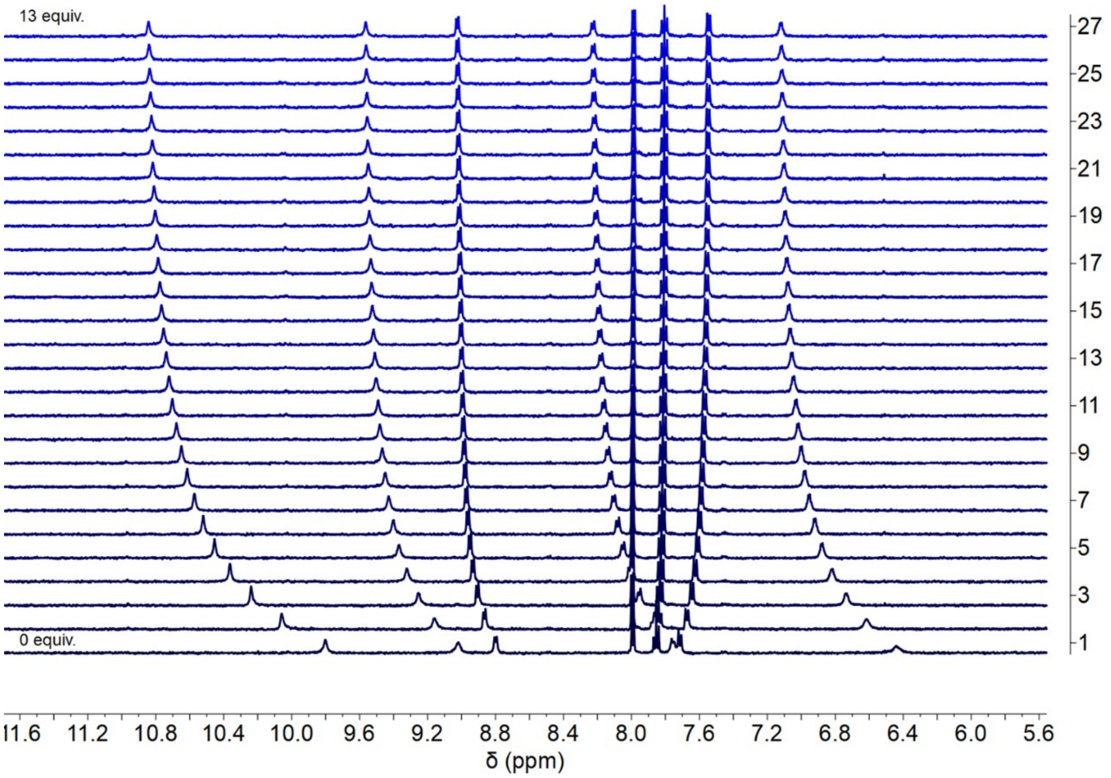

Figure S67. Stack plot of ^{1}H NMR titration of complex **1** with TBA-Cl from 0 – 13 equiv. in DMSO-d_6/0.5 % H_2O at 298 K. Concentrations are normalised against dilution factors.

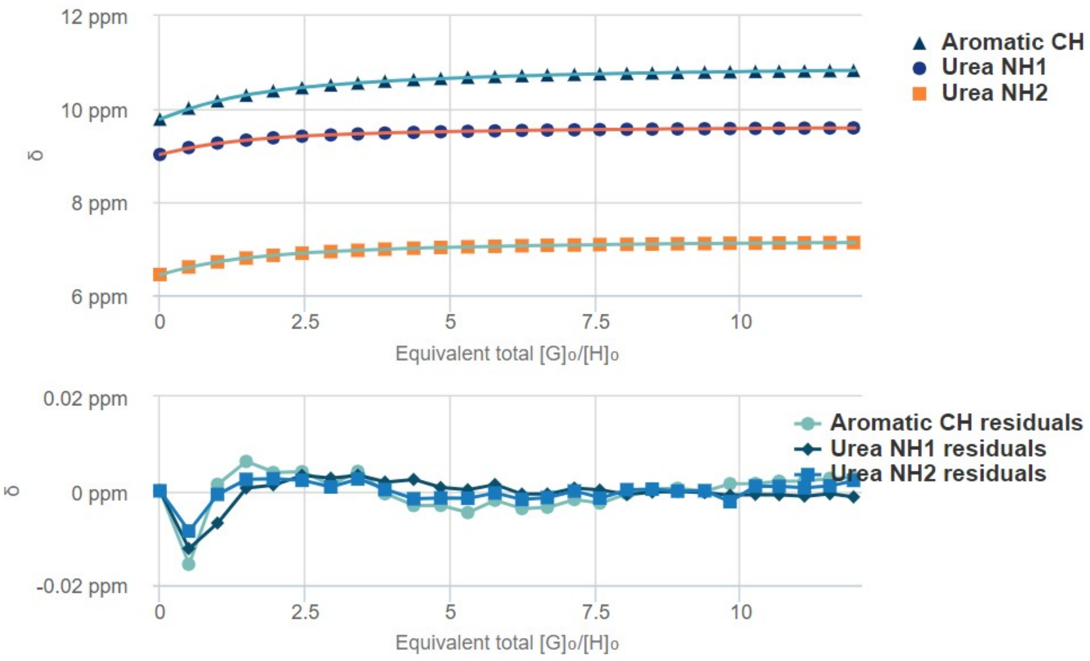

Figure S68. Proton shifts and residuals of complex **1** fitted to a 1:2 model using BindFit. Available at http://app.supramolecular.org/bindfit/view/c00453d6-80ce-4834-81bf-5f716df175a9

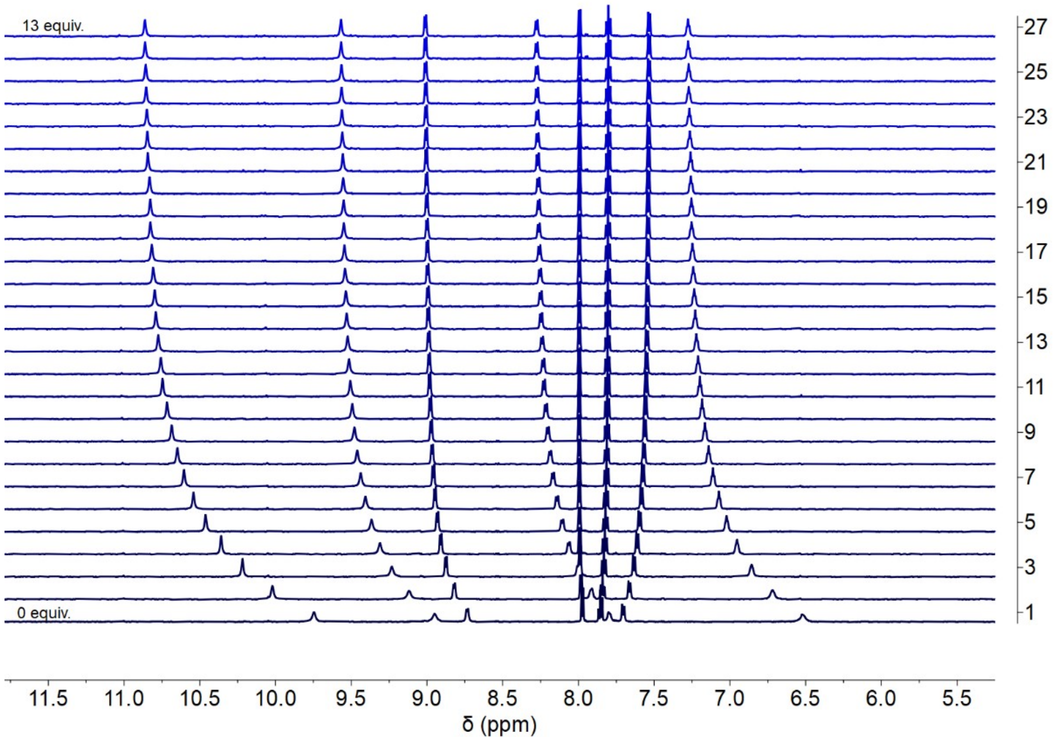

Figure S69. Stack plot of ^{1}H NMR titration of complex **2** with TBA-Cl from 0 – 13 equiv. in DMSO-d_6/0.5 % H_2O at 298 K. Concentrations are normalised against dilution factors.

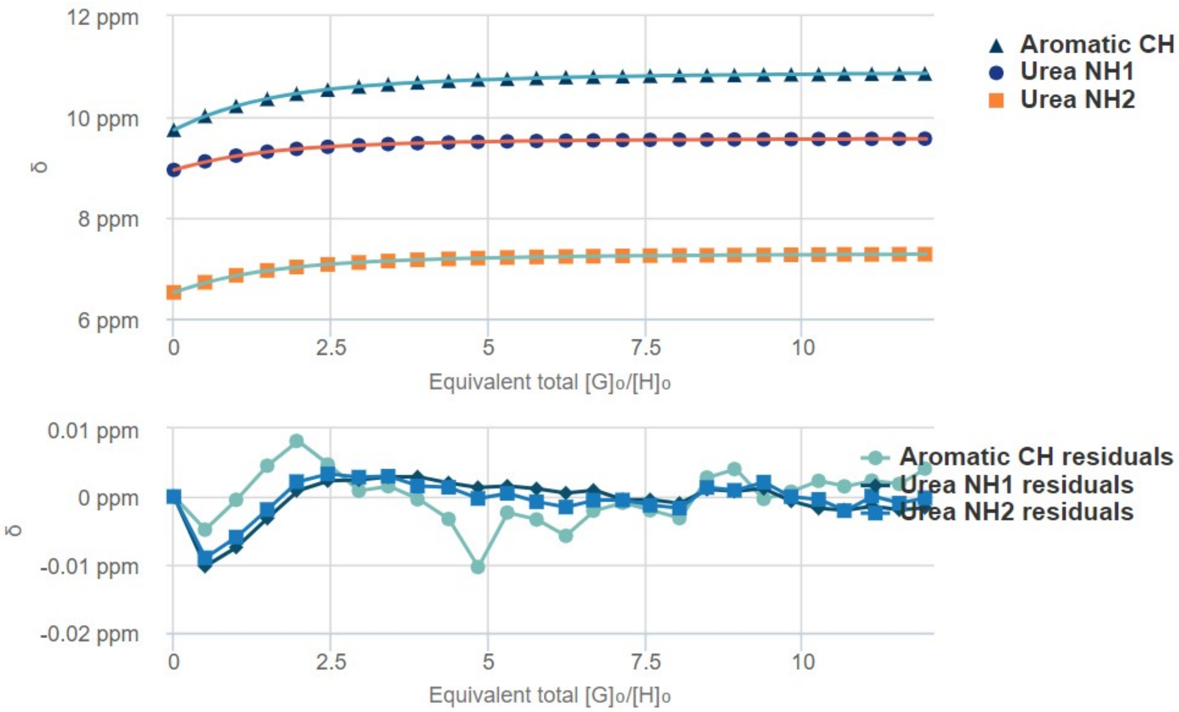

Figure S70. Proton shifts and residuals of complex **2** fitted to a 1:2 model using BindFit. Available at http://app.supramolecular.org/bindfit/view/92e0a806-d414-4223-bd3b-cbe771f16d48

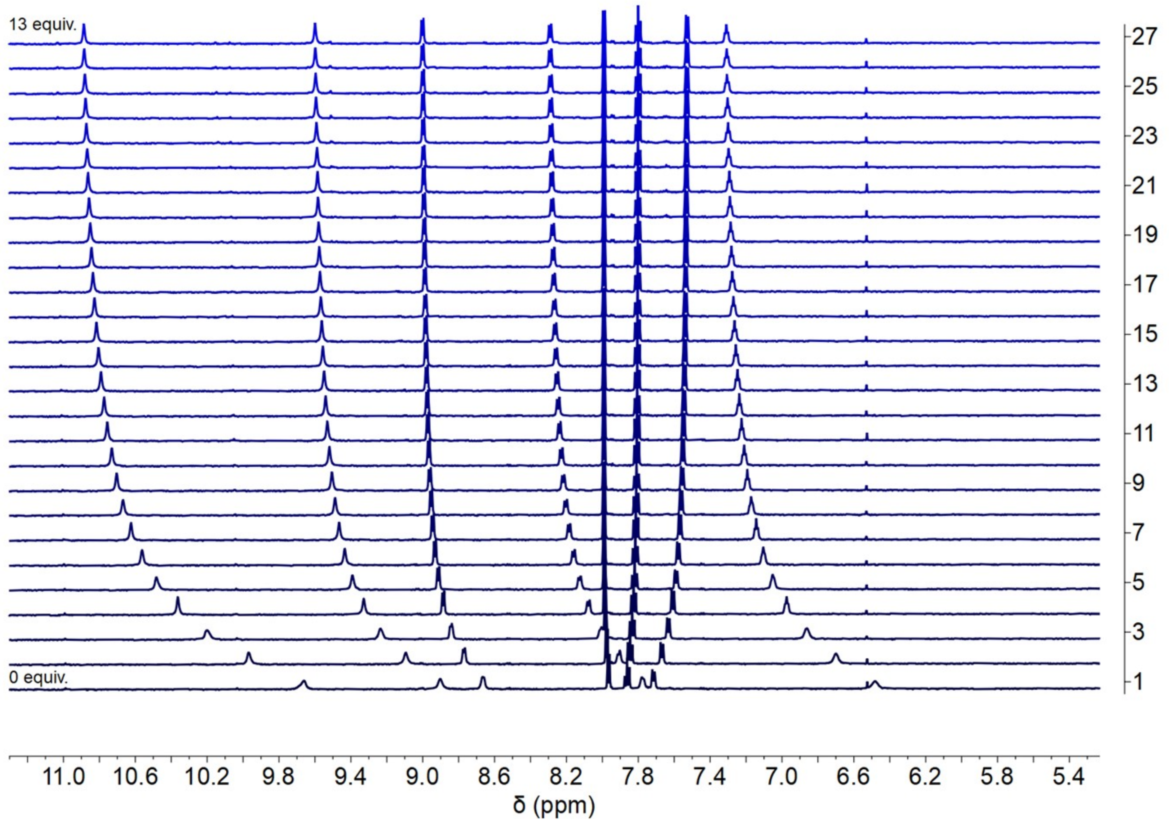

Figure S71. Stack plot of ^{1}H NMR titration of complex **3** with TBA-Cl from 0 – 13 equiv. in DMSO-d_6/0.5 % H_2O at 298 K. Concentrations are normalised against dilution factors.

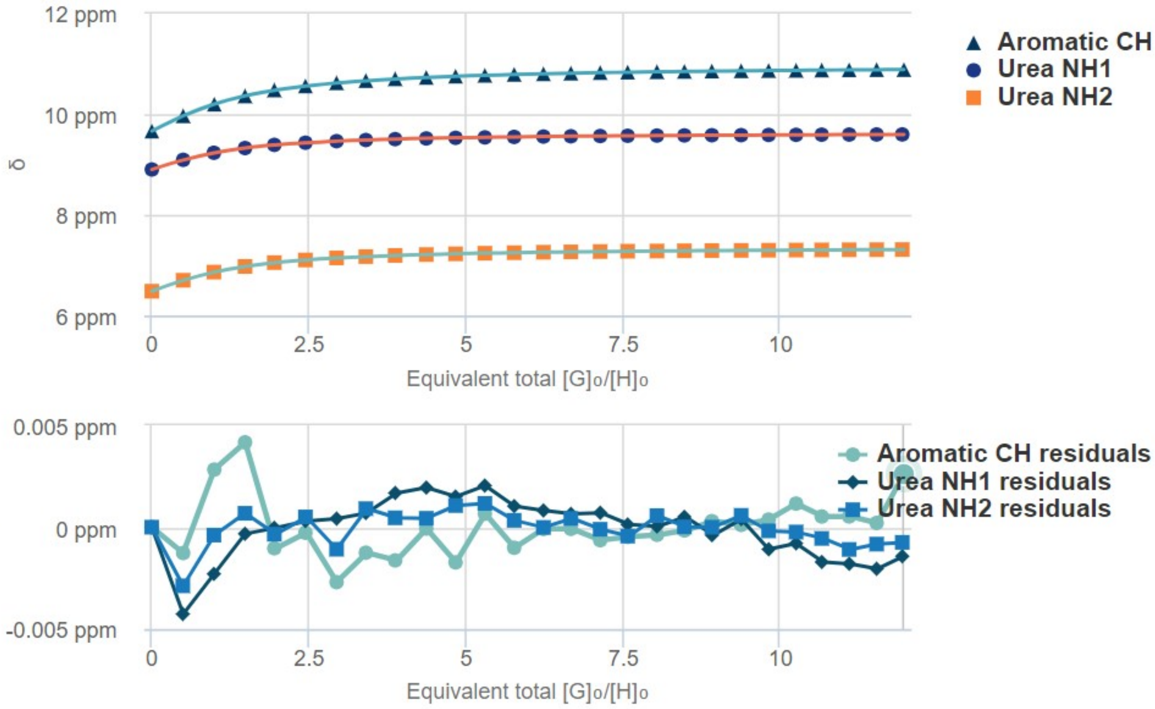

Figure S72. Proton shifts and residuals of complex **3** fitted to a 1:2 model using BindFit. Available at http://app.supramolecular.org/bindfit/view/68984d7c-7e8a-4cc4-9079-0b8939ac6422

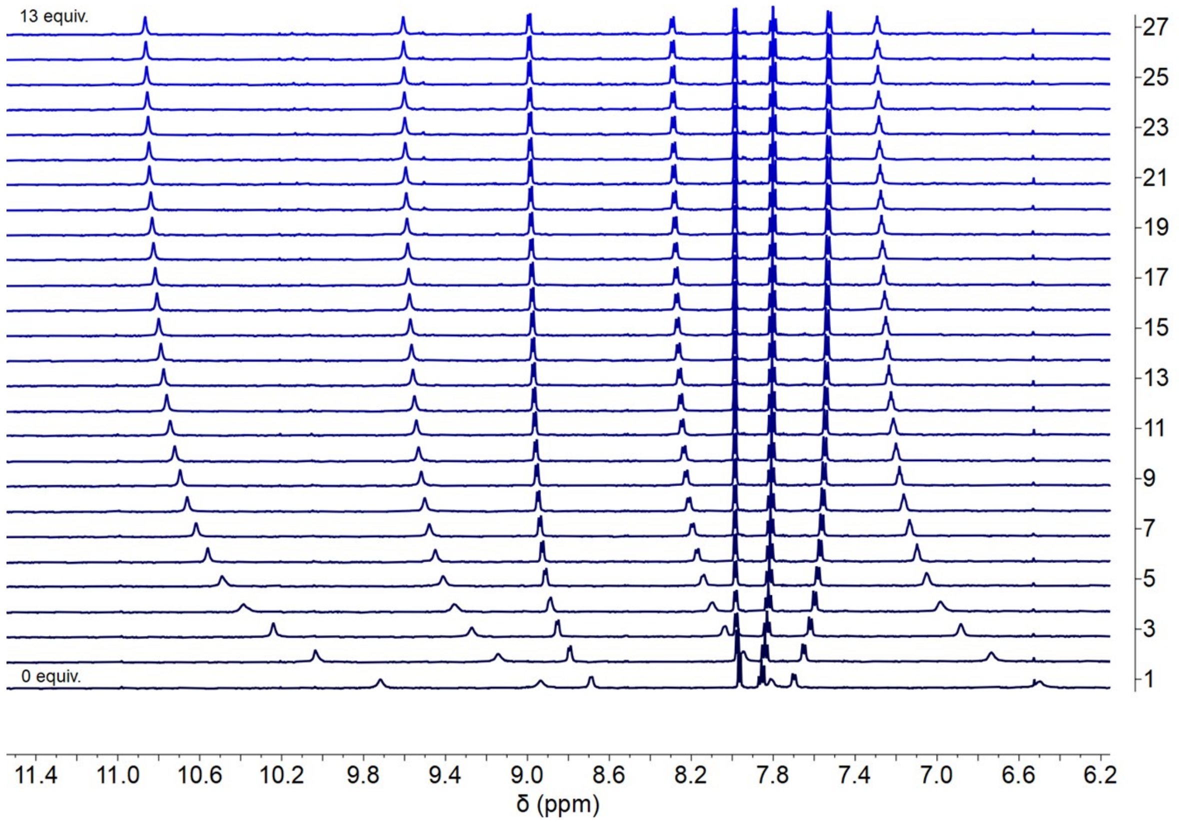

Figure S73. Stack plot of ^{1}H NMR titration of complex **4** with TBA-Cl from 0 – 13 equiv. in DMSO-d_6/0.5 % H_2O at 298 K. Concentrations are normalised against dilution factors.

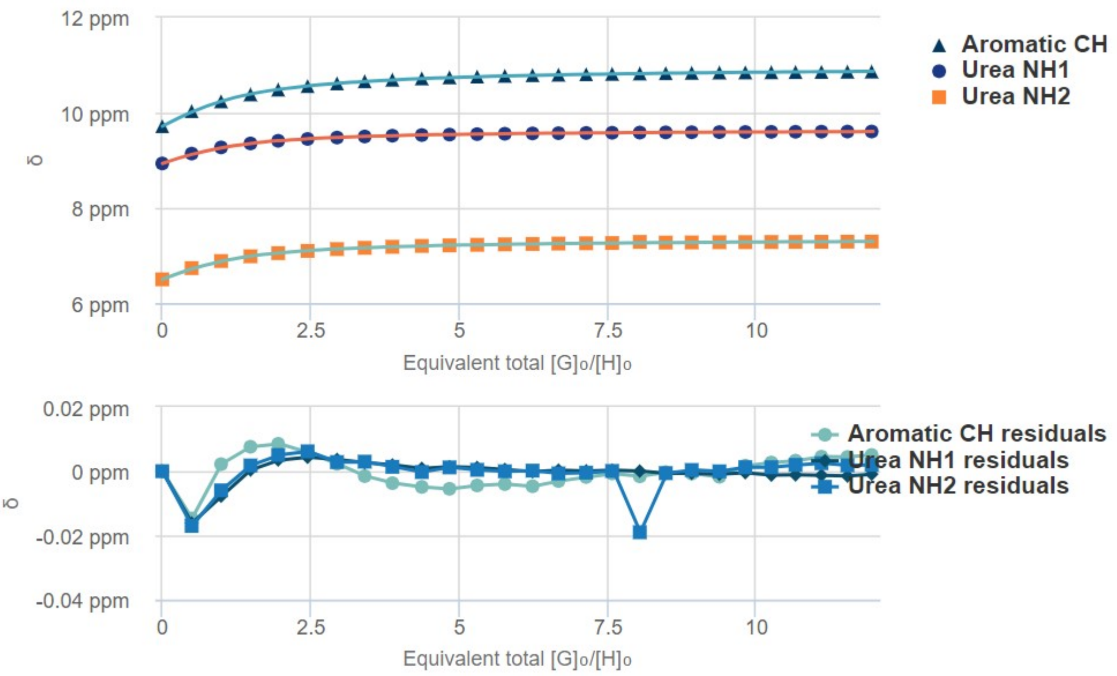

Figure S74. Proton shifts and residuals of complex **4** fitted to a 1:2 model using BindFit. Available at http://app.supramolecular.org/bindfit/view/db390323-a27d-4fcb-980c-5fac0c1f3348

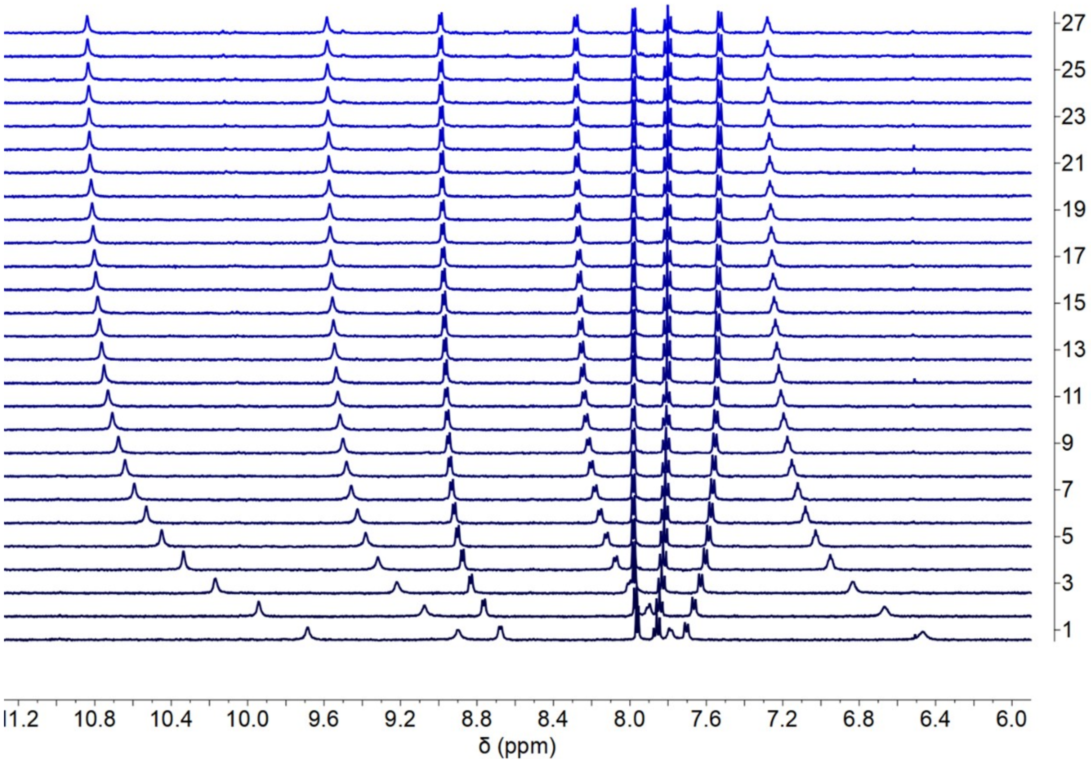

Figure S75. Stack plot of ^{1}H NMR titration of complex **5** with TBA-Cl from 0 – 13 equiv. in DMSO-d_6/0.5 % H_2O at 298 K. Concentrations are normalised against dilution factors.

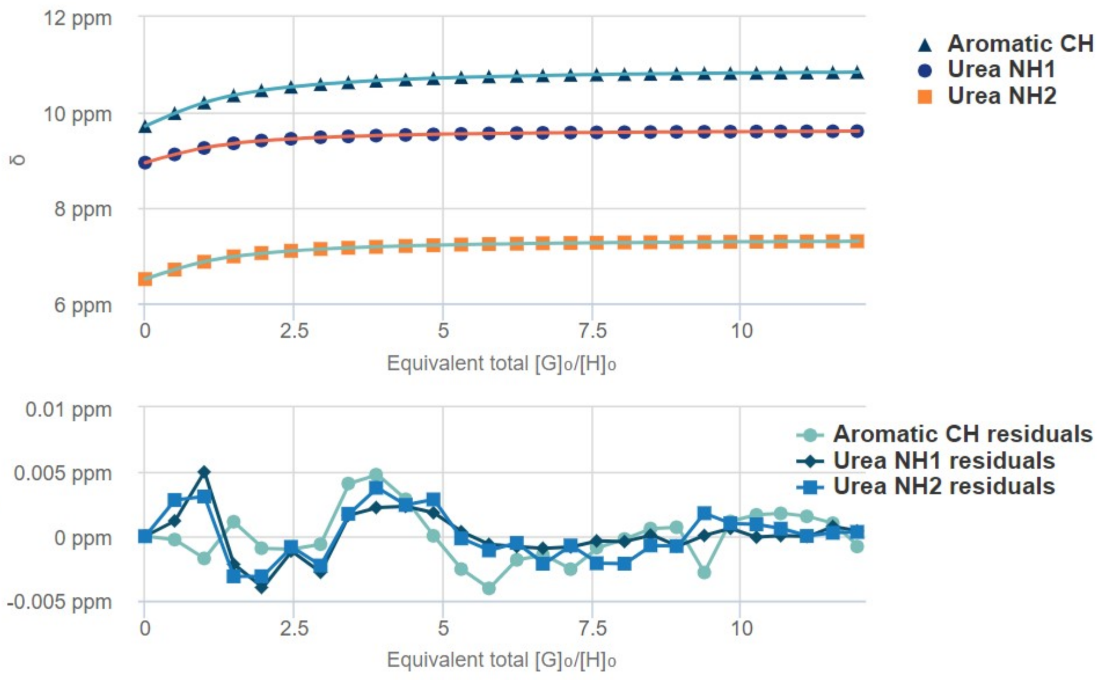

Figure S76. Proton shifts and residuals of complex **5** fitted to a 1:2 model using BindFit. Available at http://app.supramolecular.org/bindfit/view/70753745-ee5c-469e-8961-ec074bc06fec

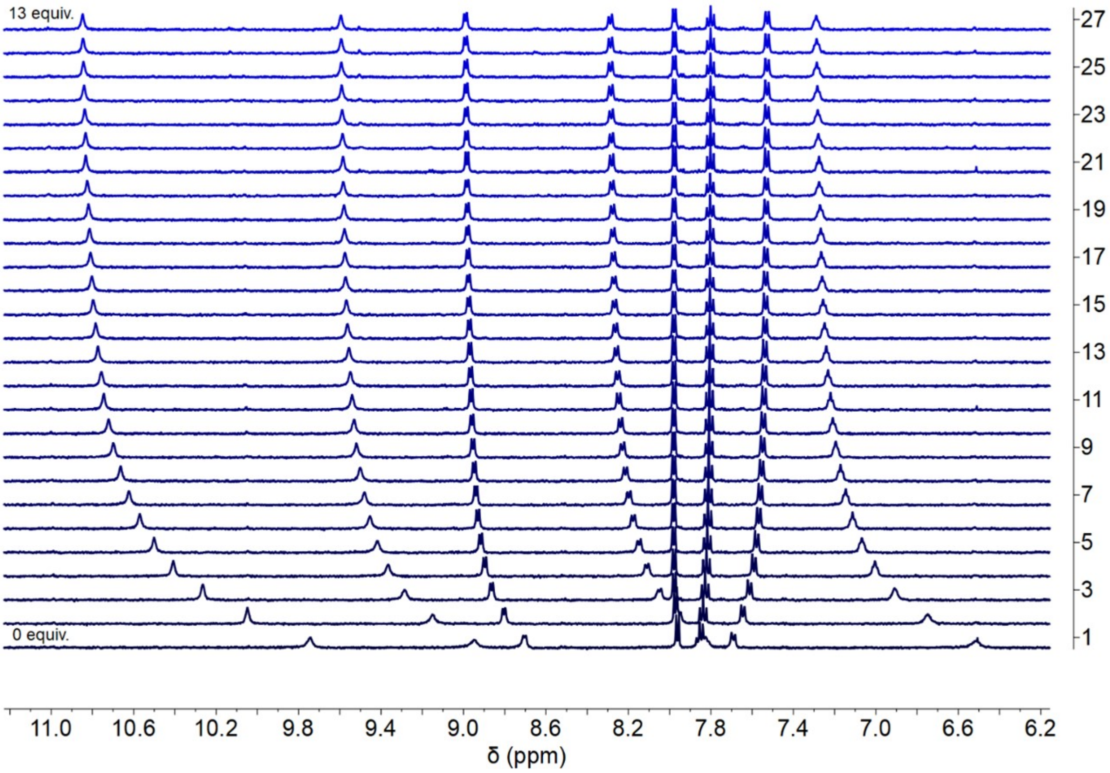

Figure S77. Stack plot of ^{1}H NMR titration of complex **6** with TBA-Cl from 0 – 13 equiv. in DMSO-d_6/0.5 % H_2O at 298 K. Concentrations are normalised against dilution factors.

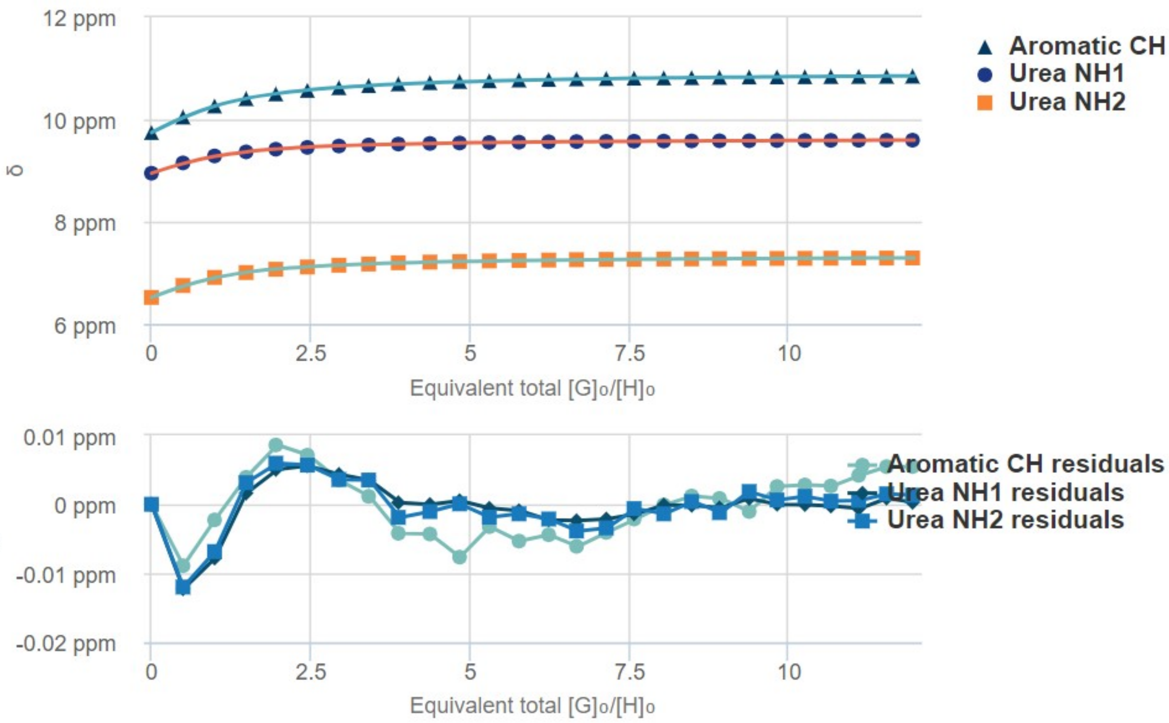

Figure S78. Proton shifts and residuals of complex **6** fitted to a 1:2 model using BindFit. Available at http://app.supramolecular.org/bindfit/view/2100297b-569b-4de9-a1cd-07c69acae043

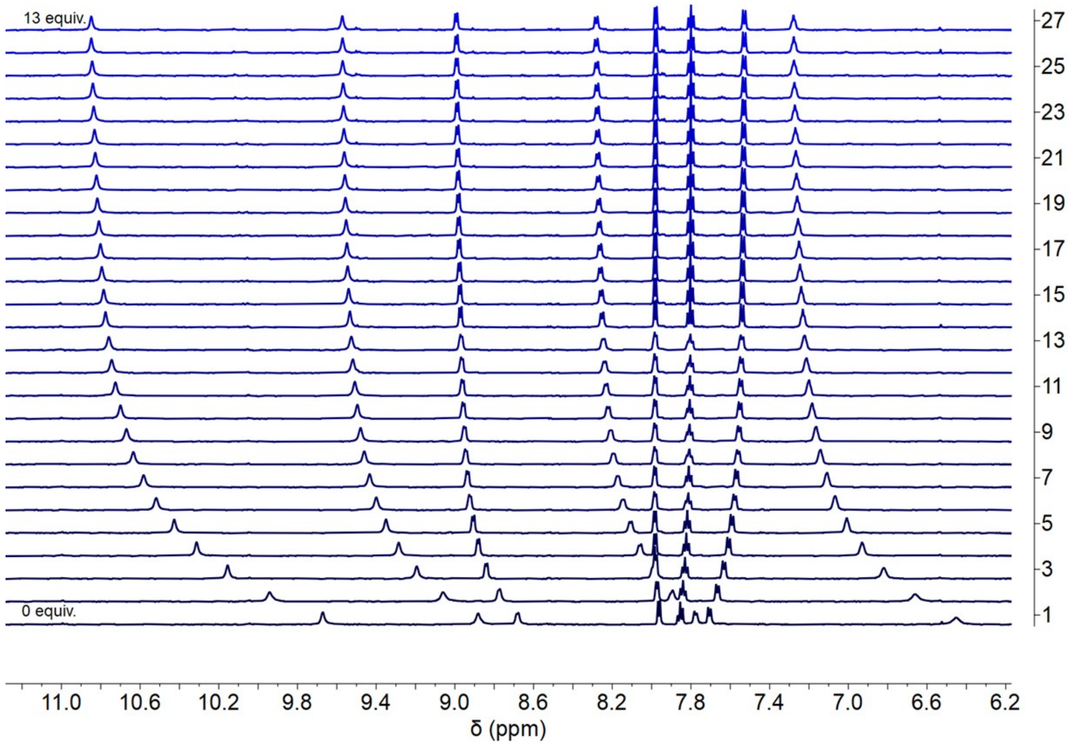

Figure S79. Stack plot of ^{1}H NMR titration of complex **7** with TBA-Cl from 0 – 13 equiv. in DMSO-d_6/0.5 % H_2O at 298 K. Concentrations are normalised against dilution factors.

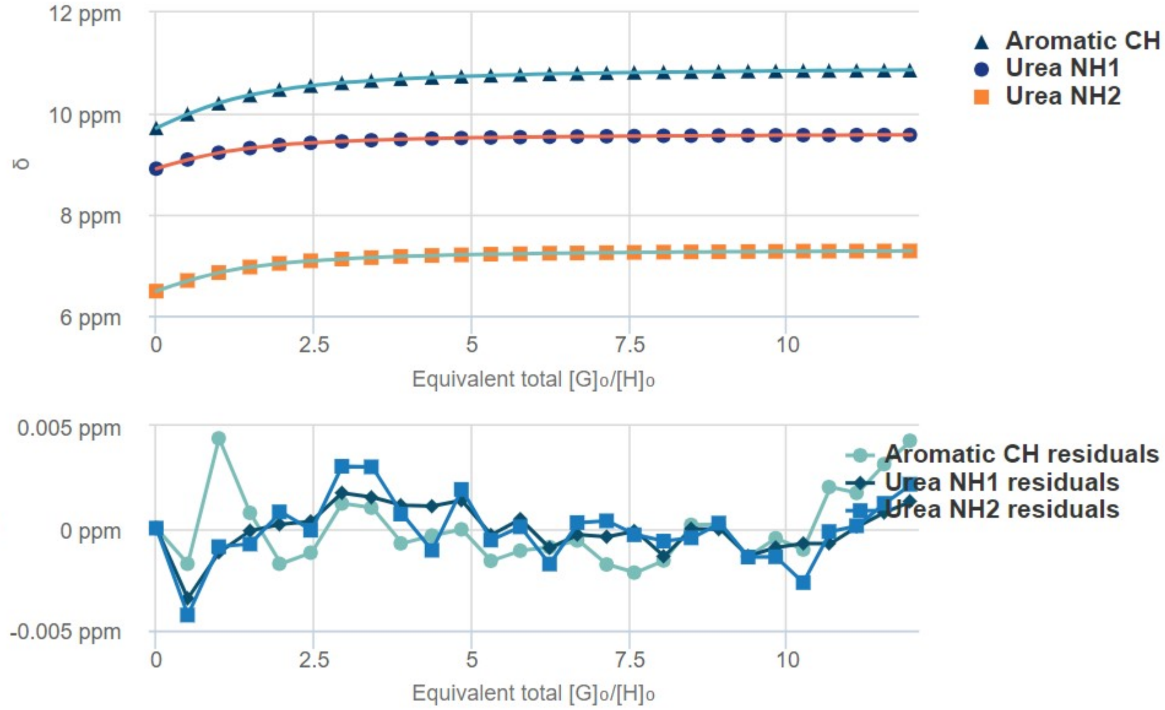

Figure S80. Proton shifts and residuals of complex **7** fitted to a 1:2 model using BindFit. Available at http://app.supramolecular.org/bindfit/view/0b265e9b-61fb-44b8-9443-44d316789f2c

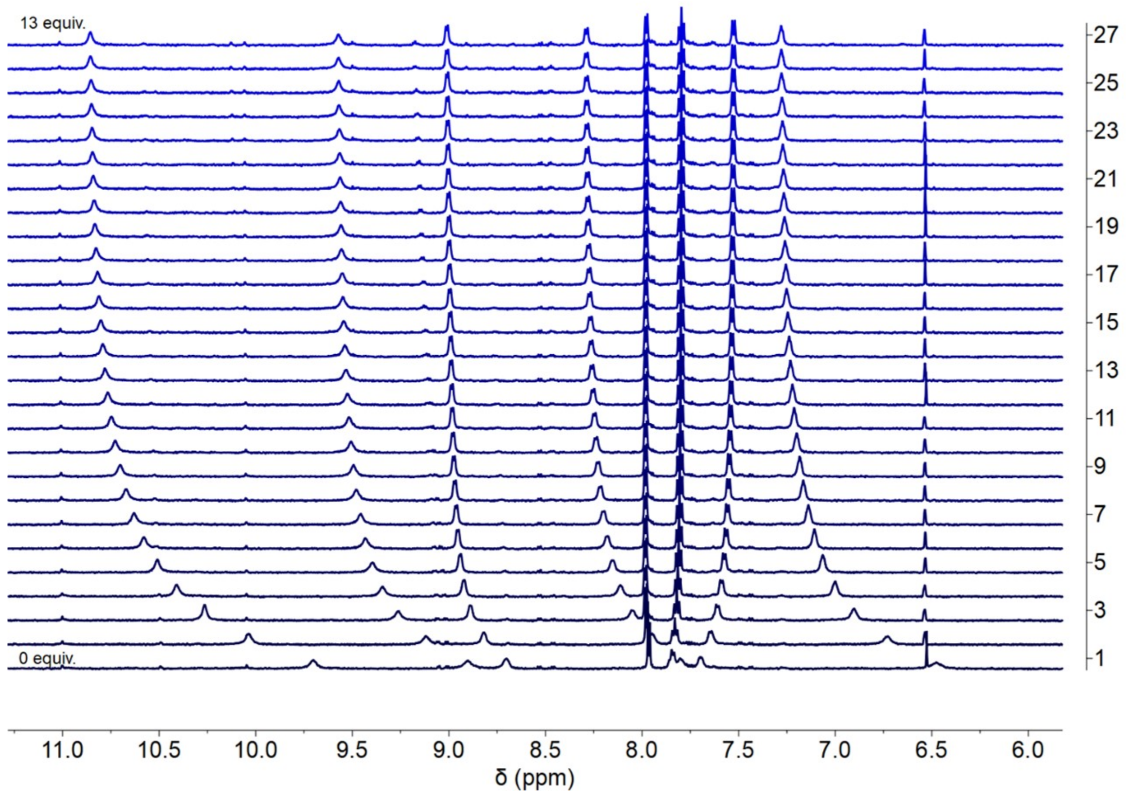

Figure S81. Stack plot of ^1H NMR titration of complex **8** with TBA-Cl from 0 – 13 equiv. in DMSO-d_6/0.5 % H_2O at 298 K. Concentrations are normalised against dilution factors.

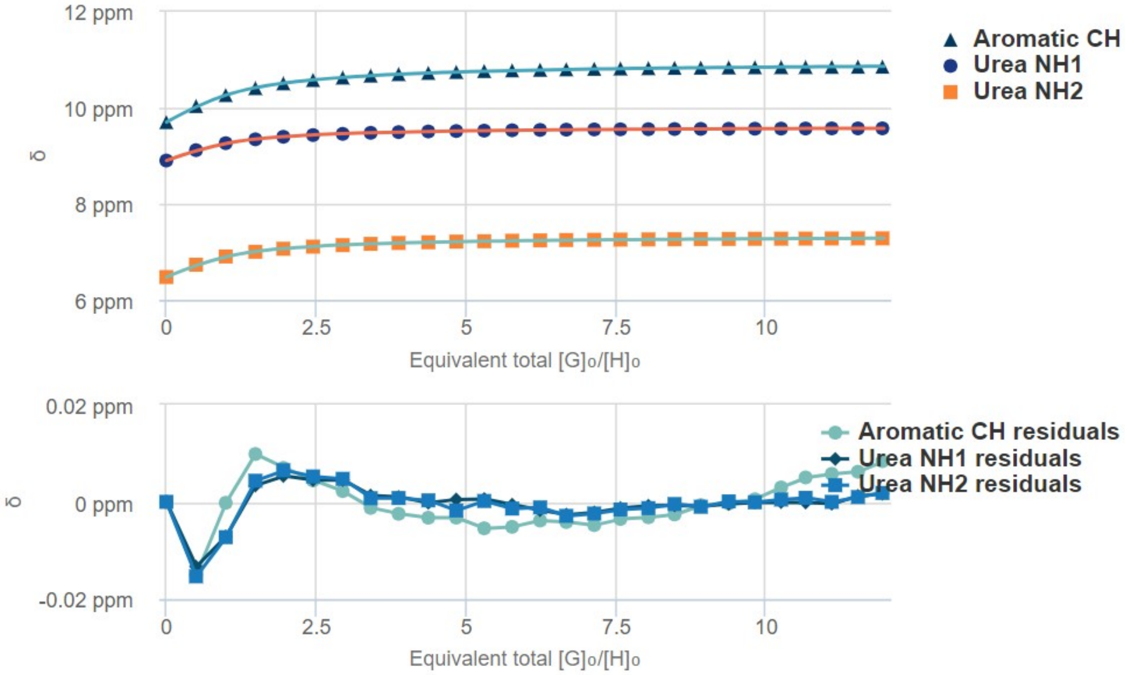

Figure S82. Proton shifts and residuals of complex **8** fitted to a 1:2 model using BindFit. Available at http://app.supramolecular.org/bindfit/view/e703faf6-4498-4128-988f-d26c45e07723

Covariance of Fit Calculations

Table S2: Binding constants (K_a) for complexes **1–8** titrated with chloride, added as TBA-Cl, at 298 K in DMSO-d_6/0.5 % H_2O, calculated using 1:1 and 1:2 binding models. Errors are <8 %.

**Complex**	**Complex Binding Constants (M^{-1})**				
	1:1 (K_a)	cov_{fit}[a]	1:2	cov_{fit}[a]	F cov_{fit}[b]
**1**	4990	8.47×10^{-5}	K_{11}: 17,900<br>K_{12}: 1440	2.21×10^{-5}	3.22
**2**	5670	8.56×10^{-5}	K_{11}: 16,500<br>K_{12}: 1480	2.82×10^{-5}	3.58
**3**	6390	7.65×10^{-5}	K_{11}: 24,500<br>K_{12}: 2280	4.08×10^{-6}	11.8
**4**	6270	1.01×10^{-4}	K_{11}: 24,100<br>K_{12}: 2070	2.28×10^{-5}	3.56
**5**	6210	1.03×10^{-4}	K_{11}: 89,900<br>K_{12}: 3750	1.03×10^{-5}	18.0
**6**	7610	1.69×10^{-4}	K_{11}: 30,500<br>K_{12}: 2230	4.61×10^{-5}	2.92
**7**	5710	1.34×10^{-4}	K_{11}: 25,100<br>K_{12}: 2760	9.76×10^{-6}	15.4
**8**	8310	2.36×10^{-4}	K_{11}: 42,100<br>K_{12}: 2030	5.13×10^{-5}	3.81

[a] The covariance of fit (cov_{fit}) for 1:1 and 1:2 binding models, derived by dividing the calculated covariance (cov_{calc}) by the covariance of the residual (experimental – calculated). [b] Factor of covariance of fit (F cov_{fit}), calculated by dividing the 1:1 cov_{fit} by 1:2 cov_{fit}. 1:2 binding is the preferred model if F $cov_{fit} > 5$.[15]

Transport data

ISE Cl^-/NO_3^- Exchange

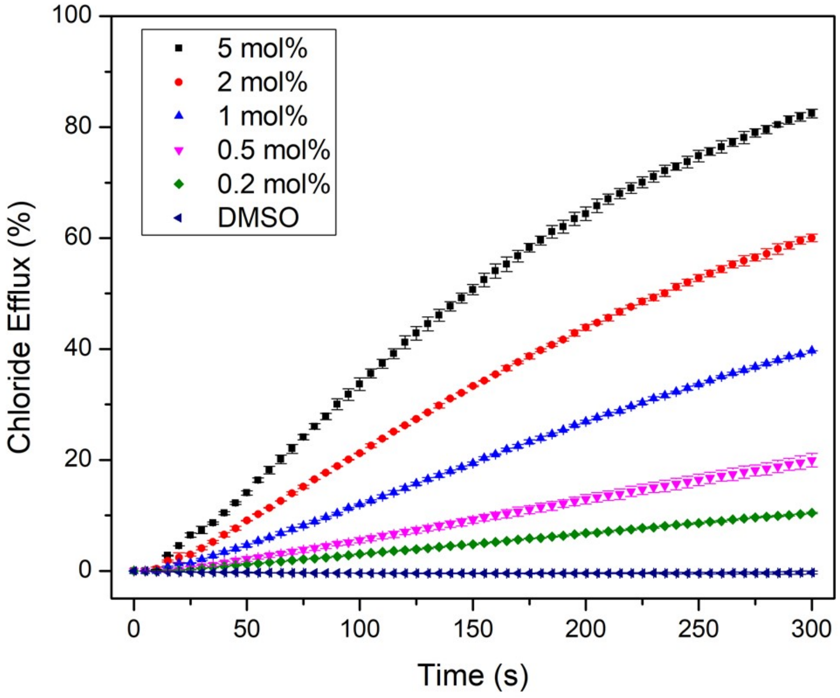

Figure S83. Cl^-/NO_3^- exchange mediated by complex **1** over 300 s at varying concentrations in POPC vesicles loaded with 487 mM NaCl, suspended in 487 mM $NaNO_3$ at pH 7.2.

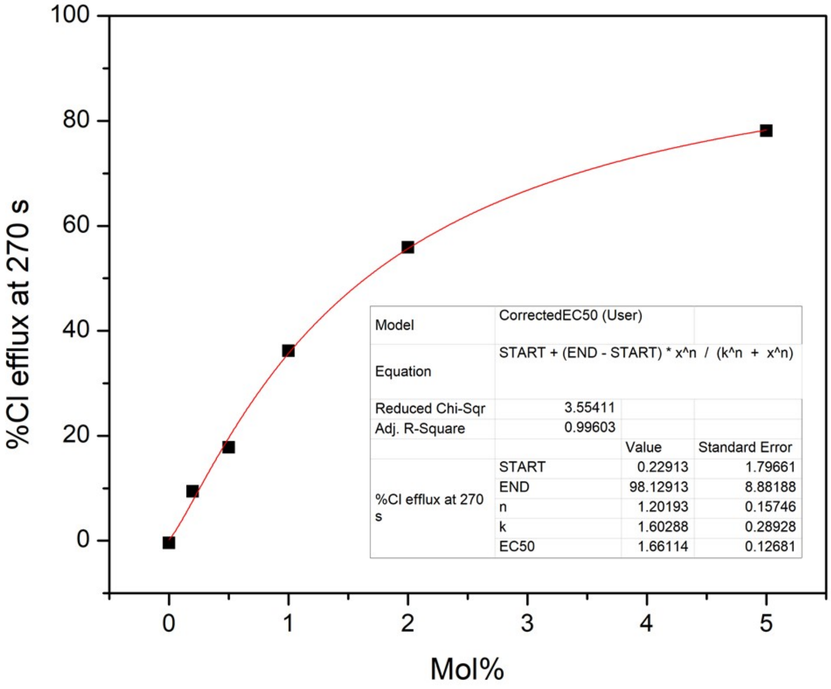

Figure S84. Hill plot obtained for complex **1** in the ISE Cl^-/NO_3^- assay, including calculated EC_{50} value and fitted curve. Cl^- efflux at 270 s was taken from each experiment; each point represents the average of 3 repeats.

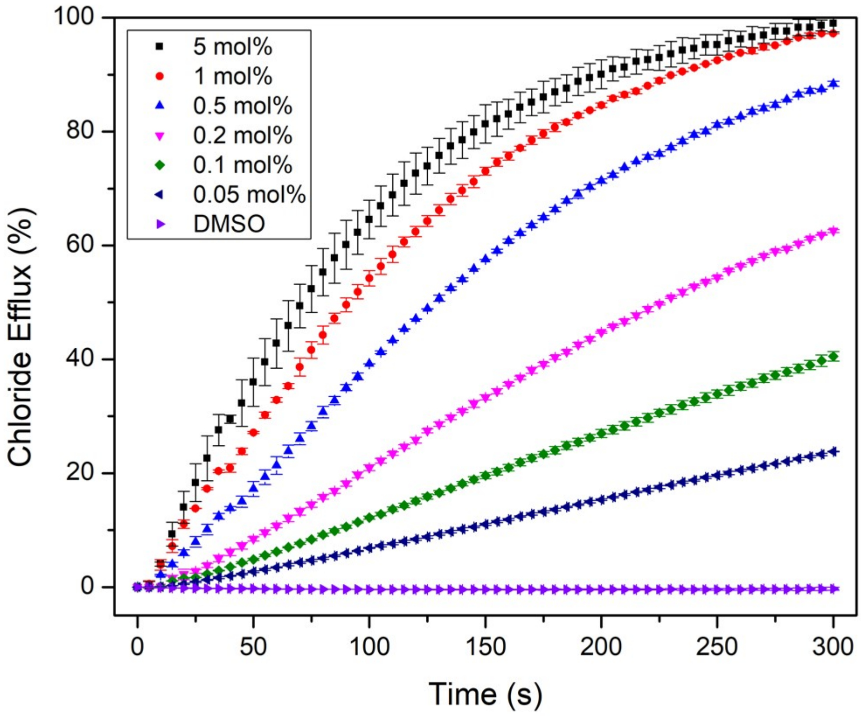

Figure S85. Cl^-/NO_3^- exchange mediated by complex **2** over 300 s at varying concentrations in POPC vesicles loaded with 487 mM NaCl, suspended in 487 mM $NaNO_3$ at pH 7.2.

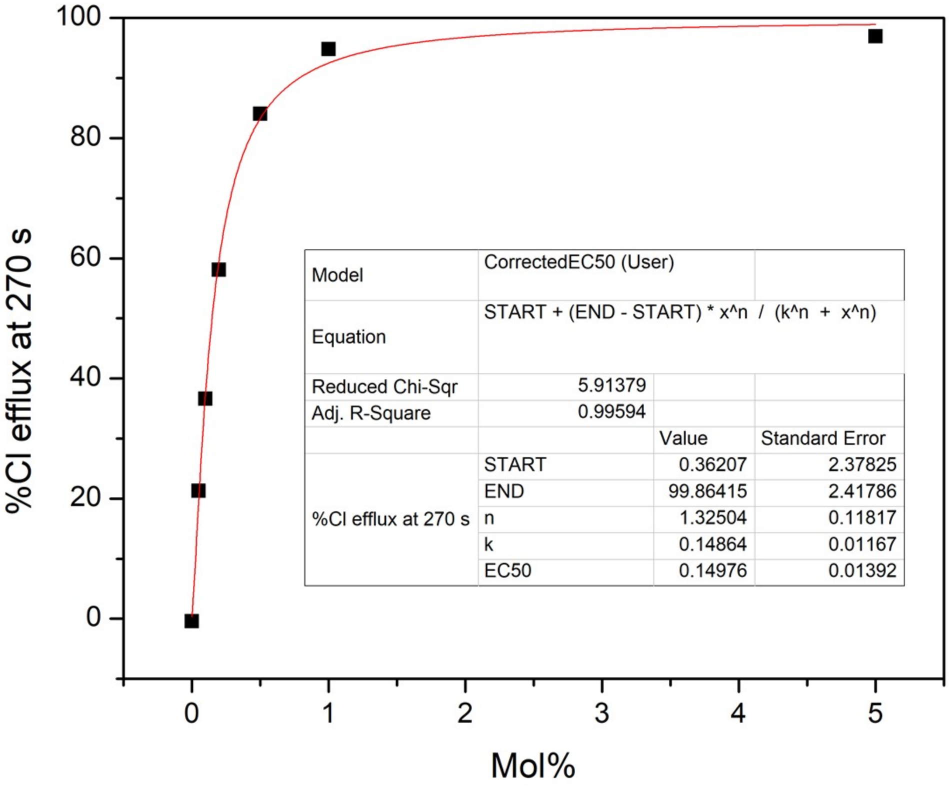

Figure S86. Hill plot obtained for complex **2** in the ISE Cl^-/NO_3^- assay, including calculated EC_{50} value and fitted curve. Cl^- efflux at 270 s was taken from each experiment; each point represents the average of 3 repeats.

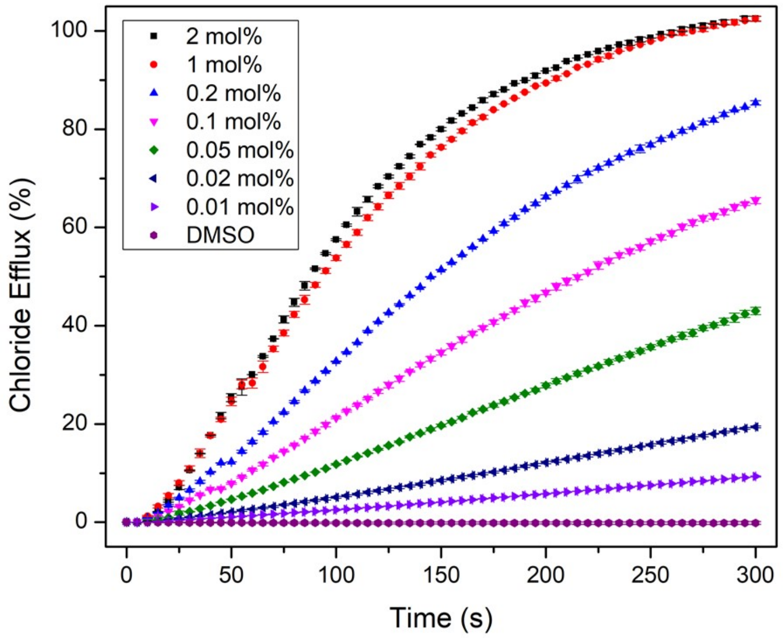

Figure S87. Cl^-/NO_3^- exchange mediated by complex **3** over 300 s at varying concentrations in POPC vesicles loaded with 487 mM NaCl, suspended in 487 mM $NaNO_3$ at pH 7.2.

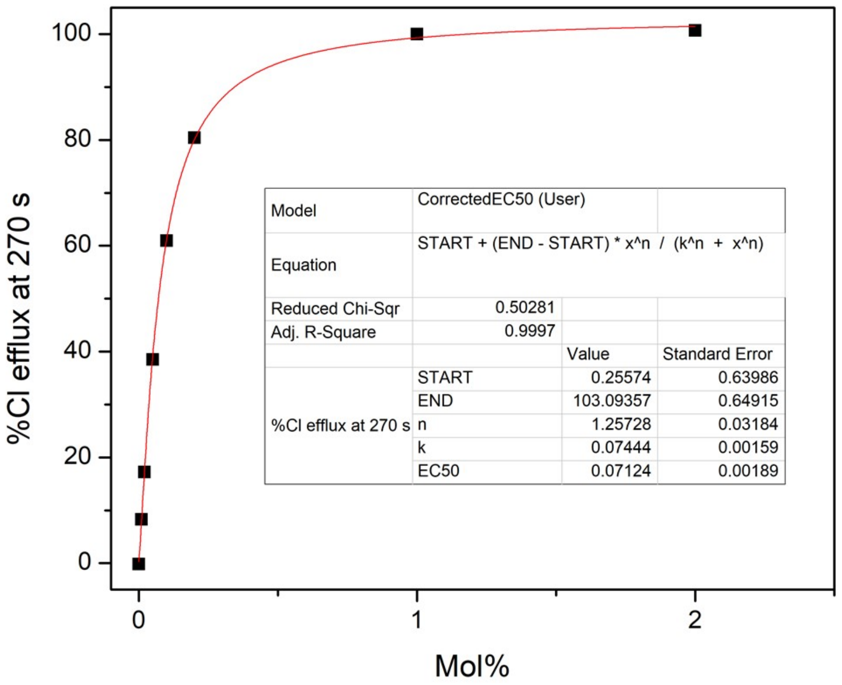

Figure S88. Hill plot obtained for complex **3** in the ISE Cl^-/NO_3^- assay, including calculated EC_{50} value and fitted curve. Cl^- efflux at 270 s was taken from each experiment; each point represents the average of 3 repeats.

ISE Cl^-/SO_4^{2-} Exchange

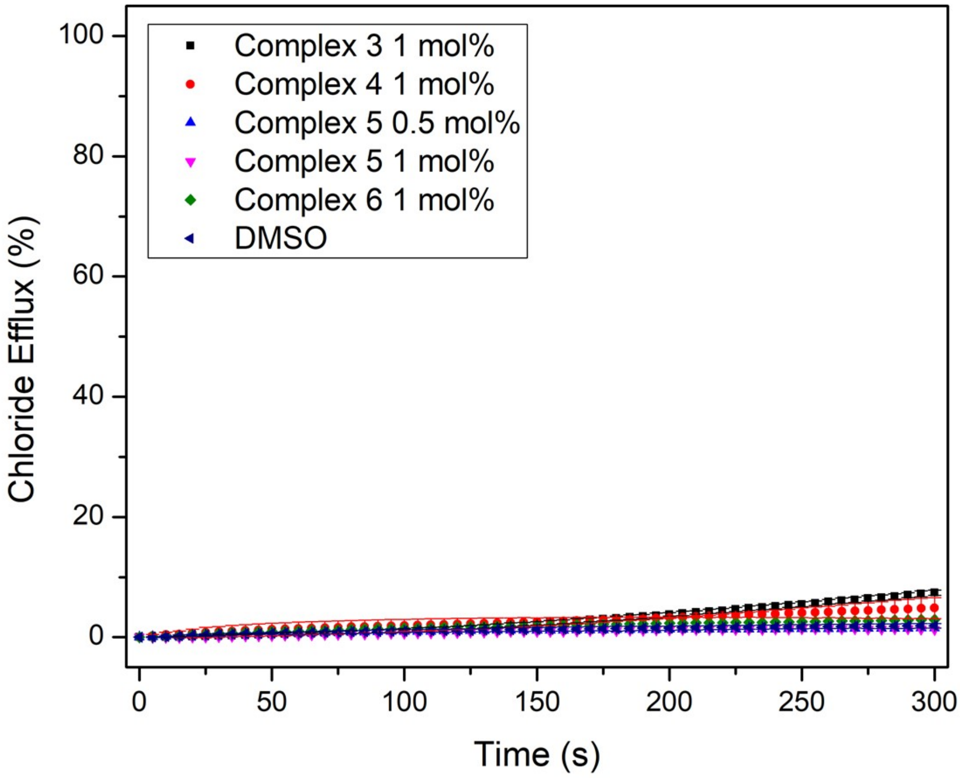

Figure S89. Cl^-/SO_4^{2-} exchange mediated by complexes **3**–**6** over 300 s at 1 and 0.5 mol% concentrations in POPC vesicles loaded with 487 mM NaCl, suspended in 487 mM Na_2SO_4 at pH 7.2.

ISE Cationophore Coupled Transport

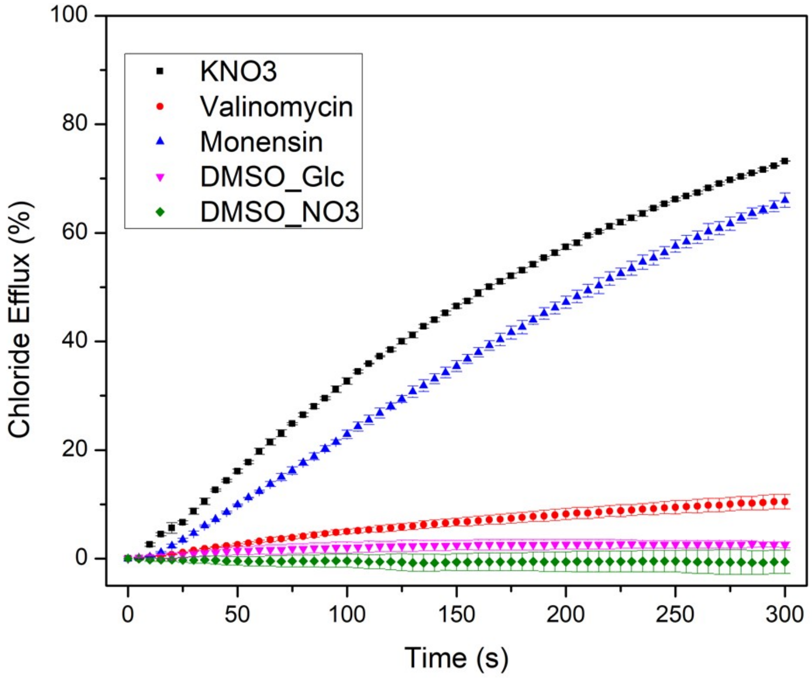

Figure S90. Cl^- efflux mediated by complex **1** over 300 s at 2 mol% in POPC vesicles loaded with 300 mM NaCl. Vesicles are suspended in either KNO_3 or KGlc in the presence of cationophores.

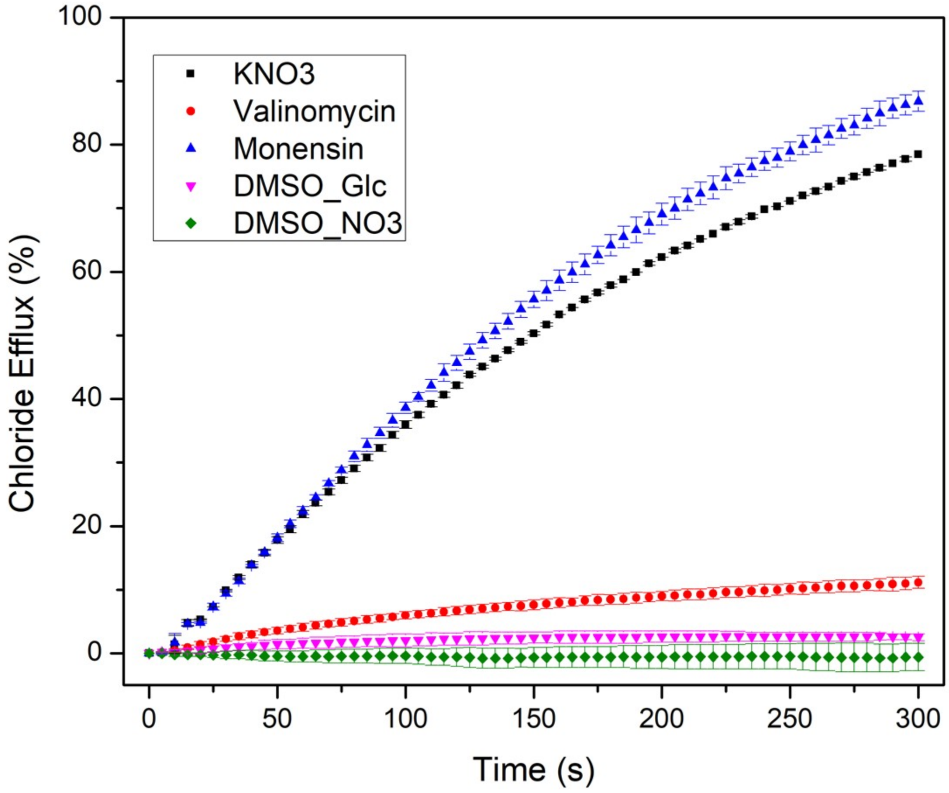

Figure S91. Cl⁻ efflux mediated by complex **2** over 300 s at 0.2 mol% in POPC vesicles loaded with 300 mM NaCl. Vesicles are suspended in either KNO_3 or KGlc in the presence of cationophores.

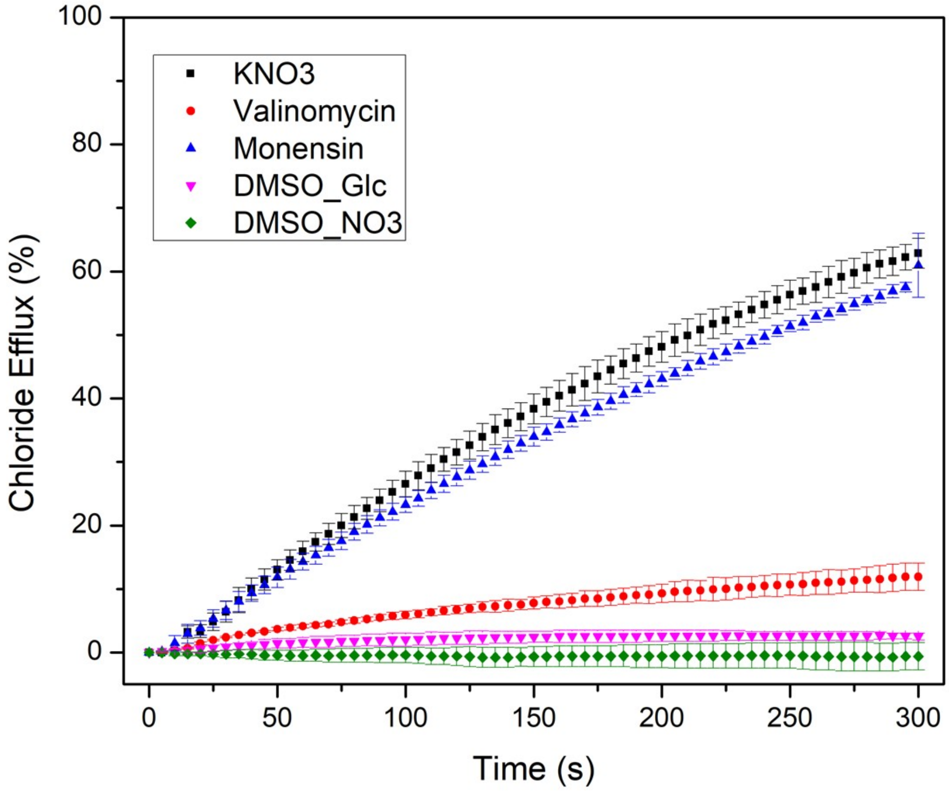

Figure S92. Cl⁻ efflux mediated by complex **3** over 300 s at 0.05 mol% in POPC vesicles loaded with 300 mM NaCl. Vesicles are suspended in either KNO_3 or KGlc in the presence of cationophores.

HPTS Assay

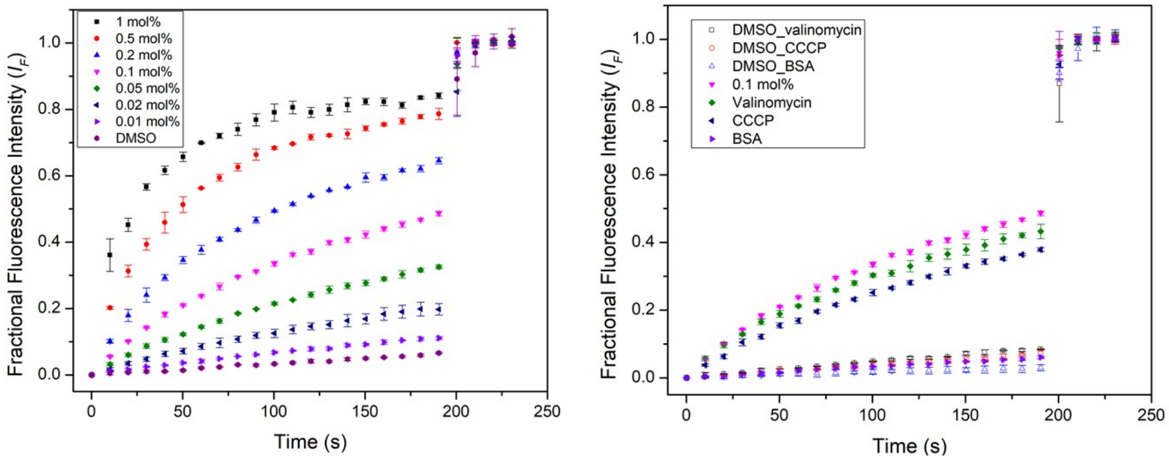

Figure S93. HPTS fluorescence for complex **1** at varying concentrations in POPC vesicles loaded with HPTS in the presence of fatty acid impurities (left) and at the EC_{50} concentration with BSA, valinomycin or CCCP (right). Each point represents the average of 3 repeats.

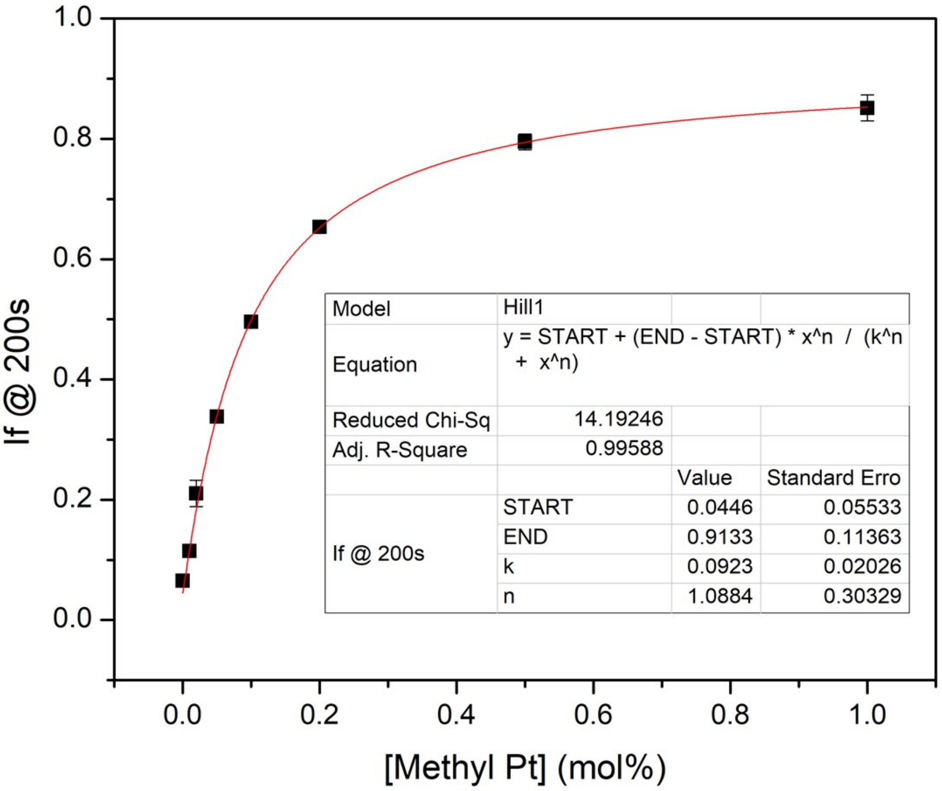

Figure S94. Hill plot obtained for complex **1** in the HPTS assay in the presence of fatty acid impurities, including calculated EC_{50} value and fitted curve. Fractional intensity at 200 s was taken from each experiment; each point represents the average of 3 repeats.

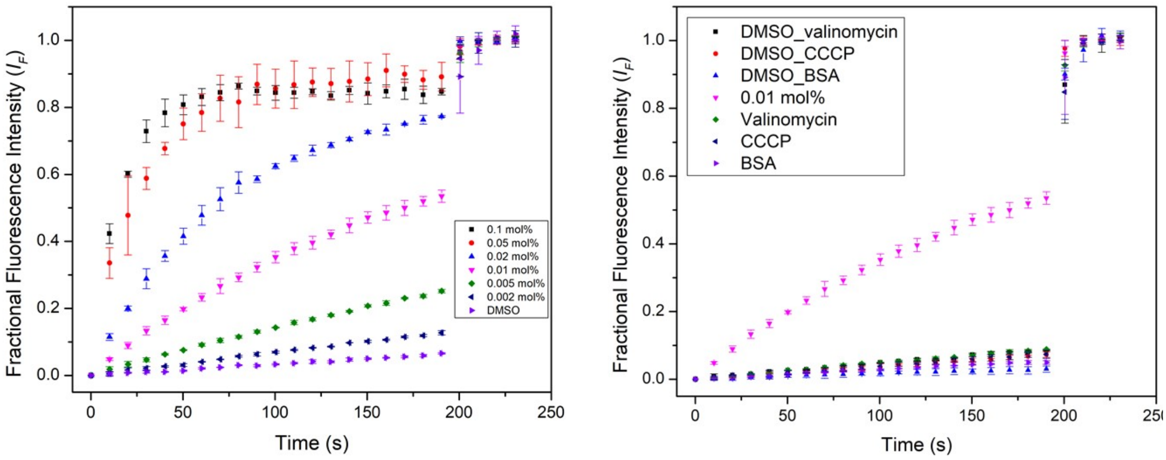

Figure S95. HPTS fluorescence for complex **2** at varying concentrations in POPC vesicles loaded with HPTS in the presence of fatty acid impurities (left) and at the EC_{50} concentration with BSA, valinomycin or CCCP (right). Each point represents the average of 3 repeats.

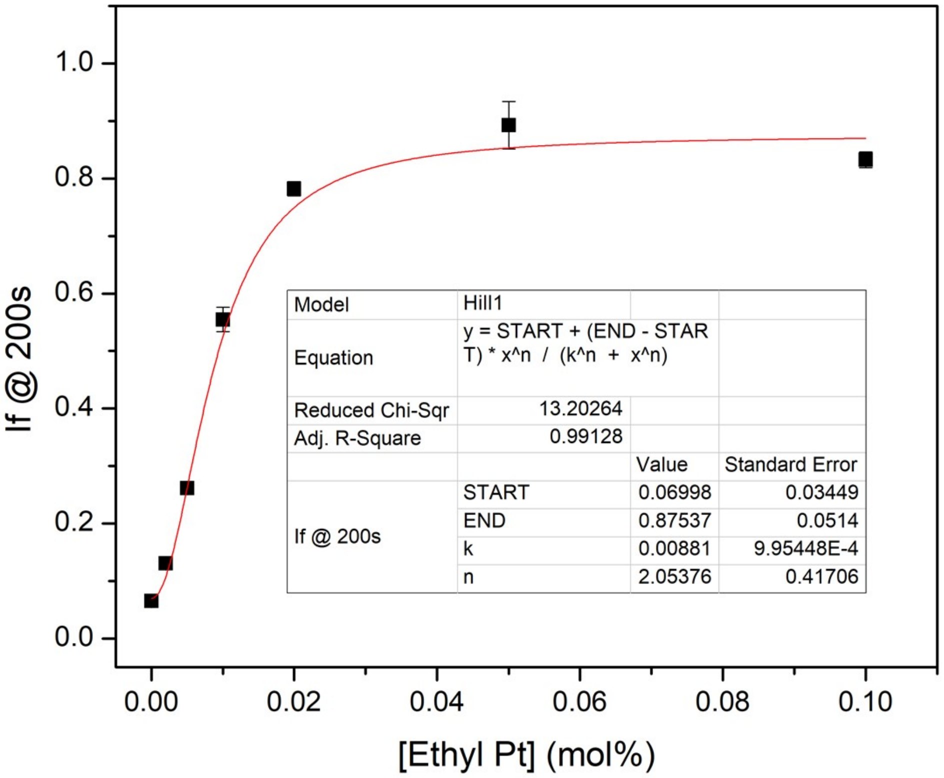

Figure S96. Hill plot obtained for complex **2** in the HPTS assay in the presence of fatty acid impurities, including calculated EC_{50} value and fitted curve. Fractional intensity at 200 s was taken from each experiment; each point represents the average of 3 repeats.

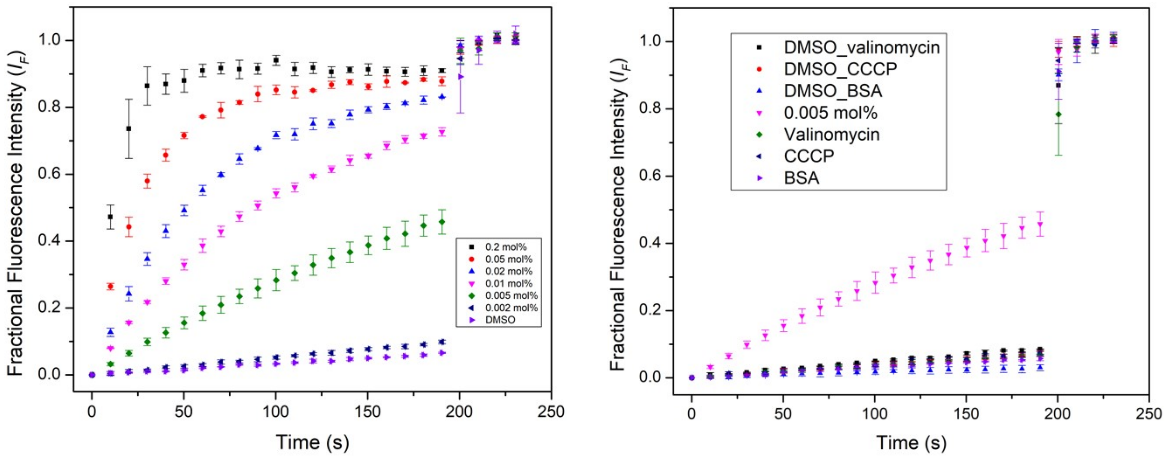

Figure S97. HPTS fluorescence for complex **3** at varying concentrations in POPC vesicles loaded with HPTS in the presence of fatty acid impurities (left) and at the EC_{50} concentration with BSA, valinomycin or CCCP (right). Each point represents the average of 3 repeats.

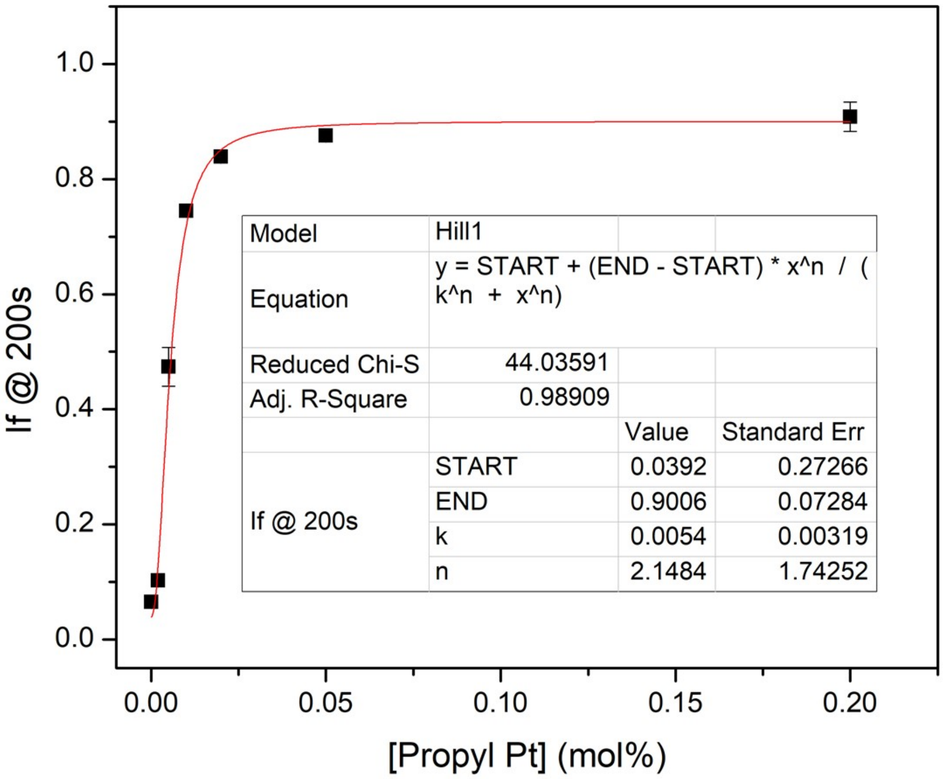

Figure S98. Hill plot obtained for complex **3** in the HPTS assay in the presence of fatty acid impurities, including calculated EC_{50} value and fitted curve. Fractional intensity at 200 s was taken from each experiment; each point represents the average of 3 repeats.

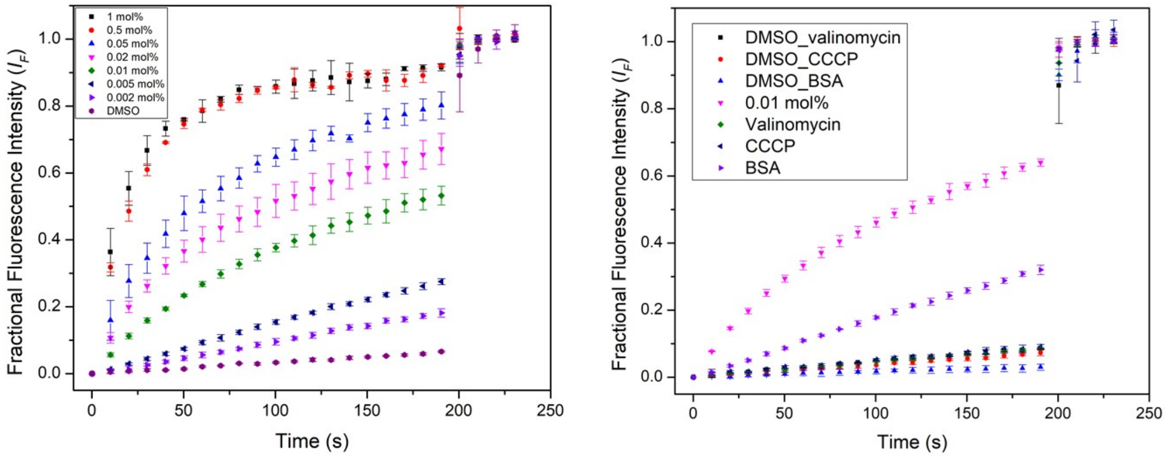

Figure S99. HPTS fluorescence for complex **4** at varying concentrations in POPC vesicles loaded with HPTS in the presence of fatty acid impurities (left) and at the EC_{50} concentration with BSA, valinomycin or CCCP (right). Each point represents the average of 3 repeats.

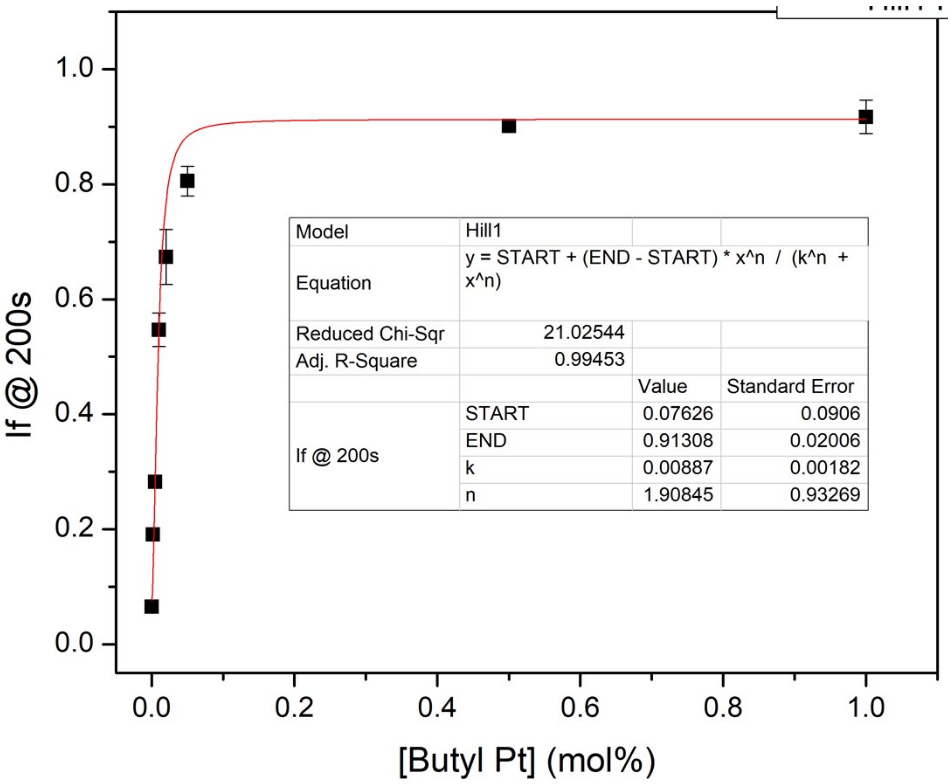

Figure S100. Hill plot obtained for complex **4** in the HPTS assay in the presence of fatty acid impurities, including calculated EC_{50} value and fitted curve. Fractional intensity at 200 s was taken from each experiment; each point represents the average of 3 repeats.

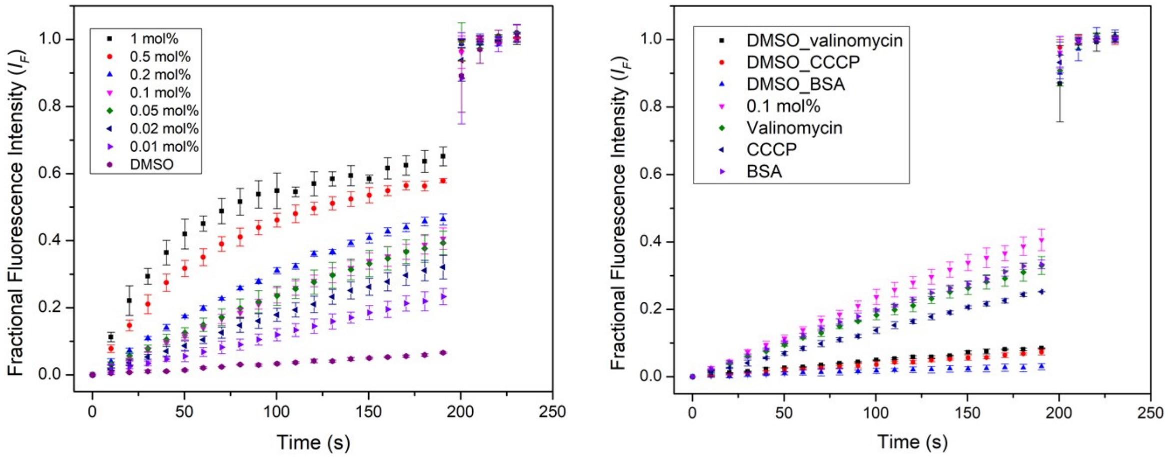

Figure S101. HPTS fluorescence for complex **5** at varying concentrations in POPC vesicles loaded with HPTS in the presence of fatty acid impurities (left) and at the EC_{50} concentration with BSA, valinomycin or CCCP (right). Each point represents the average of 3 repeats.

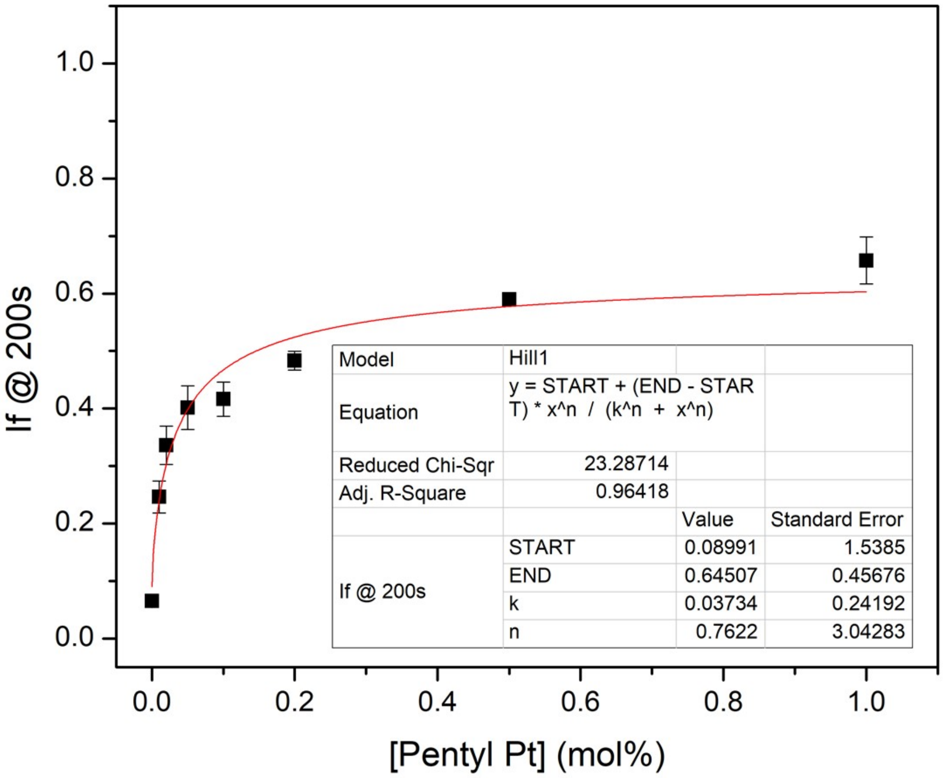

Figure S102. Hill plot obtained for complex **5** in the HPTS assay in the presence of fatty acid impurities, including calculated EC_{50} value and fitted curve. Fractional intensity at 200 s was taken from each experiment; each point represents the average of 3 repeats.

HPTS Oleic Acid Assay

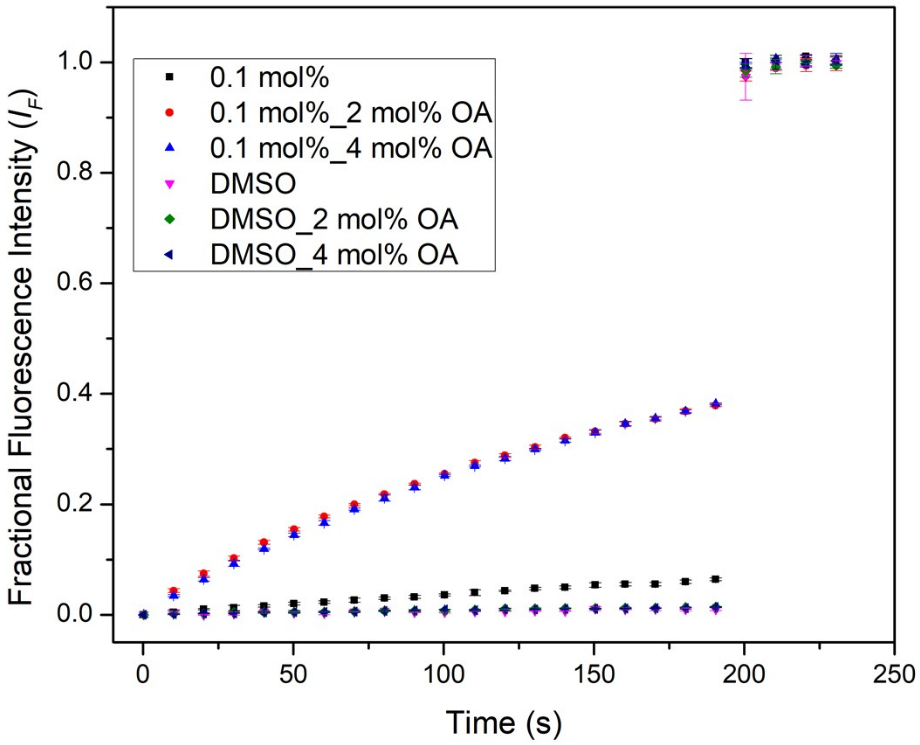

Figure S103. HPTS fluorescence for complex **1** at the EC_{50} concentration in Avanti Polar Lipids. Oleic acid was added to mimic the function of fatty acids. Each point represents the average of 3 repeats.

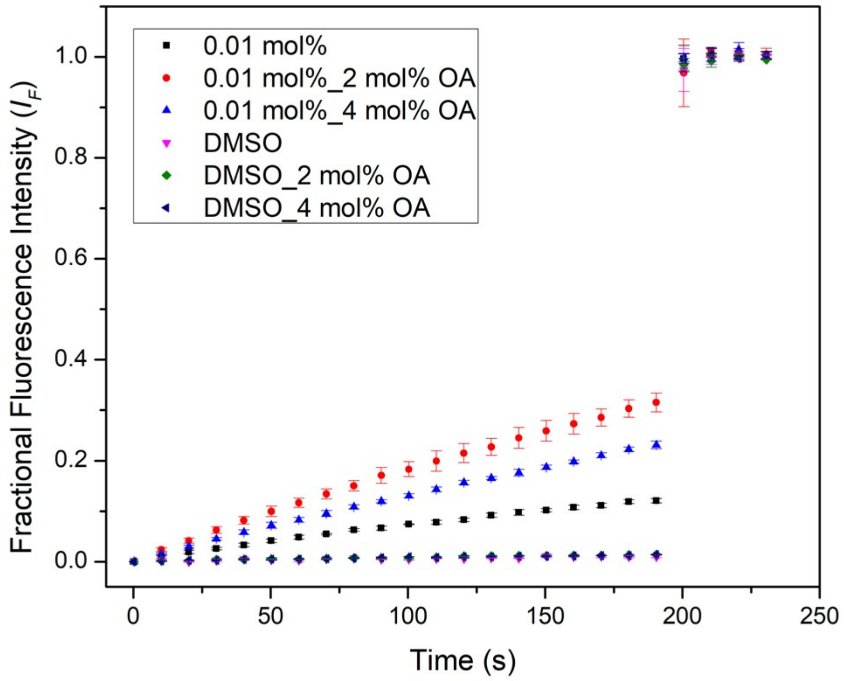

Figure S104. HPTS fluorescence for complex **2** at the EC_{50} concentration in Avanti Polar Lipids. Oleic acid was added to mimic the function of fatty acids. Each point represents the average of 3 repeats.

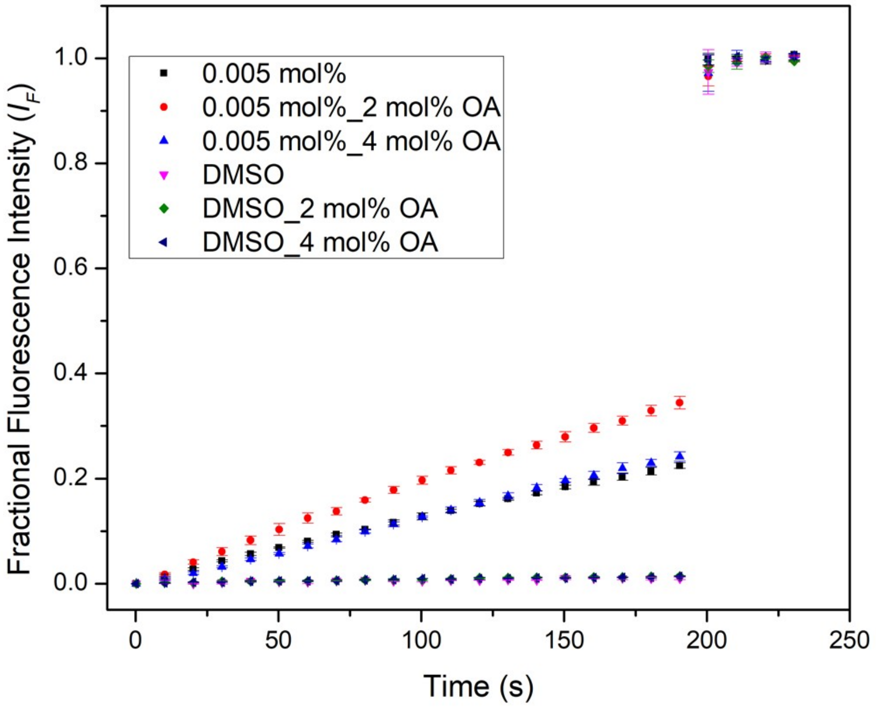

Figure S105. HPTS fluorescence for complex **3** at the EC_{50} concentration in Avanti Polar Lipids. Oleic acid was added to mimic the function of fatty acids. Each point represents the average of 3 repeats.

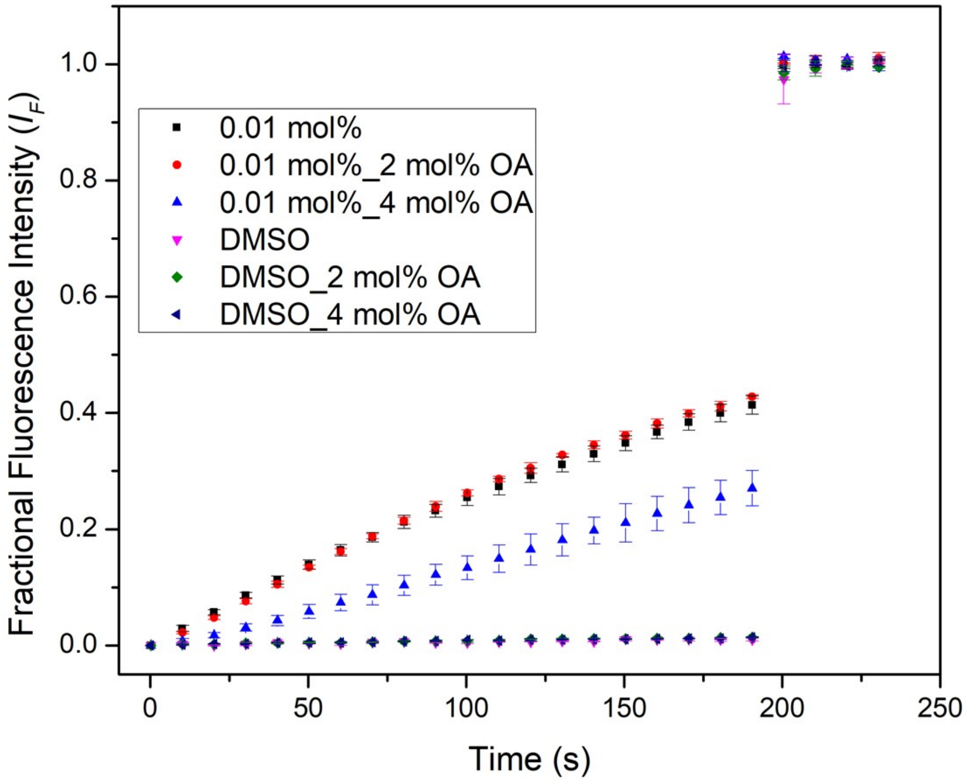

Figure S106. HPTS fluorescence for complex **4** at the EC_{50} concentration in Avanti Polar Lipids. Oleic acid was added to mimic the function of fatty acids. Each point represents the average of 3 repeats.

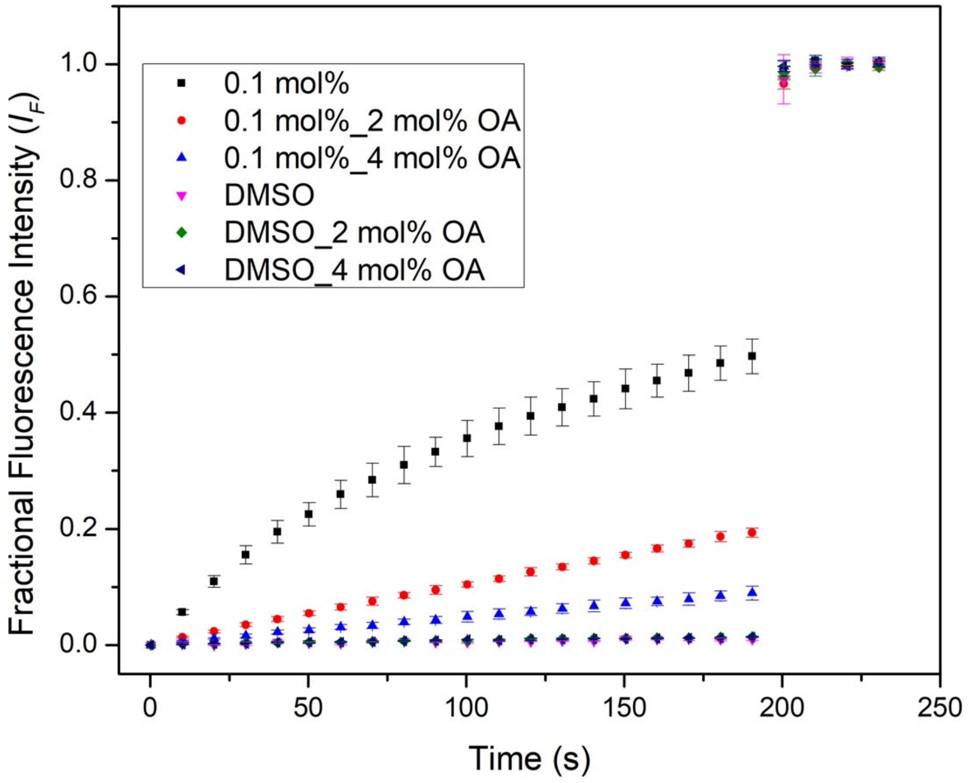

Figure S107. HPTS fluorescence for complex **5** at the EC_{50} concentration in Avanti Polar Lipids. Oleic acid was added to mimic the function of fatty acids. Each point represents the average of 3 repeats.

Anion Selectivity Assay

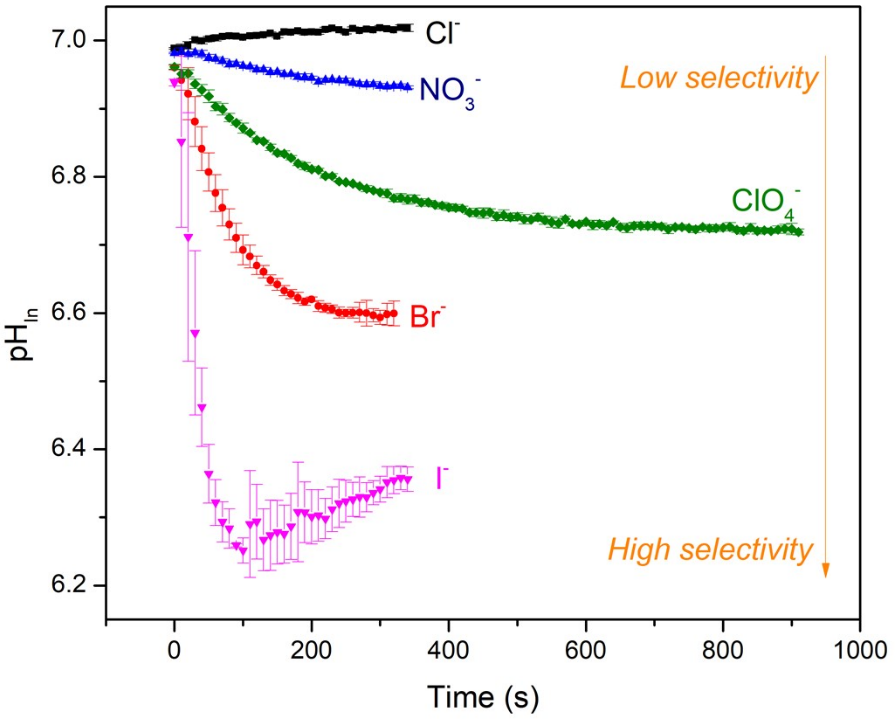

Figure S108. Anion selectivity for complex **1** in the HPTS assay. POPC vesicles were loaded with HPTS in 100 mM NaCl and suspended in an isotonic external solution with Br^-, NO_3^-, I^-, or ClO_4^- anions.

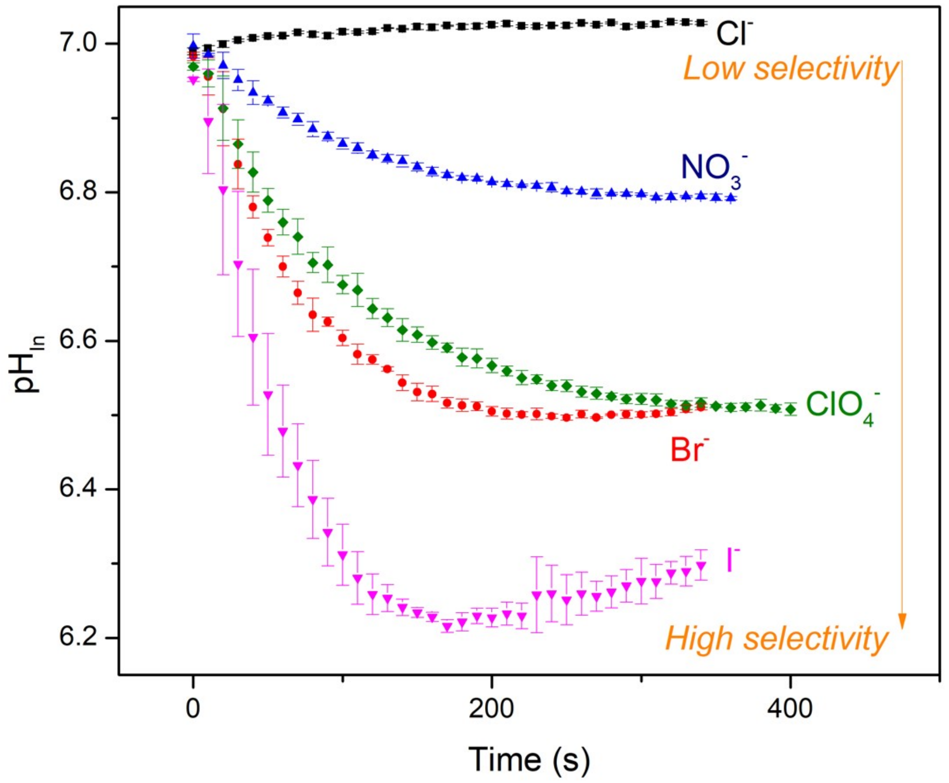

Figure S109. Anion selectivity for complex **2** in the HPTS assay. POPC vesicles were loaded with HPTS in 100 mM NaCl and suspended in an isotonic external solution with Br^-, NO_3^-, I^-, or ClO_4^- anions.

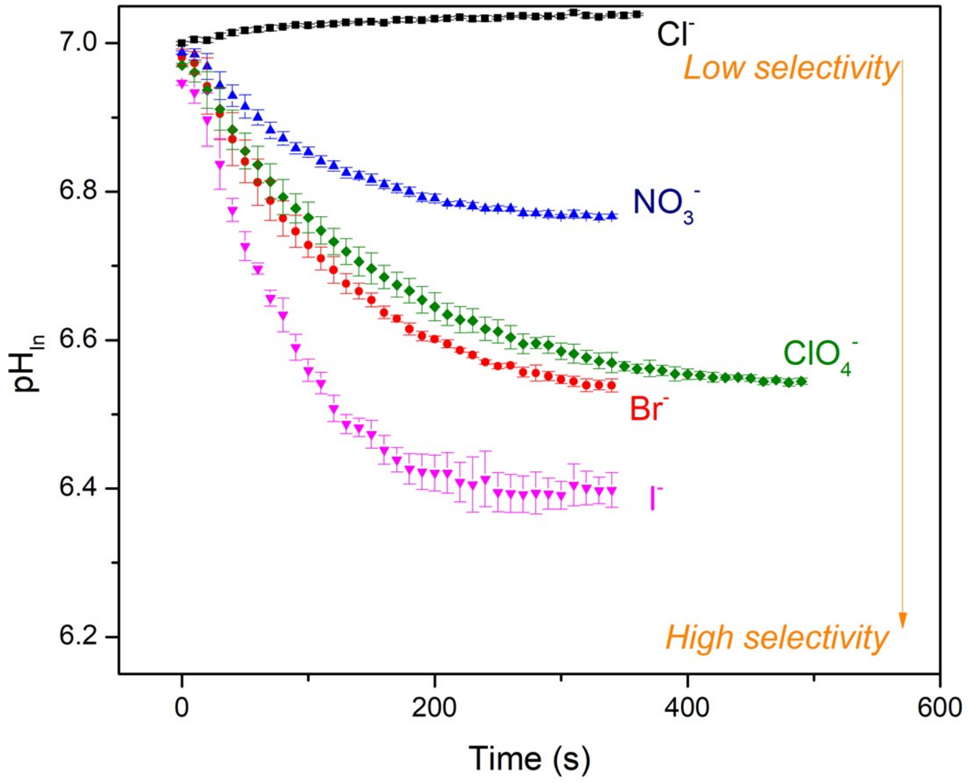

Figure S110. Anion selectivity for complex **3** in the HPTS assay. POPC vesicles were loaded with HPTS in 100 mM NaCl and suspended in an isotonic external solution with Br^-, NO_3^-, I^-, or ClO_4^- anions.

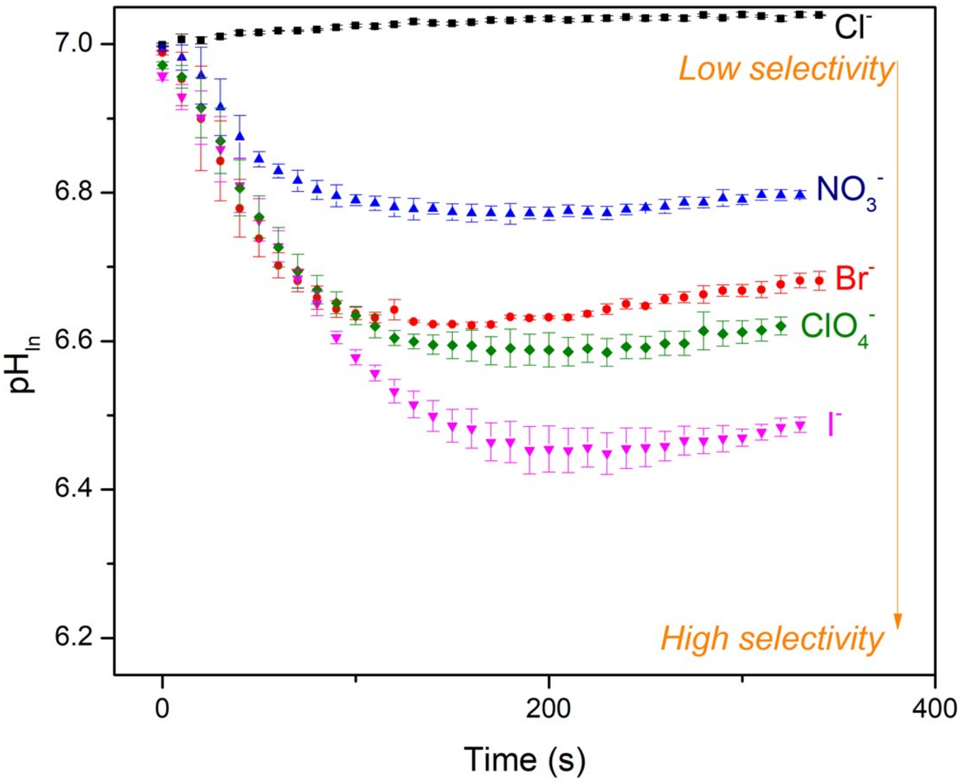

Figure S111. Anion selectivity for complex **4** in the HPTS assay. POPC vesicles were loaded with HPTS in 100 mM NaCl and suspended in an isotonic external solution with Br^-, NO_3^-, I^-, or ClO_4^- anions.

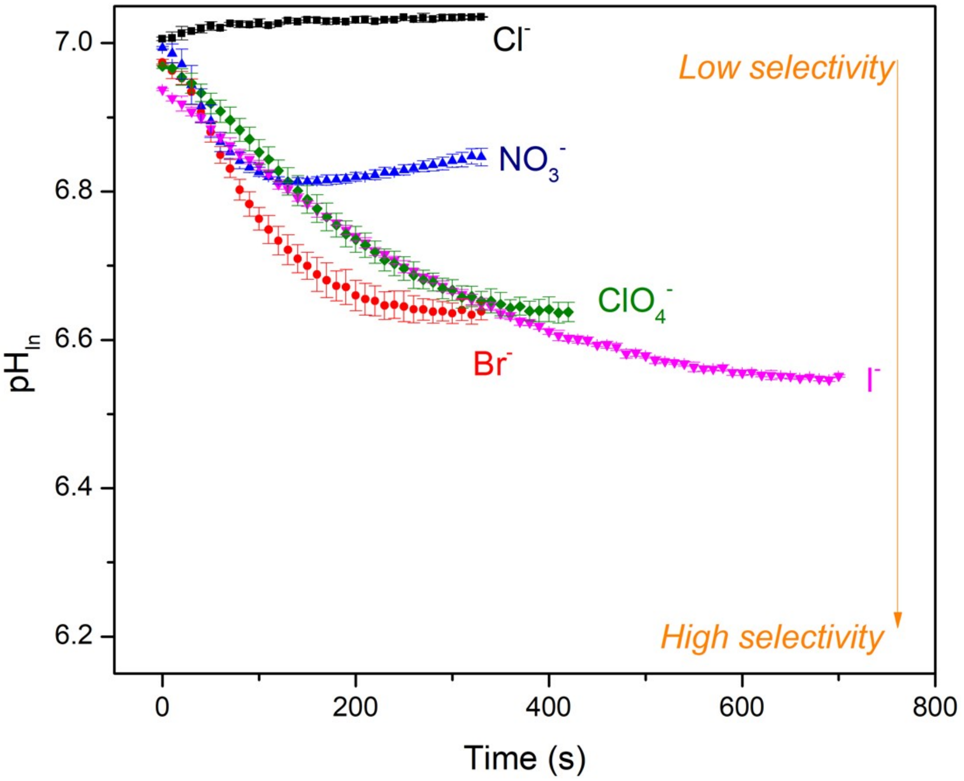

Figure S112. Anion selectivity for complex **5** in the HPTS assay. POPC vesicles were loaded with HPTS in 100 mM NaCl and suspended in an isotonic external solution with Br^-, NO_3^-, I^-, or ClO_4^- anions.

DMSO Stability Studies

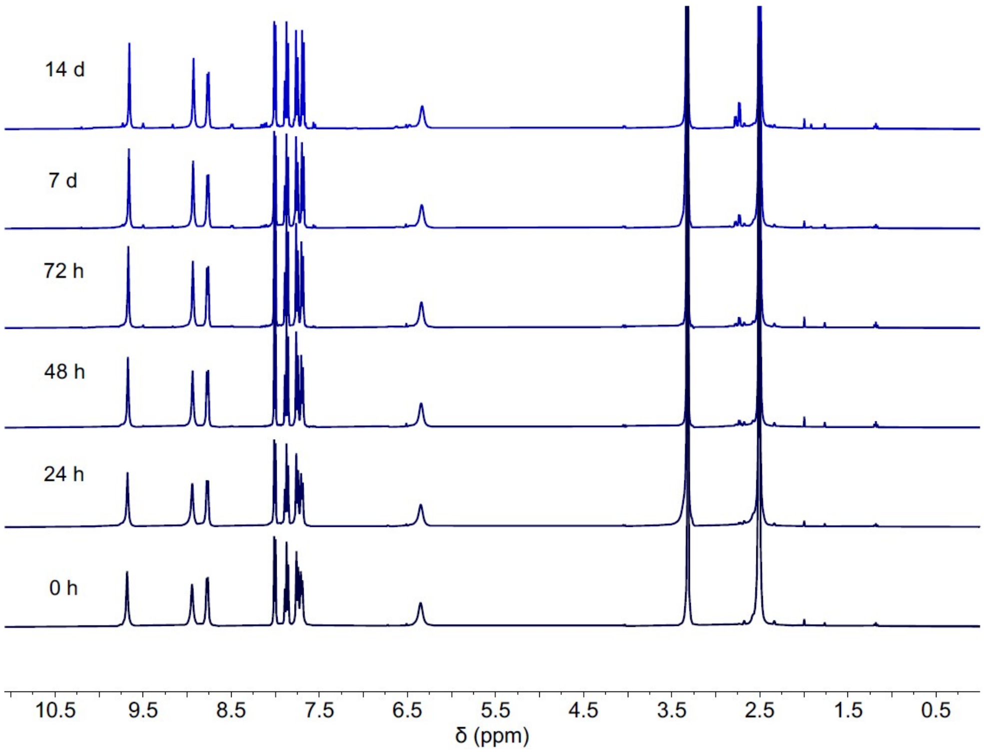

Figure S113. Stack plot of ^{1}H NMR spectra for complex **1** in DMSO-d_6 collected at various timepoints over 14 days.

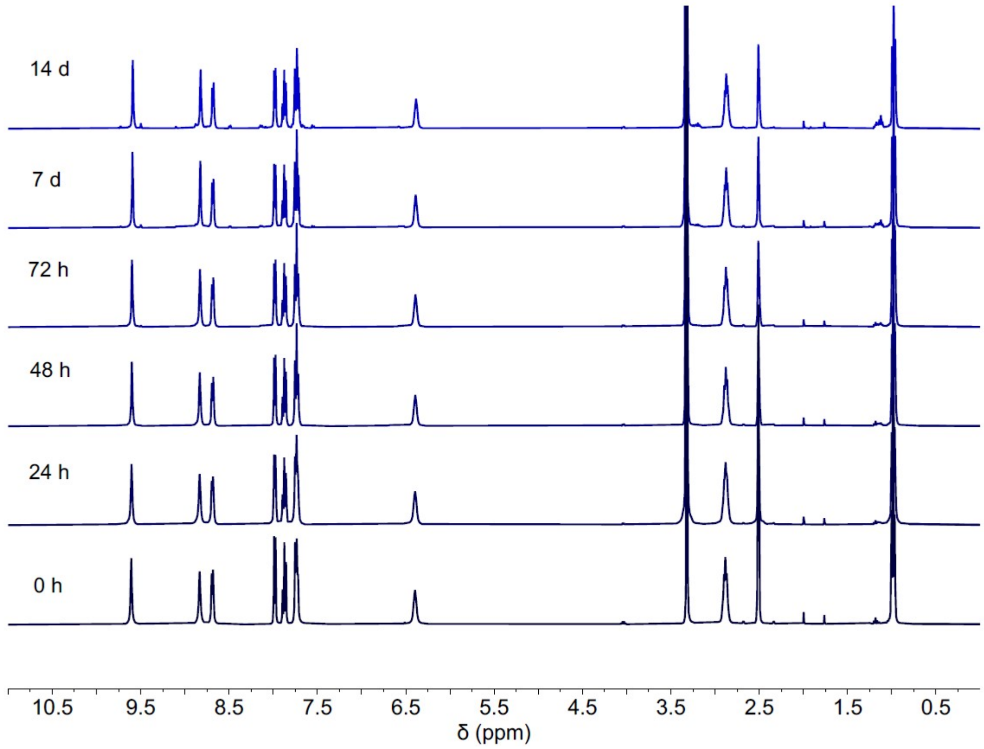

Figure S114. Stack plot of ^{1}H NMR spectra for complex **2** in DMSO-d_6 collected at various timepoints over 14 days.

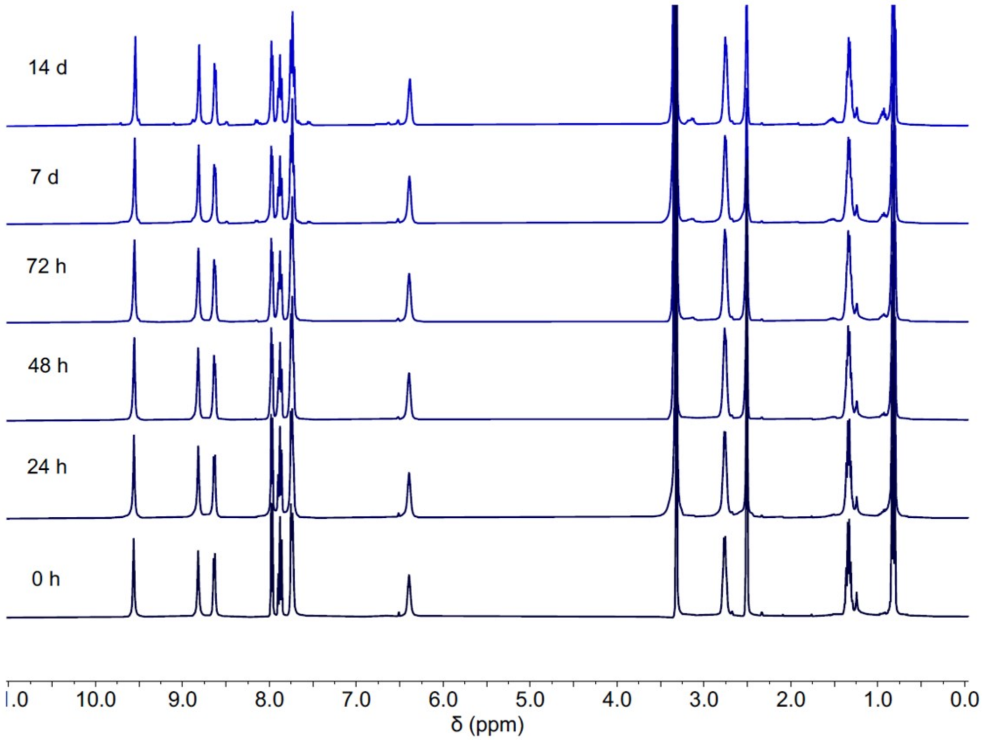

Figure S115. Stack plot of ^{1}H NMR spectra for complex **3** in DMSO-d_6 collected at various timepoints over 14 days.

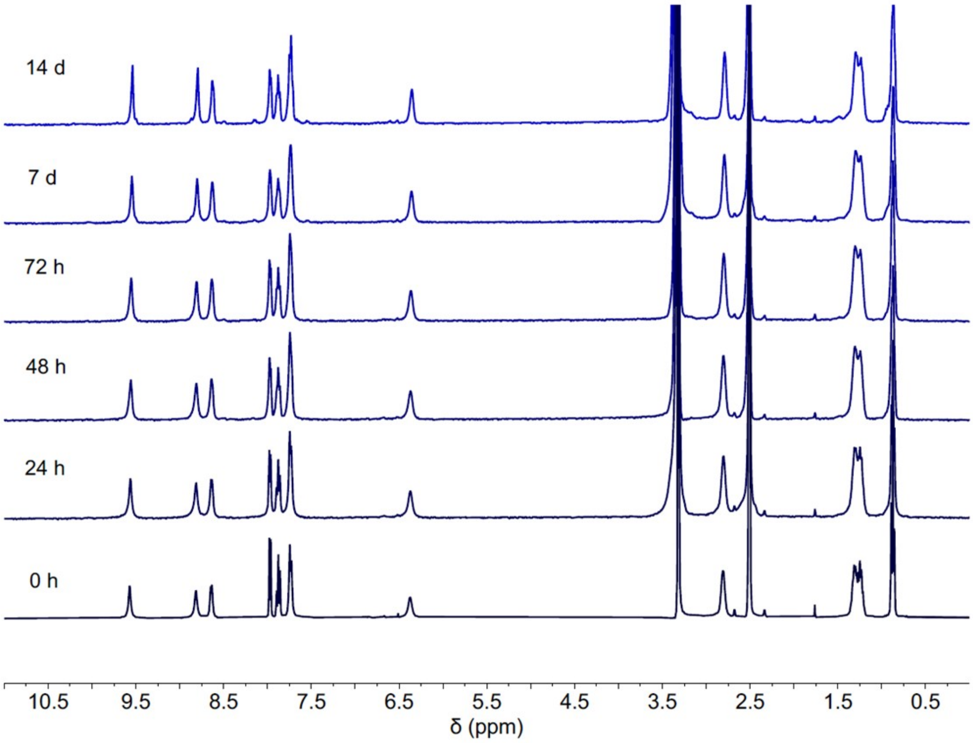

Figure S116. Stack plot of ^{1}H NMR spectra for complex **4** in DMSO-d_6 collected at various timepoints over 14 days.

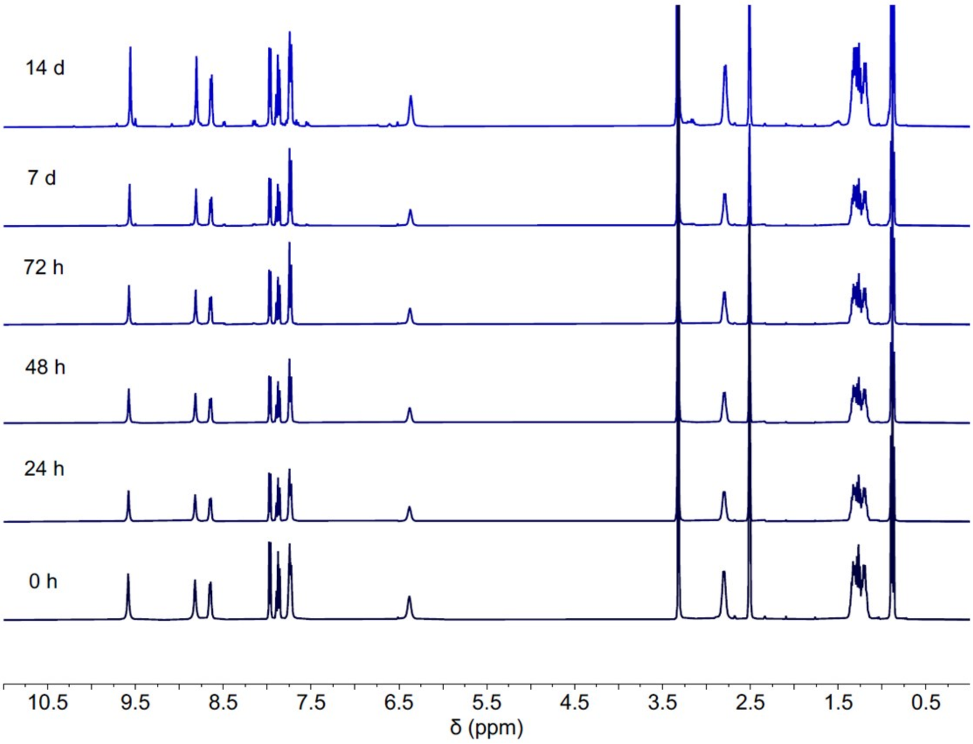

Figure S117. Stack plot of ^{1}H NMR spectra for complex **5** in DMSO-d_6 collected at various timepoints over 14 days.

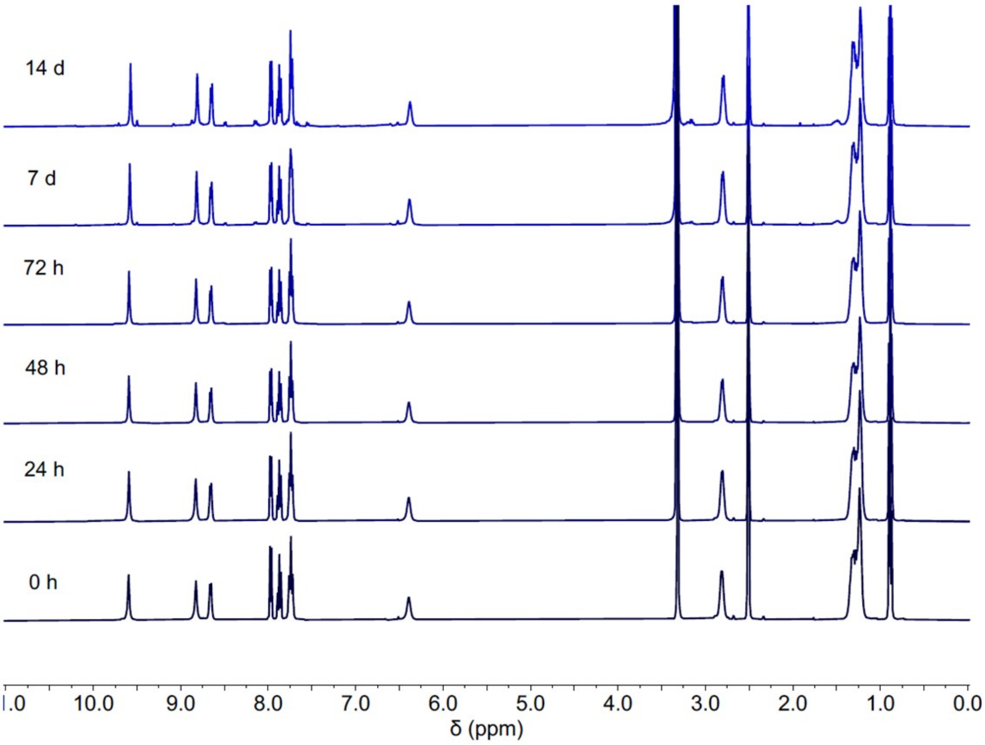

Figure S118. Stack plot of ^{1}H NMR spectra for complex **6** in DMSO-d_6 collected at various timepoints over 14 days.

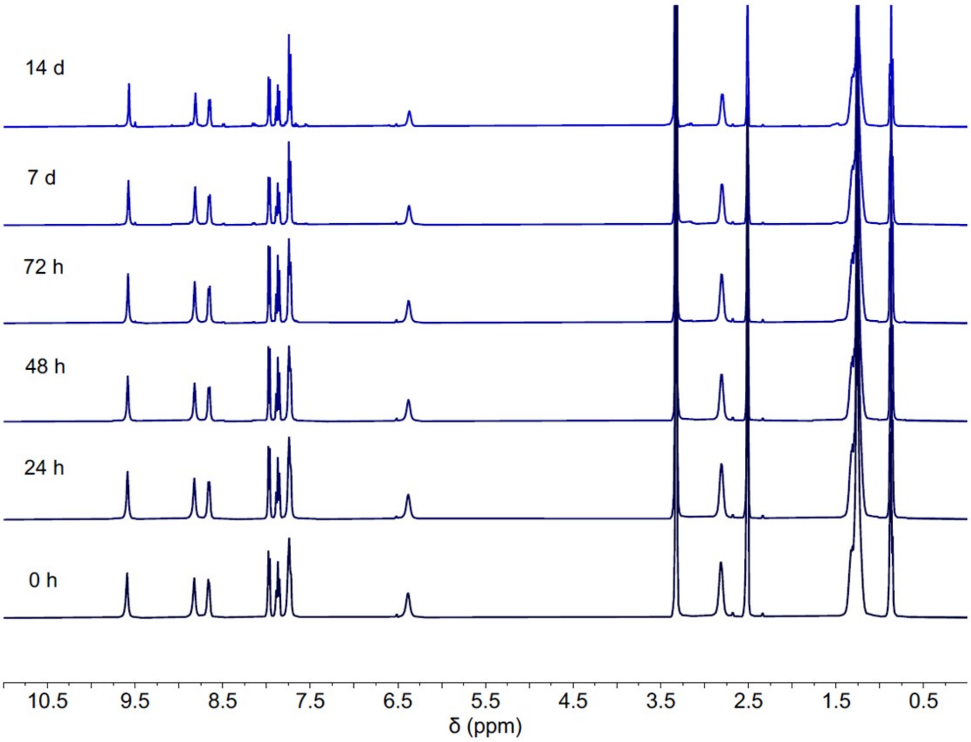

Figure S119. Stack plot of ^{1}H NMR spectra for complex **7** in DMSO-d_6 collected at various timepoints over 14 days.

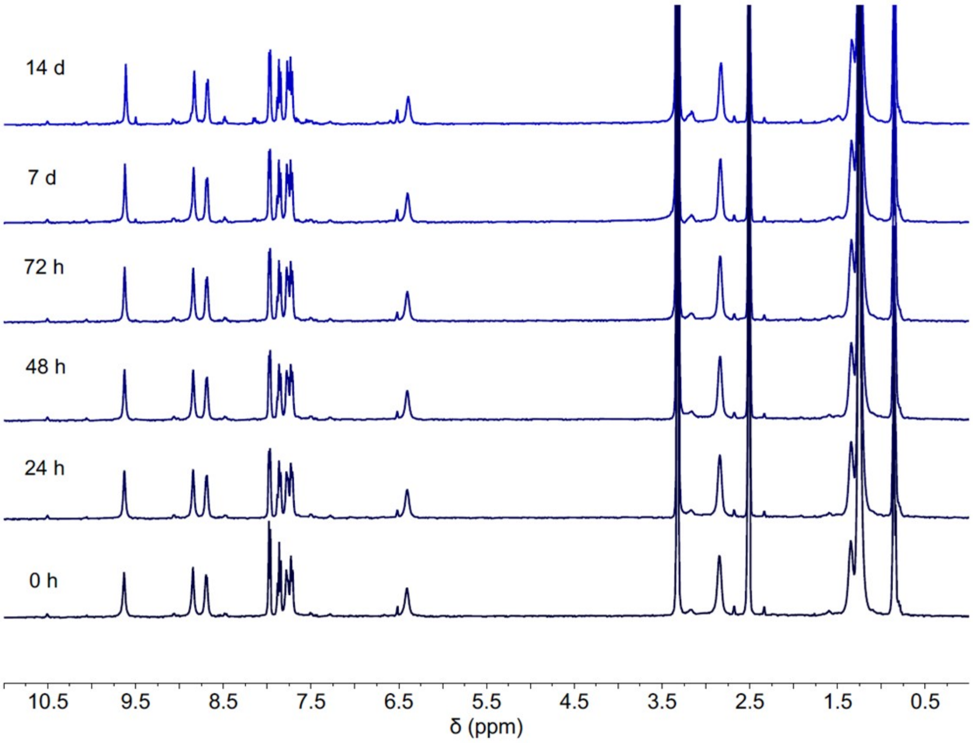

Figure S120. Stack plot of ^{1}H NMR spectra for complex **8** in DMSO-d_6 collected at various timepoints over 14 days.

References

1. http://app.supramolecular.org/bindfit/ (accessed January, 2023).
2. C. R. Bondy, P. A. Gale and S. J. Loeb, Platinum (II) nicotinamide complexes as receptors for oxo-anions, *Chem. Commun.*, 2001, 729-730.
3. L. A. Jowett and P. A. Gale, Supramolecular methods: the chloride/nitrate transmembrane exchange assay, *Supramol. Chem.*, 2019, **31**, 297-312.
4. X. Wu and P. A. Gale, Measuring anion transport selectivity: a cautionary tale, *Chem. Commun.*, 2021, **57**, 3979-3982.
5. I. H. Dissanayake, M. A. Alsherbiny, D. Chang, C. G. Li and D. J. Bhuyan, Antiproliferative effects of Australian native plums against the MCF7 breast adenocarcinoma cells and UPLC-qTOF-IM-MS-driven identification of key metabolites, *Food Biosci.*, 2023, **54**, 102864.
6. K. Jaye, M. A. Alsherbiny, D. Chang, C.-G. Li and D. J. Bhuyan, Mechanistic Insights into the Anti-Proliferative Action of Gut Microbial Metabolites against Breast Adenocarcinoma Cells, *Int. J. Mol. Sci.*, 2023, **24**, 15053.
7. M. A. Alsherbiny, D. J. Bhuyan, I. Radwan, D. Chang and C.-G. Li, Metabolomic identification of anticancer metabolites of Australian propolis and proteomic elucidation of its synergistic mechanisms with doxorubicin in the MCF7 cells, *Int. J. Mol. Sci.*, 2021, **22**, 7840.
8. L. J. Bourhis, O. V. Dolomanov, R. J. Gildea, J. A. Howard and H. Puschmann, The anatomy of a comprehensive constrained, restrained refinement program for the modern computing environment–Olex2 dissected, *Acta Crystallographica Section A: Foundations and Advances*, 2015, **71**, 59-75.
9. O. V. Dolomanov, L. J. Bourhis, R. J. Gildea, J. A. Howard and H. Puschmann, OLEX2: a complete structure solution, refinement and analysis program, *J. Appl. Cryst.*, 2009, **42**, 339-341.
10. G. M. Sheldrick, A short history of SHELX, *Acta Cryst. A: Found. Cryst.*, 2008, **64**, 112-122.
11. T. M. McPhillips, S. E. McPhillips, H.-J. Chiu, A. E. Cohen, A. M. Deacon, P. J. Ellis, E. Garman, A. Gonzalez, N. K. Sauter and R. P. Phizackerley, Blu-Ice and the Distributed Control System: software for data acquisition and instrument control at macromolecular crystallography beamlines, *J. Synchrotron Rad.*, 2002, **9**, 401-406.
12. W. Kabsch, Automatic processing of rotation diffraction data from crystals of initially unknown symmetry and cell constants, *J. Appl. Cryst.*, 1993, **26**, 795-800.
13. G. M. Sheldrick, SHELXT–Integrated space-group and crystal-structure determination, *Acta Crystallographica Section A: Foundations and Advances*, 2015, **71**, 3-8.
14. G. M. Sheldrick, Crystal structure refinement with SHELXL, *Acta Cryst. C: Struct. Chem.*, 2015, **71**, 3-8.
15. P. Thordarson, Determining association constants from titration experiments in supramolecular chemistry, *Chemical Society Reviews*, 2011, **40**, 1305-1323.
